# Nitric oxide can enhance secondary aerosol precursor formation from aromatic carbonyls

**DOI:** 10.1038/s41467-026-72628-w

**Published:** 2026-05-07

**Authors:** Shawon Barua, Avinash Kumar, Prasenjit Seal, Mojtaba Bezaatpour, Sakshi Jha, Nanna Myllys, Siddharth Iyer, Matti Rissanen

**Affiliations:** 1 Aerosol Physics Laboratory, Physics Unit, Faculty of Engineering and Natural Sciences, Tampere University, Tampere, Finland; 2 Department of Chemistry, University of Helsinki, Helsinki, Finland

**Keywords:** Atmospheric chemistry, Physical chemistry

## Abstract

Atmospheric oxidation of aromatic carbonyl compounds in urban environments can lead to highly oxygenated organic molecules (HOMs) that are direct precursors to secondary organic aerosols (SOA). Presence of NO_x_ (NO + NO_2_) is generally assumed to suppress HOM and consequently SOA formation. In this work, experiments with 1 ppm of benzaldehyde, phenylacetaldehyde, and acetophenone show that NO concentrations ranging from 10 ppb to 1 ppm can enhance HOM yields by up to tenfold, with a decreasing trend at above 300 ppb NO. Additionally, HOMs with up to 12 oxygen atoms are observed in sub-second to second timescales, indicating active pathways of rapid oxygen additions following initial oxidation. Quantum chemical calculations are employed to elucidate the oxidation pathways of these carbonyls. The considerable HOM yields and their enhancement in the presence of NO_x_ provide valuable insights into how shifting aromatic and NO_x_ emission trends influence urban air quality and aerosol formation.

## Abstract (web summary)

Aromatic pollutants contribute significantly to the formation of secondary organic aerosol. Here the authors find that limited concentrations of nitric oxide can boost the production of these materials, directly influencing air quality.

## Introduction

Aromatic carbonyls are major air pollutants^1–4^ and are primarily emitted into the atmosphere from incomplete combustion of gasoline fuels^5–7^. They are commonly used as solvents in many industrial processes, are present in consumer products, and are available in indoor air at significant concentrations^8,9^. They are also produced in situ by the atmospheric chemical transformations of other aromatic compounds^10,11^. Aromatic carbonyls feature a reactive carbonyl functional group that can significantly affect the atmospheric oxidation chemistry in comparison to the more abundant alkyl-substituted primary emissions (e.g., toluene and xylene)^12^. The degradation of aromatic carbonyls in the atmosphere is known to be mainly controlled by photolysis and reactions with OH radicals. In the troposphere, the reaction with the OH radical is generally dominant and limits their lifetimes in the range of 10 h to the order of a day. The reaction of aromatic carbonyls with OH radicals is initiated by either the abstraction of an H atom from the substituents of the aromatic ring or by the addition of the OH radical to the aromatic moiety. In aldehydes, the reaction predominantly occurs by the abstraction of the aldehydic H, whereas in ketones, OH addition to the aromatic ring is competitive with the H abstraction path^3,4,13^. Either of the two OH reactions lead to more reactive intermediates that can potentially oxidize further in the atmosphere.

It has been well established that highly oxygenated organic molecules (HOMs) constitute a major part of atmospheric condensable vapors and significantly contribute to the formation of secondary organic aerosol (SOA). Atmospheric oxidation of volatile organic compounds (VOCs) proceeds via alkyl peroxy radical (RO_2_) intermediates, and the fate of the RO_2_ radicals depends on their reactivity with a handful of reaction partners, including other RO_2_, HO_x_ (OH and HO_2_), and NO_x_^14^. Several RO_2_ radicals can rapidly increase the oxygen content and form HOM by the process of autoxidation, usually in seconds or in certain cases even in sub-second timescales^15,16^. Autoxidation refers to the sequential rearrangement of RO_2_ radicals, followed by the addition of O_2_ molecules and the repetition of the process multiple times, leading to HOM. Here, we refer to HOM as the oxidation products that contain ≥ 6 O atoms in their chemical composition^17^.

There are investigations exploring the gas phase oxidation chemistry of aliphatic and aromatic carbonyls in the literature^3,4,18–22^. Recently, Barua et al.^18^ showed that a representative aliphatic aldehyde, hexanal, can rapidly form HOMs upon oxidation by the OH radical. The rapid rearrangement (rate coefficient *k *≈ 0.2 s^-1^) of an acyl peroxy radical (RC(O)OO) intermediate is crucial for this reaction chain propagation. Previous studies on aromatic carbonyls mostly explored the initial oxidation step, while a computational study on the OH oxidation of acetophenone investigated reaction routes leading to the addition of at most six oxygen atoms to the carbon structure^4^. A key step in the formation of HOM from the aromatic toluene was recently reported to be a fast ring opening of an ipso bicyclic peroxy radical (ipso-BPR) intermediate (initial OH addition to the methyl-substituted carbon). This molecular rearrangement reaction has a rate coefficient close to 1 s^-1^ for the toluene ipso-BPR, which outcompetes other sink reactions of this RO_2_, and triggers the rapid functionalization forming HOMs in sub-second timescales^16^. Aromatic carbonyls have similar ipso-BPR pathways available to them, which may also involve molecular rearrangement and lead rapidly to HOM. To the best of our knowledge, these have not been previously explored.

In urban environments, nitrogen oxides (NO_x_ = NO + NO_2_) account for a major share of reactive trace gases in the atmosphere, driven by anthropogenic combustion emissions. During the daytime, NO_x_ controls the oxidation cycle of VOCs by reaction with RO_2_ radicals and thus greatly influences the HOM formation pathways. It is widely accepted that high NO_x_ suppresses HOM formation^23–27^ and thereby the SOA yield. In this process, the reduction of SOA yield is significantly influenced by the suppression of HOM accretion products (RO_2_ + RO_2_ → ROOR), which are known to be very condensable^26,28^. However, prior studies^29–32^ have shown that in select cases low NO_x_ concentrations can enhance the formation of HOMs via alkoxy radical (RO) mediated chemistry in alkane and alkenes. While NO can terminate RO_2_ autoxidation by forming closed-shell organonitrates (ONs), it also forms more reactive RO radicals (RO_2_ + NO → RO + NO_2_) that undergo fast isomerization followed by O_2_ addition and regenerate RO_2_. The new RO_2_ with one more O atom than the preceding RO_2_ can further undergo autoxidation and enhance HOM formation^32^. Moreover, Wang et al.^29^ have reported that HOM yields increased with the increase of NO (0.1–10 ppb initial NO) during the oxidation of several alkanes that are key components of urban air. In the VOC systems with low dimer to monomer product ratios, the suppression effect of NO_x_ on SOA by the expense of only accretion products should be less. Instead, with a potential enhancement of monomeric HOMs and highly functionalized HOM-ONs in the presence of NO, a corresponding increase in SOA yield could be expected^33,34^. Yet, at very high NO_x_ levels, HOM formation appears inevitably suppressed by autoxidation, terminating early at low oxygen to carbon ratios.

Pullinen et al.^26^ showed that the effective uptake coefficients (*γ*_*eff*_) of HOMs and HOM-ONs, arising from the same HOM peroxy radicals, on particles are similar in monoterpene photooxidation. Both with more than six O atoms in the precursor peroxy moiety condense by about 50% (*γ*_*eff*_ of about 0.5) to form SOA, whereas those with more than eight O atoms condense by about 100%. Therefore, observing the abundance of HOM-ONs in VOC oxidation processes is of importance.

In this work, we experimentally studied the OH-initiated autoxidation of three abundant aromatic carbonyl systems, benzaldehyde (PhCHO), phenylacetaldehyde (PhCH_2_CHO), and acetophenone (PhCOCH_3_) in short and long reaction time experiments. The reactions were studied in the absence and presence of NO at variable concentrations (10 ppb–1 ppm) in a flow reactor. Using state-of-the-art mass spectrometry, we show that HOM yields can increase by up to a factor of 10 at an initial NO-to-carbonyl concentration ratio of 1:10. We also employed quantum-chemical calculations to explore potential reaction mechanisms for HOM formation and verified them using kinetic simulations. Finally, we theoretically estimated the saturation concentrations of the oxidation products based on their molecular structures to assess their tendency to form ambient SOA.

## Results and discussion

### Detection of HOM in sub-second to high residence time experiments

**Fig. 1:**
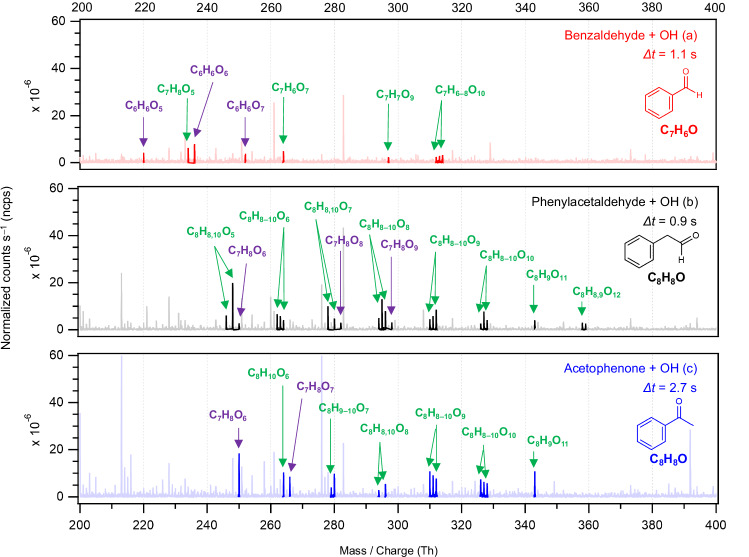
Nitrate chemical ionization mass spectra of OH-initiated oxidation of aromatic carbonyls (without NO), showing the formation of highly oxygenated organic molecules in different residence times (Δ*t*). Experiment with benzaldehyde (Δ*t* = 1.1 s) is represented in red (**a**), with phenylacetaldehyde (Δ*t* = 0.9 s) in black (**b**), and with acetophenone (Δ*t* = 2.7 s) in blue (**c**). In the product peak labels, the NO_3_^–^ is excluded from their corresponding adduct compositions. Non-fragmented products (C_x_) are labelled in green while fragmented products (C_x-1_) are labelled in purple. Background signals are shaded in light colors to distinguish them from the target peaks.

In this section, we discuss the formation of HOMs observed in variable residence time experiments of OH initiated aromatic carbonyls oxidation without the presence of NO. Figure 1 shows how early HOMs formed in the oxidation of different aromatic carbonyls conducted in the flow reactor system. We could detect HOMs in sub-second (Δ*t* = 0.9 s) reaction time in phenylacetaldehyde (PhCH_2_CHO, C_8_H_8_O) oxidation (Fig. 1b), whereas HOMs were detected as early as within 1.1 s and 2.7 s in benzaldehyde (PhCHO, C_7_H_6_O) and acetophenone (PhCOCH_3_, C_8_H_8_O) oxidation, respectively (Fig. 1a,c). We did not observe any HOM formation from the oxidation of PhCHO and PhCOCH_3_ in sub-second timescale. In Fig. 1b, we can see that oxidation products, mostly HOMs, with a number of O atoms ranging from 5 to 12, are present within 0.9 s of reaction time in PhCH_2_CHO oxidation. These include alkyl (or acyl) peroxy radicals C_8_H_9_O_z_ (z = 6, 8–12) as well as closed-shell products C_8_H_8,10_O_5–﻿11_. The PhCHO oxidation spectrum (Fig. 1a) shows relatively fewer products with up to O_10_ HOMs within 1.1 s, while we observed HOMs up to 11 O atoms in PhCOCH_3_ oxidation (Fig. 1c) only in a somewhat longer reaction time (2.7 s) experiment.

**Fig. 2:**
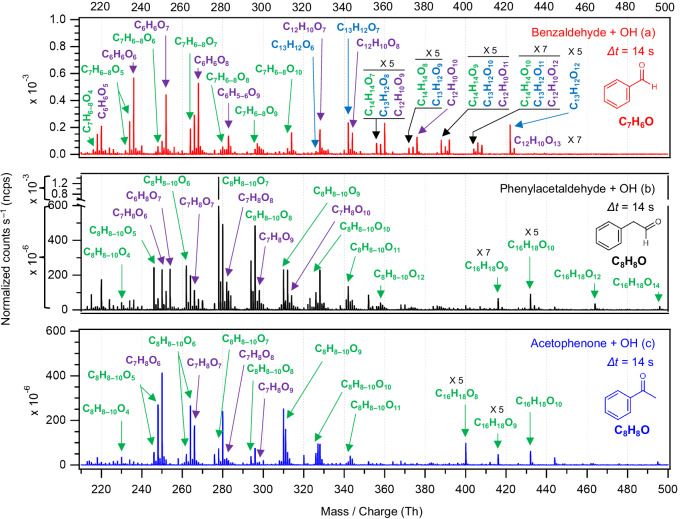
Nitrate chemical ionization mass spectra of OH initiated oxidation of aromatic carbonyls (without NO) in 14 s residence time. Experiment with benzaldehyde is represented in red (**a**), with phenylacetaldehyde in black (**b**), and with acetophenone in blue (**c**). In the product peak labels, the NO_3_^–^ is excluded from their corresponding adduct compositions. Non-fragmented products (C_x_ and C_2x_) are labelled in green while fragmented products (C_x–1,2_ and C_2(x–1)_) are labelled in purple. The black arrows in panel (**a**) with multiple compositions (labelled in green, blue, and purple) represent product peaks separated by two mass units starting from the arrow-pointing positions. Several accretion product signals are multiplied by 5–7 times for visibility.

In high residence time (14 s) experiments, we observed a wide range of oxidation products spanning from monomeric HOMs to accretion products from all the studied aromatic carbonyl systems (Fig. 2 and Table 1). The benzaldehyde (PhCHO, C_7_H_6_O) oxidation spectrum represents varieties of product signals including HOM monomers up to 10 O atoms and accretion products up to 13 O atoms. The spectrum shows that the higher intensity signals are dominated by the C_6_-oxidation products which contain one less C atom than the parent precursor C_7_H_6_O in the monomeric regime. The intensities of the fragmented products C_6_H_6_O_5–﻿9_ are higher than the neighboring non-fragmented products C_7_H_8_O_4–﻿8_ (Fig. 2a). Among the accretion products (C_12_H_10_O_7–﻿13_, C_13_H_12_O_6–﻿12_, and C_14_H_14_O_7–﻿10_), the C_12–13_ compounds dominate the high intensity product peaks and the chemical compositions of all these products suggest that they might be originating from different combinations of the monomeric C_6_- and C_7_-RO_2_ intermediates. On the other hand, both phenylacetaldehyde (PhCH_2_CHO, C_8_H_8_O) and acetophenone (PhCOCH_3_, C_8_H_8_O) spectra show that the higher intensity signals are dominated by the non-fragmented products C_8_H_8–﻿10_O_4–﻿12_ except for a fragmented product C_7_H_8_O_6_ (m/z 250.021) with higher intensity than the non-fragmented neighbor C_8_H_10_O_5_ (m/z 248.041) in the acetophenone spectrum (Fig. 2b–c). Besides, HOM monomers up to 12 O atoms and accretion products up to 14 O atoms were observed in phenylacetaldehyde oxidation, whereas acetophenone oxidation produced HOM monomers up to 11 O atoms along with the accretion products with a maximum of 10 O atoms (C_16_H_18_O_8–10_).

**Table 1:** Chemical compositions of the oxidation products identified in OH initiated oxidation of PhCHO, PhCH_2_CHO, and PhCOCH_3_

Aromatic carbonyl systems and their oxidation products		
PhCHO (C_7_H_6_O)	PhCH_2_CHO (C_8_H_8_O)	PhCOCH_3_ (C_8_H_8_O)
**Monomeric products**^**†**^		
C_7_H_6–8_O_4_ (216.015, 217.023, 218.031)	C_8_H_8–10_O_4_ (230.031, 231.039, 232.046)	C_8_H_8–10_O_4_ (230.031, 231.039, 232.046)
C_7_H_6–8_O_5_ (232.010, 233.018, 234.026) C_6_H_5–6_O_5_ (219.002, 220.010)	C_8_H_8–10_O_5_ (246.026, 247.033, 248.041) C_7_H_8_O_5_ (234.026) C_6_H_6_O_5_ (220.010)	C_8_H_8–10_O_5_ (246.026, 247.033, 248.041) C_7_H_8_O_5_ (234.026) C_6_H_6_O_5_ (220.010)
C_7_H_6–8_O_6_ (248.005, 249.013, 250.021) C_6_H_5–6_O_6_ (234.997, 236.005)	C_8_H_8–10_O_6_ (262.021, 263.028, 264.036) C_7_H_8_O_6_ (250.021) C_6_H_6_O_6_ (236.005)	C_8_H_8–10_O_6_ (262.021, 263.028, 264.036) C_7_H_8_O_6_ (250.021) C_6_H_6_O_6_ (236.005)
C_7_H_6–8_O_7_ (264.000, 265.008, 266.015) C_6_H_5–6_O_7_ (250.992, 252.000)	C_8_H_8–10_O_7_ (278.015, 279.023, 280.031) C_7_H_8_O_7_ (266.015) C_6_H_8_O_7_ (254.015)	C_8_H_8–10_O_7_ (278.015, 279.023, 280.031) C_7_H_8_O_7_ (266.015) C_6_H_6_O_7_ (252.000)
C_7_H_6–8_O_8_ (279.995, 281.003, 282.010) C_6_H_5–6_O_8_ (266.987, 267.995)	C_8_H_8–10_O_8_ (294.010, 295.018, 296.026) C_7_H_8_O_8_ (282.010) C_6_H_8_O_8_ (270.010)	C_8_H_8–10_O_8_ (294.010, 295.018, 296.026) C_7_H_8_O_8_ (282.010)
C_7_H_6–8_O_9_ (295.990, 296.997, 298.005) C_6_H_5–6_O_9_ (282.982, 283.990)	C_8_H_8–10_O_9_ (310.005, 311.013, 312.021) C_7_H_8_O_9_ (298.005)	C_8_H_8–10_O_9_ (310.005, 311.013, 312.021) C_7_H_8_O_9_ (298.005)
C_7_H_6–8_O_10_ (311.985, 312.992, 314.000)	C_8_H_8–10_O_10_ (326.000, 327.008, 328.016) C_7_H_8_O_10_ (314.000)	C_8_H_8–10_O_10_ (326.000, 327.008, 328.016)
	C_8_H_8–10_O_11_ (341.995, 343.003, 344.011) C_8_H_8–10_O_12_ (357.990, 358.998, 360.006)	C_8_H_8–10_O_11_ (341.995, 343.003, 344.011)
**Accretion products**		
C_12_H_10_O_7–13_ (328.031, 344.026, 360.021, 376.016, 392.011, 408.006, 424.001) C_13_H_12_O_6–12_ (326.052, 342.047, 358.042, 374.037, 390.031, 406.026, 422.021) C_14_H_14_O_7–10_ (356.062, 372.057, 388.052, 404.047)	C_16_H_18_O_9_ (416.083) C_16_H_18_O_10_ (432.078) C_16_H_18_O_12_ (464.068) C_16_H_18_O_14_ (496.058)	C_16_H_18_O_8_ (400.089) C_16_H_18_O_9_ (416.083) C_16_H_18_O_10_ (432.078)

^†^Different oxygenated product groups (O_4_ to O_12_) are separated by horizontal lines for readability.

The products are detected as their NO_3_^–^ adducts and the corresponding mass to charge ratios (m/z) measured by the nitrate CI-APi-TOF are given in the parenthesis. Residence time = 14 s.

The spectra (Figs. 1, 2) of all the three aromatic carbonyls are populated with non-fragmented products with a maximum of two more H atoms in their compositions (C_x_H_y+2_O_z_) compared to their parent precursors (C_x_H_y_O) which suggests that the oxidation initiated by the addition of OH radical to the aromatic moiety is significant. This is because the OH addition is the only channel that can give such amounts of H atoms (H_8_ in benzaldehyde, and H_10_ in phenylacetaldehyde and acetophenone) corresponding to the formation of closed-shell hydroperoxide (via RO_2_ + HO_2_ → ROOH + O_2_) or alcohols (via the Russell mechanism, RO_2_ + R’O_2_ → ROH + R’_–H_C = O + O_2_). It is also important to note that in OH addition channel the first alkyl peroxy radical being a C_x_H_y+1_O_4_ system, unimolecular autoxidation process can predominantly produce an even number of oxygen-containing products if it outcompetes over any possible bimolecular reactions.

Figure 2 shows that within the given reaction time, the aromatic carbonyl oxidation spectra have a well-defined odd-even oxygen distribution in the identified product peaks including RO_2_ radicals. This implies that alkyl (or acyl) peroxy bimolecular reaction steps producing more reactive alkoxy (or acyloxy) radicals (RO_2_ + RO_2_ → 2 RO + O_2_) are likely playing an important role during oxidation chain propagation to produce the observed odd oxygen-containing products in the OH addition channel (Supplementary Figs. 13, 14). However, one should also remember that the autoxidation-produced even number of oxygen-containing RO_2_ can also contribute to the formation of odd-O closed-shell products via the Russell mechanism. In Fig. 2c, we can see that the odd oxygen products (C_8_H_10_O_5,7,9_) dominate the acetophenone oxidation spectrum. This indicates that the autoxidation process that requires H-shifts in the early RO_2_ intermediates to produce even oxygen products seems inefficient, and the mechanism seems to require an alkoxy step in the path to HOM formation. The dominant C_8_H_8_O_7_ HOM in the phenylacetaldehyde oxidation spectrum (Fig. 2b) suggests that the autoxidation-produced alkyl peroxy intermediate C_8_H_9_O_6_ is very likely in competition with a bimolecular reaction step (RO_2_ + RO_2_ → 2 RO + O_2_) producing alkoxy intermediate C_8_H_9_O_5_ which following a rearrangement can grab one O_2_ molecule and subsequently continue reaction chain propagation unimolecularly yielding more products with odd number of oxygen atoms (Supplementary Figs. 13, 14).

While the OH addition pathway is crucial for generating non-fragmented oxidation products (C_x_H_y+2_O_z_), it represents a minor reaction channel. The dominant H-abstraction pathway, on the other hand, can primarily lead to the formation of fragmented products (C_x–1,2_) observed in the oxidation of the studied aromatic carbonyls. The mechanistic details of the early oxidation steps of the aromatic carbonyls are shown in Supplementary Figs. 3–4 while a possible generic reaction route of the autoxidation process leading to the formation of HOM is detailed in the subsequent discussion. It should be emphasized that the possibility of any fragmentation reactions along the autoxidation chain propagation via the OH addition channel cannot be ruled out while accounting for the observed fragmented products. The mechanisms of formation of such products, e.g., C_x-1_H_y_O_6_ (Figs. 1, 2 – C_6_H_6_O_6_ in panel (a), and C_7_H_8_O_6_ in both panels (b) and (c)) are shown in Supplementary Figs. 18–20. Overall, the oxidation product distribution as well as the intensities of the product peaks in Fig. 2 reveal that the reactivity of PhCH_2_CHO is likely to be the highest and that of PhCOCH_3_ is the lowest towards the OH-initiated autoxidation of the studied aromatic carbonyls. This observation was verified by the low residence time experiments (Fig. 1), where the oxidation of PhCH_2_CHO produced HOMs even in the sub-second timescale.

### Enhancement of HOM yields in presence of NO

**Fig. 3:**
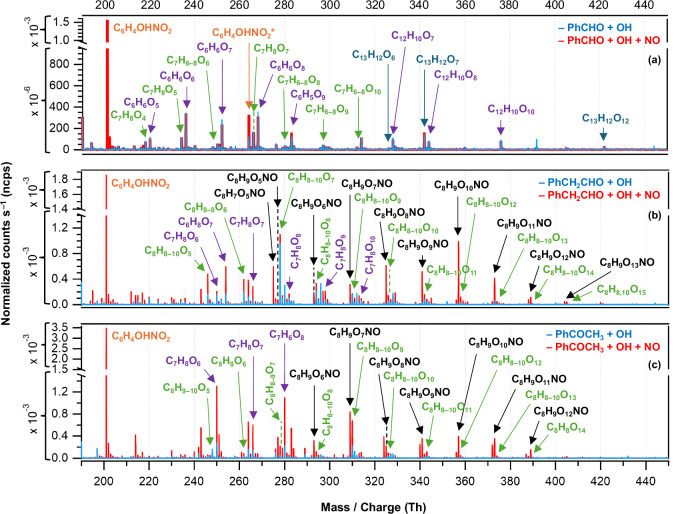
Overlaid nitrate chemical ionization mass spectra of OH initiated oxidation of aromatic carbonyls without (in blue) and with the presence of 100 ppb of NO (in red). The panels **a**, **b**, and **c** represent the experiments with benzaldehyde (PhCHO, C_7_H_6_O), phenylacetaldehyde (PhCH_2_CHO, C_8_H_8_O), and acetophenone (PhCOCH_3_, C_8_H_8_O), respectively. In the product peak labels, the NO_3_^–^ is excluded from their corresponding adduct compositions. The non-fragmented (C_x_) and fragmented (C_x-1,2_) products are labelled in green and purple, respectively, while the organonitrates (in the presence of NO) are labelled in black color. Nitrophenols (C_6_H_4_OHNO_2_) which formed in the presence of NO with the highest intensities are labelled in orange color. Label C_6_H_4_OHNO_2_* indicates the adduct with HNO_3_NO_3_^–^. Reaction time, Δ*t* = 14 s.

**Fig. 4:**
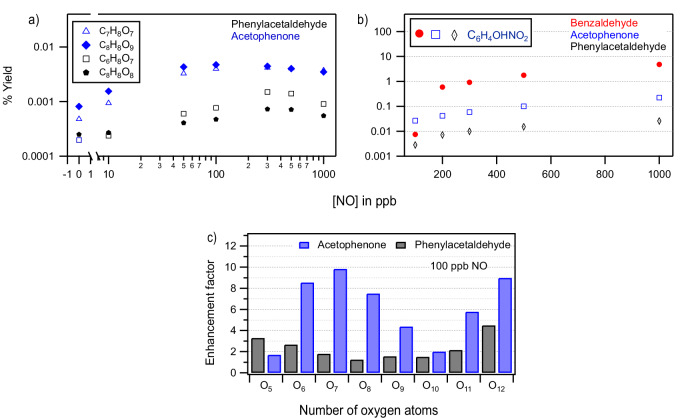
Enhancement of highly oxygenated organic molecule (HOM) yields in presence of NO during the OH initiated oxidation of phenylacetaldehyde (black) and acetophenone (blue). **a** HOM yields detected by nitrate chemical ionization mass spectrometer as a function of NO concentration, shown in a logarithmic scale. Several representative HOMs, including both non-fragmented C_8_ and fragmented C_6–7_ HOMs, are shown in filled and open markers, respectively. **b** The yield of nitrophenol (C_6_H_4_OHNO_2_) as a function of NO concentration in all the aromatic carbonyl systems studied, including benzaldehyde, is shown in red. Note the logarithmic scale on the y-axis. **c** Total yield enhancement of oxygenated products grouped by different oxygen numbers in the presence of 100 ppb NO. Reaction time, Δ*t* = 14 s. Source data are provided as a Source Data file.

The aromatic carbonyl OH oxidation experiments in the presence of NO showed a significant increase in HOM signal intensities and corresponding yields except for PhCHO (Figs. 3 and 4a below). Interestingly, highly oxygenated organonitrates were observed to form only in PhCH_2_CHO and PhCOCH_3_ but not in PhCHO. In the experiments without and with NO, the monomeric HOMs evolved from O_12_ to O_15_ in PhCH_2_CHO and from O_11_ to O_14_ in PhCOCH_3_. However, the intensities of the HOM accretion products decreased as expected. To compare the HOM enhancement factor among the three aromatic carbonyls, the oxidation mass spectra in the presence of 100 ppb NO are highlighted in this section. In the PhCHO spectrum (Fig. 3a), we can see almost no enhancing effect (except a little increase in the C_6_H_5_O_9_ signal) in HOMs but a significant increase in the intensities of nitrophenol (C_6_H_4_OHNO_2_) signals at m/z 201 and 264 corresponding to its clusters with NO_3_^–^ and HNO_3_NO_3_^–^ respectively. The likely formation process of nitrophenol from the OH oxidation of benzaldehyde in the presence of NO_x_ has been described by Calvert et al.^35^ The mechanism is associated with the fast aldehydic H-abstraction by OH which further involves a CO_2_ loss from the benzolyloxy radical intermediate and finally a reaction between the benzoxy radical and NO_2_ to form nitrophenol (Supplementary Fig. 5). In our experiments, although we injected only NO in the flow reactor, NO_2_ is continuously produced by the reaction of NO with O_3_ (NO + O_3_ → NO_2_ + O_2_) and with RO_2_ (NO + RO_2_ → NO_2_ + RO). The time series of NO and NO_2_ are shown in Supplementary Fig. 27. The prompt nitrophenol formation process is likely taking over the influence of NO on HOM distribution resulting very little to no enhancement in this case. Figure 4b shows that the yield of nitrophenol is roughly one and two orders of magnitude higher for benzaldehyde than that of acetophenone and phenylacetaldehyde, respectively, during NO injection steps between 200 ppb and 1 ppm. The formation of nitrophenol from phenylacetaldehyde and acetophenone is also expected to proceed via H-abstraction channel followed by two carbon loss along the reaction path. This implies that along this channel, the studied aromatic carbonyl systems can undergo fragmentation reactions early in the oxidation process while keeping the aromatic ring intact. The subsequent reactions may also lead to the formation of observed C_x–1,2_ products.

On the other hand, both phenylacetaldehyde (PhCH_2_CHO) and acetophenone (PhCOCH_3_) showed a significant increase in the C_x(=8)_ and C_x–1(=7)_ HOM yields with the latter one giving the higher yields for almost all the oxygenated product groups O_6_–O_12_ (see Figs. 3b–c and 4) at 100 ppb of NO. In Fig. 4c, the enhancement factor (F) was calculated by taking the ratio of cumulative yields of C_6–8_H_8–10_O_z_ grouped by the number of oxygen atoms of the oxygenated products measured in the experiments with and without the presence of NO.

We can see that the yield enhancement factor is always higher for the HOMs derived from PhCOCH_3_ (with the highest F = 9.8 for O_7_ HOMs) than PhCH_2_CHO except for O_5_ products where the factor is higher for PhCH_2_CHO (F = 3.3) than PhCOCH_3_ (F = 1.7). The overall higher enhancement factor for PhCOCH_3_ HOMs compared to that of PhCH_2_CHO indicates that the autoxidation in PhCOCH_3_ is slower, allowing enhanced interaction of NO with the peroxy radial intermediates to produce more reactive alkoxy (or acyloxy) radicals followed by a subsequent increase in HOM turnover. In contrast, a lower enhancement factor for PhCH_2_CHO implies that the autoxidation is relatively faster than PhCOCH_3,_ limiting the influence of NO in the HOM formation process. An overall faster autoxidation, along the path to HOMs, of PhCH_2_CHO compared to PhCOCH_3_ is also supported by computationally calculated reaction rate coefficients that are discussed in the next section. Moreover, in the experiments with NO, the prominent signals of organonitrates (C_8_H_9_O_z_ + NO → C_8_H_9_O_z_NO) provide strong evidence in favor of OH addition channel as the major contributor to HOM via the formation of the initial C_8_H_9_O_4_ (C_x_H_y+1_O_4_) peroxy radicals in PhCH_2_CHO and PhCOCH_3_ oxidation. More insights into the abundance of HOMs under varying NO concentrations with focus on closed-shell C_x_H_y+2_O_z_ products are discussed in Supplementary Section S12.

### Mechanistic insight into HOM

**Fig. 5:**
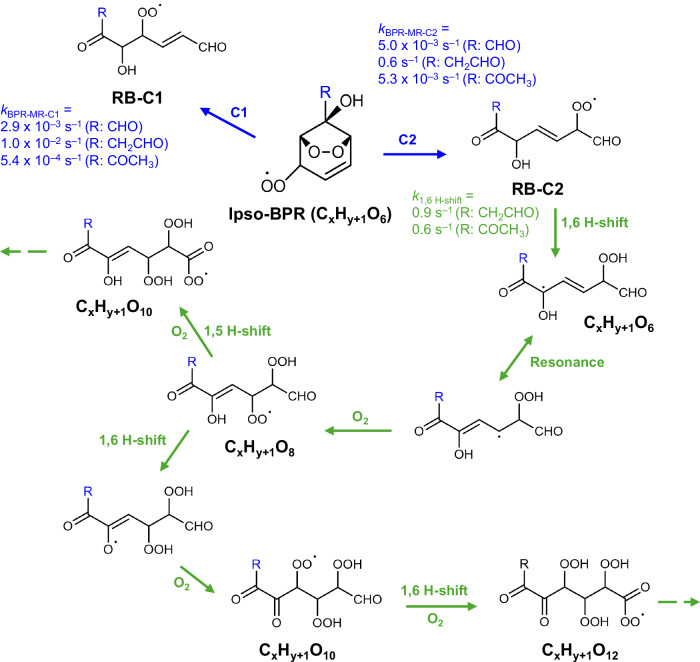
Mechanistic pathways leading to possible highly oxygenated organic molecule (HOM) formations from the ipso bicyclic peroxy radical (ipso-BPR) intermediate in OH-initiated oxidation of PhCHO, PhCH_2_CHO, and PhCOCH_3_. The blue channels are the two possible ring openings observed for ipso-BPR forming RB-C1 and RB-C2, and the green channels are the reaction sequences towards O_12_ HOMs once the BPR ring opens. The rate coefficients were obtained using the ROHF-ROCCSD(T)-F12a/VDZ-F12//ωB97X-D/aug-cc-pVTZ level of theory. The MC-TST rates are highlighted in blue, and the MESMER rates are in green. BPR-MR = bicyclic peroxy radical molecular rearrangement.

The autoxidation of these aromatic carbonyls proceeds via the initial attack of OH on PhCHO, PhCH_2_CHO, and PhCOCH_3_. The OH can either add to a carbon with a double bond or abstract an H atom, both forming C-centered radicals. The reaction schemes are provided in Supplementary Sections S5–6. The aldehydic-H abstraction is significantly more competitive than other H-abstractions or OH addition^3,4^. However, our computations for the PhCHO system did not find a competitive autoxidation path to HOM along this channel (see Supplementary Table 5). According to our results, it is the minor OH addition channel that initiates the autoxidation of aromatic carbonyls and the formation of HOM. We focused on the ipso-BPR intermediate (see Fig. 5 below and Supplementary Fig. 3) as it was recently shown to play a key role in the rapid formation of HOM from toluene autoxidation^16^. The molecular rearrangement of the ipso-BPR yields ring-broken structures RB-C1 and RB-C2 via C1 and C2 blue channels (see Fig. 5). These are more flexible than the double-ringed BPR and have significant excess energy of about 50 kcal/mol (Supplementary Table 4). This enables rapid subsequent autoxidation, along similar lines as toluene^16^, leading to HOMs with up to O_12_ oxygen content (shown via green channels). The BPR molecular rearrangement rates in Fig. 5 indicate that the overall reactivity of the studied aromatic carbonyls towards HOM formation is primarily dominated by the C2 channel. For PhCH_2_CHO, the BPR molecular rearrangement rate coefficient of 0.6 s^-1^ is similar to that of toluene reported by Iyer et al.^16^. It is significantly slower for PhCHO and PhCOCH_3_, comparable to benzene and ortho-xylene reported by the same authors. The first 1,6 H-shift in the ring-open structure of the C2 channel (RB-C2) leads to a resonance-stabilized alkyl radical, C_x_H_y__+1_O_6_, that can add O_2_ and undergo autoxidation to form O_12_-HOMs as shown. The rate coefficients of the 1,6 H-shift reaction immediately after the BPR ring opening are 0.9 and 0.6 s^–1^ respectively, for PhCH_2_CHO and PhCOCH_3_. These are faster than the predicted rate coefficient by Vereecken et al.^36^ for this reaction (0.18 s^–1^). This is likely due to the allylic nature of the abstracted H-atom in our case which tends to make these H-abstractions faster. The calculated rate coefficients of BPR molecular rearrangement and the subsequent 1,6 H-shift reveal that the overall reactivity of PhCOCH_3_ towards HOM formation is lower than that of PhCH_2_CHO. This is also reflected in their oxidation experiments in the presence of NO. Although other H-shift reactions are available for the ring-opened RO_2_, our previous work suggests that the 1,6 H-shift is likely the fastest^16^ and is therefore considered here.

### D_2_O experiments

The H/D exchange experiments provide additional insight into the product identities by giving an independent count of the exchangeable hydrogens in the form of OH/OOH groups. This is commonly used as an important tool to support the products’ molecular structures derived by the proposed reaction mechanism. The amounts of OH/OOH groups in the oxidation products observed in H/D exchange experiments are listed in Table 2 and the mass spectra are shown in Supplementary Fig. 8. A variable number of H/D conversion in some of the oxidation products implies that more than one isomer with varying OH/OOH groups is observed for those products, which further indicates that other alternative pathways than the generic one shown in Fig. 5 can also contribute to their formation process.

**Table 2:** The number of OH/OOH groups with exchangeable H atoms identified in the oxidation products of different aromatic carbonyl OH oxidation experiments in presence of D_2_O

PhCHO (C_7_H_6_O)		PhCH_2_CHO (C_8_H_8_O)		PhCOCH_3_ (C_8_H_8_O)	
Composition	H/D	Composition	H/D	Composition	H/D
C_7_H_6_O_5_ C_7_H_7_O_5_ C_7_H_8_O_5_	1 - 2	C_8_H_8_O_5_ C_8_H_9_O_5_ C_8_H_10_O_5_	- - -	C_8_H_8_O_5_ C_8_H_9_O_5_ C_8_H_10_O_5_	1 - 2–3
C_7_H_6_O_6_ C_7_H_7_O_6_ C_7_H_8_O_6_	2 - 2	C_8_H_8_O_6_ C_8_H_9_O_6_ C_8_H_10_O_6_	2 - 2	C_8_H_8_O_6_ C_8_H_9_O_6_ C_8_H_10_O_6_	2 - 2–3
C_7_H_6_O_7_ C_7_H_7_O_7_ C_7_H_8_O_7_	2 - 2–3	C_8_H_8_O_7_ C_8_H_9_O_7_ C_8_H_10_O_7_	2 2 3	C_8_H_8_O_7_ C_8_H_9_O_7_ C_8_H_10_O_7_	- - 2–3
C_7_H_6_O_8_ C_7_H_7_O_8_ C_7_H_8_O_8_	- 2 2–3	C_8_H_8_O_8_ C_8_H_9_O_8_ C_8_H_10_O_8_	3 - 3	C_8_H_8_O_8_ C_8_H_9_O_8_ C_8_H_10_O_8_	- - 4
C_7_H_6_O_9_ C_7_H_7_O_9_ C_7_H_8_O_9_	3 3 3	C_8_H_8_O_9_ C_8_H_9_O_9_ C_8_H_10_O_9_	2 2 3	C_8_H_8_O_9_ C_8_H_9_O_9_ C_8_H_10_O_9_	2–3 2–3 2–3
C_7_H_6_O_10_ C_7_H_7_O_10_ C_7_H_8_O_10_	- - 4	C_8_H_8_O_10_ C_8_H_9_O_10_ C_8_H_10_O_10_	- - 4	C_8_H_8_O_10_ C_8_H_9_O_10_ C_8_H_10_O_10_	- 3 4
		C_8_H_8_O_11_ C_8_H_9_O_11_ C_8_H_10_O_11_	2–3 - -	C_8_H_8_O_11_ C_8_H_9_O_11_ C_8_H_10_O_11_	3–4 3–4 3–4

In the reaction mechanism presented in Fig. 5, the bicyclic peroxy radical (BPR) intermediate and its ring-broken rearrangement products RB-C1 and RB-C2 which are alkyl peroxy radicals (C_x_H_y+1_O_6_) are having one OH group in their structure. The alkyl peroxy radical RB-C2 can undergo a bimolecular reaction with itself and produce closed-shell products alcohol (C_x_H_y+2_O_5_) and ketone (C_x_H_y_O_5_) with two and one OH groups respectively according to classical Russell mechanism (Supplementary Fig. 12) agreeing with the H/D number in Table 2. The radical C_x_H_y+1_O_6_ can also terminate the autoxidation propagation by its reaction with HO_2_ yielding C_x_H_y+2_O_6_ which is a closed-shell hydroperoxide with a total of two OH/OOH groups in its structure as seen in H/D exchange experiments (see Table 2). When the autoxidation intermediate C_x_H_y+1_O_8_ radical having two labile H groups (OH and OOH) reacts with HO_2_, it produces a closed-shell hydroperoxide C_x_H_y+2_O_8_ with one more OOH group in its structure. Accordingly, the mass shift of PhCH_2_CHO oxidation product C_8_H_10_O_8_ by three mass units in the spectrum associated with D_2_O experiment is in full agreement with the assigned structure of C_x_H_y+1_O_8_ in the autoxidation mechanism. This holds the same for C_x_H_y+1_O_10_ radical with three labile H groups (two OOH and one OH) that can produce C_x_H_y+2_O_10_ by the reaction with HO_2_, leading to an increased H/D number to four for all the aromatic carbonyls (Table 2). Furthermore, the C_x_H_y+1_O_8_ radical can also undergo a bimolecular RO_2_ + RO_2_ reaction (Russell mechanism, the same as C_x_H_y+1_O_6_ does) producing closed-shell products alcohol (C_x_H_y+2_O_7_) and ketone (C_x_H_y_O_7_) with three and two labile H-containing groups respectively. A mass shift of PhCOCH_3_ oxidation product C_8_H_10_O_8_ by four mass units in H/D exchange experiment can be explained by a branching of RB-C2 C_x_H_y+1_O_6_ intermediate towards alkoxy radical C_x_H_y+1_O_5_ and subsequent reaction propagation along its formation which is shown in Supplementary Fig. 14. Overall, the D_2_O experiment widely supports the autoxidation mechanism associated with the OH addition channel as the key pathway for the formation of the observed HOMs in the OH-initiated oxidation of aromatic carbonyls.

### Volatility estimation and atmospheric implications

While it is well acknowledged that peroxy radical (RO_2_) autoxidation can be perturbed by NO, leading to the formation of organonitrates (RONO_2_), the influence of NO on the growth of organic aerosol particles is yet to be explored in various atmospheric conditions involving different VOC types. Empirical studies suggest that organonitrates have higher volatility than the corresponding non-nitrogen-containing product^37^. A recent chamber study with monoterpenes by Yan et al.^38^ showed that NO_x_ suppresses particle growth by shifting HOM volatility towards higher end and by reducing mass concentrations in the extremely low-volatility category. However, previous reports show that highly functionalized organonitrates originated from different VOCs can contribute to particle growth and aerosol mass loading^39–41^.

**Fig. 6:**
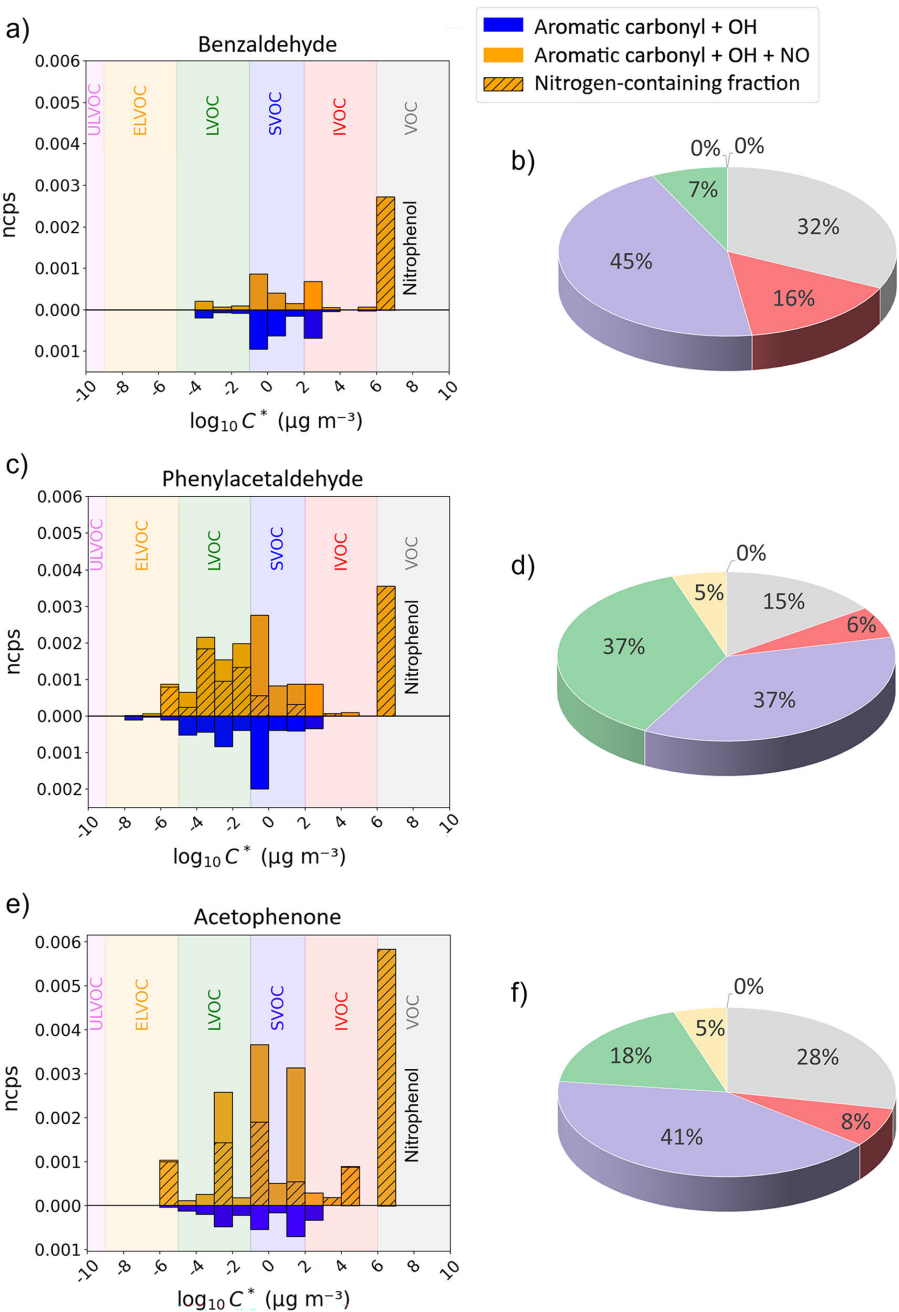
A comprehensive visualization of the volatility in terms of saturation concentration (log_10_C^*^) of the OH initiated oxidation products of aromatic carbonyls. Results from benzaldehyde are presented in panels **a**–**b**, phenylacetaldehyde in panels **c–d**, and acetophenone in panels **e–f**. The bar charts represent the abundance of the oxidation products in normalized counts per second (ncps) within different volatility bins with (orange) and without (blue) the presence of 100 ppb NO. The nitrogen-containing fractions are highlighted in diagonal hatching patterns. The pie charts depict the proportion of compounds (in ncps) within each volatility class based on the average log_10_C^*^ of individual compounds in the presence of NO (100 ppb). Different volatility classes are separated by individual color shades: volatile organic compounds (VOC) in grey, intermediate VOC (IVOC) in red, semi-VOC (SVOC) in violet, low VOC (LVOC) in green, extremely low VOC (ELVOC) in brown, and ultra-low VOC (ULVOC) in purple. Source data are provided as a Source Data file.

In this study, the detected products are categorized into different volatility classes based on their saturation mass concentration, C* (µg m^−3^), including ultra-low VOC (ULVOC, C* ≤ 10^–9^ µg m^−3^), extremely low VOC (ELVOC, 10^–9^ < C* ≤ 10^−5^ µg m^−3^), low VOC (LVOC, 10^–5^ < C* ≤ 10^−1^ µg m^−3^), semi VOC (SVOC, 10^–1^ < C* ≤ 10^2 ^µg m^−3^), intermediate VOC (IVOC, 10^2^ < C* ≤ 10^6 ^µg m^−3^)^42^. The relationship between saturation concentration and molar mass of the compounds estimated using six vapor pressure prediction models is shown in Supplementary Fig. 24. The bar charts (Fig. 6a, c, e) show that the concentration of oxygenated products (in normalized counts per second, ncps) across different volatility bins increased during the oxidation of phenylacetaldehyde (PhCH_2_CHO) and acetophenone (PhCOCH_3_) in presence of NO whereas in the case of benzaldehyde (PhCHO) oxidation, the influence of NO is reflected only on the formation of nitrophenol. In both PhCH_2_CHO and PhCOCH_3_ oxidation, NO enhanced the non-nitrogen-containing oxygenated products including HOMs as well as the organonitrates that contribute to the overall enhancement of the oxidation products in low-volatility to extremely low-volatility categories. Additionally, the pie charts (Fig. 6d, f) summarize that the OH-initiated oxidation of PhCH_2_CHO and PhCOCH_3_ in the presence of NO contributes to 37% and 18% of low-volatility product concentrations out of the total detected product concentrations, respectively, while both aromatic carbonyls contribute to 5% of extremely low-volatility product concentrations based on the current estimation. We consider that the sensitivity of the oxidation products is equal because NO_3_^–^ ionization is selective to HOMs and because of lack of methods that can account for differences in sensitivities across various oxygenated products. The exception is nitrophenol (C_6_H_4_OHNO_2_) that has an anomalously high sensitivity to NO_3_^–^-CIMS^43,44^ and thus it showed the highest intensity signal among all the oxidation products in the studied aromatic carbonyl oxidation. The distribution of the number of oxidation products in different volatility bins is shown in Supplementary Fig. 25.

To examine whether the experimental observations are relevant in the ambient atmosphere, we conducted kinetic simulations using our flow reactor conditions and those relevant to the ambient atmospheric conditions. In our flow reactor experiments, high aromatic carbonyl concentrations were used to allow for their reaction with OH radicals to be competitive with the reaction of the oxidant with TME. The simulation results show that the reactive radical species (e.g., OH, and primary RO_2_) produced in our experiments are close to the expected ambient concentrations (see Supplementary Section S15). Moreover, simulations with atmospherically relevant precursor concentrations show that HOMs form from the oxidation of phenylacetaldehyde and acetophenone, in agreement with our experimental observations.

This work also highlights the non-identical oxidation behavior across similar species (aromatic carbonyls). The tracking of complex oxidation chemistry leading to functionalized aerosol precursors from different VOCs in future studies requires information from several complementary investigations. Short reaction timescales provide key information on the efficiency of the pseudo-unimolecular autoxidation propagation by peroxy radicals, whereas longer reaction timescales and co-reactant addition, e.g., NO illustrate the ultimate direct aerosol precursor formation processes. Importantly, several complementary detection schemes are needed as none of the main CIMS-based methodologies are able to cover the whole condensable and key radical distributions by themselves. Furthermore, while computational insights into the oxidation mechanisms enable us to determine the key pathways to the formation of the important low-volatility products, experimental structural insights into these products aid in better volatility predictions.

The present work demonstrates how several representative aromatic carbonyls can rapidly form HOM upon atmospheric oxidation and contribute to atmospheric secondary aerosol. We experimentally show that the fastest achievable HOM formation is associated with phenylacetaldehyde forming O_12_ HOM in sub-second time scale, whereas benzaldehyde and acetophenone form HOM in around one- and three-second reaction time, respectively. The kinetic simulations under atmospherically relevant precursor concentrations show that the formation of HOM from phenylacetaldehyde and acetophenone is in line with our experimental results. Our experiments in the presence of high NO concentrations show that HOM yields enhance remarkably along with the formation of nitrophenol and HOM-ONs in OH-initiated oxidation of phenylacetaldehyde and acetophenone. However, at NO levels above 300 ppb—where the [NO]/[RO_2_] ratio for aromatic carbonyls is closer to polluted urban atmospheres—we observe a suppression of HOM formation and a corresponding decrease in yields with increasing NO. Interestingly, benzaldehyde neither showed any noticeable enhancement in HOM yields nor apparently formed organonitrates except for the formation of increased nitrophenol in the presence of variable NO concentrations. In the oxidation of phenylacetaldehyde and acetophenone, the alkoxy (RO) mediated autoxidation likely plays an important role in the enhancement of HOM in the presence of NO. The higher enhancement factor for acetophenone can be due to slower molecular rearrangement of the intermediate BPR, a crucial step for RO_2_ autoxidation, allowing NO to induce more RO-mediated chemistry compared to that for phenylacetaldehyde. Hydrogen to deuterium (H/D) exchange experiments indicate that the number of functional groups with exchangeable H atoms in the oxidation products agrees with the proposed reaction mechanisms. In addition, the theoretical volatility calculations show that a significant number of the oxidation products belong to the low- to extremely low-volatility class and their concentrations increase in the presence of NO. Therefore, this study highlights how NO modulates the importance of RO-mediated pathways that contribute to the HOM and condensable material budgets in urban environments.

## Methods

### Experimental setup

The OH oxidation of the aromatic carbonyl was conducted using a borosilicate flow reactor setup (Supplementary Fig. 1). A nitrate-based chemical ionization atmospheric pressure interface time-of-flight mass spectrometer (NO_3_^–^-CI-APi-TOF) was used to detect the oxidation products as NO_3_^−^ adducts. A sheath flow of 20 L min^−1^ to the CI inlet, and flow reactor bath gas maintaining a total sample flow of 8.1 L min^−1^ were supplied by a zero-air generator (AADCO-737-15). X-ray was used to produce NO_3_^–^ from gas-phase nitric acid (HNO_3_) carried by N_2_ that mixed with the sheath flow. The aromatic carbonyls were supplied to the reactor from individual gas cylinders while the oxidant hydroxyl radical (OH) was produced in situ by the ozonolysis of tetramethylethylene (TME). Ozone was produced by photolyzing the zero air using a mercury lamp (UVP, Analytik Jena), and the concentration was measured using an ozone analyzer (2B Technologies model 205). The initial concentrations of reactant precursors in the flow reactor were determined by controlling the individual gas flows using calibrated mass flow controllers (Alicat Scientific). The details including the chemicals and gas cylinders are given in Supplementary Sections S1–2. We conducted variable residence time experiments, including short (0.9–2.7±0.05 s) and long (14±0.05 s) residence times, by controlling the reaction time between the precursor and the oxidant. Short residence time experiments were achieved by providing the precursor VOC flow via a movable injector tube within the reactor and adjusting the distance of the injector tip with respect to the mass spectrometer orifice. On the other hand, the full reactor volume with no injector was used to achieve the long residence time. Table 3 lists the conditions for the conducted experiments including initial reactant concentrations at the beginning of the flow reactor. In short residence time experiments, the VOC concentrations of 1–3 ppmv with 99–105 ppbv of TME and 263–344 ppbv of ozone were utilized, and residence times of 1.1 ± 0.05 s, 0.9 ± 0.05 s, and 2.7 ± 0.05 s were maintained for the oxidation of benzaldehyde, phenylacetaldehyde, and acetophenone, respectively, chosen by the appearance of any HOM product signals. In all long residence time experiments, a constant VOC concentration (1 ppmv) was used while 52–109 ppbv of TME and 263–318 ppbv of ozone were adjusted.

**Table 3:** The experimental conditions for OH-initiated oxidation of the studied aromatic carbonyls: benzaldehyde, phenylacetaldehyde, and acetophenone

Experiment type VOC	[VOC]^Ф^ ppmv	[TME] ^Ф^ ppbv	[O_3_] ^Ф^ ppbv	[OH] ^≠^ pptv	[NO] ^Ф^ ppbv	D_2_O y/n^±^	RT s
**Short residence time**							
Benzaldehyde (C_7_H_6_O) Phenylacetaldehyde (C_8_H_8_O) Acetophenone (C_8_H_8_O)	1 1.5 3	98.7 104.8 104.8	344 263 318	5.2 3.9 4.8	N/A N/A N/A	n n n	1.1 0.9 2.7
**Long residence time**							
Benzaldehyde (C_7_H_6_O) Phenylacetaldehyde (C_8_H_8_O) Acetophenone (C_8_H_8_O)	1 1 1	52.4 104.8 52.4	263 263 318	3.9 3.9 4.8	N/A N/A N/A	n n n	14 14 14
**Experiments with NO**							
Benzaldehyde (C_7_H_6_O) Phenylacetaldehyde (C_8_H_8_O) Acetophenone (C_8_H_8_O)	1 1 1	52.4 104.8 52.4	263 263 263	3.9 3.9 3.9	50–1000 10–1000 10–1000	n n n	14 14 14
**Experiments with D**_**2**_**O**							
Benzaldehyde (C_7_H_6_O) Phenylacetaldehyde (C_8_H_8_O) Acetophenone (C_8_H_8_O)	1 1 1	52.4 104.8 52.4	263 263 318	3.9 3.9 4.8	N/A N/A N/A	y y y	14 14 14

*N/A* not applicable, *RT* Residence time.

^Ф^ Reactant concentrations at the beginning of the flow reactor.

^≠^ The initial OH concentrations (prior to adding precursor VOC, i.e., with only TME + O_3_ in the flow reactor) were calculated using bimolecular rate coefficients ${k}_{{O}_{3}-{TME}}=1.5\times {10}^{-15}$ cm^3^ molecule^–1^ s^–1^, ${k}_{{OH}-{TME}}=1.0\times {10}^{-10}$ cm^3^ molecule^–1^ s^–1^^62^, and the expression $\left[{OH}\right]=\left({k}_{{O}_{3}-{TME}}*\left[{O}_{3}\right]\right)/{k}_{{OH}-{TME}}$.

^±^ D_2_O added = y, not added = n.

To evaluate the influence of NO_x_ in the OH-initiated oxidation of the aromatic carbonyls, we conducted experiments with the presence of NO with concentrations ranging from 10 ppbv to 1000 ppbv. The NO range was chosen to maintain an experimental NO to VOC ratio up to 1:1 given that the usage of flow reactor platforms generally necessitates using high initial VOC concentration to produce appreciable initial RO_2_ radicals. In addition, to estimate the number of functional groups with labile H atoms (OH and/or OOH) in the oxidation products, we conducted hydrogen-to-deuterium (H/D) exchange experiments by introducing D_2_O into the flow reactor during aromatic carbonyl OH oxidation reactions. A near complete H/D conversion (98–99%) was achieved which is monitored by the signals of HNO_3_NO_3_^–^ and (HNO_3_)_2_NO_3_^–^ fully converting to DNO_3_NO_3_^–^ and (DNO_3_)_2_NO_3_^–^, respectively (see Supplementary Fig. 7). By tracking the mass shifts of signals of interest on the mass spectrum, the number of such functional groups in the corresponding oxidation products is determined. The time profiles of reactive species, VOC, OH, RO_2_, NO, and NO_2_ under different reaction conditions are shown in Supplementary Figs. 26–28.

### Quantum chemical calculations

The optimizations along with the vibrational frequency analyses for reactants, transition states, intermediates, and products were performed with density functional theory (DFT) using ωB97X-D/aug-cc-pVTZ^45–47^ electronic structure methods as implemented in Gaussian 16^48^. The DFT energies were further refined using MOLPRO^49^ version 2024.3 at ROHF-ROCCSD(T)-F12a/VDZ-F12 level of theory. This energy refinement provides accurate rate coefficients for the crucial reaction pathways in the autoxidation.

Conformer sampling was performed for the two crucial reaction steps in this autoxidation using the Merck Molecular Force Field (MMFF) method implemented in the Spartan′20 program^50,51^. Two crucial steps in the formation of HOM were studied: the molecular rearrangement of ipso-BPR and the subsequent 1,6-H-shift reaction of the ring-open peroxy radicals for PhCH_2_CHO and PhCOCH_3_. Details of the conformational analysis are provided in Supplementary Section S3.

The rate coefficients (*k*) reported in this work were estimated using the multi-conformer transition state theory (MC-TST)^51^ that includes Eckart quantum mechanical tunneling (к)^52^ as shown in Eq. (1) for unimolecular reactions.

1 $$k=\kappa \frac{{k}_{B}T}{h}\frac{{{\sum }_{i}^{{{\rm{all}}}\; {{\rm{TS}}}\; {{\rm{conf}}}.}\exp (-\frac{\Delta {E}_{i}}{{k}_{B}T})Q}_{{TS},i}}{{\sum }_{j}^{{{\rm{all}}}\; {{\rm{R}}}\; {{\rm{conf}}}.}\exp (-\frac{\Delta {E}_{j}}{{k}_{B}T}){Q}_{R,j}}\exp \left(-\frac{{E}_{{TS}}-{E}_{R}}{{k}_{B}T}\right)$$

In this equation, *k*_B_, and *h* are Boltzmann’s and Planck’s constants, respectively. *T* is the temperature at which the rate coefficients were calculated (298.15 K). *ΔE*_*i*_ is the zero-point-corrected energy of the *i*^*th*^ TS conformer relative to the lowest-energy transition state conformer, and *Q*_*TS,i*_ is the partition function of the *i*^*th*^ transition state conformer. Similarly, *ΔE*_*j*_ and *Q*_*R,j*_ are the corresponding values for reactant conformer *j*. *E*_*TS*_ − *E*_*R*_ is the zero-point-corrected barrier height corresponding to the lowest-energy TS and reactant conformers. The molecular rearrangement reactions have negligible tunneling, and consequently, the tunneling coefficients were assumed to be one in their computed rate coefficients.

### Master equation simulations

We employed the master equation solver for multi-energy well reactions (MESMER) program^53^ to account for the excess energy in the peroxy radicals after BPR molecular rearrangement when estimating the subsequent 1,6 H-shift rate coefficients for PhCH_2_CHO and PhCOCH_3_. The SimpleRRKM method with Eckart tunneling was used to treat unimolecular isomerization reactions. The intermediates were assigned as “modeled” in the simulations and Lennard–Jones parameters sigma (σ) and epsilon (ε) were given. The values of σ = 6.25 Å and ε = 518.9 K were used for PhCH_2_CHO, while σ = 6.72 Å and ε = 586.4 K were used for PhCOCH_3_^54^. For simulating the collisional energy transfer, we used exponential energy decay, *ΔE*_*down*_ = 225 cm^–1^, in our simulations. The temperature and pressure were given values of 298 K and 760 Torr, respectively, and numerical precision = qd was used. In addition, a grain size of 100 cm^–1^ and a value of 60 *k*_B_T for the energy spanned by the grains were used. The MESMER input file corresponding to one of the studied reactions is provided in the supplementary files as an example.

### Volatility calculations

Molecular volatility is affected by both molar weight and polarity, and it is important to consider molecular structure and functionality in volatility calculations. We calculated volatility of the oxidation products for those which we could predict the molecular structures (see Supplementary Figs. 12–23). Because different approaches treat the number and types of functional groups in a molecule differently, we applied several of them to obtain an average volatility estimate.

To determine the vapor pressure of these compounds, we employed four established methods that derive the species vapor pressure directly from the molecular structure: Nannoolal, SIMPOL, EVAPORATION, and Myrdal-Yalkowsky. These methods consider different molecular attributes, such as functional groups, thermodynamic principles, and empirical correlations, to estimate molecular vapor pressure. The Nannoolal method utilizes a group contribution approach, where the overall properties of a molecule are determined by summing the contributions of its individual structural groups^55^. The widely applied SIMPOL model uses a similar procedure, though with different structural motifs^37^. The EVAPORATION method also works similarly, yet it also considers certain functional group interactions, and adjusts for functionalized diacids with empirical modifications^56^. The Myrdal-Yalkowsky model assumes a log-linear relationship between vapor pressure and temperature and uses the enthalpy of fusion and melting point. It adjusts vapor pressure from a reference temperature based on thermodynamic principles, making it particularly useful for non-volatile organic compounds^57^. By averaging results from multiple approaches, we aimed to enhance the reliability of our volatility predictions and mitigate biases associated with any single approach.

In order to compare the volatility results obtained from the above approaches to values reported in several recent targeted SOA studies, the saturation concentration (proportional to volatility) of each oxidation product at 300 K is calculated within the two-dimensional volatility basis set (2D-VBS)^58^ framework. For this purpose, we applied a recent empirical parameterization for complex organic mixtures derived from 2D-VBS studies to estimate the saturation concentration, as shown in Eq. (2)^42^:

2 $${\log }_{10}{C}_{300\,K}^{*}=	 \left(25-{n}_{C}\right)\times 0.48-\left({n}_{O}-3{n}_{N}\right){b}_{O}\\ 	 -2\frac{{n}_{C}\left({n}_{O}-3{n}_{N}\right)}{{n}_{C}+{n}_{O}-3{n}_{N}}{b}_{{CO}}-{n}_{N}{b}_{N}$$

where *n*_*C*_ is the number of carbon atoms, *n*_*O*_ is the number of oxygen atoms, and *b*_*O*_ stands for the average effect of each added oxygen atom on log_10_C*. The terms *n*_*N*_ and *b*_*N*_ account for organonitrates, and they indicate number of nitrogen atoms and effect of each nitrate group (–ONO_2_) on log_10_C*, respectively. The coefficient *b*_*CO*_ introduces non-linearity in the effect of oxygen on volatility. In this approach, a *b*_*CO*_ of –0.3, a *b*_*N*_ of 2.5, and two different values for *b*_*O*_ (*b*_*O_monomer*_ = 1.4, and *b*_*O_accretion*_ = 1.17) were used to treat the monomeric and accretion products separately as presented by Stolzenburg et al.^59^. Here, a lower value of *b*_*O*_ for accretion products balances the contribution of oxygen in peroxide (ROOR) bonds, which has a lower effect in reducing the molecule’s volatility.

Secondly, for comparison purposes, the same parameterization given in Eq. (2) was also applied using alternative parameter values (*b*_*O*_ = 0.2 and *b*_*CO*_ = 0.9) that were adjusted based on a Boreal forest dataset^60^. All these approaches allow us to inspect the sensitivity of different parameterization on volatility and see the spread of the different volatility numbers for the same chemical composition.

### Kinetic simulations

To estimate the concentrations of reactive species in the flow reactor under different aromatic carbonyl oxidation conditions, we used a kinetic simulator, Kinetiscope version 1.1.1136.x64^61^. In the reaction scheme, TME ozonolysis reaction was used as an OH radical source. The reactions of NO with OH, O_3_, and RO_2_ were included for simulating experiments in the presence of NO. The bimolecular reactions of RO_2_ radicals were considered as the sink of the species. Simulations were also done to evaluate the feasibility of the formation of HOM from the oxidation of the studied aromatic carbonyls under ambient atmospheric conditions. In this case, atmospherically relevant concentrations of precursor VOCs, OH, NO, RO_2_, and HO_2_ were used. In both cases, a single-reactor model with constant volume, pressure, and temperature was employed in the simulation as applicable for gas-phase chemistry. The temperature was set to 298.15 K. In the simulation setting, a total number of particles 1 × 10^9^ and a random number seed 12947 were used, while the maximum simulation time was set to 14 s. Details of all the simulations are discussed in Supplementary Section S15.

## Supplementary information

Supplementary Information

Transparent Peer Review file

## Source data

Source Data

## Data Availability

Details about the experimental setup and mass spectrometry, mechanistic details of the early oxidation steps of the aromatic carbonyls, mass spectra of H/D exchange experiments, experimental reproducibility, more insights into HOMs, guess mechanisms for predicting molecular structures for volatility estimation, additional details about volatility classification and regression analysis, and details of kinetic simulations are provided in the Supplementary Information. The quantum chemistry output files (.log and.out), a MESMER input file (.xml), Excel files (.xlsx) with results from mass spectrometry and volatility models, a Word file (.docx) with predicted molecular structures, and Kinetiscope (.rxn) files – one with kinetic simulation input parameters for a laboratory experimental condition, another with the parameters for an ambient atmospheric condition – are available in the Zenodo repository at [10.5281/zenodo.18420667]. Source data are provided with this paper.
