# Supplementary material for: Nitric oxide can enhance secondary aerosol precursor formation from aromatic carbonyls
Source: Nat Commun. 2026 May 7;17:5454. doi: 10.1038/s41467-026-72628-w (PMC13282404; doi:10.1038/s41467-026-72628-w)
Supplement: Supplementary file 1 — Supplementary Information [file 41467_2026_72628_MOESM1_ESM.pdf]

Supplementary Information

Nitric oxide can enhance secondary aerosol precursor formation from aromatic carbonyls

Shawon Barua[1,*], Avinash Kumar[1], Prasenjit Seal[1], Mojtaba Bezaatpour[1], Sakshi Jha[1], Nanna Myllys[2], Siddharth Iyer[1], and Matti Rissanen[1,2,*]

[1]Aerosol Physics Laboratory, Physics Unit, Faculty of Engineering and Natural Sciences, Tampere University, 33720 Tampere, Finland

[2]Department of Chemistry, University of Helsinki, 00560 Helsinki, Finland

*Correspondence to: shawon.barua@tuni.fi, matti.rissanen@tuni.fi

Contents

S1 Flow reactor setup and mass spectrometry ……………………………………….…………….. 02

S2 Chemicals ………………………………………………………………………….............. 04

S3 Conformational sampling analysis ……………………………………………………... 04

S4 OH addition versus H-abstraction predicted by SAR ……………………………….. 05

S5 Formation of ipso bicyclic peroxy radical (i-BPR) ………………………………….. 07

S6 CO elimination versus O_2 addition ……………………………………………….. 09

S7 Formation of Nitrophenol from benzaldehyde oxidation in presence of NO_x 10

S8 Computational exploration via H-abstraction channel in benzaldehyde …………….. 10

S9 Sensitivity analysis with ΔE_{down} in MESMER …………………………………............ 11

S10 D_2O experiments ……………………………………………………………………….. 12

S11 Experimental reproducibility ……………………………………………………… 14

S12 HOM measurement in varying NO conditions ……………………………………… 16

S13 Mechanistic exploration for predicting molecular structures ………………………... 19

S14 Volatility classification and regression analysis ………...…………………………… 31

S15 Kinetic simulation …………………………………………………………………… 35

S15.1 Simulations without and with NO ……………………………………………….. 35

S15.2 Impact of HO_2 and PAN on OH and RO_2 …………………………………….. 40

S15.3 From laboratory conditions to ambient atmospheric conditions ……………… 42

Supplementary references ……………………………………………………………….. 45

S1. Flow reactor setup and mass spectrometry

A schematic of the borosilicate glass flow reactor setup is shown in Supplementary Fig. 1. A nitrate-based chemical ionization mass spectrometer (NO_3^--CIMS) with an Eisele type[1] atmospheric pressure inlet was coupled to the flow reactor. The mass spectrometer was manufactured by Tofwerk AG/Aerodyne Research Inc. and was equipped with a high-resolution time-of-flight mass analyzer (HToF, mass resolution: 3500 m/Δm). All the experiments were conducted at room temperature and 1 atm pressure of air with < 0.1% relative humidity. Because we did not introduce any photochemistry during the experiments, no lamp was used and thus there was no actinic flux measurement. Note that with the use of a borosilicate flow reactor, we did not observe any effect of laboratory lights. All the reactant gas supply lines were connected to the reactor via PTFE tubing and Swagelok fittings. The gas flows were controlled by Alicat mass flow controllers (MFC). The mass spectrometer inlet flow (8.1 lpm) and the volume of the reactor (1 m length and 4.7 cm inside diameter) define the residence time of the reaction mixture inside the reactor. Short residence time experiments were achieved by providing the precursor VOC flow via a movable injector tube within the reactor and adjusting the distance of the injector tip with respect to the mass spectrometer orifice. The deuterated water (D_2O) line and the NO line were used separately only during the hydrogen to deuterium (H/D) exchange experiment and the oxidation experiment in presence of NO, respectively.

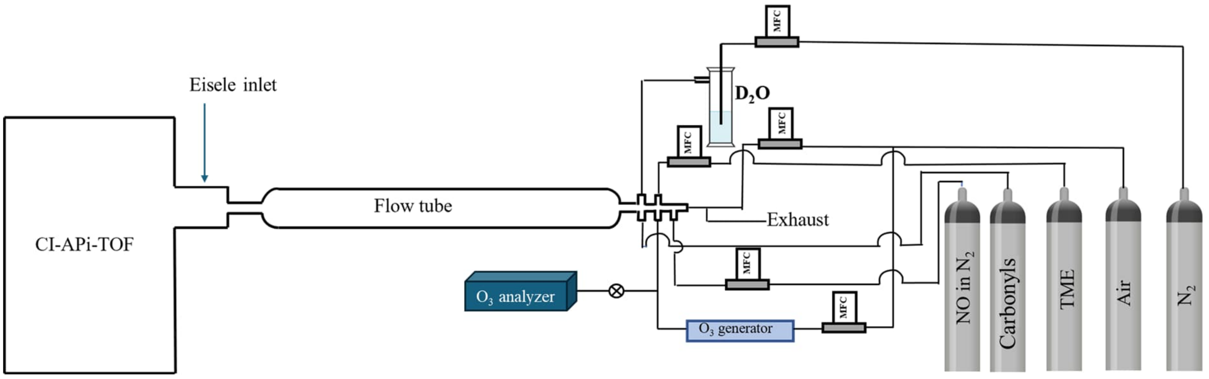

Supplementary Figure 1. A nitrate (NO_3^-) based chemical ionization mass spectrometer coupled to ambient pressure flow reactor. TME = tetramethylethylene. The oxidant OH radical was produced in situ by TME + O_3 reaction. MFC = mass flow controller. CI-APi-TOF = chemical ionization atmospheric pressure interface time-of-flight mass spectrometer. Modified from Barua et al.[2] under CC-BY 4.0 license.

At the beginning of the flow reactor, the in-situ production of oxidant OH radical was carried out by the ozonolysis reaction of tetramethylethylene (TME) as shown in Supplementary Fig. 2. The reaction produces acetone (CH_3COCH_3) and $C_3H_5O_3$ peroxy radical as the biproducts with OH radical. Although the biproducts adds some level of complexity to the reaction mixture in the flow reactor, they did not seem to complicate the detection of highly oxygenated organic molecules (HOMs) produced in the oxidation of aromatic carbonyls because of their distinct chemical compositions. The maximum residence time of the experiments was 14 s. The experiments were conducted in the absence of seed particles and we do not expect significant particle formation to occur.

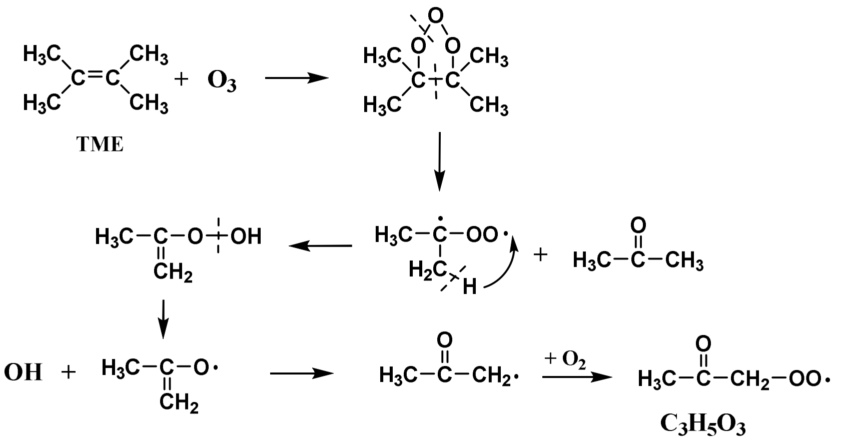

Supplementary Figure 2. Formation of OH radical along with acetone (CH_3COCH_3) and $C_3H_5O_3$ peroxy radical in the reaction between TME and ozone (O_3).

The initial concentrations of VOC and NO in the gas stream were calculated by using equation:

$$[X] = \frac{measured\ flow_x}{measured\ flow_t} \times [MR]$$

where species X can be either VOC or NO, $measured\ flow_x$ is the measured flow of the species X introduced in the gas stream, $measured\ flow_t$ is the measured total flow of the gas stream, and MR is the mixing ratio of the species X in the gas cylinder. On the other hand, the time profiles of VOC, NO, NO_2, etc. were estimated from kinetic simulation model discussed in Section S15 below. The average concentrations of OH, and primary RO_2 radicals under different reaction conditions are given in Supplementary Table 8.

The mass spectrometric data processing, including averaging, mass axis calibration, and peak integration, was done using the tofTools v6.03 package for MATLAB. The signal intensities of all the detected species were normalized using the following expression:

$$S = \frac{[X * NO_3^-]}{[NO_3^-] + [HNO_3NO_3^-] + [(HNO_3)_2NO_3^-]}$$

where $[X * NO_3^-]$ represents the intensity of an individual species X as NO_3^- adduct. In the denominator, $[NO_3^-]$, $[HNO_3NO_3^-]$, and $[(HNO_3)_2NO_3^-]$ represent the intensities of reagent ion monomer, dimer, and trimer, respectively.

We determined the calibration coefficient for our NO_3^--CIMS to be 2.0×10^9 molecule/cm^3/ncps by calibrating the instrument for sulfuric acid using the method shown by Kürten et al.[3] A detection limit (LOD) of 2.82×10^3 molecules/cm^3 for sulfuric acid was determined using the equation below.

$$LOD = \frac{3.3 \times \sigma}{S}$$

Here, S is the slope of the calibration curve and σ is the standard deviation of the responses of blank measurements. The HOMs were quantified based on the assumption that they are charged as efficiently as sulfuric acid. The concentrations of the oxidation products including HOMs were calculated by multiplying the individual normalized product signals with the calibration coefficient. The same calibration coefficient was commonly used for all oxidation products because of lack of methods that can account for differences in sensitivity across various oxygenated products. Then, the yields of the products were estimated by dividing their concentrations with the concentration of consumed precursor VOC (i.e., Y = ΔHOM/ΔVOC). Also, note that the flow reactor system exhibits unavoidable wall loss of reactive and condensable species, which contributes to the overall uncertainty in the experimental yields. The normal uncertainty range with the measurement technique has been assumed as asymmetric -50% to +100% (about a factor of two).

S2. Chemicals

High purity Nitrogen gas (5.0 grade) was obtained from Woikoski and Linde Oy. The NO gas cylinder (100 ppm in N_2) was obtained from Air Products. Deuterium oxide (99.9 atom % D) was obtained from Sigma Aldrich and was transferred to the flow reactor by bubbling nitrogen gas through a liquid D_2O reservoir. The following chemicals were used to make individual gas cylinders diluted in N_2: Tetramethylethylene (98%), benzaldehyde (purity ≥ 99.0%), phenylacetaldehyde (purity ≥ 98.0%), and acetophenone (99%) all from Sigma Aldrich. All the chemicals were used without further purification.

S3. Conformational sampling analysis

The conformers are generated by varying all torsional angles of each molecular species by 120°. Single-point quantum chemical calculations are performed on the conformers generated

using the Spartan ’20 program at B3LYP/6-31+G* level of theory using Gaussian 16 code. Only those within 5 kcal/mol in relative electronic energies with respect to the minimum energy geometry are considered for subsequent geometry optimizations. For the H-shift transition states (TS), we first constrain the H atom at an approximate distance from the relevant C and O atoms and then optimize the structure at B3LYP/6-31+G* level of theory. The optimized geometry is then used as an input for an unconstrained TS calculation. Once the TS geometry is found, an MMFF conformer sampling was carried out using Spartan′20 with the O---H and H---C bond lengths constrained. The partial bonds with torsions enabled are added to these two crucial bonds prior to the conformer sampling. The partial bonds results in improving the MMFF optimization, which in turn, provides geometries that are closer to local energy minima during the conformer sampling. After the initial sorting of the conformers, two sets of optimizations are performed, the first of which is done at B3LYP/6-31+G* (low-DFT) level of theory and structures within 2.0 kcal/mol of the minimum are then further re-optimized at ωB97X-D/aug-cc-pVTZ (high-DFT) to get the global minimum geometry. Finally, we perform energy refinement of the global minimum structure at ROHF-ROCCSD(T)-F12a/VDZ-F12 level of theory employing MOLPRO 2022.2. This energy refinement is done to get accurate and reliable energies for the estimation of the rate coefficients for some of the crucial autoxidation steps.

S4. OH addition versus H-abstraction predicted by SAR

The aromatic carbonyl OH oxidation can be initiated either by an H atom abstraction by OH from the substituent group of the phenyl ring or by the addition of the OH radical to the ring. To estimate the branching ratios between the two, we calculate the rate coefficients of the initial step of aromatic carbonyl OH oxidation reaction based on structure-activity relationship (SAR) data available in the literature.[4,5] The H-abstraction rate coefficients are calculated by using the formula, $k_{abs} = k_a \times F(X) \times F(Y)$ reported by Jenkin et al.[4] and Ziemann et al.[5] Here, k_a indicates the rate coefficient associated with the group (–C(O)H), $–CH_2–$, $–CH_3$) from which the H atom is being abstracted, where F(X) and F(Y) are the substituent group factors. In phenylacetaldehyde, for aldehydic H-abstraction, the substituent group factor F(X) corresponds to a $–CH_2–$ group. For a secondary H-abstraction from the $–CH_2–$, the substituent group factors F(X) and F(Y) correspond to –C(O)H and –Ph groups respectively.

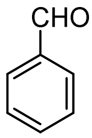

Benzaldehyde

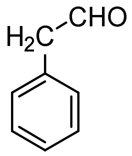

Phenylacetaldehyde

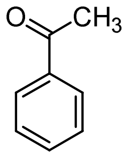

Acetophenone

The OH addition rate coefficients are calculated by using the formula, $\log k_{add} = -11.71 - 1.34 \times \Sigma\sigma^{+}$. In this approach, rate constants are calculated from a correlation between the rate constants and the sum of the electrophilic substituent constants $\Sigma\sigma^{+}$.[5] In all aromatic carbonyls, the values of substitution constant relative to *meta* position σ^{+}_{meta} are also used for *ipso* position as suggested by Ziemann et al.[5] for substituted aromatics. In benzaldehyde, we do not find a $\sigma^{+}_{ortho,para}$ for –CHO and we take the substituent constant from that of a closest match –C(O)OH for OH addition to *ortho* and *para* positions. In phenylacetaldehyde, we do not find either of $\sigma^{+}_{ortho,para}$ and σ^{+}_{meta} for $–CH_2CHO$ and we take the substituent constants from a closest functional group $–CH_2C(O)OCH_2CH_3$. Similarly, in acetophenone, due to the unavailability of $\sigma^{+}_{ortho,para}$ and σ^{+}_{meta} for $–C(O)CH_3$, we take the substituent constants from –C(O)OH group.[5] All the rate coefficients calculated using SAR are shown in Supplementary Tables 1–3.

Supplementary Table 1. Rate coefficients (in cm^3 $molecule^{-1}$ s^{-1}) of H-abstraction and OH addition reactions in benzaldehyde OH oxidation predicted by SAR.

H-abstraction; BR_{abs} = 85.52 %				
**Abstraction site**	**k_a†**	**F(X)‡**	**k_{abs}**	**BR (%)**
–CHO	20.8×10^{-12}	1 (X = –Ph)	2.08×10^{-11}	85.52
OH addition; BR_{add} = 14.48 %				
**Addition site**	**σ**	**σ^{+}**	**k_{add}**	**BR (%)**
Ipso	1	0.36	6.42×10^{-13}	2.64
Ortho	2	0.421	$2 \times (5.32 \times 10^{-13})$	4.37
Meta	2	0.36	$2 \times (6.42 \times 10^{-13})$	5.28
Para	1	0.421	5.32×10^{-13}	2.19

σ = symmetry factor. BR = branching ratio. $k_{overall} = 2.43 \times 10^{-11}$ cm^3 $molecule^{-1}$ s^{-1}.
† Values taken from Jenkin et al.[4] ‡ Values taken from Ziemann et al.[5]

Supplementary Table 2. Rate coefficients of H-abstraction and OH addition reactions in phenylacetaldehyde OH oxidation predicted by SAR.

H-abstraction; BR_{abs} = 62.48 %				
**Abstraction site**	k_a †	**F(X)‡, F(Y)‡**	k_{abs}	**BR (%)**
–CHO	20.8×10^{-12}	1.23 (X = $–CH_2–$)	2.56×10^{-11}	61.10
$–CH_2–$	0.77×10^{-12}	0.75 (X = –CHO)<br>1 (Y = –Ph)	5.78×10^{-13}	1.38
OH addition; BR_{add} = 37.52 %				
**Addition site**	**σ**	**σ⁺**	k_{add}	**BR (%)**
Ipso	1	-0.01	2.01×10^{-12}	4.80
Ortho	2	-0.164	$2 \times (3.23 \times 10^{-12})$	15.42
Meta	2	-0.01	$2 \times (2.01 \times 10^{-12})$	9.59
Para	1	-0.164	3.23×10^{-12}	7.71

σ = symmetry factor. BR = branching ratio. $k_{overall} = 4.19 \times 10^{-11}$ cm^3 $molecule^{-1}$ s^{-1}.
† Values taken from Jenkin et al.[4] ‡ Values taken from Ziemann et al.[5]

Supplementary Table 3. Rate coefficients of H-abstraction and OH addition reactions in acetophenone OH oxidation predicted by SAR.

H-abstraction; BR_{abs} = 2.53 %				
**Abstraction site**	k_a †	**F(X)‡**	k_{abs}	**BR (%)**
$–CH_3$	0.13×10^{-12}	0.75 (X = –C(O)–)	9.75×10^{-14}	2.53
OH addition; BR_{add} = 97.47 %				
**Addition site**	**σ**	**σ⁺**	k_{add}	**BR (%)**
Ipso	1	0.322	7.22×10^{-13}	18.71
Ortho	2	0.421	$2 \times (5.32 \times 10^{-13})$	27.57
Meta	2	0.322	$2 \times (7.22 \times 10^{-13})$	37.41
Para	1	0.421	5.32×10^{-13}	13.78

σ = symmetry factor. BR = branching ratio. $k_{overall} = 3.86 \times 10^{-12}$ cm^3 $molecule^{-1}$ s^{-1}.
† Values taken from Jenkin et al.[4] ‡ Values taken from Ziemann et al.[5]

S5. Formation of ipso bicyclic peroxy radical (i-BPR)

The first carbon centered radical ($C_xH_{y+1}O_2$ in Supplementary Fig. 3) formed via OH addition to the studied aromatic carbonyls can subsequently lead to non-fragmented oxidation products. The OH addition can take place at different positions of the phenyl ring, all of which can lead to the formation of bicyclic peroxy radicals (BPR). Recently, the formation of ipso-BPR

intermediate (initial OH addition to the carbon with a substituent group) and its subsequent ring-fragmenting rearrangement reaction has been found to be crucial for rapid formation of highly oxygenated organic molecules (HOMs) in aromatics and has been well established for toluene autoxidation.[6] Therefore, we focus on this intermediate to find reaction routes to explain the observed HOMs in aromatic carbonyl OH oxidation experiments. The computed rates and branching ratios for the two possible ipso-BPR rearrangement reactions (see Fig. 5 in the main manuscript) are given in Supplementary Table 4.

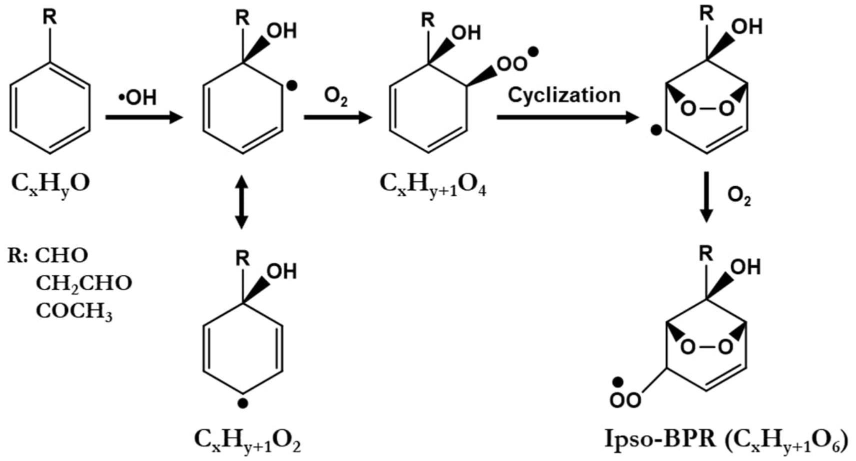

Supplementary Figure 3. An example of the formation of a bicyclic peroxy radical (BPR, $C_xH_{y+1}O_6$) in OH initiated oxidation of aromatic carbonyls showing the initial addition of OH to the carbon with the substituent group (R: CHO/CH_2CHO/$COCH_3$), i.e., ipso position.

Supplementary Table 4. Ipso-BPR opening energetics, rates, and branching ratios.

Ipso-BPR

ROHF-ROCCSD(T)-F12a/VDZ-F12//DFT-methods

Ipso-BPR (ΔE=0)	$\Delta E_{C1\ channel}$† (kcal/mol)		Rates (s^{-1})	Branching Ratio (%)	$\Delta E_{C2\ channel}$† (kcal/mol)		Rates (s^{-1})	Branching Ratio (%)
	TS	RB-C1‡			TS	RB-C2‡		
PhCHO	21.2	- 51.8	2.9 x 10^{-3}	**36.7**	20.5	- 48.2	5.0 x 10^{-3}	**63.3**
$PhCH_2CHO$	20.2	- 52.6	1.0 x 10^{-2}	**1.6**	17.9	- 48.9	0.6	**98.4**
$PhCOCH_3$	22.3	- 54.6	5.4 x 10^{-4}	**9.3**	20.6	- 48.2	5.3 x 10^{-3}	**90.7**

† Ipso-BPR rearrangement channels. ‡ Ring broken peroxy radical intermediates.

S6. CO elimination versus O_2 addition

The aldehydic H-abstractions in PhCHO and $PhCH_2CHO$ readily form benzoyl (PhCO) and phenylacetyl ($PhCH_2CO$) radicals, respectively. The methyl H-abstraction in $PhCOCH_3$ form an alkyl radical ($PhCOCH_2$) followed by the formation of alkoxy radical $PhCOCH_2O$ in the latter steps (see Supplementary Fig. 4). One possible way to form the observed fragmented products (with $C_{x-1,2}$) can be associated with the H-abstraction channel. In $PhCH_2CHO$ and $PhCOCH_3$, our calculation shows that a CO elimination ($k = 3.1 \times 10^7 s^{-1}$) and an alkoxy β-scission ($k = 1.2 \times 10^9 s^{-1}$) reaction with HCHO loss, respectively, are likely feasible. In contrast, in PhCHO, the CO elimination ($k = 1.4 \times 10^{-4} s^{-1}$) from benzoyl radical intermediate is very slow. The subsequent reactions can allow the elimination of a CO_2 molecule ($k = 9.6 \times 10^8 s^{-1}$) from the benzoyloxy radical (PhC(O)O) intermediate (see Supplementary Fig. 4).

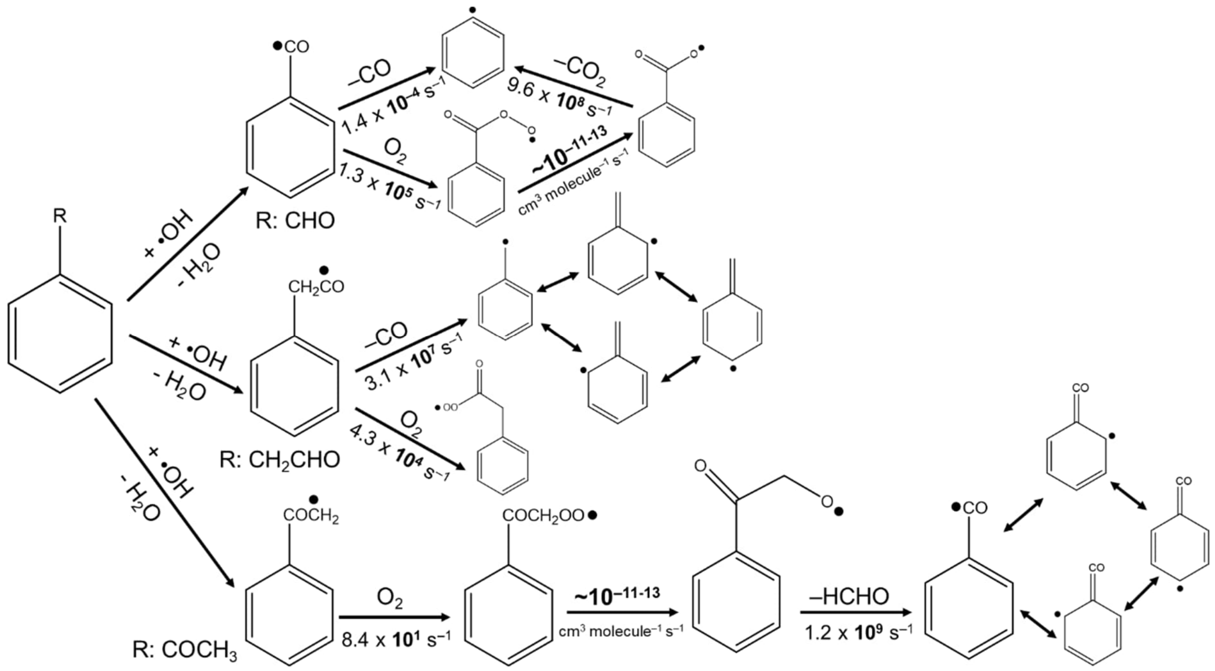

Supplementary Figure 4. The kinetics of the CO elimination and O_2 additions in aromatic carbonyl OH oxidation. The peroxy-alkoxy rates (R'O_2 to R'O) were assumed to lie between 10^{-11} and 10^{-13} $cm^3 molecule^{-1} s^{-1}$. The rate coefficients were obtained using the M08-HX/def2-TZVP level of theory. No conformer sampling was performed.

S7. Formation of Nitrophenol from benzaldehyde oxidation in presence of NO_x

The fast aldehydic H-abstraction by oxidant OH in benzaldehyde forms a carbon centered benzoyl radical (PhCO). The PhCO rapidly adds an O_2 molecule and forms a benzoyl peroxy radical ($PhC(O)O_2$) intermediate (see Supplementary Fig. 5). The reaction of $PhC(O)O_2$ with itself or with other reaction partners, e.g., RO_2, HO_2, and most importantly NO in this case, can form benzoyloxy radical of PhC(O)O which loses a CO_2 molecule yielding a benzyl radical. The benzyl radical upon O_2 addition followed by another bimolecular reaction, e.g., with NO, form the benzoxy radical (PhO). Finally, a reaction between the PhO radical and NO_2 forms nitrophenol that involves an intramolecular H-shift from C2 (where the NO_2 is attached) to the O atom at C1.

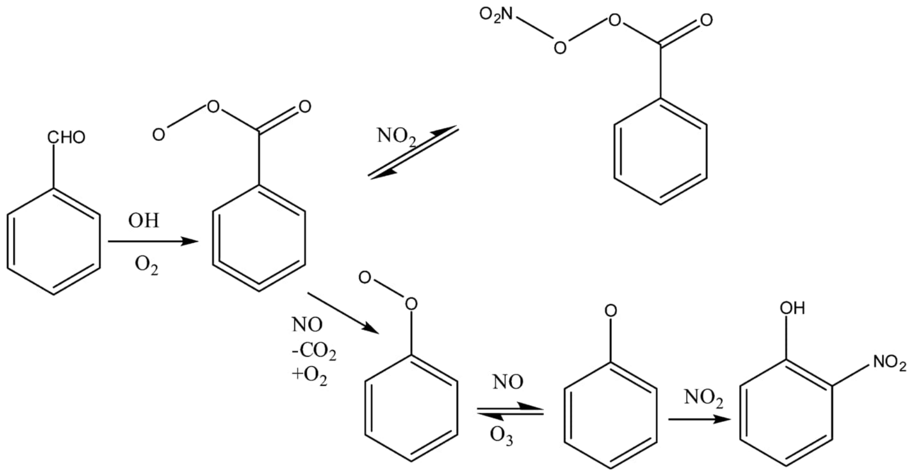

Supplementary Figure 5. Atmospheric degradation of benzaldehyde involving oxidation initiated by OH radical to form nitrophenol (adapted from Calvert et al.).[7]

S8. Computational exploration via H-abstraction channel in benzaldehyde

We looked at the possible isomerization reactions of $PhC(O)O_2$ to find a gateway to HOM along the H-abstraction channel. The peroxy O atom of the $PhC(O)O_2$ may attach to any of the double bonds in the aromatic moiety (i.e., ortho, meta, and para positions) and form carbon centered radicals with an additional five-to-seven-member ring. It may also undergo H-shift reactions with the secondary carbons at those positions in the aromatic moiety. However, our calculated reaction rate barriers (in kcal/mol at ωB97X-D/6-31+G* level of theory) for these reactions are very high (see Supplementary Table 5) and suggest that these isomerization reactions are unlikely to happen that can follow subsequent reactions to form HOM.

Supplementary Table 5. Reaction barriers of different isomerization reactions of $PhC(O)O_2$.

$PhC(O)O_2$ isomerization	Reaction barrier (kcal/mol)†
5-member Endo	25.3
6-member Endo	73.2
7-member Endo	76.4
1,5-Hshift	33.2
1,6-Hshift	113.4
1,7-Hshift	111.0

† Calculated at ωB97X-D/6-31+G* level of theory.

S9. Sensitivity analysis with ΔE_{down} in MESMER

In this work, the master equation solver for multi-energy well reactions (MESMER) program[8] was employed to account for the excess energy in peroxy radicals formed in i-BPR molecular rearrangement reaction when estimating the subsequent 1,6 H-shift rate coefficients for $PhCH_2CHO$ and $PhCOCH_3$. Details of simulation parameters are given in Methods section in the main manuscript. We used a ΔE_{down} value of 225 cm^{-1} for simulating collision energy transfer which is within the range recommended by MESMER for N_2 bath gas and has been used previously in similar simulations.[2,9] In addition, we ran multiple simulations for ΔE_{down} values ranging from 100 to 300 cm^{-1} and they did not change the 1,6 H-shift reaction rate coefficients of the ring broken RB-C2 alkyl peroxy intermediates in $PhCH_2CHO$ and $PhCOCH_3$ oxidation (Supplementary Fig. 6).

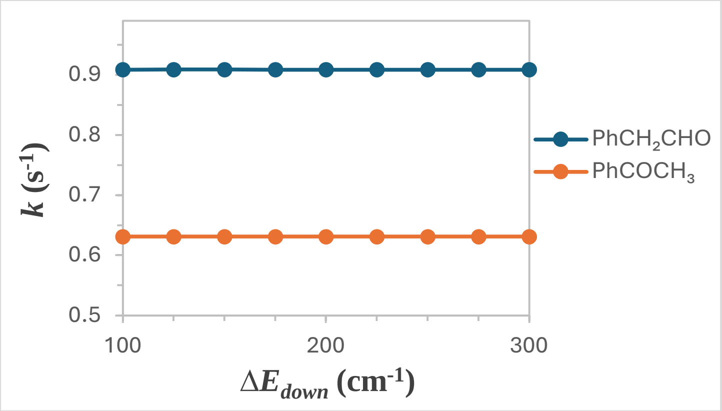

Supplementary Figure 6. Isomerization (1,6 H-shift) reaction rate coefficients of i-BPR-MR derived ring broken peroxy radicals (RB-C2) as a function of ΔE_{down} values in phenylacetaldehyde ($PhCH_2CHO$ in blue) and acetophenone ($PhCOCH_3$ in orange) oxidation.

S10. D_2O experiments

In the presence of excess D_2O, we could exchange the labile H atoms available with the oxidation products by D atoms. An example of such reactions is given below.

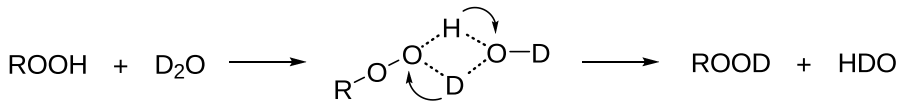

In the aromatic carbonyl OH oxidation experiments in presence of D_2O, a near complete H/D exchange was achieved which was monitored by the shift of $HNO_3NO_3^-$ and $(HNO_3)_2NO_3^-$ signals by one and two mass unit, respectively on the mass spectrum (see Supplementary Fig. 7). Based on the peak areas of $HNO_3NO_3^-$ and $DNO_3NO_3^-$ (in black) after the introduction of D_2O in the flow reactor, we calculated about 98–99 % H/D conversion in different aromatic carbonyl oxidation experiments. In the case of reagent dimer anion, about 97–98 % of $(HNO_3)_2NO_3^-$ converted to $(DNO_3)_2NO_3^-$ (in black) while 1–2 % remained as $HNO_3DNO_3NO_3^-$. A similar conversion level is always reflected in the detected oxygenated reaction products, and hence it is unlikely we are underestimating the number of labile H groups present.

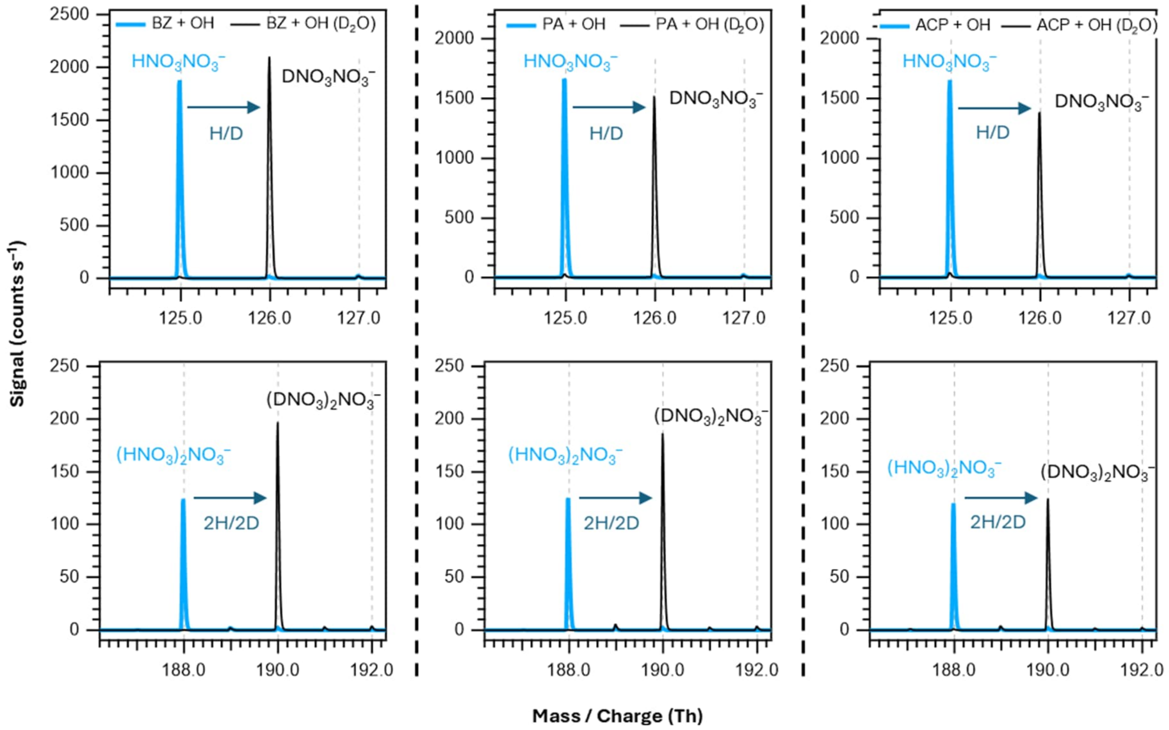

Supplementary Figure 7. H/D exchange in the reagent ion $HNO_3NO_3^-$ converting it to $DNO_3NO_3^-$ during different aromatic carbonyl OH oxidation reaction in presence of D_2O. BZ = benzaldehyde, PA= phenylacetaldehyde, and ACP = acetophenone.

A close comparison of mass spectra obtained from experiments without and with D_2O reveals that the addition of D_2O did not change the distribution of oxidation products except the shifts of product signals equivalent to the number of OH, OOH, and (or) C(O)OOH groups present in them (see Supplementary Fig. 8). Furthermore, NO_3^- ionization is almost completely insensitive to relative humidity changes.

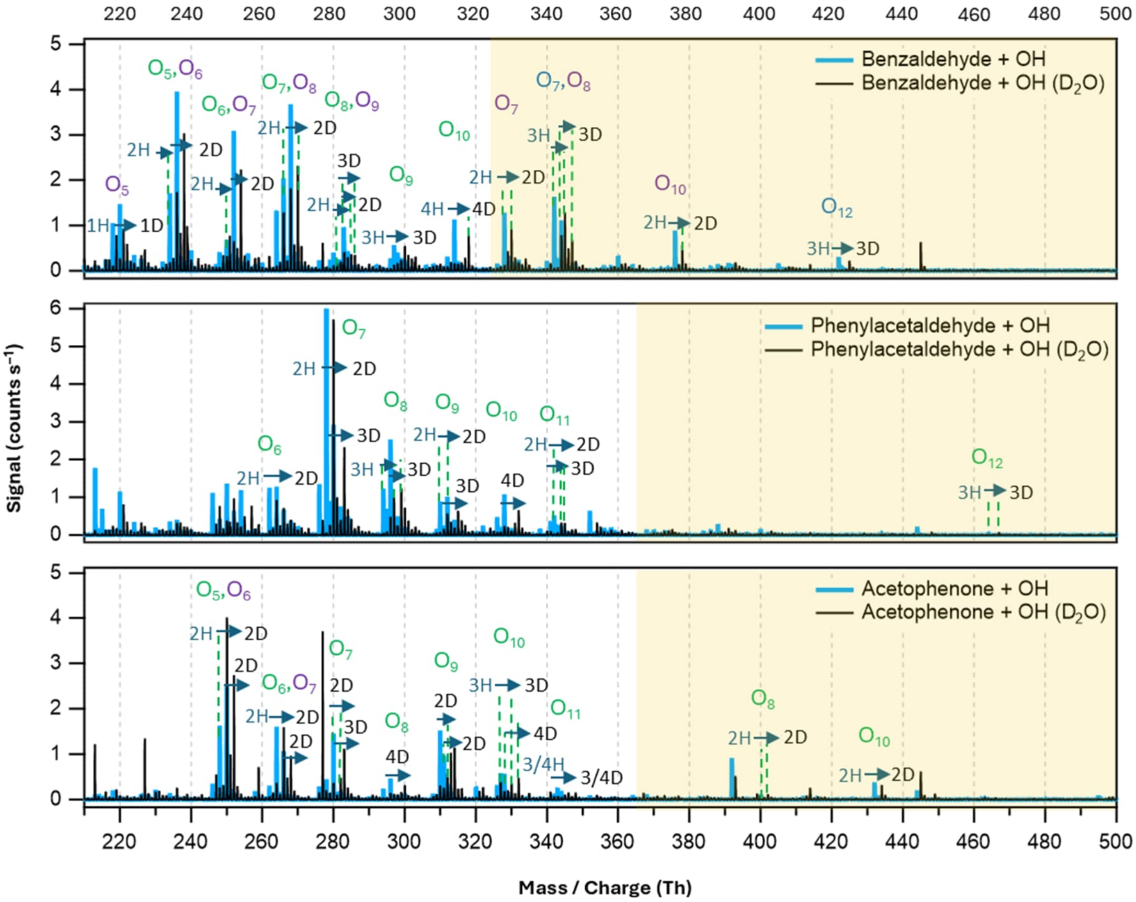

Supplementary Figure 8. Overlaid nitrate chemical ionization mass spectra of OH initiated oxidation of aromatic carbonyls without (in blue) and with the presence of D_2O (in black). The peak mass shifts during D_2O addition illustrate the number of exchangeable H-atoms present in the product molecular structures. The accretion products are highlighted in light brown background. The labels in green indicate non-fragmented (C_x and C_{2x}) products with different number of oxygen atoms while the labels in purple indicate fragmented (C_{x-1} and $C_{2(x-1)}$) products. The blue labels (O_7 and O_{12}) in the accretion product region of benzaldehyde spectrum (top panel) indicate products with 13 C atoms (i.e., C_7-RO_2 + C_6-RO_2).

S11. Experimental reproducibility

The results we obtained from different aromatic carbonyl oxidation experiments – formation of HOMs and distribution of oxidation products – are reproducible. Here, we show a few representative mass spectra of aromatic carbonyl oxidation experiments performed on different dates (and times) in Supplementary Fig. 9 below: phenylacetaldehyde (PA, panels a–b in black and grey), benzaldehyde (BZ, panels c–d in red and light red), and acetophenone (ACP, panels e–f in blue and light blue). However, every experiment is practically different and reproducing the experimental conditions of two separate experiments exactly the same, which require matching several gas flows from unique gas feeds through dedicated mass flow control systems, is almost impossible. In the case of PA, we can see the presence of some background signals at around mass/charge (m/z) 373 and 401 in Supplementary Fig. 9a (18.01.2024) which are negligible in Supplementary Fig. 9b performed on 01.02.2024 using same precursor conditions. In BZ (Supplementary Fig. 9c–d) and ACP (Supplementary Fig. 9e–f) experiment pairs, we had little differences in precursor BZ and ozone (O_3) concentrations, respectively, as their initial conditions while other reactants remained same. This was reflected in product signal intensities with somewhat higher signals in 1.3 ppm BZ condition compared to 1 ppm BZ as expected (Supplementary Fig. 9c–d). A similar observation is seen in the case of ACP under 263 ppb versus 312 ppb O_3 conditions (Supplementary Fig. 9e–f). Besides, we see different background signals m/z 226 and m/z 392 in panels e and f, respectively, of Supplementary Fig. 9. On the other hand, we could reproduce the same number and distribution of oxidation products in individual pairs of PA, BZ, and ACP experiments. Therefore, while we can reproduce the oxidation products of a precursor VOC in separate experiments, it is unusual to make an average of several spectra recorded at different times. Instead, it is common practice to present individual mass spectra normalized by the reagent ion signals without further manipulating the raw data for a fair comparison of results under different conditions (including different VOCs) as shown in Figs. 1–3 in the main manuscript.

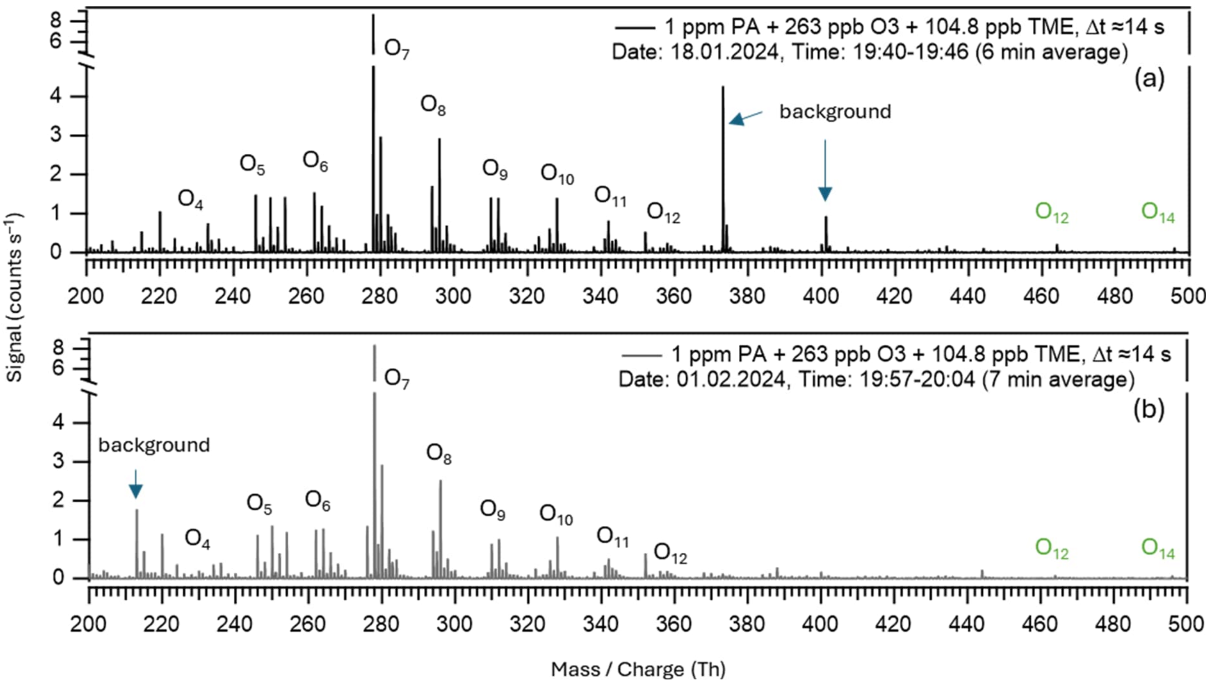
1 ppm PA + 263 ppb O3 + 104.8 ppb TME, Δt ≈14 s
Date: 18.01.2024, Time: 19:40-19:46 (6 min average)
(a)
background
O4
O5
O6
O7
O8
O9
O10
O11
O12
O14
1 ppm PA + 263 ppb O3 + 104.8 ppb TME, Δt ≈14 s
Date: 01.02.2024, Time: 19:57-20:04 (7 min average)
(b)
Signal (counts s-1)
Mass / Charge (Th)

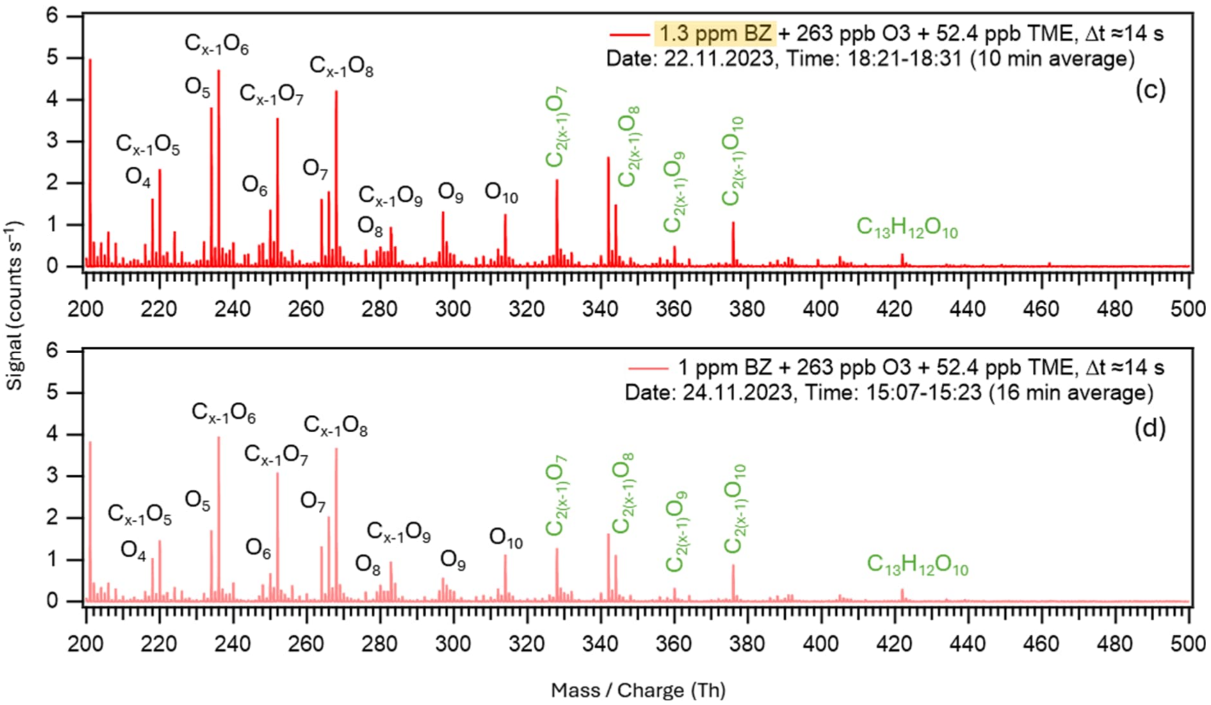
1.3 ppm BZ + 263 ppb O3 + 52.4 ppb TME, Δt ≈14 s
Date: 22.11.2023, Time: 18:21-18:31 (10 min average)
(c)
Cx-1O5
Cx-1O6
Cx-1O7
Cx-1O8
Cx-1O9
C2(x-1)O7
C2(x-1)O8
C2(x-1)O9
C2(x-1)O10
C13H12O10
1 ppm BZ + 263 ppb O3 + 52.4 ppb TME, Δt ≈14 s
Date: 24.11.2023, Time: 15:07-15:23 (16 min average)
(d)
Signal (counts s-1)
Mass / Charge (Th)

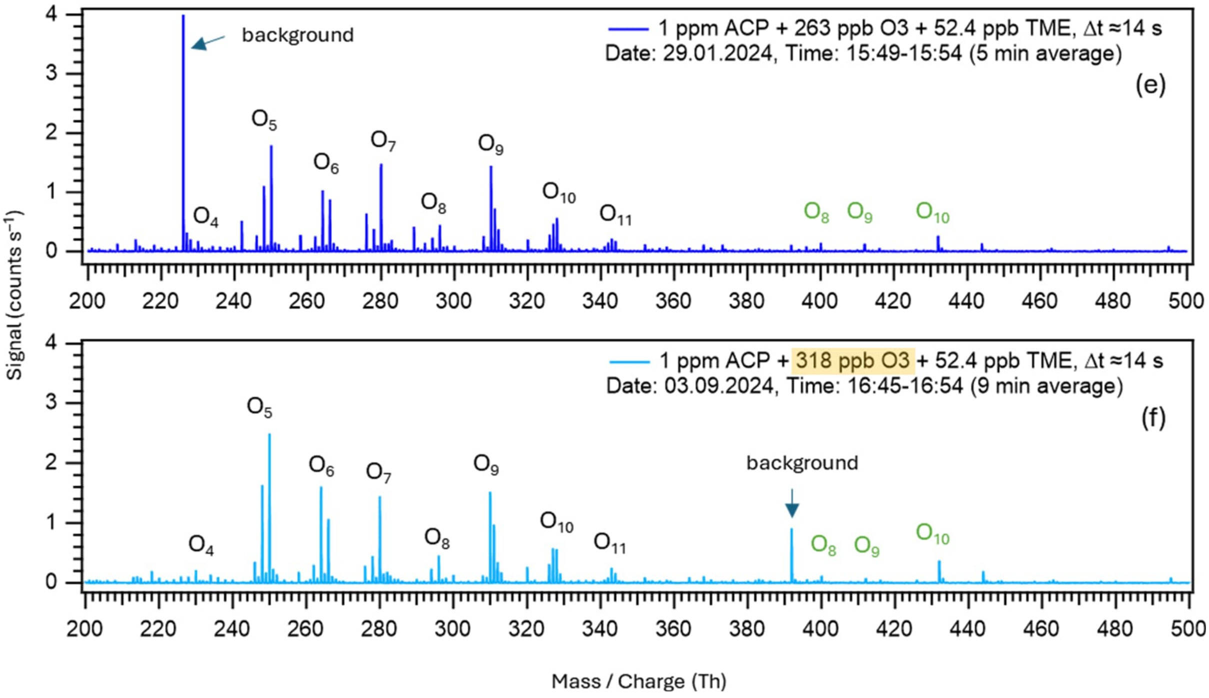

Supplementary Figure 9. Aromatic carbonyl oxidation spectra recorded in different times showing the reproducibility of the oxidation products: phenylacetaldehyde (PA, panels a–b in black and grey), benzaldehyde (BZ, panels c–d in red and light red), and acetophenone (ACP, panels e–f in blue and light blue). The labels on top of the oxidation product signals (O_{4-12}) indicate the extent of oxygenation in them. Monomeric product signals are labeled in black and that of accretion products are labeled in green. In the case of BZ (panels c–d), the labels starting with C_{x-1} and $C_{2(x-1)}$ indicate the products with loss of one carbon atom compared to precursor BZ and corresponding accretion products, respectively.

S12. HOM measurement in varying NO conditions

This section provides additional insights into the abundance of HOMs under varying NO concentrations. While we see a significant increase in HOM yields (Fig. 4a in the main manuscript) in PA and ACP oxidation under the studied range of initial NO (10–1000 ppb), Supplementary Fig. 10a below shows that the intensities of HOMs at around 500 ppb and above started to get lower than their intensities at zero NO condition. Although the abundance of the HOMs under higher NO conditions (≥ 500 ppb) was less, their higher yields were because of the lower consumptions of corresponding precursor VOCs. Indeed, in the presence of NO, the OH radical competes between its reactions with VOC and with NO leading to lower VOC consumption (see Supplementary Fig. 27 in Section S15). In Supplementary Fig. 10c, the enhancement factor (F) was calculated by taking the ratio of cumulative intensities of $C_{6–8}H_{8–}$

$_{10}O_z$ grouped by the number of oxygen atoms of the oxygenated products measured in the experiments with and without the presence of 100 ppb NO. Both PA and ACP showed a significant increase in the $C_{x(=8)}$ and $C_{x-1(=7)}$ HOM signal intensities with the latter one giving the higher intensities for almost all the oxygenated product groups O_6–O_{12}. Besides, we see a small suppression of total O_5 product intensities (F = 0.9, blue bar in Supplementary Fig. 10c) in ACP under this NO condition.

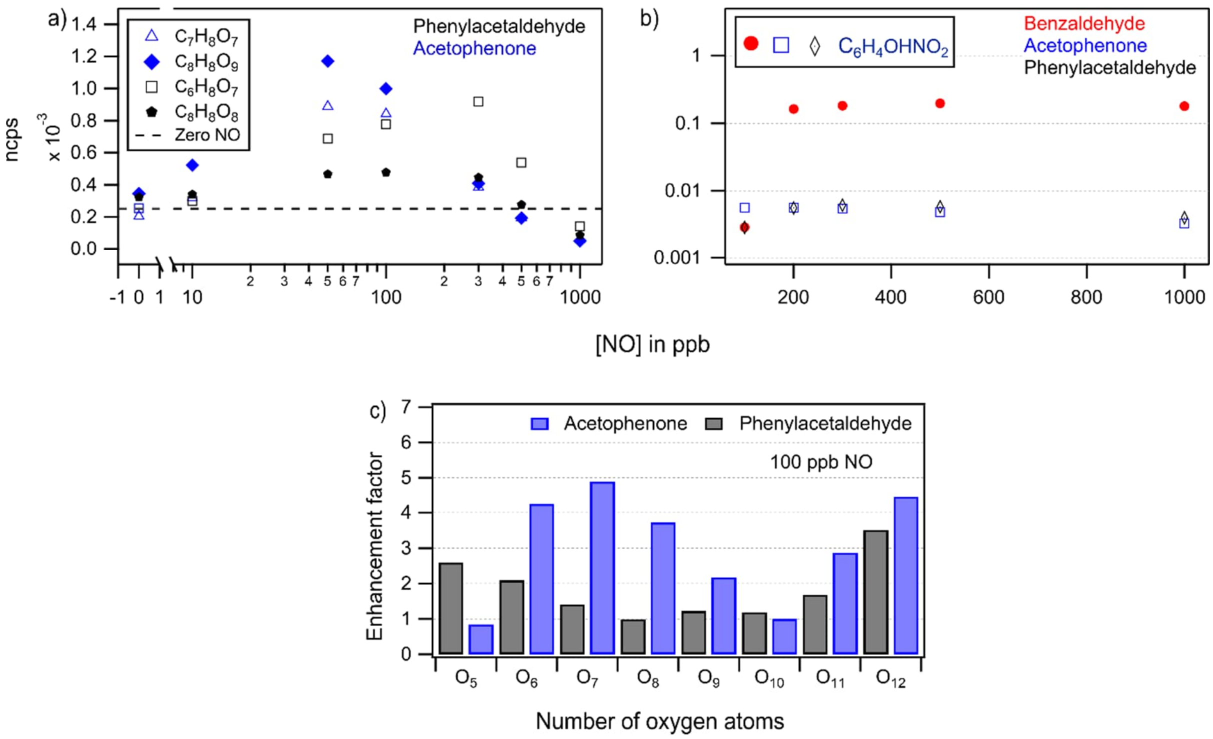

Supplementary Figure 10. Enhancement of HOM signals in presence of NO during the OH initiated oxidation of phenylacetaldehyde (black) and acetophenone (blue). (a) HOM signals detected by NO_3^--CIMS as a function of NO concentration shown in a logarithmic scale. Several representative HOMs including both non-fragmented C_8 and fragmented $C_{6–7}$ HOMs are shown in filled and unfilled markers, respectively. The black dashed line gives a rough estimation of the representative HOM peak intensities with zero NO. (b) The response of nitrophenol ($C_6H_4OHNO_2$) as a function of NO concentration in all the studied aromatic carbonyl systems including benzaldehyde in red. Note the logarithmic scale in y-axis. (c) Total signal enhancement of oxygenated products grouped by different oxygen number in presence of 100 ppb NO. Reaction time, Δt = 14 s.

We also inspected the signals of closed-shell $C_8H_{10}O_z$ products (i.e., ROOH and (or) ROH) in phenylacetaldehyde and acetophenone oxidation as a function of varying NO concentrations. In the presence of NO, we observed a decreasing trend in many of the $C_8H_{10}O_z$ signals (black

markers) while some of them showed an initial increase (green markers) in phenylacetaldehyde oxidation (Supplementary Fig. 11a). The opposite – an initial increase in many and a constant decrease in some products – was seen in acetophenone oxidation (green and black markers in Supplementary Fig. 11b). This reflected in the total $C_8H_{10}O_z$ signals (red markers in Supplementary Fig. 11a–b) as a constant decreasing trend in phenylacetaldehyde but as an initial increasing trend in acetophenone oxidation experiments with the increase of NO. Note that the signals that showed an initial increase in their intensities under varying NO, started to go below their initial signal levels (i.e., the signal level when [NO] = 0 ppb) only under very high NO concentrations (i.e., mostly from 500 ppb onwards).

In the presence of NO, a decrease in both HO_2 and RO_2 is expected limiting the reaction $RO_2 + HO_2 \rightarrow ROOH$. At the same time, NO can also limit the $RO_2 + RO_2$ reactions and the formation of closed-shell ROH via Russell mechanism ($RO_2 + R'O_2 \rightarrow R_{-H}{=}O + R'OH + O_2$). On the other hand, the reaction of RO_2 with NO produces alkoxy radical RO and the alkoxy-peroxy pathway can lead to oxidation chain propagation enhancing HOMs.[10] Furthermore, the alkoxy radical RO upon reaction with O_2 can form closed-shell carbonyl ($R_{-H}{=}O$) and recycle HO_2 ($RO + O_2 \rightarrow R_{-H}{=}O + HO_2$). Due to the general complexity of RO_2 bimolecular reactions and branching ratios, an accurate estimation of HO_2 cannot be achieved. Therefore, a direct correlation between HO_2 and $C_8H_{10}O_z$ and their underlying mechanisms cannot be established with certainty.

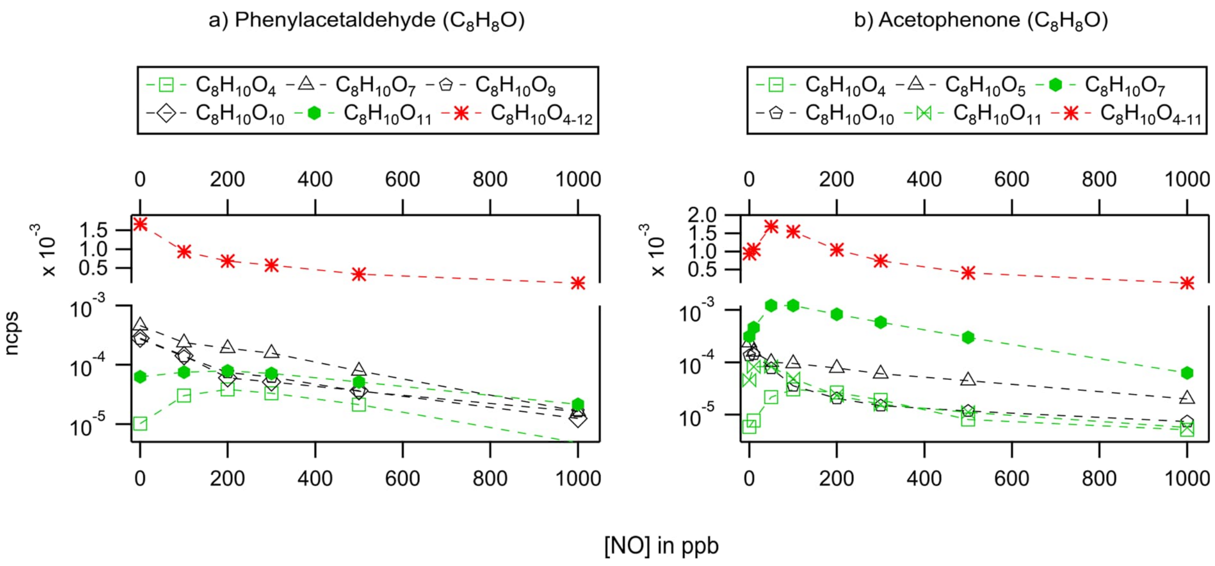

Supplementary Figure 11. Closed-shell $C_8H_{10}O_z$ product signals as a function of initial NO concentrations in phenylacetaldehyde (a) and acetophenone (b) oxidation experiments initiated by OH radicals. The sum of the products ($C_8H_{10}O_{4\text{–}12}$ in panel (a**)** and $C_8H_{10}O_{4\text{–}11}$ in panel (b))

are presented in red markers and excludes $C_8H_{10}O_6$ that overlaps with nitrophenol-$HNO_3NO_3^-$ adduct signal. The products beyond O_{12} in phenylacetaldehyde and beyond O_{11} in acetophenone were also excluded, which did not form in the absence of NO. Products shown with green markers showed an initial increase with NO and dropped below the initial signal level only under very high NO conditions (mostly 500 ppb onwards). The black markers represent the products that showed a decreasing trend with increasing NO.

S13. Mechanistic exploration for predicting molecular structures

In this section, we show the additional mechanisms extended from Fig. 5 of the main manuscript to predict the molecular structures of the oxidation products supported by the H/D exchange experiments in presence of D_2O. We use the predicted structures of the oxidation products to calculate their volatility discussed in the main manuscript and Section S14 below. Although other alternative pathways are likely available, here we explore those mainly arising from the i-BPR and the subsequent ring-opened peroxy radical. Besides, the alternative autoxidation pathways that can agree with the H/D exchange experiments are likely to produce similar product structures with the same number of OH/OOH groups. Therefore, they will not significantly alter the current volatility estimation by group additivity and volatility basis set based methods even though the spatial arrangements of the actual isomeric structures could be different.

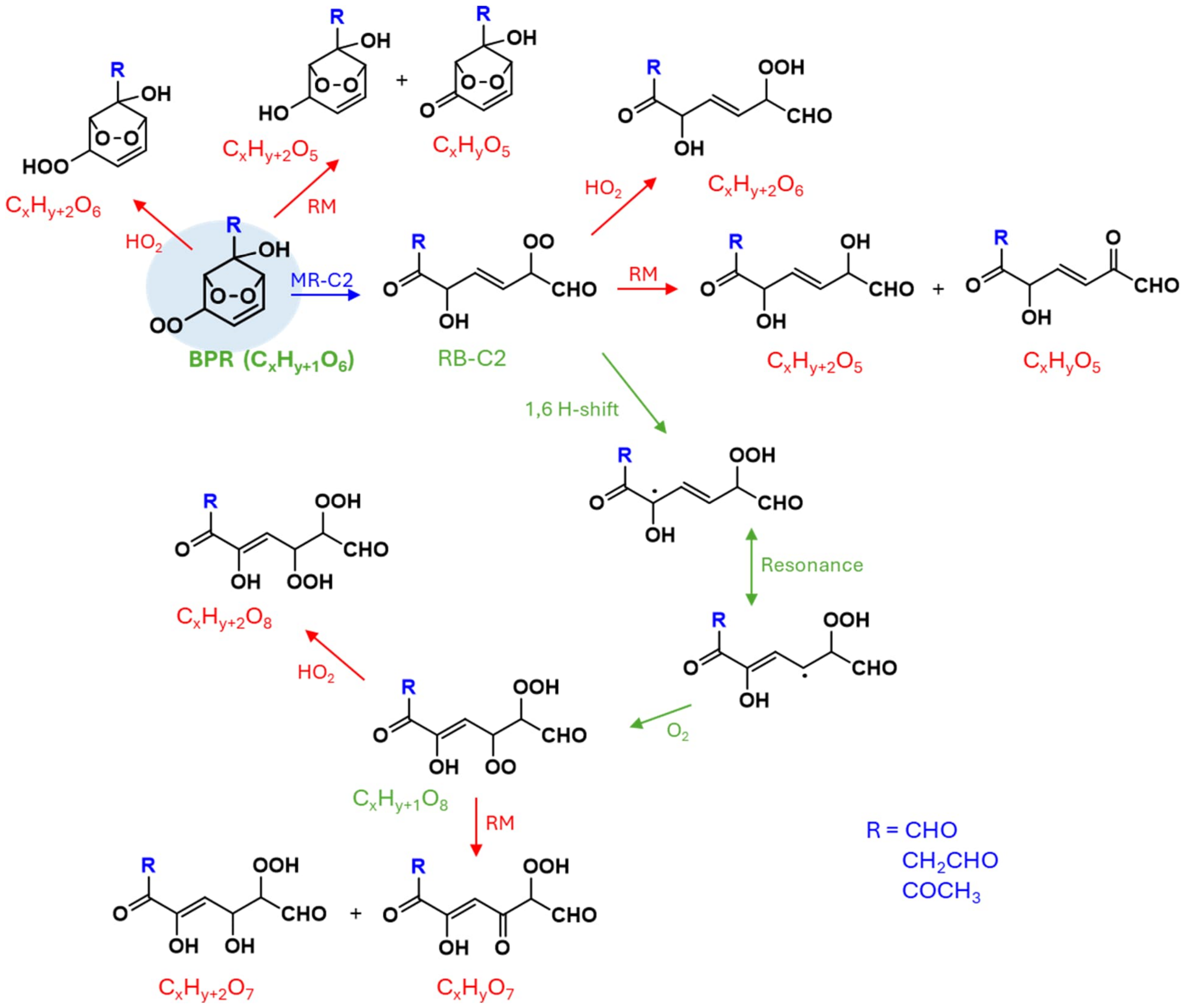

Supplementary Figure 12. Potential formation pathways of $C_xH_yO_{5,7}$, $C_xH_{y+2}O_{5,7}$, $C_xH_{y+2}O_{6,8}$, and $C_xH_{y+1}O_8$ from bicyclic peroxy radical (BPR, $C_xH_{y+1}O_6$). The green arrows indicate chain propagation reactions while the red arrows indicate chain termination reactions. MR-C2 = molecular rearrangement via C2 channel. RM = Russell mechanism ($RO_2 + R'O_2 \rightarrow ROH + R'_{-H}C{=}O + O_2$).

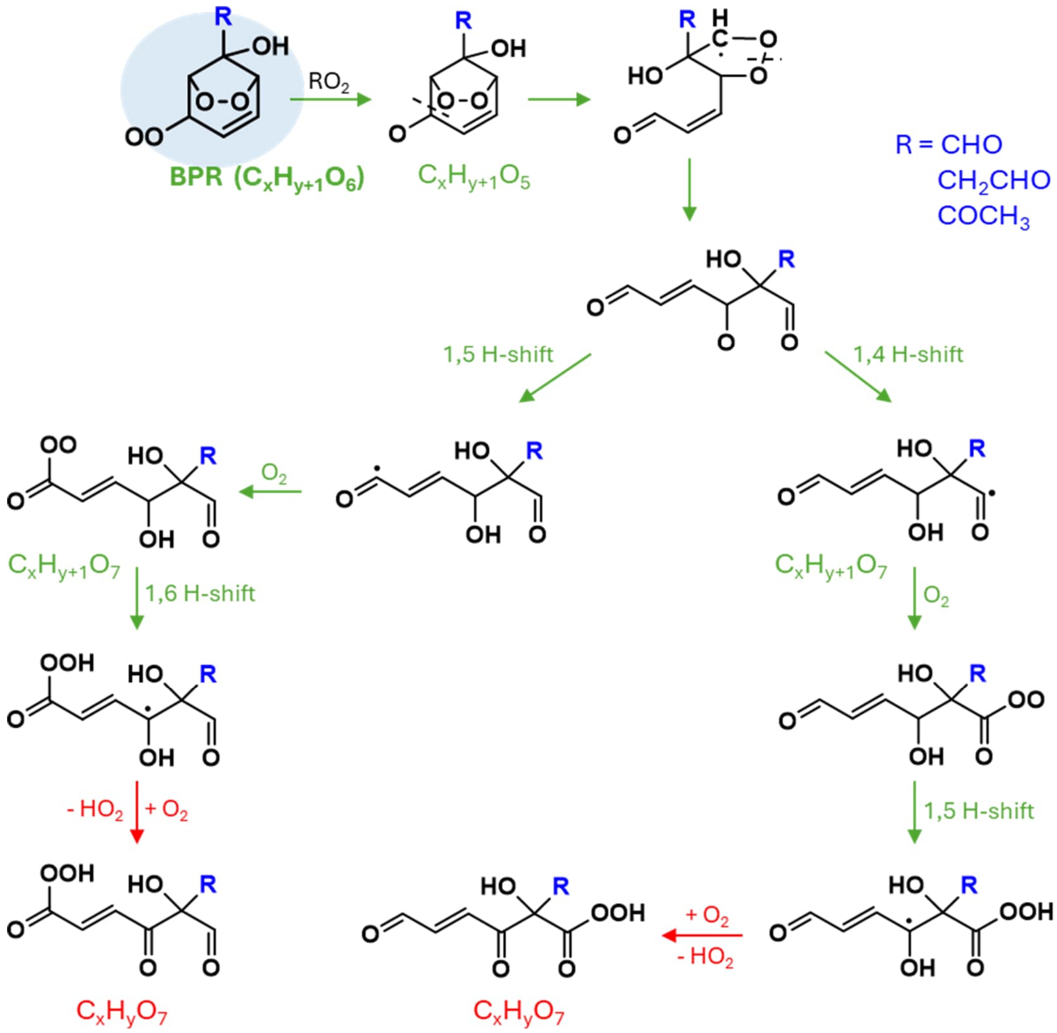

Supplementary Figure 13. Potential formation pathways of $C_xH_{y+1}O_7$, and $C_xH_yO_7$ from bicyclic peroxy radical (BPR, $C_xH_{y+1}O_6$) via bicyclic alkoxy radical $C_xH_{y+1}O_5$. The green arrows indicate chain propagation reactions while the red arrows indicate chain termination reactions.

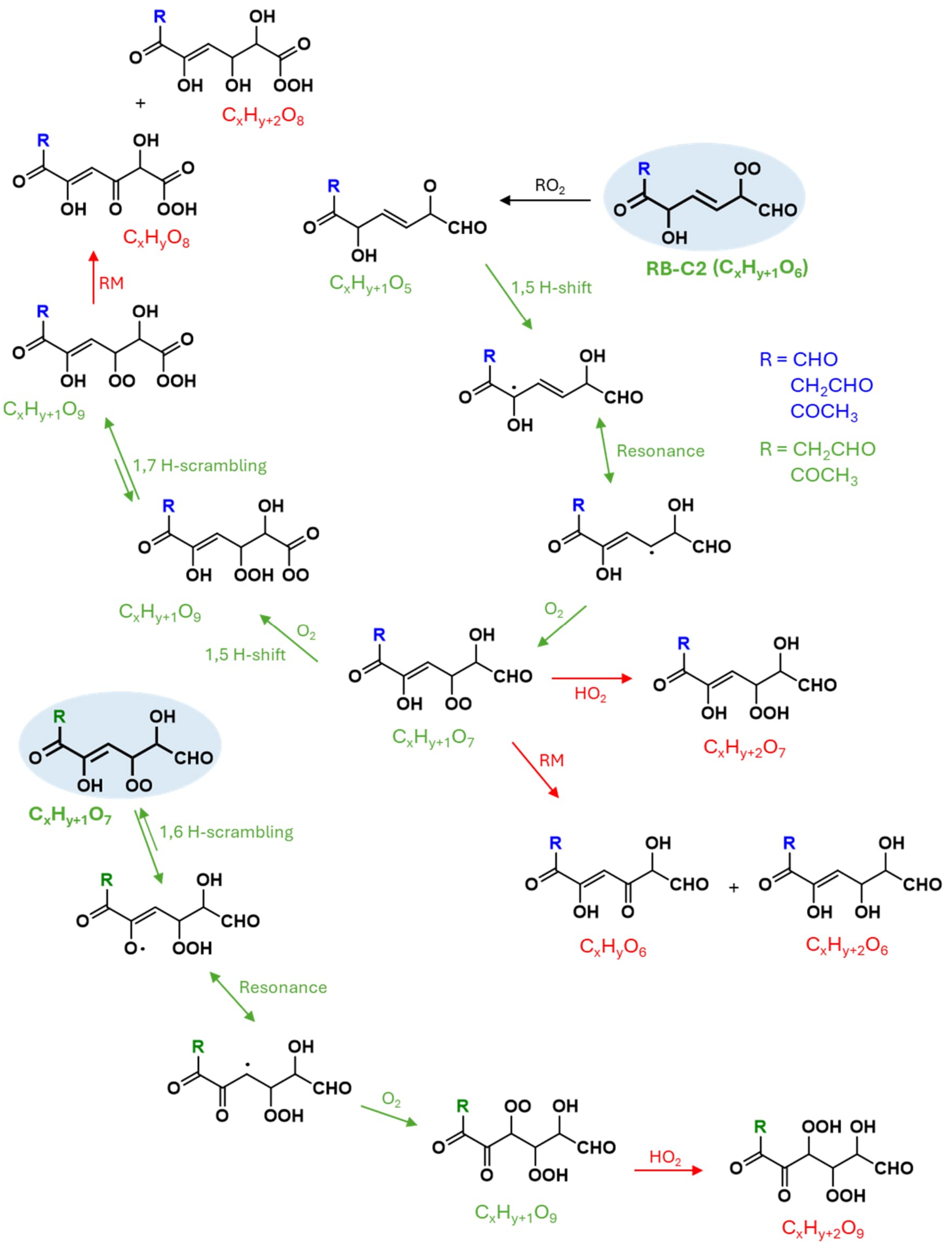

Supplementary Figure 14. Potential formation pathways of $C_xH_{y+1}O_{7,9}$, $C_xH_yO_{6,8}$, and $C_xH_{y+2}O_{6–9}$ from RB-C2 ($C_xH_{y+1}O_6$). The green arrows indicate chain propagation reactions while the red arrows indicate chain termination reactions. RM = Russell mechanism (RO_2 + $R'O_2 \rightarrow ROH + R'_{-H}C{=}O + O_2$).

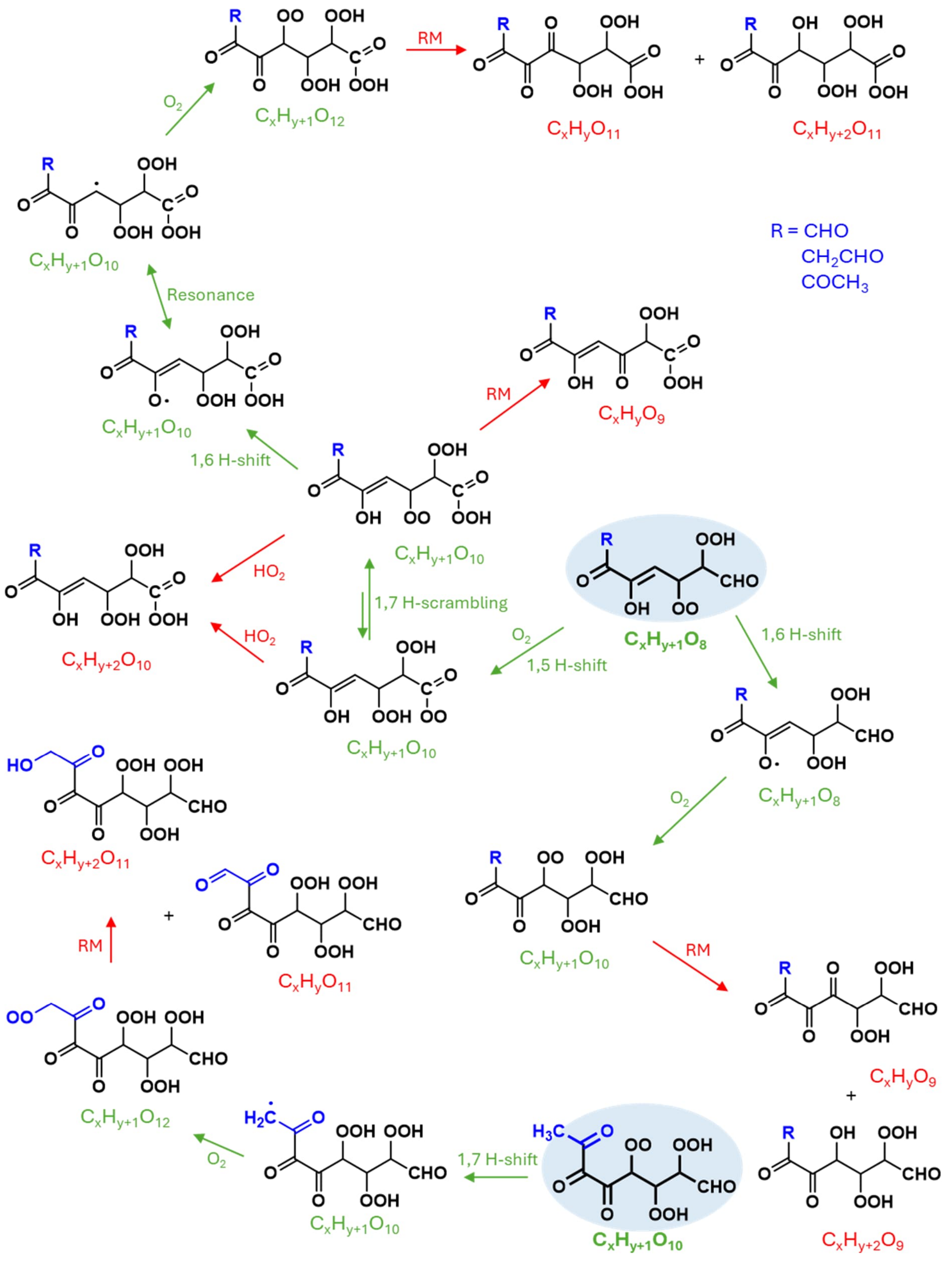

Supplementary Figure 15. Potential formation pathways of $C_xH_yO_{9,11}$, $C_xH_{y+2}O_{9,11}$, $C_xH_{y+1}O_{10,12}$, and $C_xH_{y+2}O_{10}$ from O_8 peroxy radical ($C_xH_{y+1}O_8$). The green arrows indicate chain propagation reactions while the red arrows indicate chain termination reactions. RM = Russell mechanism (RO_2 + $R'O_2$ → ROH + $R'_{-H}C{=}O$ + O_2). Note that the $C_xH_{y+1}O_{12}$ peroxy

radicals reacts also with HO_2 to form closed shell $C_xH_{y+2}O_{12}$ ($RO_2 + HO_2 \rightarrow ROOH + O_2$) with one more OOH functional group.

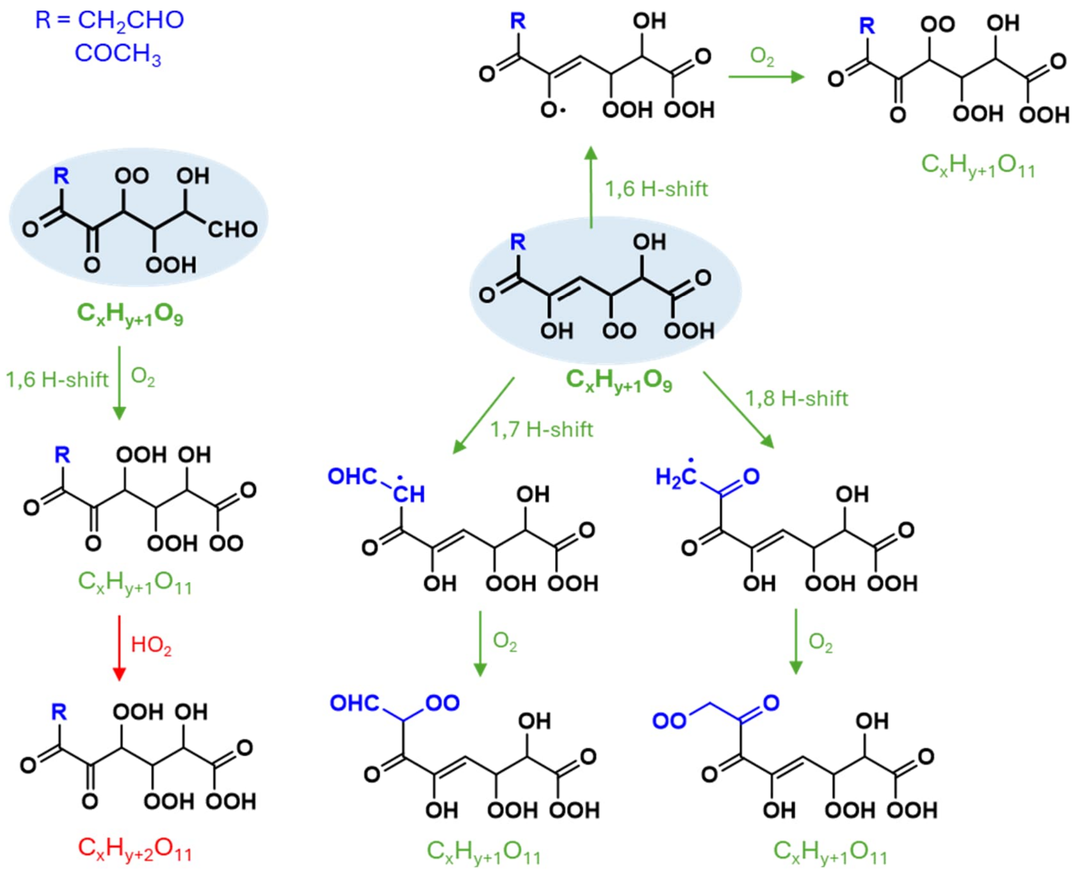

Supplementary Figure 16. Potential formation pathways of $C_xH_{y+1}O_{11}$, and $C_xH_{y+2}O_{11}$ from O_9 peroxy radical ($C_xH_{y+1}O_9$, see Supplementary Fig. 14) in phenylacetaldehyde and acetophenone oxidation. The green arrows indicate chain propagation reactions while the red arrows indicate chain termination reactions.

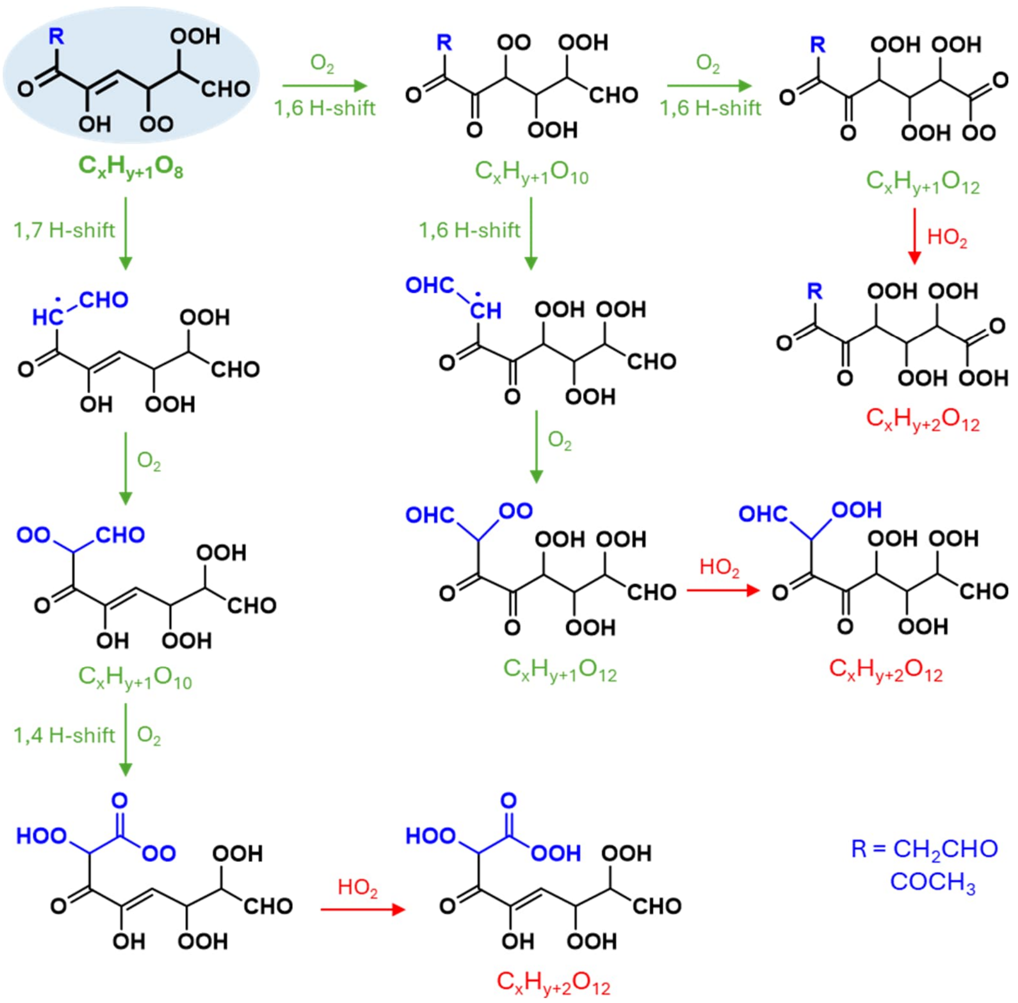

Supplementary Figure 17. Additional potential pathways of formation of $C_xH_{y+1}O_{12}$, and $C_xH_{y+2}O_{12}$ from O_8 peroxy radical ($C_xH_{y+1}O_8$, see Supplementary Fig. 15) in phenylacetaldehyde and acetophenone oxidation. The green arrows indicate chain propagation reactions while the red arrows indicate chain termination reactions.

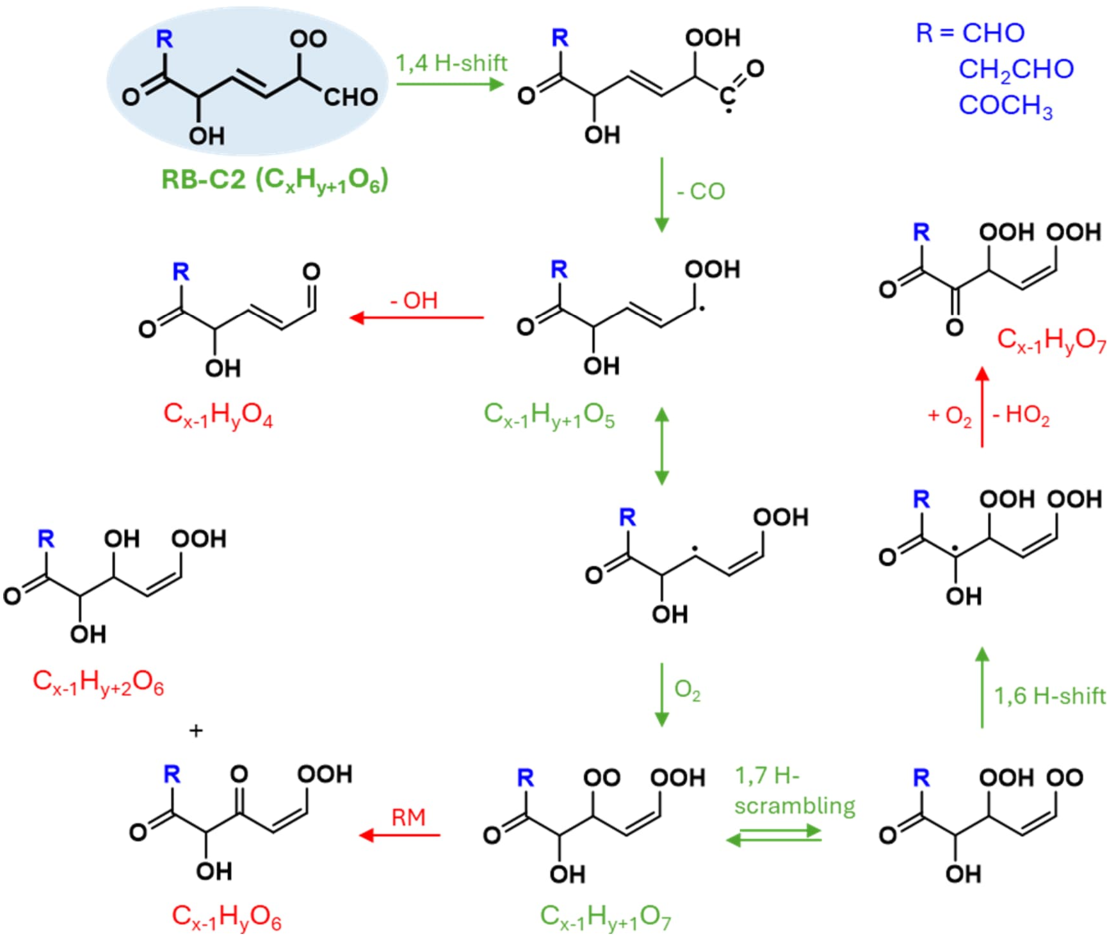

Supplementary Figure 18. Potential formation pathways of $C_{x-1}H_yO_{6,7}$ from peroxy radical RB-C2 ($C_xH_{y+1}O_6$). The green arrows indicate chain propagation reactions while the red arrows indicate chain termination reactions. RM = Russel mechanism (RO_2 + $R'O_2$ → ROH + $R'_{-H}C{=}O$ + O_2).

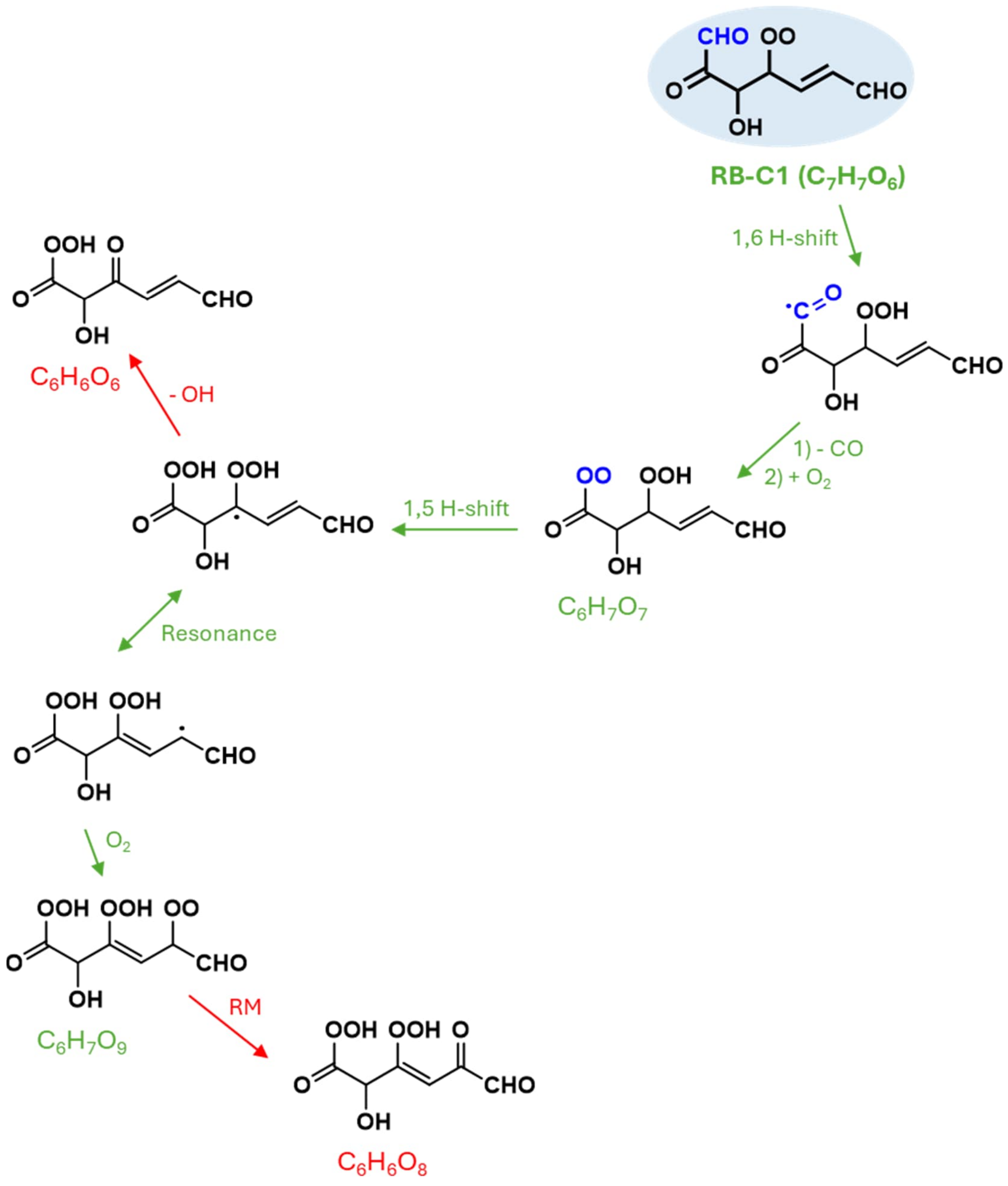

Supplementary Figure 19. Potential formation pathways of $C_6H_6O_{6,8}$ from peroxy radical RB-C1 ($C_7H_7O_6$) in benzaldehyde oxidation. The green arrows indicate chain propagation reactions while the red arrows indicate chain termination reactions. RM = Russel mechanism (RO_2 + $R'O_2 \rightarrow ROH + R'_{-H}C{=}O + O_2$).

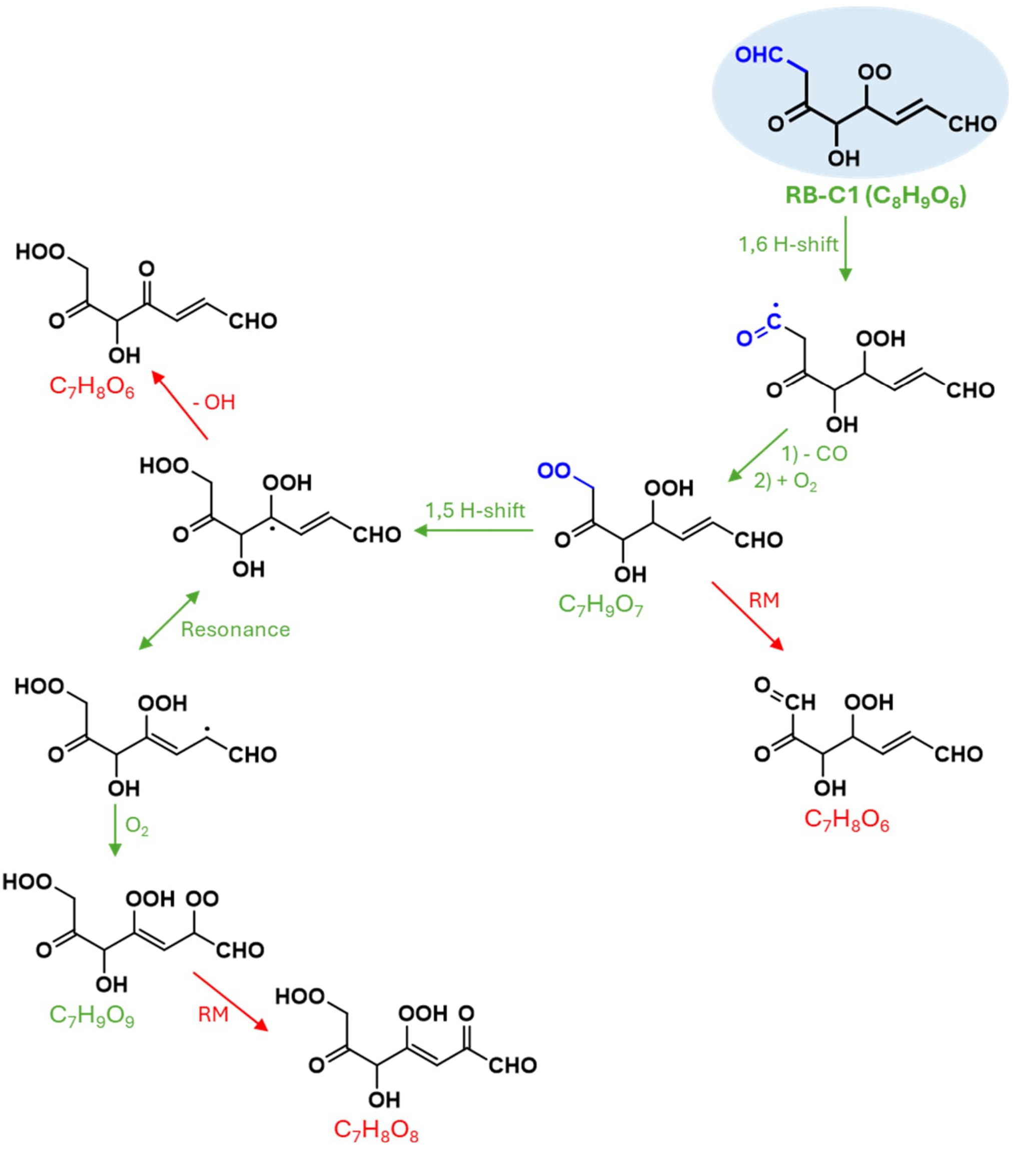

Supplementary Figure 20. Potential formation pathways of $C_7H_8O_{6,8}$ from peroxy radical RB-C1 ($C_8H_9O_6$) in phenylacetaldehyde oxidation. The green arrows indicate chain propagation reactions while the red arrows indicate chain termination reactions. RM = Russel mechanism ($RO_2 + R'O_2 \rightarrow ROH + R'_{-H}C{=}O + O_2$).

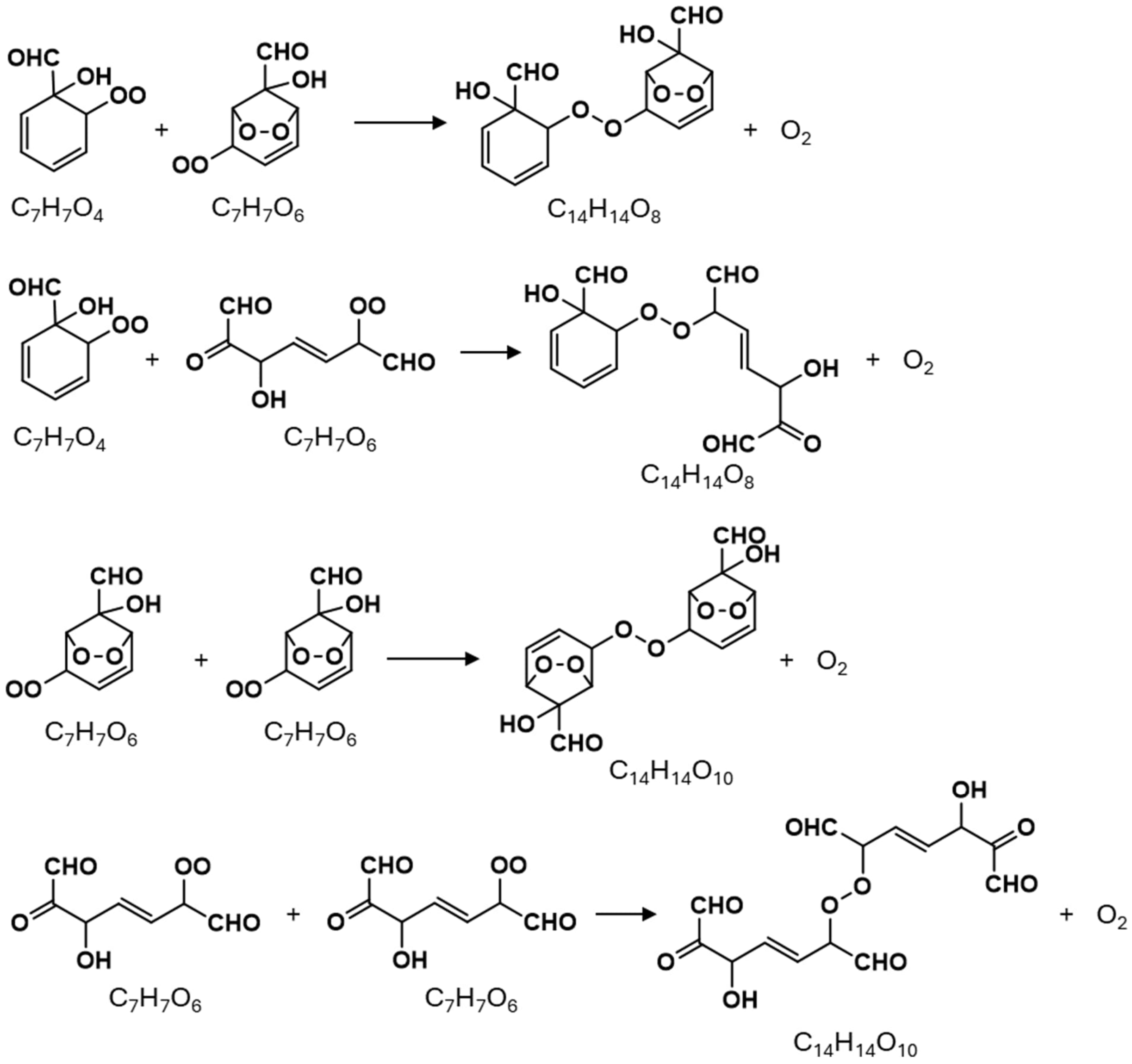

Supplementary Figure 21. Potential pathways of forming accretion products ($C_{14}H_{14}O_{8,10}$) by different combinations of monomeric alkyl peroxy (RO_2) radicals (i.e., RO_2 + $R'O_2$ = ($ROOR'$ + O_2) in benzaldehyde OH oxidation.

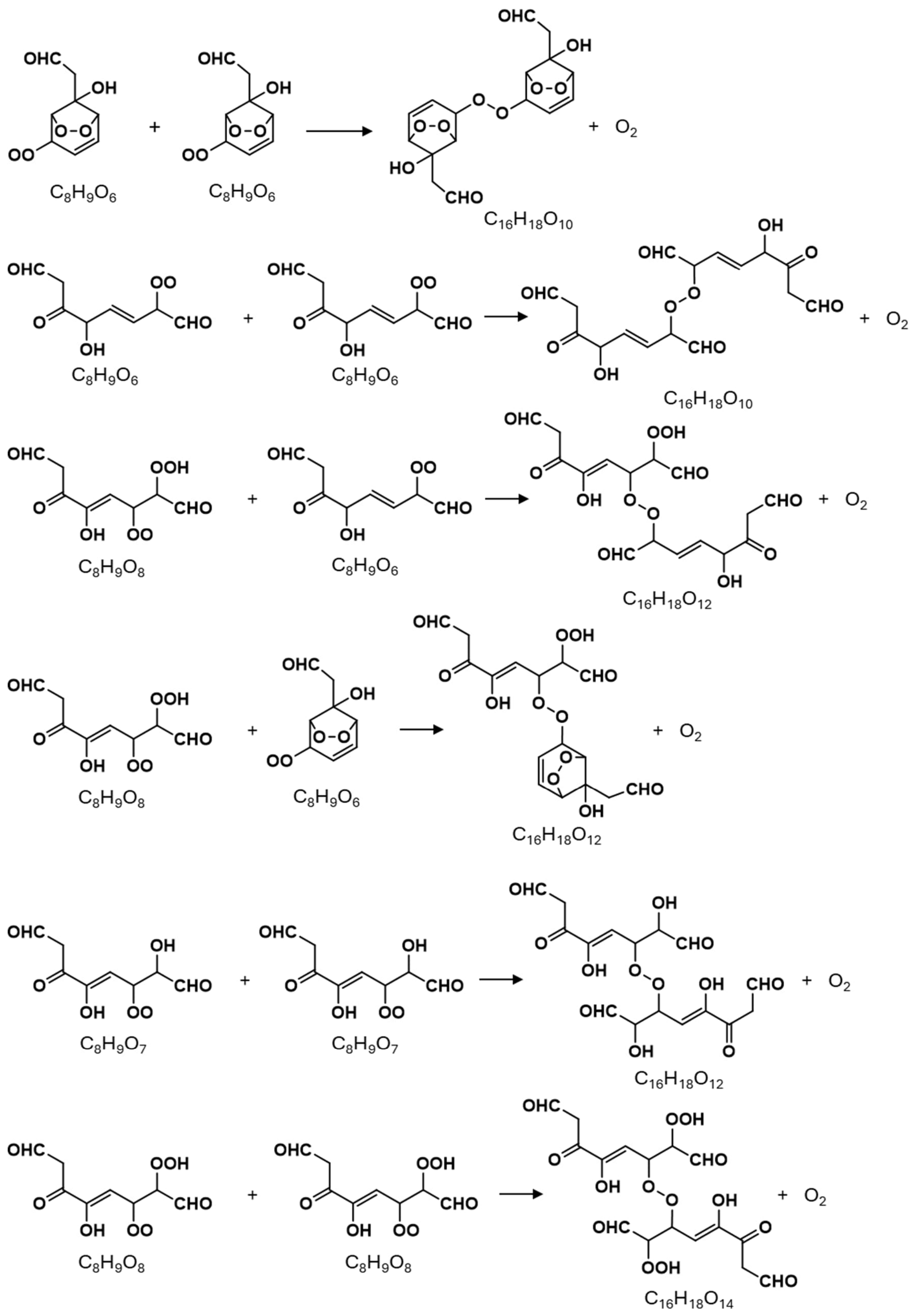

Supplementary Figure 22. Potential formation pathways of accretion products ($C_{16}H_{18}O_{10,12,14}$) by different combinations of monomeric alkyl peroxy (RO_2) radicals (i.e., RO_2 + $R'O_2$ = (ROOR' + O_2) in phenylacetaldehyde OH oxidation.

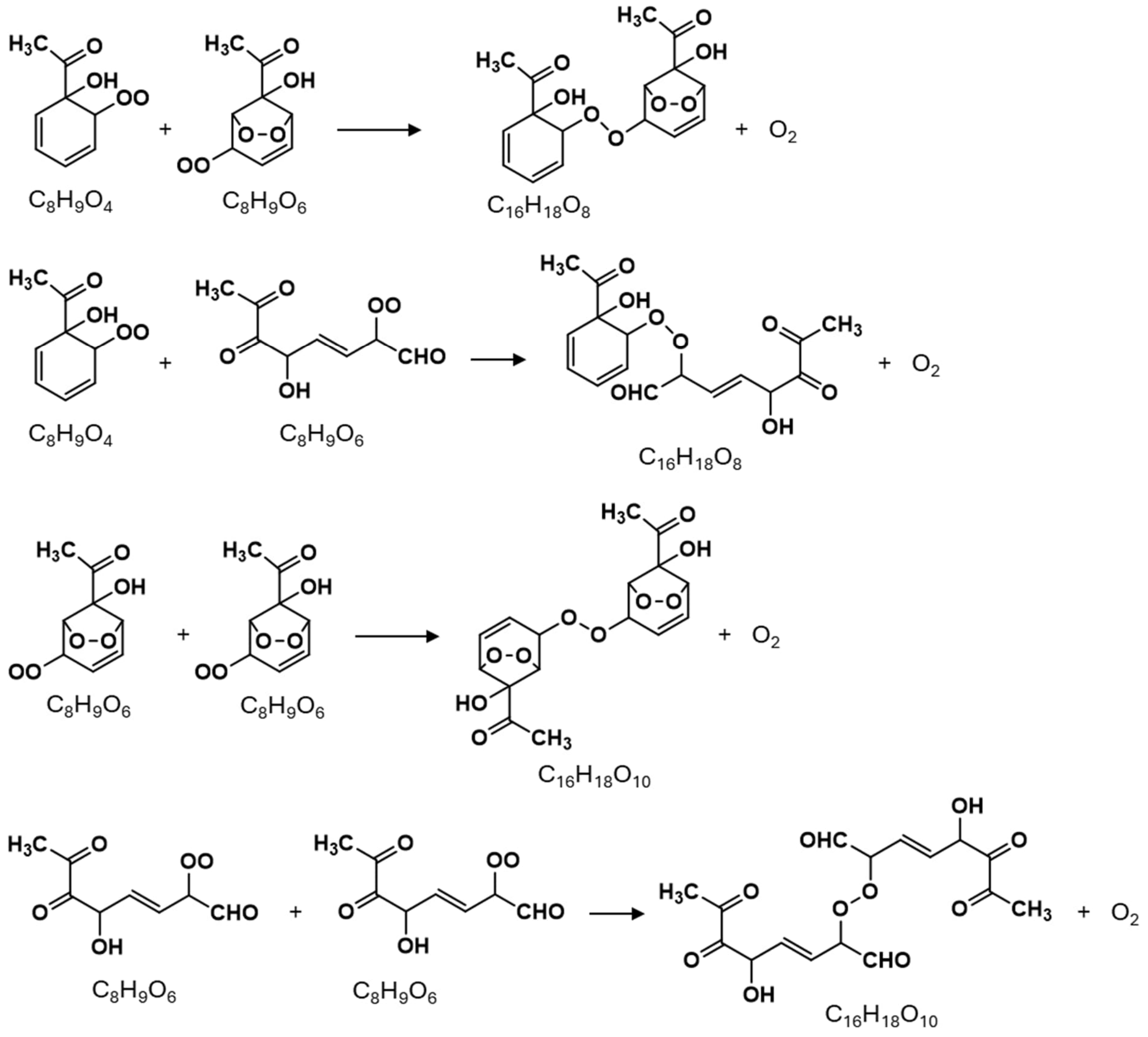

Supplementary Figure 23. Potential formation pathways of accretion products ($C_{16}H_{18}O_{8–10}$) by different combinations of monomeric alkyl peroxy (RO_2) radicals (i.e., RO_2 + $R'O_2$ = ($ROOR'$ + O_2) in acetophenone OH oxidation.

S14. Volatility classification and regression analysis

This section provides additional details on the prediction of vapor concentration of products (experiments in the presence of NO) and their classification across different volatility classes provided in Fig. 6 in the main manuscript. The oxidation products of phenylacetaldehyde, benzaldehyde, and acetophenone are categorized into volatility classes based on their predicted vapor concentrations, determined by averaging the results of six models: Nanoolal,[11] EVAP,[12] Myrdal-Yalkowsky,[13] SIMPOL,[14] Stolzenburg,[15] and Mohr.[16]

The scatter plots (see Supplementary Fig. 24) illustrate the relationship between saturation vapor concentration and molar mass (MM) for the compounds, as estimated using six vapor

pressure prediction models. The averaged values represent the average of these six predictions. Linear regression analyses were performed for the whole data of the six models (dashed black line) as well as exclusively for the averaged values (solid black line), demonstrating a clear correlation. These findings highlight the different vapor pressure trends based on different models while also emphasizing variations among individual estimates. Supplementary Fig. 25 presents pie charts illustrating the percentage distribution of the number of oxidation products across different volatility classes categorized based on their saturation mass concentration, C^* (μg m^{-3}), including ultra-low VOC (ULVOC, $C^* \leq 10^{-9}$ μg m^{-3}), extremely-low VOC (ELVOC, $10^{-9} < C^* \leq 10^{-5}$ μg m^{-3}), low VOC (LVOC, $10^{-5} < C^* \leq 10^{-1}$ μg m^{-3}), semi VOC (SVOC, $10^{-1} < C^* \leq 10^{2}$ μg m^{-3}), intermediate VOC (IVOC, $10^{2} < C^* \leq 10^{6}$ μg m^{-3}).[15]

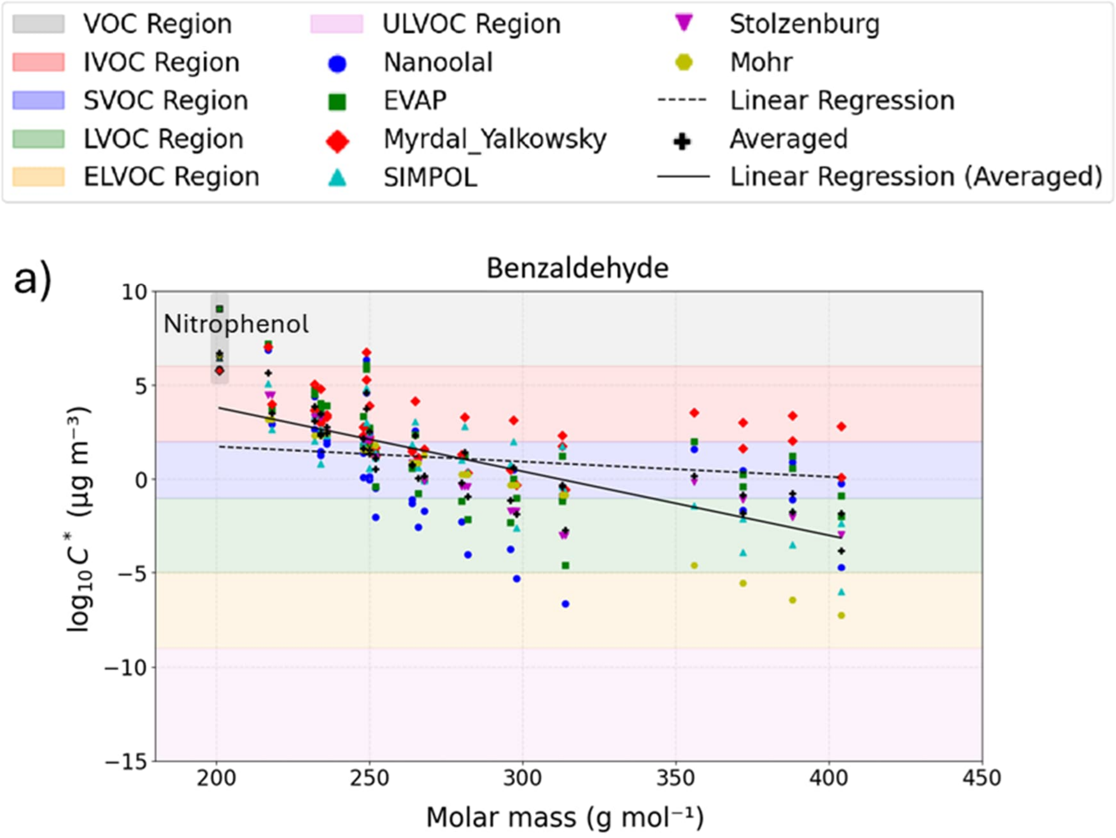

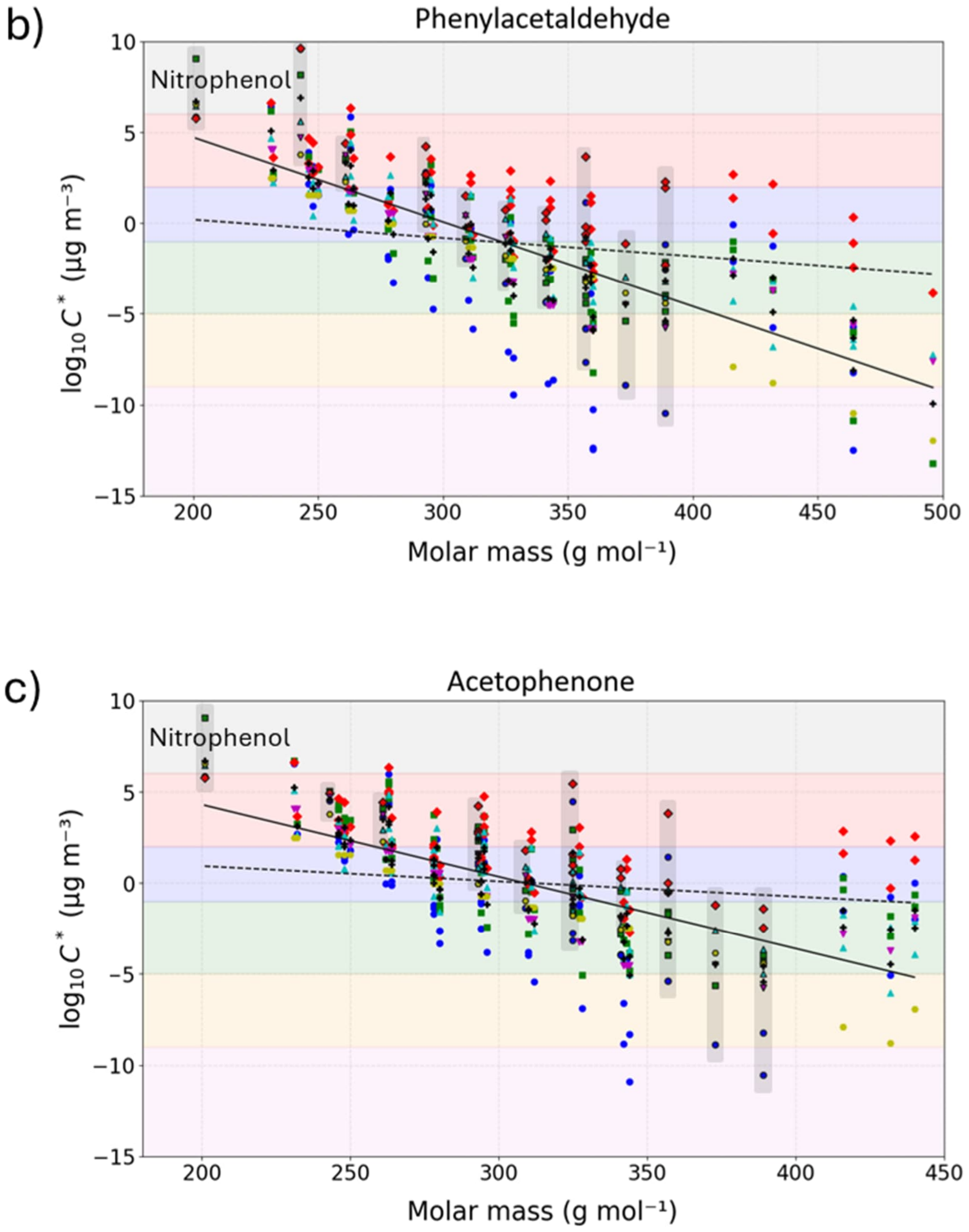

Supplementary Figure 24. The relationship between molar mass and $\log_{10}C^*$ of the detected oxidation products estimated using six vapor pressure prediction models. Different markers represent individual models, while nitrogen-containing compounds (mostly organonitrates except the nitrophenol) are highlighted with black borders within grey columns.

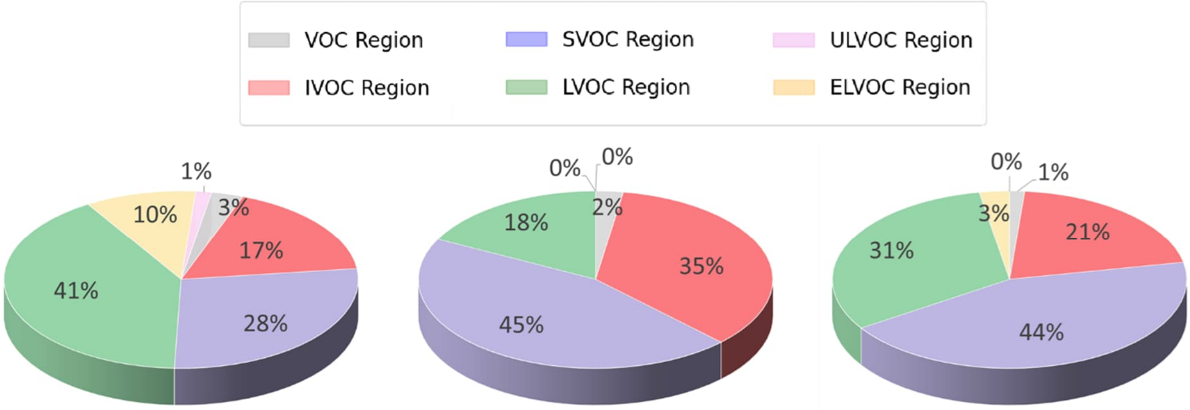

Phenylacetaldehyde **Benzaldehyde** **Acetophenone**

Supplementary Figure 25. Volatility classification of oxidation products from phenylacetaldehyde, benzaldehyde, and acetophenone (in the presence of 100 ppb NO). The classifications are based on the averaged vapor concentration predictions from six models: Nanoolal, EVAP, Myrdal-Yalkowsky, SIMPOL, Stolzenburg, and Mohr. The pie charts illustrate the percentage distribution of oxidation products across six volatility classes (ULVOC, ELVOC, LVOC, SVOC, IVOC, and VOC), determined using the averaged predictions from these models.

The correlation of the linear regression lines for the total dataset from all six models (dashed black line) and the averaged dataset (solid black line), as shown in Supplementary Fig. 24, is presented in Supplementary Table 6. The root mean square error (RMSE) values for both lines are calculated based on the entire dataset, meaning that for the solid black line derived from the averaged dataset, its RMSE is determined using all individual data points rather than the averaged values. Additionally, Supplementary Table 7 reports the RMS difference for each individual model (Nanoolal, EVAP, Myrdal-Yalkowsky, SIMPOL, Stolzenburg, and Mohr), representing the deviation of each model's predictions from the averaged linear regression line. These values quantify how much each model deviates from the overall trend predicted by the combined model set.

Supplementary Table 6. Linear regression equations and root mean square error (RMSE) values for vapor concentration predictions of oxidation products from acetophenone, benzaldehyde, and phenylacetaldehyde. The regression is performed using the total dataset from six models (dashed black line) and the averaged dataset (solid black line). RMSE values indicate the overall deviation of predictions from the regression line.

**Model**	**Linear Regression Formula**	**RMSE ($\mu g\ m^{-3}$)**
Acetophenone (total dataset)	$\log_{10}C^*$ = -0.0084 MM + 2.6053	3.0497
Acetophenone (averaged dataset)	$\log_{10}C^*$ = -0.0395 MM + 12.1966	1.4850
Benzaldehyde (total dataset)	$\log_{10}C^*$ = -0.0080 MM + 3.3261	2.7469
Benzaldehyde (averaged dataset)	$\log_{10}C^*$ = -0.0340 MM + 10.6084	1.2990
Phenylacetaldehyde (total dataset)	$\log_{10}C^*$ = -0.0101 MM + 2.2131	3.8112
Phenylacetaldehyde (averaged dataset)	$\log_{10}C^*$ = -0.0465 MM + 14.0257	1.5165

† MM = molar mass.

Supplementary Table 7. Root mean square (RMS) difference ($\mu g\ m^{-3}$) between individual model predictions (Nanoolal, EVAP, Myrdal-Yalkowsky, SIMPOL, Stolzenburg, and Mohr) and the averaged linear regression line for acetophenone, benzaldehyde, and phenylacetaldehyde. These values represent the extent to which each model deviates from the mean behavior across all models.

**Model**	**Acetophenone**	**Benzaldehyde**	**Phenylacetaldehyde**
Nanoolal	3.3433	2.7554	3.8556
EVAP	2.1908	2.2599	2.2300
Myrdal-Yalkowsky	2.7918	2.5636	3.4101
SIMPOL	1.4839	1.3361	1.4573
Stolzenburg	1.6269	1.3242	1.7134
Mohr	1.4483	1.7116	1.2121

S15. Kinetic simulation

S15.1 Simulations without and with NO

Chemical kinetic simulations are carried out using Kinetiscope Program[17,18] to estimate the concentrations of oxidant OH and primary RO_2 radicals in different aromatic carbonyl oxidation reactions without (reaction steps 1–3, 8–10 below) and with the presence of NO (reaction steps 1–12 below). We use an experimental rate coefficient of 1.2×10^{-11} cm^3 $molecule^{-1}$ s^{-1} for benzaldehyde (BZ) + OH reaction.[19] For the reactions of phenylacetaldehyde (PA) + OH and acetophenone (ACP) + OH, we do not find experimental rate coefficients in the literature and hence use SAR predicted rate coefficients of 4.19×10^{-11} and 3.86×10^{-12} cm^3 $molecule^{-1}$ s^{-1}, respectively for these reactions (see Supplementary Tables 2–3 in Section S4). In our flow reactor setup, we produce OH radicals in situ by the ozonolysis reaction of tetramethyl ethylene (TME). The produced OH radicals react with TME as well as with the aromatic carbonyl in the flow reactor. In the simulations, we use reaction rate coefficients k_{TME-O_3} of 1.5×10^{-15} and k_{TME-OH} of 1.0×10^{-10} cm^3 $molecule^{-1}$ s^{-1} accounting for the corresponding reactions.[20] As initial precursor concentrations, 1 ppm

(2.46×10^{13} molecules cm^{-3}) of VOC, 52–109 ppb ($1.28–2.68 \times 10^{12}$ molecules cm^{-3}) of TME, and 263–318 ppb ($6.47–7.82 \times 10^{12}$ molecules cm^{-3}) of ozone, identical to the experimental conditions, are used (see Table 1 of the main manuscript for details). In the flow reactor experiments, high VOC concentrations were used to scavenge OH, especially as TME reacts so fast with it. Following the reaction of aromatic carbonyl with OH, the initially formed carbon centered radical readily undergoes a pseudo unimolecular reaction with O_2 to form a primary RO_2 radical. For simplification, we show the formation of different RO_2 radicals directly from the reactions of aromatic carbonyl with OH, and TME with OH. Here, TME produced RO_2 and aromatic carbonyl produced RO_2 are separated by the expressions RO_2_T and RO_2_C respectively.

With the addition of NO in the reaction system, it is expected to influence the concentrations of OH radicals and primary RO_2 radicals. Therefore, to simulate the experiments with NO, we include the bimolecular reaction rate coefficients of $k_{NO-OH} = 3.3 \times 10^{-11}$, $k_{HONO-OH} = 6.0 \times 10^{-12}$, $k_{NO-O_3} = 1.8 \times 10^{-14}$, and $k_{NO_2-OH} = 4.1 \times 10^{-11}$ cm^3 molecule^{-1} s^{-1},[21] with respect to their corresponding reactions in the simulation. Besides, bimolecular rate coefficients for RO_2 + RO_2 and RO_2 + NO reactions are set to the generic values of 3.2×10^{-11} and 9.0×10^{-12} cm^3 molecule^{-1} s^{-1}, respectively[22,23] to account for sinks of RO_2 radicals. The influences of NO + HO_2, RO_2 + HO_2, and RO_2 + NO_2 reactions on OH and RO_2_C radical concentrations are examined separately and discussed in the section below. An example of the reaction steps used in the current simulation is as follows. The results are shown in Supplementary Fig. 26 and Supplementary Table 8.

1. TME + O_3 => OH ($k_{TME-O_3} = 1.5 \times 10^{-15}$ cm^3 molecule^{-1} s^{-1})
2. TME + OH => RO_2_T ($k_{TME-OH} = 1.0 \times 10^{-10}$ cm^3 molecule^{-1} s^{-1})
3. PA + OH => RO_2_C ($k_{PA-OH} = 4.24 \times 10^{-11}$ cm^3 molecule^{-1} s^{-1})
4. NO + OH => HONO ($k_{NO-OH} = 3.3 \times 10^{-11}$ cm^3 molecule^{-1} s^{-1})
5. HONO + OH => H_2O + NO_2 ($k_{HONO-OH} = 6.0 \times 10^{-12}$ cm^3 molecule^{-1} s^{-1})
6. NO + O_3 => NO_2 + O_2 ($k_{NO-O_3} = 1.8 \times 10^{-14}$ cm^3 molecule^{-1} s^{-1})
7. NO_2 + OH => HNO_3 ($k_{NO_2-OH} = 4.1 \times 10^{-11}$ cm^3 molecule^{-1} s^{-1})
8. 2 RO_2_T => Sink_a ($k_{RO_2-RO_2} = 3.2 \times 10^{-11}$ cm^3 molecule^{-1} s^{-1})
9. 2 RO_2_C => Sink_b ($k_{RO_2-RO_2} = 3.2 \times 10^{-11}$ cm^3 molecule^{-1} s^{-1})
10. RO_2_T + RO_2_C => Sink_ab ($k_{RO_2-RO_2} = 3.2 \times 10^{-11}$ cm^3 molecule^{-1} s^{-1})
11. RO_2_T + NO => $RONO_2$ ($k_{RO_2-NO} = 9.0 \times 10^{-12}$ cm^3 molecule^{-1} s^{-1})

12. RO_2_C + NO => $RONO_2$ (k_{RO_2-NO} = 9.0×10^{-12} cm^3 $molecule^{-1}$ s^{-1})

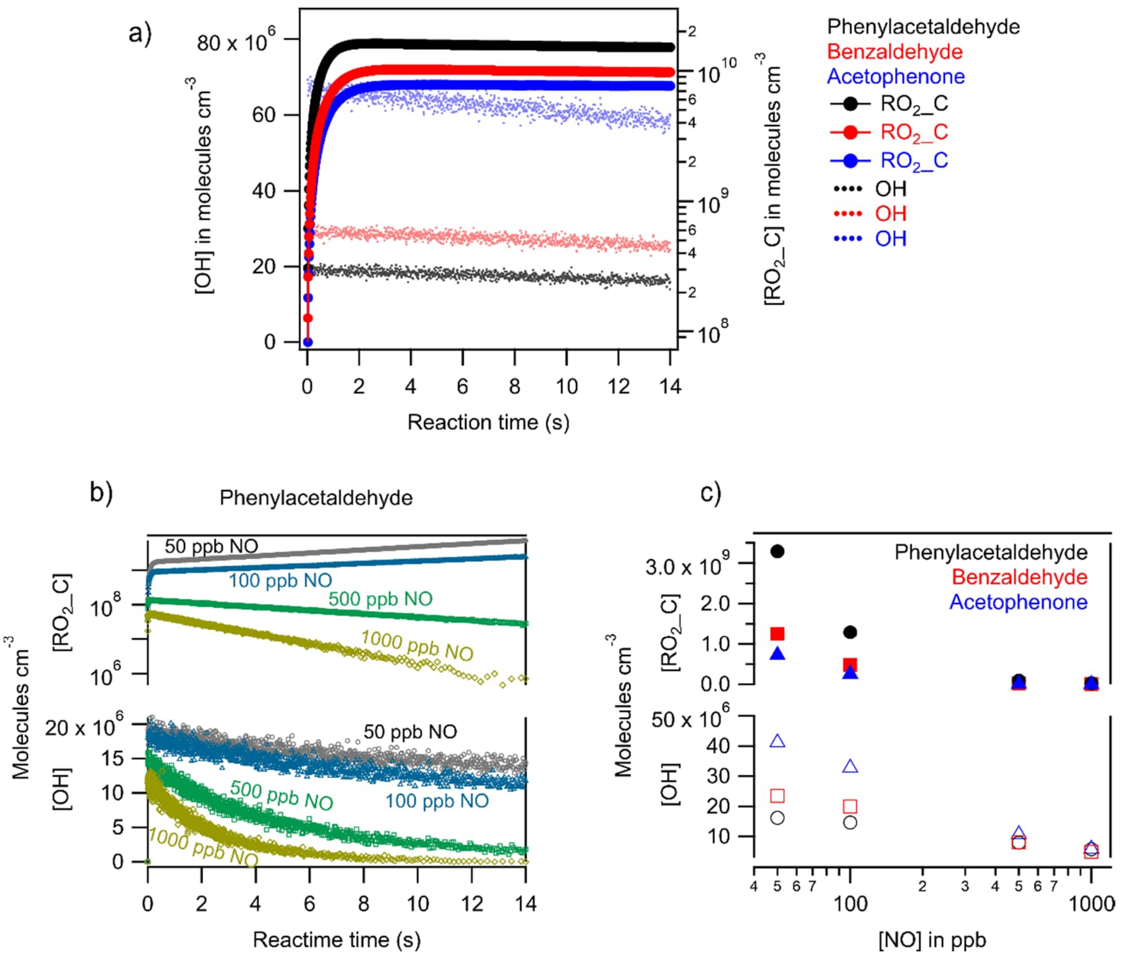

Supplementary Figure 26. Concentration profiles of OH and primary RO_2_C radicals produced in aromatic carbonyl oxidation derived by chemical kinetic simulations under laboratory flow reactor experimental condition without NO (a) and with NO (b–c). Panel (b) shows results from phenylacetaldehyde. In panel (c), average concentrations of OH and RO_2_C during 14 s reaction time are presented in open and filled markers, respectively.

Supplementary Fig. 26a and Supplementary Table 8 imply that the average concentrations of oxidant OH and primary RO_2_C radicals in the flow reactor are comparable in different aromatic carbonyl oxidation experiments. In the experiments without NO, the simulation produced average concentrations of OH radicals are 2.75×10^7, 1.77×10^7, and 6.26×10^7 molecules cm^{-3} for the experiments with BZ, PA, and ACP, respectively.

Supplementary Table 8. Average concentrations of OH and RO_2 radicals produced in different aromatic carbonyl oxidation experiments derived from chemical simulations under laboratory conditions. Reaction time, Δt = 14 s.

**Expt. type**	**Model input**				**Model output**	
**(VOC)**	**[VOC] ppmv**	**[TME] ppbv**	**[O_3] ppbv**	**$[NO]_{t=0}$ ppbv**	**[OH] #/cm^3**	**[RO_2_C] #/cm^3**
**Simulation without NO**						
BZ	1	52.4	263	–	2.75×10^{7}	9.50×10^{9}
PA	1	104.8	263	–	1.77×10^{7}	1.51×10^{10}
ACP	1	52.4	318	–	6.26×10^{7}	7.30×10^{9}
**In the presence of NO**						
BZ	1	52.4	263	50	2.35×10^{7}	1.25×10^{9}
				100	2.00×10^{7}	4.79×10^{8}
				500	8.11×10^{6}	2.68×10^{7}
				1000	4.99×10^{6}	7.33×10^{6}
PA	1	104.8	263	50	1.62×10^{7}	3.29×10^{9}
				100	1.47×10^{7}	1.29×10^{9}
				500	7.99×10^{6}	9.23×10^{7}
				1000	5.86×10^{6}	3.04×10^{7}
ACP	1	52.4	263	50	4.12×10^{7}	7.24×10^{8}
				100	3.28×10^{7}	2.58×10^{8}
				500	1.07×10^{7}	1.15×10^{7}
				1000	5.90×10^{6}	2.83×10^{6}

The corresponding RO_2_C radical concentrations are 9.50×10^{9}, 1.51×10^{10}, and 7.30×10^{9} molecules cm^{-3}, respectively. While the OH concentrations for BZ and PA experiments are closer to each other, it is higher for the ACP experiment by a factor of 2 to 3. This can be attributed to the slower OH reactivity with ACP compared to BZ and PA (see Section S4 above). It is also reflected in the concentrations of primary RO_2_C radical in the three studied systems, with PA yielding the highest RO_2_C concentration as it has the highest reactivity with OH of the three molecules. In the presence of NO, Supplementary Fig. 26b shows how the concentration profiles of OH and RO_2_C radicals evolve in time with PA oxidation experiment as an example. In all the aromatic carbonyl systems studied, the primary RO_2_C radical concentration steadily increases with time under 50 ppb and 100 ppb NO conditions. However, at 500 ppb and 1000 ppb NO conditions, the concentrations of RO_2_C radicals decrease with time soon after achieving their initial peak values. In the case of OH radicals, the concentrations show a general decreasing trend with time while at higher NO conditions, we observe a faster decreasing tendency in concentrations as expected. It is also interesting to see that the higher OH reactivity of PA compared to the other two aromatic carbonyls is reflected even under high NO conditions in terms of the PA-derived primary RO_2_C radical concentration being higher than that of BZ and ACP (see Supplementary Fig. 26c and Supplementary Table 8).

It is also important to note that NO_2 is produced in the flow reactor via reactions of NO, while NO is directly injected into the reactor from a cylinder. The time series of NO, NO_2, and aromatic carbonyls under different initial NO conditions are shown in Supplementary Fig. 27. While calculating HOM yields (Fig. 4 in the main manuscript), the consumed aromatic carbonyl concentrations (ΔVOC) were estimated from the difference of initial and final concentrations (see Supplementary Fig. 27b,d,f). Under 100 ppb $[NO]_{t=0}$ condition, the ΔVOC concentrations were 7.82×10^{10}, 2.02×10^{11}, and 4.22×10^{10} molecules cm^{-3} of benzaldehyde, phenylacetaldehyde, and acetophenone, respectively.

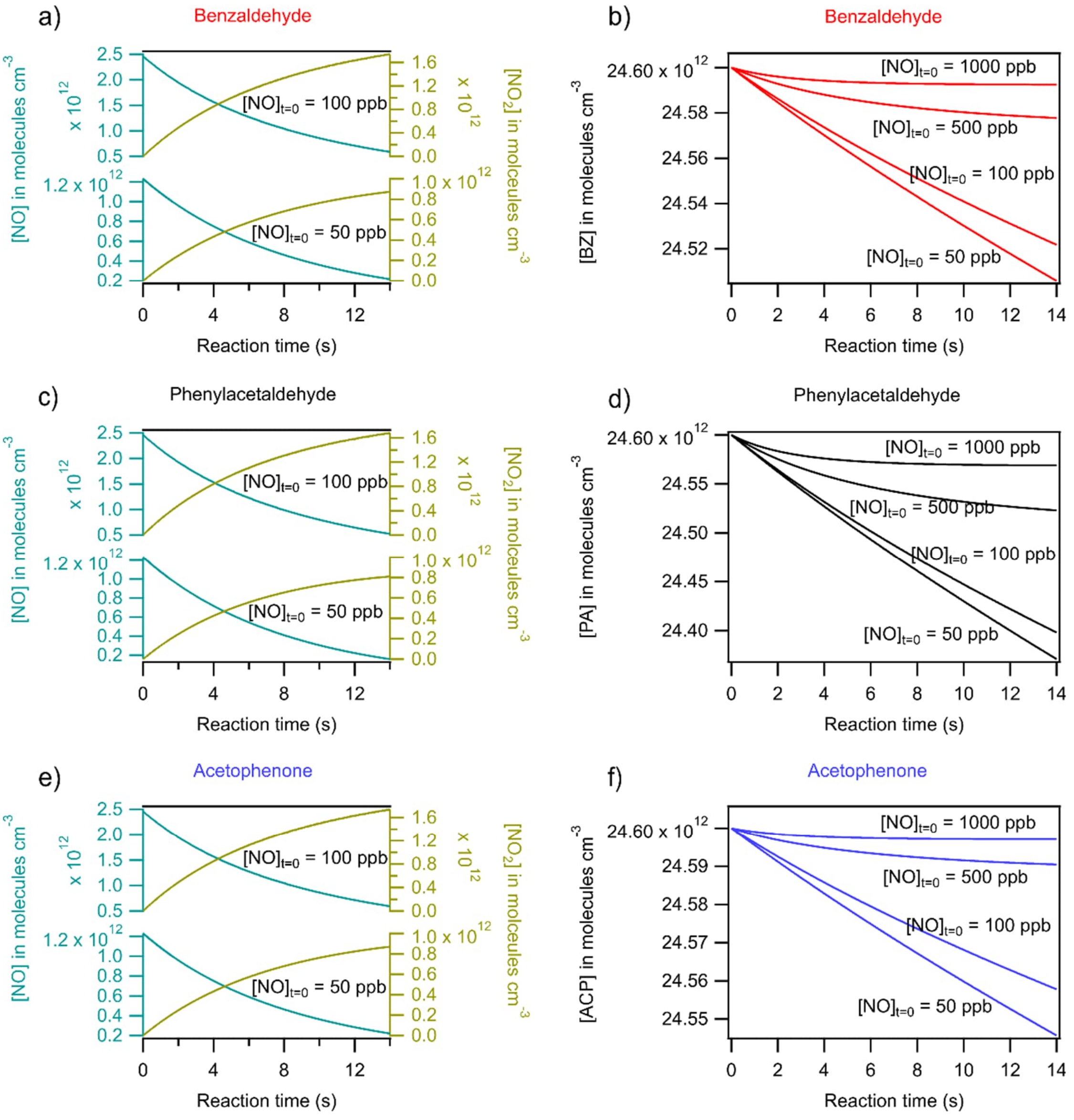

Supplementary Figure 27. Concentration profiles of NO, NO_2, and aromatic carbonyls as a function of reaction time under different initial $[NO]_{t=0}$ conditions: benzaldehyde (a–b), phenylacetaldehyde (c–d), and acetophenone (e–f). Reaction time, Δt = 14 s. BZ = benzaldehyde, PA = phenylacetaldehyde, and ACP = acetophenone.

S15.2 Impact of HO_2 and PAN on OH and RO_2

In the gas-phase oxidation process of volatile organic compounds (VOCs), it is usual to produce hydroperoxy radicals (HO_2) alongside the production of alkyl peroxy radicals (RO_2). In the presence of NO, the HO_2 radicals react with NO to recycle OH and NO_2 radicals in the reaction system (see reaction step 13).[21] In addition, the reaction of HO_2 with RO_2 can produce closed-shell hydroperoxide (ROOH) along with other products.[24] Here, we include these reactions as other sinks of RO_2 in the simulation (reaction steps 14–15).

13. HO_2 + NO => OH + NO_2 (k_{HO_2-NO} = 8.91×10^{-12} cm^3 $molecule^{-1}$ s^{-1})
14. RO_2_T + HO_2 => Sink_c ($k_{RO_2-HO_2}$ = 2.2×10^{-11} cm^3 $molecule^{-1}$ s^{-1})
15. RO_2_C + HO_2 => Sink_d ($k_{RO_2-HO_2}$ = 2.2×10^{-11} cm^3 $molecule^{-1}$ s^{-1})

In the flow reactor system, we presume that the production of HO_2 radicals is about 30% of total primary RO_2 radicals (RO_2_T + RO_2_C). Therefore, to examine the influence of the HO_2 reactions with NO and RO_2 on the concentrations of OH and RO_2_C radicals, we run a separate set of simulations on Kinetiscope including the reaction steps 13–15. An initial HO_2 concentration of 30% of total RO_2 (e.g., 4.78×10^8 molecules cm^{-3} of HO_2 in PA oxidation at 100 ppb NO) obtained from the previous simulation without HO_2 is used in the subsequent simulation. Supplementary Fig. 28a shows the results of PA oxidation process without and with involving the reaction steps 13–15. In all aromatic carbonyl simulations, the reaction steps 13–15 do not seem to alter the average concentrations of OH and RO_2_C radicals given that the reaction scheme (reaction steps 1–15) is lacking a constant source of 30% HO_2.

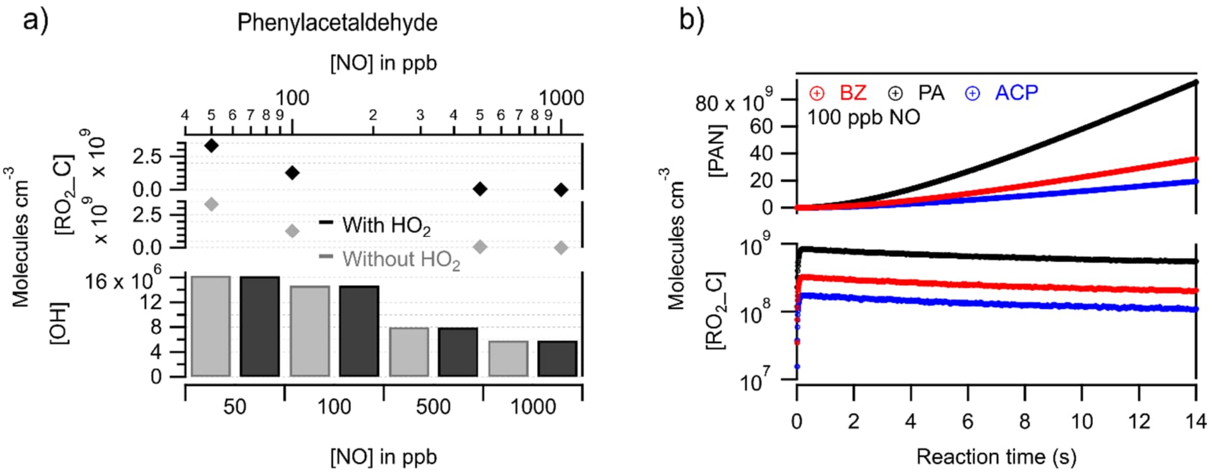

Supplementary Figure 28. Average concentrations of OH and primary RO_2_C radicals produced in phenylacetaldehyde oxidation derived by chemical kinetic simulations under laboratory conditions with varying NO (a). In panel (a), simulations that include the reactions

of HO_2 with NO and RO_2 (reaction steps 13–15) are presented in black, while simulations without these reaction steps are presented in grey. Panel (b) shows the time series of RO_2_C radicals, and maximum possible peroxyacyl nitrate (PAN) concentrations formed in different aromatic carbonyl OH oxidation reactions under 100 ppb initial NO condition. PA = phenylacetaldehyde (black), ACP = acetophenone (blue), and BZ = benzaldehyde (red).

The aromatic carbonyl produced RO_2_C reacts with NO_2 also that is produced in situ in the flow reactor oxidation system according to reaction steps 5 and 6. Here, the reaction of RO_2_C with NO_2 acts as a sink of RO_2_C producing organic nitrates including peroxyacyl nitrates (PAN). We estimate a maximum possible PAN formation by including the reaction step 13' in another separate set of simulations. The idea is to observe to what extent the NO_2 reaction drops the average concentration of RO_2_C limiting its potential to oxidize further to form HOM. Alkyl and acyl peroxy radicals react with NO_2 with rate coefficients of 9.0×10^{-12} and 1.1×10^{-11} cm^3 $molecule^{-1}$ s^{-1}, respectively.[23] Because we cannot differentiate between the two peroxy radicals in a pool of total RO_2_C, an average rate coefficient $k_{RO_2-NO_2}$ of 1.0×10^{-11} cm^3 $molecule^{-1}$ s^{-1} is used in the simulation.

13'. RO_2_C + NO_2 => PAN ($k_{RO_2-NO_2}$ = 1.0×10^{-11} cm^3 $molecule^{-1}$ s^{-1})

The simulation results are presented in Supplementary Table 9, and the time series of RO_2_C and PAN are shown in Supplementary Fig. 28b. With the inclusion of reaction step 13', we observe the drop of RO_2_C concentrations only by a factor of up to 2 in different aromatic carbonyl oxidation simulations. Supplementary Fig. 28b shows that at 100 ppb NO condition, the average concentrations RO_2_C are 2.60×10^8, 6.83×10^8, and 1.41×10^8 cm^3 $molecule^{-1}$ s^{-1} for BZ, PA, and ACP oxidation, respectively. On the other hand, the average concentrations of PAN (maximum) are 1.02×10^{10}, 2.71×10^{10}, and 5.47×10^9 cm^3 $molecule^{-1}$ s^{-1} for BZ, PA, and ACP oxidation, respectively, at 100 ppb NO condition. Because the primary source of acyl peroxy radical is the aldehydic H abstraction by OH in BZ and PA but not in ACP (see branching ratios in Supplementary Tables 1–3, Section S4), the actual PAN concentrations formed via reaction step 13' are less than values reported here. This implies that despite the formation of PAN, the concentrations of peroxy radicals produced in the studied systems are significant enough to follow further oxidation and form HOMs as observed experimentally. Note that in the simulation model the influence of NO is reflected as the suppression of peroxy radicals RO_2_C as expected and hence the experimental observation of

its enhancing effect on HOM likely involves alkoxy (RO) mediated chemistry that is not included in the model. Incorporating the RO mediated complex chemistry in the model will require extensive molecular level quantum chemical calculations which are unfeasible. It is also important to mention that the concentrations of oxidant OH and primary RO_2_C radicals reported here are at their upper limits as the loss of the radicals to the reactor wall is not accounted.

Supplementary Table 9. Average concentrations of OH, RO_2 radicals, and PAN produced in different aromatic carbonyl oxidation experiments derived from chemical simulations including the reaction step RO_2_C + NO_2 → PAN under laboratory conditions. Reaction time, Δt = 14 s.

Expt.	$[NO]_{t=0}$ ppbv	[OH] #/cm^3	[RO_2_C] #/cm^3	[PAN] #/cm^3
BZ	50	2.35×10^7	6.19×10^8	1.33×10^{10}
	100	2.00×10^7	2.60×10^8	1.02×10^{10}
	500	8.11×10^6	2.14×10^7	1.83×10^9
	1000	4.99×10^6	6.60×10^6	3.55×10^8
PA	50	1.62×10^7	1.57×10^9	3.38×10^{10}
	100	1.46×10^7	6.83×10^8	2.71×10^{10}
	500	7.98×10^6	7.38×10^7	6.31×10^9
	1000	5.86×10^6	2.74×10^7	1.47×10^9
ACP	50	4.11×10^7	3.56×10^8	7.63×10^9
	100	3.29×10^7	1.41×10^8	5.47×10^9
	500	1.07×10^7	9.19×10^6	7.87×10^8
	1000	5.90×10^6	2.55×10^6	1.36×10^8

Therefore, in our flow reactor experiments, the concentrations of the reactive radical species are very close to the expected concentrations in the ambient air, and we believe they are well representative.

S15.3 From laboratory conditions to ambient atmospheric conditions

We observe rapid formation of HOM from aromatic carbonyl OH oxidation reactions in as low as sub-second timescale for PA, conducted in the laboratory flow reactor setup. To inspect the feasibility of HOM formation from OH initiated oxidation of aromatic carbonyls (BZ, PA, and ACP) in the ambient air, we simulate the oxidation reactions under atmospherically relevant conditions of the studied VOCs and reactive partners. The ambient concentrations of these aromatic carbonyls are less than 1 ppb.[25,26] However, in indoor air, the concentrations can be significantly higher emitting from heated electronics, and consumer products. For example, in

products like detergents, lotions, and perfumes, the concentrations of acetophenone can range from several tens to hundreds of ppm.[27] In our simulation, considering an ambient atmosphere, the initial concentration aromatic carbonyl is given 1ppb ($2.46\times\ 10^{10}$ molecules cm^{-3}). A generic concentration of oxidant [OH] = $1.0\times\ 10^{7}$ molecules cm^{-3} is used. Two different concentrations of NO (0.1 and 1 ppb, i.e., $2.46\times\ 10^{9}$, and $2.46\times\ 10^{10}$ molecules cm^{-3}) are used to mimic moderately clean to low NO_x conditions. Considering a lower limit of atmospheric RO_2 as $1.0\times\ 10^{7}$ molecules cm^{-3} in VOC limited condition and a higher limit of atmospheric RO_2 as $1.5\times\ 10^{9}$ molecules cm^{-3} in NO_x limited condition, we use a generic value of [RO_2] = $5.0\times\ 10^{8}$ molecules cm^{-3} in the simulation. The concentration of HO_2 radicals is assumed to be the same as RO_2 so that we maintain an initial [HO_2]/]RO_2] = 1. We also consider that the carbon centered radical intermediates can readily add an oxygen molecule forming alkyl (or acyl) peroxy radicals (RO_2 or $RC(O)O_2$) via fast pseudo-unimolecular reactions and hence we skip the reaction step for simplification as explained earlier. In a first set of simulations, we run reaction steps 1–8 (see below) and estimate the average concentrations of primary peroxy radicals RO_2_C. Knowing the branching ratios of OH addition to the *ipso* position of different aromatic carbonyls which are 2.64, 4.80, and 18.71 % respectively for BZ, PA, and ACP (see Section S4 above), we consider that roughly 1 % of RO_2_C goes to *ipso* bicyclic peroxy radical (*ipso*-BPR) intermediate in BZ and PA oxidation, and 10 % of RO_2_C goes to *ipso*-BPR in ACP oxidation processes. Accordingly, in a second set of simulations, we give relevant initial concentration of BPR and run the whole reaction scheme (steps 1–13 below) to estimate the production of O_8 HOM from PA and ACP. The *ipso*-BPR molecular rearrangement reaction rates $k_{BPR-MR-C2}$ of $5.0\times\ 10^{-3}$, 0.6, and $5.3\times\ 10^{-3}$ s^{-1} are used for BZ, PA, and ACP, respectively, obtained from our quantum chemical computations. The subsequent 1,6 H-shift rate coefficient $k_{1,6\ H-shift}$were computed for PA and ACP to compare the speediness of their overall autoxidation rates. These rates explain the difference in the influence of NO between the two aromatic carbonyls observed experimentally. The HOM_O_8 (reaction step 13 below) is shown to produce directly after the 1,6 H-shift reaction of BPR_C2. Here, a pseudo-unimolecular O_2 addition reaction (converting O_6 to O_8) is embedded in that reaction step. We did not compute the 1,6 H-shift rate coefficient after the BPR rearrangement for BZ. Therefore, in the case of BZ, we run the simulation with reaction steps 1– 12. One example of the reaction steps is as follows:

1. PA + OH => RO_2_C (k_{PA-OH} = $4.24\times\ 10^{-11}$ cm^3 molecule^{-1} s^{-1})
2. NO + OH => HONO (k_{NO-OH} = $3.3\times\ 10^{-11}$ cm^3 molecule^{-1} s^{-1})

3. HONO + OH => H_2O + NO_2 ($k_{HONO-OH}$ = 6.0×10^{-12} cm^3 $molecule^{-1}$ s^{-1})
4. NO_2 + OH => HNO_3 (k_{NO_2-OH} = 4.1×10^{-11} cm^3 $molecule^{-1}$ s^{-1})
5. RO_2_C + RO_2 => Sink_a ($k_{RO_2-RO_2}$ = 3.2×10^{-11} cm^3 $molecule^{-1}$ s^{-1})
6. RO_2_C + NO => $RONO_2$ (k_{RO_2-NO} = 9.0×10^{-12} cm^3 $molecule^{-1}$ s^{-1})
7. RO_2 + HO_2 => Sink_b ($k_{RO_2-HO_2}$ = 2.2×10^{-11} cm^3 $molecule^{-1}$ s^{-1})
8. RO_2_C + HO_2 => Sink_c ($k_{RO_2-HO_2}$ = 2.2×10^{-11} cm^3 $molecule^{-1}$ s^{-1})
9. BPR => BPR_C2 ($k_{BPR-MR-C2}$ = 0.6 s^{-1})
10. BPR + RO_2 => Sink_d ($k_{RO_2-RO_2}$ = 3.2×10^{-11} cm^3 $molecule^{-1}$ s^{-1})
11. BPR + NO => BPR_NO (k_{RO_2-NO} = 9.0×10^{-12} cm^3 $molecule^{-1}$ s^{-1})
12. BPR + HO_2 => Sink_e ($k_{RO_2-HO_2}$ = 2.2×10^{-11} cm^3 $molecule^{-1}$ s^{-1})
13. BPR_C2 => HOM_O_8 ($k_{1,6\,H-shift}$ = 0.9 s^{-1})

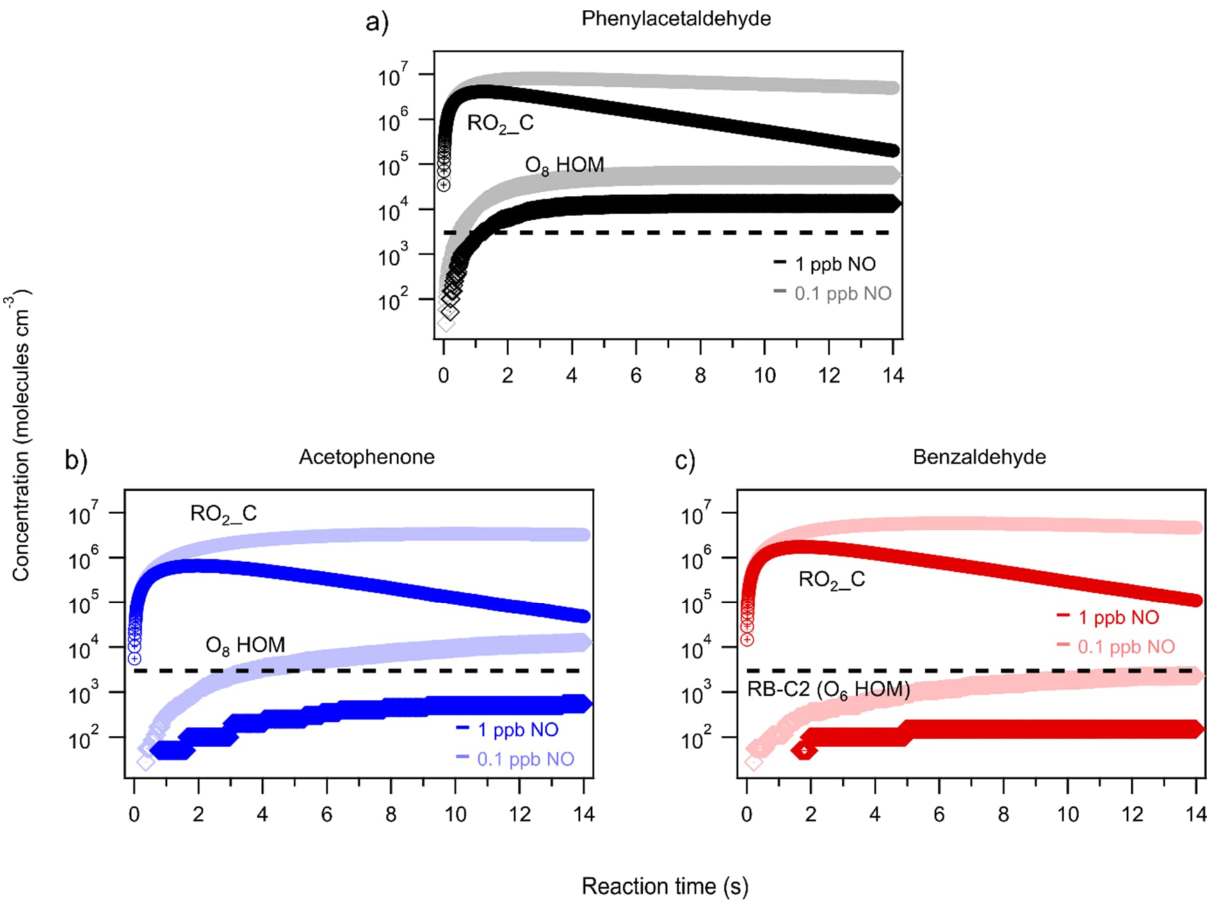

Supplementary Figure 29. Time series of primary peroxy radicals (RO_2_C) and HOMs produced in aromatic carbonyl OH oxidation reaction simulated under atmospheric conditions (a–c). Reactant concentrations: aromatic carbonyl = 1ppb (2.46×10^{10} molecules cm^{-3}), OH = 1.0×10^{7} molecules cm^{-3}, NO = 0.1, 1 ppb (2.46×10^{9}, 2.46×10^{10} molecules cm^{-3}), and RO_2 = 5.0×10^{8} molecules cm^{-3}. The black dashed line indicating a reference

concentration of 3.0×10^3 molecules cm^{-3} intersects the product curves at different reaction times. In panel (a), the O_8 HOM reaches the reference concentration before 1 s. In panels (b) and (c), the O_8 HOM and RB-C2 (O_6 HOM), respectively, reach the reference concentration in varying reaction times.

In the case of PA, Supplementary Fig. 29a shows that in reference to a concentration of 3.0×10^3 molecules cm^{-3}, O_8 HOM appears in sub-second reaction time in both 0.1 and 1 ppb NO conditions. This observation is completely in line with the experimental observation of HOMs within 0.9 s from PA oxidation initiated by OH. For acetophenone in Supplementary Fig. 29b, the O_8 HOM with a concentration of 3.0×10^3 molecules cm^{-3} is formed around 3 s reaction time at 0.1 ppb NO condition, still in close agreement with the experimental observation of HOMs within 2.7s. On the other hand, the production of ring-opened O_6 HOM in BZ oxidation (Supplementary Fig. 29c) seems to be rather slow compared to the experimental observation of HOMs. This is important to note that HOM concentrations in Supplementary Fig. 29 are the lower limits of individual HOM production based on the contribution of only *ipso*-BPR. If there is a possibility of forming HOM from other BPR channels, the actual concentration of these products will be more than what is seen here. In the case of OH initiated oxidation of BZ, the simulation results (concentration of RO_2_C vs O_6 HOM) also indicate that the reaction channel involving the aldehydic H abstraction by OH may contribute to especially C_{x-1} HOM and hence any yet undiscovered pathway along this channel can be of importance. The experimental results shown in Fig. 1 (short reaction time experiments) of the main manuscript were conducted without NO addition. However, these simulations that include the reactions of NO with other radical species have only the suppression effect as discussed before. Therefore, it is important to remember the reaction conditions while making a direct comparison between the experiment and simulation. Nevertheless, the fast formation of HOMs in aromatic carbonyl oxidation with OH in the laboratory flow reactor system is very well reflected in the simulation model operated under atmospheric conditions.

Supplementary References

(1) Sipilä, M.; Sarnela, N.; Jokinen, T.; Junninen, H.; Hakala, J.; Rissanen, M. P.; Praplan, A.; Simon, M.; Kürten, A.; Bianchi, F.; Dommen, J.; Curtius, J.; Petäjä, T.; Worsnop, D. R. Bisulfate – Cluster Based Atmospheric Pressure Chemical Ionization Mass Spectrometer for High-Sensitivity (< 100 ppqV) Detection of Atmospheric Dimethyl Amine: Proof-of-Concept and First Ambient Data from Boreal Forest. *Atmospheric Meas. Tech.* **2015**, *8* (10), 4001–4011. https://doi.org/10.5194/amt-8-4001-2015.

(2) Barua, S.; Iyer, S.; Kumar, A.; Seal, P.; Rissanen, M. An Aldehyde as a Rapid Source of Secondary Aerosol Precursors: Theoretical and Experimental Study of Hexanal Autoxidation. *Atmospheric Chem. Phys.* **2023**, *23* (18), 10517–10532. https://doi.org/10.5194/acp-23-10517-2023.

(3) Kürten, A.; Rondo, L.; Ehrhart, S.; Curtius, J. Calibration of a Chemical Ionization Mass Spectrometer for the Measurement of Gaseous Sulfuric Acid. *J. Phys. Chem. A* **2012**, *116* (24), 6375–6386. https://doi.org/10.1021/jp212123n.

(4) Jenkin, M. E.; Valorso, R.; Aumont, B.; Rickard, A. R.; Wallington, T. J. Estimation of Rate Coefficients and Branching Ratios for Gas-Phase Reactions of OH with Aliphatic Organic Compounds for Use in Automated Mechanism Construction. *Atmospheric Chem. Phys.* **2018**, *18* (13), 9297–9328. https://doi.org/10.5194/acp-18-9297-2018.

(5) Ziemann, P. J.; Atkinson, R. Kinetics, Products, and Mechanisms of Secondary Organic Aerosol Formation. *Chem. Soc. Rev.* **2012**, *41* (19), 6582. https://doi.org/10.1039/c2cs35122f.

(6) Iyer, S.; Kumar, A.; Savolainen, A.; Barua, S.; Daub, C.; Pichelstorfer, L.; Roldin, P.; Garmash, O.; Seal, P.; Kurtén, T.; Rissanen, M. Molecular Rearrangement of Bicyclic Peroxy Radicals Is a Key Route to Aerosol from Aromatics. *Nat. Commun.* **2023**, *14* (1), 4984. https://doi.org/10.1038/s41467-023-40675-2.

(7) Calvert, J. G.; Mellouki, A.; Orlando, J. J.; Pilling, M. J.; Wallington, T. J. *The Mechanisms of Atmospheric Oxidation of the Oxygenates*; Oxford scholarship online; Oxford University Press: New York, 2020. https://doi.org/10.1093/oso/9780199767076.001.0001.

(8) Glowacki, D. R.; Liang, C.-H.; Morley, C.; Pilling, M. J.; Robertson, S. H. MESMER: An Open-Source Master Equation Solver for Multi-Energy Well Reactions. *J. Phys. Chem. A* **2012**, *116* (38), 9545–9560. https://doi.org/10.1021/jp3051033.

(9) Iyer, S.; Rissanen, M. P.; Valiev, R.; Barua, S.; Krechmer, J. E.; Thornton, J.; Ehn, M.; Kurtén, T. Molecular Mechanism for Rapid Autoxidation in α-Pinene Ozonolysis. *Nat. Commun.* **2021**, *12* (1), 878. https://doi.org/10.1038/s41467-021-21172-w.

(10) Kang, S.; Wildt, J.; Pullinen, I.; Vereecken, L.; Wu, C.; Wahner, A.; Zorn, S. R.; Mentel, T. F. Formation of Highly Oxygenated Organic Molecules from *α* -Pinene Photooxidation: Evidence for the Importance of Highly Oxygenated Alkoxy Radicals. *Atmospheric Chem. Phys.* **2025**, *25* (22), 15715–15740. https://doi.org/10.5194/acp-25-15715-2025.

(11) Nannoolal, Y.; Rarey, J.; Ramjugernath, D. Estimation of Pure Component Properties. *Fluid Phase Equilibria* **2008**, *269* (1–2), 117–133. https://doi.org/10.1016/j.fluid.2008.04.020.

(12) Compernolle, S.; Ceulemans, K.; Müller, J.-F. EVAPORATION: A New Vapour Pressure Estimation Methodfor Organic Molecules Including Non-Additivity and Intramolecular Interactions. *Atmospheric Chem. Phys.* **2011**, *11* (18), 9431–9450. https://doi.org/10.5194/acp-11-9431-2011.

(13) Myrdal, P. B.; Yalkowsky, S. H. Estimating Pure Component Vapor Pressures of Complex Organic Molecules. *Ind. Eng. Chem. Res.* **1997**, *36* (6), 2494–2499. https://doi.org/10.1021/ie950242l.

(14) Pankow, J. F.; Asher, W. E. SIMPOL.1: A Simple Group Contribution Method for Predicting Vapor Pressures and Enthalpies of Vaporization of Multifunctional Organic Compounds. *Atmospheric Chem. Phys.* **2008**, *8* (10), 2773–2796. https://doi.org/10.5194/acp-8-2773-2008.

(15) Stolzenburg, D.; Wang, M.; Schervish, M.; Donahue, N. M. Tutorial: Dynamic Organic Growth Modeling with a Volatility Basis Set. *J. Aerosol Sci.* **2022**, *166*, 106063. https://doi.org/10.1016/j.jaerosci.2022.106063.

(16) Mohr, C.; Thornton, J. A.; Heitto, A.; Lopez-Hilfiker, F. D.; Lutz, A.; Riipinen, I.; Hong, J.; Donahue, N. M.; Hallquist, M.; Petäjä, T.; Kulmala, M.; Yli-Juuti, T. Molecular Identification of Organic Vapors Driving Atmospheric Nanoparticle Growth. *Nat. Commun.* **2019**, *10* (1), 4442. https://doi.org/10.1038/s41467-019-12473-2.
(17) Bunker, D. L.; Garrett, B.; Kleindienst, T.; Long, G. S. Discrete Simulation Methods in Combustion Kinetics. *Combust. Flame* **1974**, *23* (3), 373–379. https://doi.org/10.1016/0010-2180(74)90120-5.
(18) Gillespie, D. T. A General Method for Numerically Simulating the Stochastic Time Evolution of Coupled Chemical Reactions. *J. Comput. Phys.* **1976**, *22* (4), 403–434. https://doi.org/10.1016/0021-9991(76)90041-3.
(19) Atkinson, R.; Arey, J. Atmospheric Degradation of Volatile Organic Compounds. *Chem. Rev.* **2003**, *103* (12), 4605–4638. https://doi.org/10.1021/cr0206420.
(20) Manion, J. A.; Huie, R. E.; Levin, R. D.; Burgess Jr, D. R.; Orkin, V. L.; Tsang, W.; McGivern, W. S.; Hudgens, J. W.; Knyazev, V. D.; Atkinson, D. B.; Chai, E.; Tereza, A. M.; Lin, C.-Y.; Allison, T. C.; Mallard, W. G.; Westley, F.; Herron, J. T.; Hampson, R. F.; Frizzell, D. H. NIST Chemical Kinetics Database, NIST Standard Reference Database 17, Version 7.0 (Web Version), Release 1.6.8, Data Version 2015.09, National Institute of Standards and Technology, MD. **2015**.
(21) Atkinson, R.; Baulch, D. L.; Cox, R. A.; Crowley, J. N.; Hampson, R. F.; Hynes, R. G.; Jenkin, M. E.; Rossi, M. J.; Troe, J. Evaluated Kinetic and Photochemical Data for Atmospheric Chemistry: Volume I - Gas Phase Reactions of O_x, HO_x, NO_x and SO_x Species. *Atmospheric Chem. Phys.* **2004**, *4* (6), 1461–1738. https://doi.org/10.5194/acp-4-1461-2004.
(22) Berndt, T.; Scholz, W.; Mentler, B.; Fischer, L.; Herrmann, H.; Kulmala, M.; Hansel, A. Accretion Product Formation from Self- and Cross-Reactions of RO_2 Radicals in the Atmosphere. *Angew. Chem. Int. Ed.* **2018**, *57* (14), 3820–3824. https://doi.org/10.1002/anie.201710989.
(23) Jenkin, M. E.; Valorso, R.; Aumont, B.; Rickard, A. R. Estimation of Rate Coefficients and Branching Ratios for Reactions of Organic Peroxy Radicals for Use in Automated Mechanism Construction. *Atmospheric Chem. Phys.* **2019**, *19* (11), 7691–7717. https://doi.org/10.5194/acp-19-7691-2019.
(24) Boyd, A. A.; Flaud, P.-M.; Daugey, N.; Lesclaux, R. Rate Constants for RO_2 + HO_2 Reactions Measured under a Large Excess of HO_2. *J. Phys. Chem. A* **2003**, *107* (6), 818–821. https://doi.org/10.1021/jp026581r.
(25) Ho, K. F.; Lee, S. C.; Louie, P. K. K.; Zou, S. C. Seasonal Variation of Carbonyl Compound Concentrations in Urban Area of Hong Kong. *Atmos. Environ.* **2002**, *36* (8), 1259–1265. https://doi.org/10.1016/s1352-2310(01)00570-2.
(26) Uebori, M.; Imamura, K. Analysis of Aliphatic and Aromatic Carbonyl Compounds in Ambient Air by LC/MS/MS. *Anal. Sci.* **2004**, *20* (10), 1459–1462. https://doi.org/10.2116/analsci.20.1459.
(27) Yang, B.; Liang, A.; Wang, L. The Atmospheric Oxidation Mechanism of Acetophenone Initiated by the Hydroxyl Radicals. *Atmos. Environ.* **2023**, *309*, 119905. https://doi.org/10.1016/j.atmosenv.2023.119905.
