# Supplementary material for: Nitric oxide can enhance secondary aerosol precursor formation from aromatic carbonyls
Source: Nat Commun. 2026 May 7;17:5454. doi: 10.1038/s41467-026-72628-w (PMC13282404; doi:10.1038/s41467-026-72628-w)
Supplement: Supplementary file 2 — Transparent Peer Review file [file 41467_2026_72628_MOESM2_ESM.pdf]

nature portfolio

Peer Review File

Nitric oxide can enhance secondary aerosol precursor formation from aromatic carbonyls

Corresponding Author: Mr Shawon Barua

This file contains all reviewer reports in order by version, followed by all author rebuttals in order by version.

Attachments originally included by the reviewers as part of their assessment can be found at the end of this file.

Version 0:

Reviewer comments:

Reviewer #1

(Remarks to the Author)
General comments:
This manuscript entitled “Rapid formation of aerosol precursors from the autoxidation of aromatic carbonyls and the remarkable enhancing influence of NO addition” reports a systematic investigation of the autoxidation of three representative aromatic carbonyl compounds (benzaldehyde, phenylacetaldehyde, and acetophenone). Using a flow tube reactor coupled with high-resolution mass spectrometry, the authors demonstrate that these species rapidly form highly oxygenated organic molecules (HOMs) within seconds. Most strikingly, the study reveals that the addition of NO, which is conventionally considered to suppress HOM and SOA formation, instead substantially enhances the production of HOMs, particularly from phenylacetaldehyde and acetophenone. Complementary quantum chemical calculations are employed to rationalize the key role of OH addition and subsequent radical propagation steps in driving the observed chemistry.

The identification of aromatic carbonyls as efficient precursors for HOM formation and the counterintuitive role of NO represent a potentially important shift in our understanding of urban atmospheric oxidation chemistry and SOA formation. However, major revisions that need to be considered before this manuscript is ready for publication:

Specific comments:
1. The current title, “Rapid formation of aerosol precursors from the autoxidation of aromatic carbonyls and the remarkable enhancing influence of NO addition”, is somewhat long and contains multiple pieces of information, which makes it less concise. The core scientific focus—namely the mechanism of aromatic carbonyl autoxidation and the role of NO—is not highlighted clearly. I suggest refining the title to emphasize the key reaction pathway or mechanism and the effect of NO, rather than listing results. A more focused title would better convey the main contribution and attract reader interest.

2. The abstract is clear and well-structured, but certain expressions (e.g., “rapid”, “remarkably”, “significant advancement”) are rather qualitative and could be replaced by more quantitative wording. Furthermore, while the results at high NO are striking, it would be useful to clarify the atmospheric representativeness of the tested NO range and the extent to which the findings can be generalized beyond the three selected aromatic carbonyls.

3. Lines 34-46: Compared with other aromatic VOCs (e.g., toluene, xylene), what is the unique importance of aromatic carbonyls in HOM/SOA formation? Do aromatic carbonyls contribute sufficiently to ambient SOA budgets to warrant focused mechanistic studies? Could the authors provide an order-of-magnitude estimate of their potential SOA contribution compared to other aromatic VOCs?

4. Lines 83-85: How transferable are the mechanistic insights from alkanes or biogenics to aromatic carbonyls? Is it justified to generalize the NOx effect across such chemically distinct systems? Moreover, the discussion currently reads as a sequential listing of individual studies. It would benefit from a logical categorization and integrative analysis of the literature, rather than a simple chronological or numerical listing, to enhance the clarity and coherence of the argument.

5. Lines 116-118: How accurately can the residence times (0.9–2.7 s) be constrained given possible mixing and diffusion

effects in the movable injector configuration?

6. Lines 122-126: What criteria were used to define “quantifiable HOM signals”? Could this selection bias the comparison between different precursors? Could secondary products from TME ozonolysis interfere with the detection or formation of aromatic carbonyl-derived HOMs? Could the authors comment on the potential detection biases of NO3-CIMS, particularly toward highly oxygenated species, and whether this affects quantification of total oxidation products?

7. Lines 126-128: How representative is the chosen NO concentrations (up to 1 ppm) of polluted urban atmospheres? Could the authors justify their experimental range in terms of atmospheric relevance?

8. Lines 135-138: What was the water vapor or D2O mixing ratio in the reactor, and could isotopic substitution affect OH chemistry or secondary reactions? How were potential wall losses, OH and RO2 concentrations, and residence times accounted for in the flow reactor experiments? Could these factors influence the formation yields of HOMs and organonitrates?

9. Lines 138-140: How was the degree of H/D exchange quantified, and could incomplete exchange lead to underestimation of labile H groups?

10. Lines 156-158: What is the estimated uncertainty in the calculated barrier heights and rate coefficients, and how might this affect conclusions on HOM formation timescales? How do the calculated rate coefficients compare with existing experimental or theoretical data for similar aromatic peroxy radical reactions?

11. Lines 175-176: Why was ΔEdown set to 225 cm-1, and was any sensitivity analysis performed with different ΔEdown values (e.g., 100–300 cm-1)?

12. Lines 188-202: What is the justification for averaging results from the four vapor pressure estimation methods (Nannoolal, SIMPOL, EVAPORATION, Myrdal-Yalkowsky)? Since these models have different underlying assumptions and parameterizations, does averaging meaningfully reduce bias, or does it obscure systematic deviations? Some of the methods (e.g., Myrdal-Yalkowsky) are designed for relatively simple organic compounds—how reliable are they when applied to multifunctional HOM species with peroxides and nitrates?

13. Lines 255-257: Could the authors provide detection limits, response factor corrections, and possible biases in quantification, particularly for nitrogen-containing products? How confident are the authors in the structural assignment of the detected HOMs and organonitrates? Were any complementary techniques (e.g., reference standards) used to confirm the molecular structures?

14. In Section 3.2, the effect of NO on product distribution is discussed. Could the authors quantify the relative enhancement of low-volatility products? Is there a potential ionization bias in detecting nitrogen-containing compounds?

15. In Sections 3.3 and 3.4, the mechanistic interpretation of product formation is primarily attributed to RO2 autoxidation pathways. Could the authors clarify whether alternative pathways (e.g., direct OH addition to aromatics) were considered, and how these may contribute to the observed product spectrum?

16. Figure 2: The reported signal intensities on the y-axis are surprisingly low (10-6–10-3 normalized counts s-1). The authors are encouraged to explain how the normalization was carried out, and to discuss whether ionization efficiency differences across compounds may bias the apparent product intensities. Furthermore, the spectra are shown without any indication of measurement uncertainty. Including error bars, replicate spectra, or at least a discussion of reproducibility and detection limits would significantly strengthen the robustness of the conclusions; such information could also be provided in the Supporting Information.

Reviewer #2

(Remarks to the Author)

Reviewer #3

(Remarks to the Author)
Barua et al. 2025 report results from flow tube reactor experiments on three aromatic volatile organic compounds. They measure the yields of highly oxidised molecules from each VOC under varying chemical conditions and report a mechanism by which nitric oxide can enhance this yield. The mechanism has not been well explored before, but could have important implications for the composition of VOC oxidation products, the loading of secondary organic aerosol (SOA) and the composition of SOA.

The conclusions and claims would be significant and novel to the gas-phase chemistry community, however, as detailed below, the current work does not fully support them, and additional evidence is needed. Because of this, I do not recommend publication as is, but advise major revisions.

The derived rate coefficients are very useful findings for improving chemical mechanisms.

In my experience, the authors are correct that suppression of SOA yields from VOC (photo)oxidation by nitrogen oxides is a widely accepted process (e.g. 10.1126/sciadv.ado1482).

Line 77 – I suggest including another reference for the acceptance of NOx suppression, as this reference provides a sort of overview, rather than a focus on specific VOCs: doi.org/10.1126/sciadv.ado1482

Line 77 – is this sentence too specific? I mean, it's not only the suppression of accretion products that reduces SOA yield, it's also the suppression of RO2 autoxidation by formation of organonitrates, i.e. the degree to which autoxidation can proceed. But if my understanding is incorrect, please state in introduction that evidence so far indicates autoxidation itself is not affected by organonitrate formation, rather accretion of autoxidation products is affected.

Line 81 - could this sentence be better connected with the preceding sentence so that the reader knows that the references in the previous sentence detail the alkoxy radical mechanism behind NO enhancement?

Line 83 – for the sentence that references Wang et al., could this be made clearer as to what the correlation between HOM yields from alkanes and NOx is? E.g., as NOx increases does HOM yield increase? At the moment the sentence just references one NOx mixing ratio rather than a trend. Also, could this sentence be extended to discuss the ratios of NOx to RO2, rather than just the absolute mixing ratio of NOx?

Line 85 – suggest a paragraph break starting with the Pullinen et al. sentence so the reader is better able to distinguish between chemical mechanism introduction and aerosol microphysics introduction.

Line 89-94 – starting with 'In the VOC systems where HOM ..' through to the end of the paragraph, I don't understand the points being made. Are you saying that theoretically increased NO:NO2:RO2:HO2 should be able to increased HOM yield, but that consistently observations show the opposite tendency? Whatever the point(s) are, they need to be clearer, and potentially not right after a statement about uptake coefficients, since these coefficients are more about microphysics than they are about gas-phase chemistry.

Line 98 – rather than state mixing ratios of NOx, it is more helpful that the reader is told what the ratios of NO:NO2:RO2:HO2 concentration are, so that they have a better chance to judge whether the investigation is relevant to the chemical regimes of their interest.

Table 1 or Section S1 - temperature, pressure (in main text/tables rather than just captions), relative humidity of experiments and any actinic flux into the flow reactor needs stating. Likewise, a statement about presence or absence of wall effects needs to be included.

Section 2.1 or S1 - I don't see how NO is introduced to the reactor. I don't see whether NO2 or HO2 is present in the reactor. Does nucleation of particles occur in any experiments? How is NO (and NO2) concentration measured? To estimate HOM yield, I expect the VOC concentration at the start and end of the reactor needs measuring, is this true?, if so please show the setup in Fig. S1.

Table 1 - Whilst the provided absolute mixing ratios are helpful, it would be very useful to have a column dedicated to the ratios of gas-phase concentrations of NO:NO2:RO2:HO2.

Table 1 – Please state where in the reactor the provided mixing ratios are applicable, e.g. at the point of injection, at the sampling point, other.

Table 1 – Either in this table (which seems appropriate), or somewhere else, the estimated OH abundance (concentration or mixing ratio) needs stating

Line 205 and associated subsequent lines - To my understanding, the 2D-VBS is a framework for mapping the properties of organic molecules (either individual molecules or groups). But here it referred to as if it's a parametrisation for vapour pressure. Although the parameterisation given in Eq. 2 may be related to 2D-VBS publications, it is a method to estimate vapour pressure, which is fundamentally equivalent to the purpose of the group contribution methods described before it, and so shouldn't be confused with the 2D-VBS. Additionally, on this point, all the methods described for vapour pressure estimation may be applied to the volatility dimension of the 2D-VBS, however as it reads, only eq. 2 is applicable.

Line 219 - is this describing another parameterisation, or alternative values being input to the same parameterisation (eq. 2), please clarify

Section 2.5 – please separate description of the simulations used to estimate reactive species in the conducted experiments more clearly from descriptions of simulations used to to probe the ambient urban atmosphere. For simulations of conducted experiments please specify how the simulation was setup to reproduce experimental conditions. I don't understand what random number seed is. If details are included in Section S11, please make better reference to this. Please tell the reader where they can access the input files for the kinetic simulations.

Section 2.5 and associated supplementary information (S11, by the way is S2.2 within S11 mis-numbered?) – a relatively simple chemical scheme is applied to estimate some very important values, such as concentrations of OH and HO2. However, no verification of the scheme is provided, though the available measurements must allow for this. For example, relative changes to the concentrations of the pool of carbonyl peroxy radicals. HO2 will react with RO2 to form hydroperoxides, but this is not included in the chemical scheme, it's omission needs to be justified explicitly.

Method in general – HOM yield needs to be defined. Also, more needs to be done (probably in section 2.1) to tell the reader how comparable experiments at varying NOx are, even if this only means better reference to supplementary information sections. For example, although ozone and TME mixing ratios are comparable between w/o NOx and w/ NOx experiments, the introduction of NOx will perturb the NO:NO2:RO2:HO2 concentration ratios, as well as the OH concentration, so the reader needs some information about these things to prepare for interpreting the results and conclusion section.

Method in general – the NO:VOC ratio for the investigated VOCs is likely to be greater than 1 throughout the troposphere. However, the experiments here do not exceed 1. Could the authors therefore explain how their experiments are relevant to the troposphere, and particuarly urban areas? Similarly, could the authors explain how the method can provide evidence that even at high NO:VOC concentration ratio, HOM yields are relatively high?

Figure S1 – please include the location of additional sampling instruments, such as the ozone analyser and the instrument for measuring NO and VOC.

Section 3.1 – please do more (e.g. in Fig. captions) to remind the reader that the presented results are for experiments excluding NOx.

Section 3.2 – this results section is key to the significance of the reported finding that HOM yields increase with increasing NO. However, there are several issues:
i) The text suggests that HOM yield is shown in Figs. 3 and 4, however, the ordinate of these plots is a normalised signal. To my understanding, showing a HOM yield would require normalised signal to mean signal divided by consumed VOC, is this the case? If it is, it needs stating that this is what normalised signal means. If it's not, then the main text needs changing to correctly refer to what the plots are showing. Similarly, does enhancement factor in Fig. 4 refer to enhancement of signals normalised by VOC consumption?

Section 3.5 – again I think results need to be normalised by VOC consumption to make a fair comparison. I like that attention is given as to which HOM monomers are changing abundance, e.g. N-containing and non-N-containing. Could the change in abundance of accretion products also be reported?, as the introduction suggests this is important when considering NO influence.

Final paragraph of section 3.5 and related supplementary information – the NO concentration (1 ppb) used for the polluted urban scenario is about a factor of 100 too small, e.g. Fig. 1 of https://doi.org/10.1016/S1352-2310(01)00378-8. By omitting simulation of representative urban conditions, the implications of the study are substantially curtailed.

Generally in results, it is not clear whether the reported enhancement of HOM yield is due only to direct influence of NO on RO2 (e.g. encouraging the alkoxy channel through RO2+NO), or whether it's due to NO addition decreasing hydroperoxide formation through decreasing HO2 (RO2+HO2->ROOH). I think this needs to be explored more to provide better insight into these key mechanisms. For example, by reporting changes to signals of highly oxidised and less highly oxidised closed-shell hydroperoxides (without N) as NO concentration changes.

Discussion generally – could the authors explicitly state what work they think is needed to ensure we correctly capture the chemical behaviour of gas-phase organic radicals of different parent VOC under varying chemical regimes, such that we may accurately represent gas-phase chemistry in atmospheric simulation?

References
Line 35, references 1-3 are quite old and focus on the emissions from transport sources, but they do not seem to verify the claim that 'aromatic carbonyls are major air pollutants'. Could the authors reference some more recent literature that better reports the relative importance of these compounds to air quality?

The manuscript is generally very well written, there are just some minor points that need attention:
Line 36: 'constitute indoor air at significant concentrations' doesn't quite make sense, something more like: 'are present in indoor air at significant concentrations' is preferred

Line 56: 'refer to HOM'?

Simon P. O'Meara

Version 1:

Reviewer comments:

Reviewer #1

(Remarks to the Author)
I thank the authors for their rigorous response to the comments. The additional analysis has substantially improved the manuscript, and I believe that it is now ready for publication.

Reviewer #2

(Remarks to the Author)

Reviewer #3

(Remarks to the Author)
Please see attached review of the revised manuscript.

Version 2:

Reviewer comments:

Reviewer #3

(Remarks to the Author)
The authors make a valuable contribution to the field of research around HOMs generation and the relationship with reactants of organic radicals.

In terms of the ratio of parent VOC reactivity with oxidants to NO concentration, the paper goes notably beyond the [NO] maximum of Wang et al. (2021), appears to go beyond that of Nie et al. (2023) and is comparable to Kang et al. (2025) (though comparisons are somewhat challenging given the changing ratios of reactants, including NO:NO2). In their rebuttal, the authors acknowledge that their minimum ratio may not be representative of highly polluted urban atmospheres, and have correspondingly removed references to highly polluted atmospheres in the main manuscript. However, I do not think the authors have revised the manuscript far enough given that the revised text of the manuscript still conveys a message that as NOx increases into concentrations relevant to polluted atmospheres, HOM yields from two aromatic carbonyls increases. Figure 4, combined with the author's calculations (in rebuttal) on the ambient relevance of NO to RO2_c formation ratio, proves that is clearly not the case. And rather than 'starkly contrasting the traditional understanding of oxidation suppression by NOx', I think the messaging should be that consistent with previous publications, increasing NOx from 0 leads to enhancement followed by suppression (relevant to the peak). If the authors want to contrast the value of [NOx] at which HOM yield peaks with previous studies, I think they will need to compare the RO2 formation rate:NO:NO2 ratio (maybe including other parameters in the ratio that the authors know about) between their study and previous studies.

Perhaps stated more clearly, given that:
i) using 1 ppm of NO in their experiments can give NO to RO2_c formation ratio 2.5 times less than in a polluted atmosphere, and
ii) that the HOM yields peak between 100 and 300 ppb of NO (giving a NO to RO2_c formation ratio 25 times less for 100 ppb NO than in polluted atmospheres (all else being the same)),

I think the authors need to point out that at NO to RO2_c formation ratios that can be relevant to polluted urban atmospheres, i.e. 300 ppb NO and above for their experiments, increasing NO is observed to have a suppressing effect on HOM yield, a trend consistent with previous studies finding a decreasing SOA yield and decreasing HOM accretion product yield at increasing NOx.

Simon P. O'Meara

Open Access This Peer Review File is licensed under a Creative Commons Attribution 4.0 International License, which permits use, sharing, adaptation, distribution and reproduction in any medium or format, as long as you give appropriate credit to the original author(s) and the source, provide a link to the Creative Commons license, and indicate if changes were made.
In cases where reviewers are anonymous, credit should be given to 'Anonymous Referee' and the source.
The images or other third party material in this Peer Review File are included in the article's Creative Commons license, unless indicated otherwise in a credit line to the material. If material is not included in the article's Creative Commons license and your intended use is not permitted by statutory regulation or exceeds the permitted use, you will need to obtain permission directly from the copyright holder.
To view a copy of this license, visit https://creativecommons.org/licenses/by/4.0/

We thank the reviewers for going through our manuscript titled "*Rapid formation of aerosol precursors from the autoxidation of aromatic carbonyls and the remarkable enhancing influence of NO addition*" and for their constructive suggestions on how to improve the work. We have incorporated all the suggestions and have modified the revised manuscript accordingly. Below, the reviewer queries are reproduced in red, followed by detailed point-by-point author responses in blue, and the changes and additions to the revised manuscript and the supplementary information (SI) in purple.

Reviewer #1 (Remarks to the Author):

General comments:

This manuscript entitled "Rapid formation of aerosol precursors from the autoxidation of aromatic carbonyls and the remarkable enhancing influence of NO addition" reports a systematic investigation of the autoxidation of three representative aromatic carbonyl compounds (benzaldehyde, phenylacetaldehyde, and acetophenone). Using a flow tube reactor coupled with high-resolution mass spectrometry, the authors demonstrate that these species rapidly form highly oxygenated organic molecules (HOMs) within seconds. Most strikingly, the study reveals that the addition of NO, which is conventionally considered to suppress HOM and SOA formation, instead substantially enhances the production of HOMs, particularly from phenylacetaldehyde and acetophenone. Complementary quantum chemical calculations are employed to rationalize the key role of OH addition and subsequent radical propagation steps in driving the observed chemistry.

The identification of aromatic carbonyls as efficient precursors for HOM formation and the counterintuitive role of NO represent a potentially important shift in our understanding of urban atmospheric oxidation chemistry and SOA formation. However, major revisions that need to be considered before this manuscript is ready for publication:

Specific comments:

1. The current title, "Rapid formation of aerosol precursors from the autoxidation of aromatic carbonyls and the remarkable enhancing influence of NO addition", is somewhat long and contains multiple pieces of information, which makes it less concise. The core scientific focus—namely the mechanism of aromatic carbonyl autoxidation and the role of NO—is not highlighted clearly. I suggest refining the title to emphasize the key reaction pathway or mechanism and the effect of NO, rather than listing results. A more focused title would better convey the main contribution and attract reader interest.

Response: We agree with the reviewer and have now revised the title to a more concise "*Nitric oxide enhances secondary aerosol precursor formation from aromatic carbonyls*".

Changes to manuscript: Title in line 1, "Nitric oxide enhances secondary aerosol precursor formation from aromatic carbonyls"

2. The abstract is clear and well-structured, but certain expressions (e.g., "rapid", "remarkably", "significant advancement") are rather qualitative and could be replaced by more quantitative

wording. Furthermore, while the results at high NO are striking, it would be useful to clarify the atmospheric representativeness of the tested NO range and the extent to which the findings can be generalized beyond the three selected aromatic carbonyls.

Response: We thank the reviewer for the suggestions. The expressions, "rapid oxidation", "remarkably", "significant advancement" etc. are now removed from the abstract while the quantitative information (e.g., 0.9 s reaction time, enhancement factor of 16.9, etc.) are kept.

The NO concentration range was chosen to cover moderately polluted to highly polluted urban scenarios – and to go clearly beyond any polluted ambient air space, to show how big this effect is! And to an extent get closer to combustion device conditions where even much higher concentrations are encountered. That is, we used high NO concentrations (up to 1 ppm) to examine up to what level NO influences HOM enhancement and at what level its suppression effect takes over in aromatic carbonyl oxidation processes.

Although the suppression effect of NO on HOM formation is well acknowledged, there are limited studies in the literature that show the enhancement effect in discrete VOC systems under various NO concentrations. Therefore, we are yet to be able to generalize the findings into different classes of compounds and hence more studies focused on the effect of varying NO in VOC oxidation are warranted. It is also to be noted that the branching ratios of the RO_2 + NO reaction towards RO and organonitrates as well as the branching ratio of subsequent RO rearrangement reaction are very much dependent on the specific RO_2 structures. Similarly, in the case of the studied aromatic carbonyls, we observed differences in the relative enhancement effect of NO and discussed the likely reasons behind the observation.

Changes to manuscript: In line 30, "This observation represents an improvement in our understanding of polluted air ..." instead of "... represents a significant advancement ..."

Addition to manuscript: In lines 151–153, "The chosen NO range not only covers moderately polluted to highly polluted urban scenarios but also goes beyond any polluted ambient environment allowing us to observe the extent of the effect."

In lines 570–571, "This work also highlights the non-identical behavior of oxidation across similar species (aromatic carbonyls)."

3. Lines 34-46: Compared with other aromatic VOCs (e.g., toluene, xylene), what is the unique importance of aromatic carbonyls in HOM/SOA formation? Do aromatic carbonyls contribute sufficiently to ambient SOA budgets to warrant focused mechanistic studies? Could the authors provide an order-of-magnitude estimate of their potential SOA contribution compared to other aromatic VOCs?

Response: From molecular structure point of view, aromatic carbonyls feature a reactive carbonyl functional group and an aromatic moiety in the same molecule making it unique compared to individual class of carbonyl compounds and aromatics. For making a direct comparison of their potential SOA contribution with that of other aromatics, we consider conducting a follow up study based on chamber experiments under comparable reaction conditions. In this manuscript, we showed for the first time the formation of low-volatility aerosol precursor from three representative aromatic carbonyl oxidation in as low as sub-

second reaction time together with an unexpected oxidation enhancement by a common oxidation terminating compound. However, we agree with the reviewer that the SOA contribution comparison will be next logical step to look forward to soon and will be the scope of a follow-up study.

Addition to manuscript: In lines 37–40, "Aromatic carbonyls feature a reactive carbonyl functional group that can significantly affect the atmospheric oxidation chemistry in comparison to the more abundant alkyl substituted primary emissions (e.g., toluene and xylene).[12]"

4. Lines 83-85: How transferable are the mechanistic insights from alkanes or biogenics to aromatic carbonyls? Is it justified to generalize the NOx effect across such chemically distinct systems? Moreover, the discussion currently reads as a sequential listing of individual studies. It would benefit from a logical categorization and integrative analysis of the literature, rather than a simple chronological or numerical listing, to enhance the clarity and coherence of the argument.

Response: Regardless of the type of VOC, the effect of NO on HOM formation lies on to what extent the RO_2 + NO reaction produces reactive RO and closed-shell organonitrates. The subsequent isomerization reaction of RO radicals, depending on their structures, can often compete with their fragmentation and contribute to propagation of the oxidative radical chain (alkoxy-peroxy pathway). Thus, in all cases, the enhancement of HOM production in the presence of NO is certainly due to the modulation of the RO_2 loss and formation of alkoxy radicals that can continue to autoxidize through isomerization. One latest paper (Kang et al., 2025) is added to the citation (https://doi.org/10.5194/acp-25-15715-2025).

Although the suppression effect of NO on HOM formation is well acknowledged, there are limited studies in the literature that have shown the enhancement effect in discrete VOC systems under non-identical NO concentrations. This is why it is not yet feasible to categorize the type of compounds into different observation patterns even though a logical categorization of literature studies would be more interesting.

Changes to manuscript: In lines 82–87, "However, prior studies[29–32] have shown that in select cases low NO_x concentrations can enhance the formation of HOMs via alkoxy radical (RO) mediated chemistry in alkane and alkenes. While NO can terminate RO_2 autoxidation by forming closed shell organonitrates (ONs), it also forms more reactive RO radicals (RO_2 + NO $\rightarrow$ RO + NO_2) that undergo fast isomerization followed by O_2 addition and regenerate RO_2. The new RO_2 with one more O atom than the preceding RO_2 can further undergo autoxidation and enhance HOM formation.[33]"

5. Lines 116-118: How accurately can the residence times (0.9–2.7 s) be constrained given possible mixing and diffusion effects in the movable injector configuration?

Response: Considering an uncertainty in injector position by 2–3 mm in the flow reactor of 4.7 cm inside diameter with an 8.1 slpm total flow, we can estimate around 0.03 s uncertainty in the residence time (RT) calculation. Additional uncertainty with the mass flow controller (MFC) flows leads to a total 0.05 s RT uncertainty. While the other reactants are premixed at

the beginning of the flow reactor, only the VOC of interest is brought inside reactor at different positions using the movable injector. We maintain a laminar flow in the reactor and the VOC flow with the injector is mixed at the center of gas stream. This way we can ensure an immediate mixing of the VOC by diffusion down the stream. It is also important to note that the mass spectrometer pinhole samples the gas mixture from the middle of the stream.

Changes to manuscript: In lines 127–129, "We conducted variable residence time experiments including short (0.9–2.7±0.05 s) and long (14±0.05 s) residence times by controlling the reaction time between the precursor and the oxidant."

In lines 135–137, "… and residence times of 1.1±0.05 s, 0.9±0.05 s, and 2.7±0.05 s were maintained for the oxidation of benzaldehyde, phenylacetaldehyde, and acetophenone, respectively …"

6. Lines 122-126: What criteria were used to define "quantifiable HOM signals"? Could this selection bias the comparison between different precursors? Could secondary products from TME ozonolysis interfere with the detection or formation of aromatic carbonyl-derived HOMs? Could the authors comment on the potential detection biases of NO3-CIMS, particularly toward highly oxygenated species, and whether this affects quantification of total oxidation products?

Response: We thank the reviewer for pointing out this and replace "quantifiable HOM" by "any HOMs" in the revised manuscript. The formation of HOMs in short reaction time experiments depends on how fast the first alkyl peroxy radicals (RO_2) form as well as how fast the subsequent isomerization reactions are. For example, within 1–2 s rection time acetophenone did not produce any HOMs regardless of how high the precursor concentrations were.

The ozonolysis of TME produces two byproducts, acetone (C_3H_6O) and $C_3H_5O_3$ alkyl peroxy radical, both with 3 carbon atoms along with the formation of OH radical (see Figure S2 below). The studied aromatic carbonyls (C_xH_yO) have 7 and 8 carbon atoms – C_7H_6O: benzaldehyde; C_8H_8O: phenylacetaldehyde, acetophenone. The carbonyl derived oxidation products $C_xH_{y-(y+2)}O_z$ and $C_{x-1}H_yO_z$ are distinct from any possible TME derived products by composition. During the oxidation process, TME-derived $C_3H_5O_3$ peroxy radicals can react with carbonyl-derived RO_2 radicals (e.g, $C_xH_{y+1}O_z$) and possibly form corresponding accretion products (e.g., $C_{x+3}H_{(y+1)+5}O_z$; we did not see any of them) which are also distinct from purely carbonyl-derived accretion products. Thus, TME ozonolysis does not interfere with the detection of HOMs rather it offers some level of complexation in the reaction mixture despite which we observed that the aromatic carbonyls efficiently form HOMs.

Generally, with the increase of number of oxygen atoms in the oxidation products, the binding enthalpy with NO_3^- is increased, so as the detection sensitivity. However, there are no chemical standards for HOMs. This is because the polyperoxide HOMs are likely high explosives in the condensed phase and no one can currently synthesize them. Therefore, the calibration factor for H_2SO_4 is commonly used for quantifying HOMs considering that HOMs are detected with on an average as similar sensitivity as H_2SO_4 in NO_3^--CIMS method (also see the response to Comment 16).

Changes to manuscript: In line 138, "any HOM" instead of "quantifiable HOM"

Addition to SI: In lines 32–38, "At the beginning of the flow reactor, the in situ production of oxidant OH radical was carried out by the ozonolysis reaction of tetramethylethylene (TME) as shown in Figure S2. The reaction produces acetone (CH_3COCH_3) and $C_3H_5O_3$ peroxy radical as the biproducts with OH radical. Although the biproducts adds some level of complexity to the reaction mixture in the flow reactor, they did not seem to complicate the detection of highly oxygenated organic molecules (HOMs) produced in the oxidation of aromatic carbonyls because of their distinct chemical compositions."

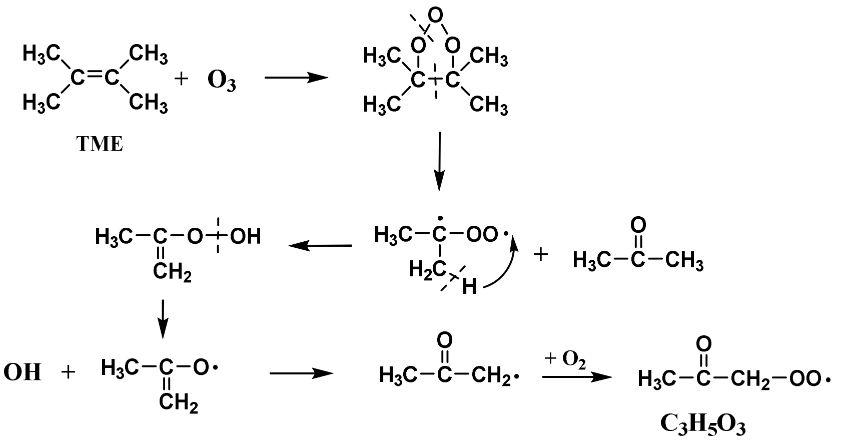

Figure S2. Formation of OH radical along with acetone (CH_3COCH_3) and $C_3H_5O_3$ peroxy radical in the reaction between TME and ozone (O_3)."

In lines 65–70, "The HOMs were quantified based on the assumption that they are charged as efficiently as sulfuric acid. The concentrations of the oxidation products including HOMs were calculated by multiplying the individual normalized product signals with the calibration coefficient. The same calibration coefficient was commonly used for all oxidation products because of lack of methods that can account for differences in sensitivity across various oxygenated products."

7. Lines 126-128: How representative is the chosen NO concentrations (up to 1 ppm) of polluted urban atmospheres? Could the authors justify their experimental range in terms of atmospheric relevance?

Response: As explained in the response to Comment 2, the NO concentration range was chosen to cover moderately polluted to highly polluted urban scenarios. Aromatic carbonyls are commonly present in urban emissions especially related to industrial processes and combustion. We used high NO concentrations (up to 1 ppm) to examine up to what level of NO it influences HOM enhancement and at what level its suppression effect takes over in aromatic carbonyl oxidation process.

Changes to manuscript: In lines 151–153, "The chosen NO range not only covers moderately polluted to highly polluted urban scenarios but also goes beyond any polluted ambient environment allowing us to observe the extent of the effect."

8. Lines 135-138: What was the water vapor or D2O mixing ratio in the reactor, and could isotopic substitution affect OH chemistry or secondary reactions? How were potential wall losses, OH and RO2 concentrations, and residence times accounted for in the flow reactor experiments? Could these factors influence the formation yields of HOMs and organonitrates?

Response: During H/D exchange experiments, we provided as much D_2O flow as needed to fully convert the reagent ions signals $HNO_3NO_3^-$ and $(HNO_3)_2NO_3^-$ to $DNO_3NO_3^-$ and $(DNO_3)_2NO_3^-$, respectively. This has been previously found as a good indicator of the completeness of replacing H_2O with D_2O in the gas flow (note that we meticulously dried the carrier air for these experiments before adding the D_2O to the gas flow). The concentration was in the range of 1670–3350 ppm of D_2O. This method has been commonly used to track the number of labile H atoms in the different VOC oxidation processes. A close comparison of mass spectra obtained from experiments without and with D_2O reveals that the addition of D_2O did not change the distribution of oxidation products except the shifts of product signals equivalent to the number of OH, OOH, and (or) C(O)OOH groups present in them (Figure S8 in the SI).

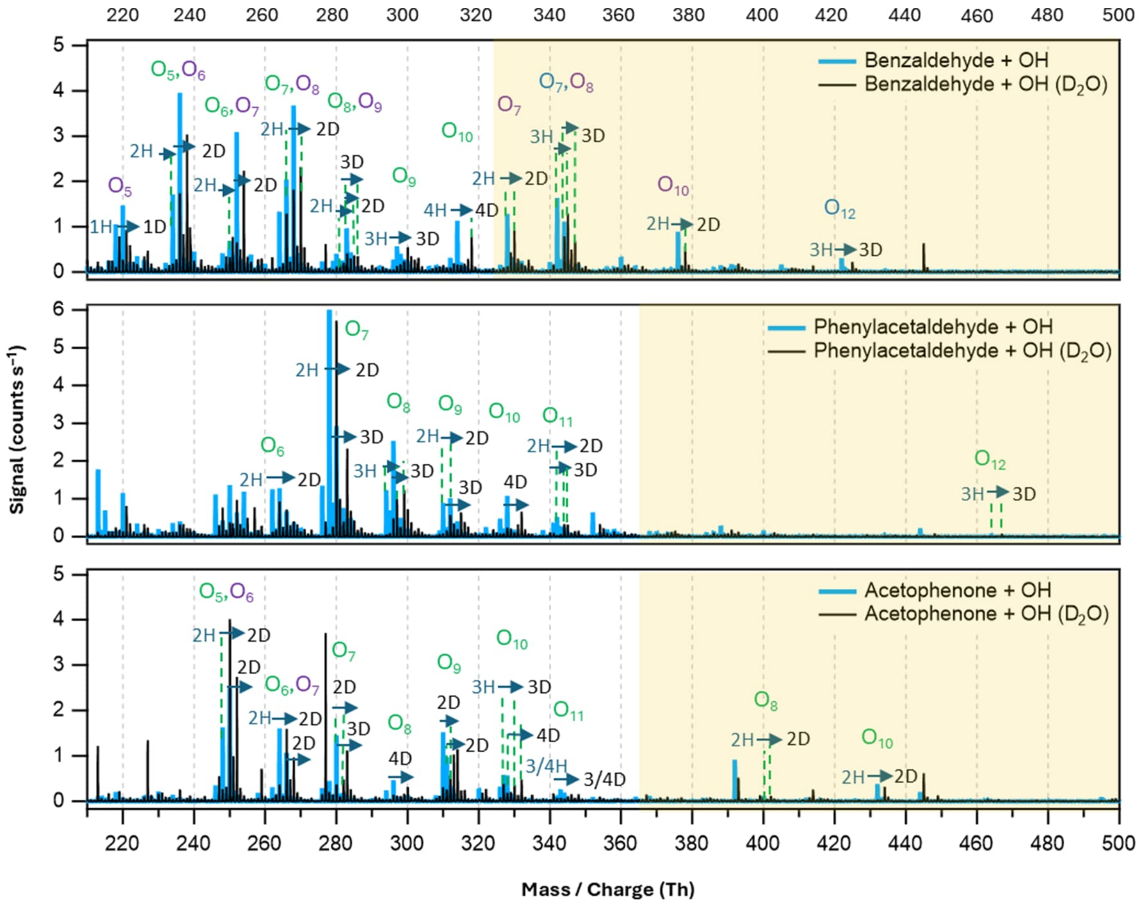

Figure S8. Overlaid nitrate chemical ionization mass spectra of OH initiated oxidation of aromatic carbonyls without (in blue) and with the presence of D_2O (in black). The peak mass shifts during D_2O addition illustrate the number of exchangeable H-atoms present in the product molecular structures. The accretion products are highlighted in light brown background. The labels in green indicate non-fragmented (C_x and C_{2x}) products with different number of oxygen atoms while the labels in purple indicate fragmented (C_{x-1} and $C_{2(x-1)}$)

products. The blue labels (O_7 and O_{12}) in the accretion product region of benzaldehyde spectrum (top panel) indicate products with 13 C atoms (i.e., C_7-RO_2 + C_6-RO_2).

Wall loss of reactive and condensable species is almost unavoidable phenomena in flow reactors and environmental chambers. The "almost" comes from the fact that a few recent setups are able to perform the full reaction in the center-stream of a gas flow in timescales that don't permit walls to interact with the gas flow. Our experiments were performed in conventional flow reactors with wall interactions present.

While it is easy to know wall interactions are present as we are sampling both reactive and condensable material, it is far from trivial to estimate their influence. This is true especially for the type of gas mixture we are studying that has a high variety of reactivities and abilities to condense, as is natural for a rapidly formed wide oxidation distribution that is produced by a chain-like chemistry mediated by reactive radical intermediates (i.e., peroxy, alkoxy, and alkyl). Now, the wall-loss-behavior between these species can be very different but there is no way to investigate it with the current experimental methods. Including such an analysis would only unnecessarily increase the uncertainty, and that we do not want to do.

Measuring all reactions in the practically identical setup reduces the uncertainty. Additionally, the short reaction times also somewhat limit the influence of the wall loss and our inlet technology has been developed to sample reactive species with minimal amount of wall interactions to allow sampling direct aerosol precursors.

The concentrations of OH and RO_2 radicals under different reaction conditions were estimated using a kinetic simulation model and details are provided in Section S15 in the Supporting Information.

While discussing yield estimation, these influences are now acknowledged in the revised SI.

Additon to SI: In lines 70–75, "Then, the yields of the products were estimated by dividing their concentrations with the concentration of consumed precursor VOC (i.e., Y = $\varDelta$HOM/$\varDelta$VOC). Also, note that the flow reactor system exhibits unavoidable wall loss of reactive and condensable species, which contributes to the overall uncertainty in the experimental yields. The normal uncertainty range with the measurement technique has been assumed as asymmetric -50% to +100% (about a factor of two)."

9. Lines 138-140: How was the degree of H/D exchange quantified, and could incomplete exchange lead to underestimation of labile H groups?

Response: Based on the peak areas of $HNO_3NO_3^-$ and $DNO_3NO_3^-$ (black peaks in Figure S7 below) after the introduction of D_2O in the flow reactor, we calculated about 98–99 % H/D conversion in different aromatic carbonyl oxidation experiments. In the case of reagent dimer anion, about 97–98 % of $(HNO_3)_2NO_3^-$ converted to $(DNO_3)_2NO_3^-$ (in black) while 1–2 % remained as $HNO_3DNO_3NO_3^-$. A similar conversion level is always reflected in the detected oxygenated reaction products, and hence it is unlikely we are underestimating the number of labile H groups present.

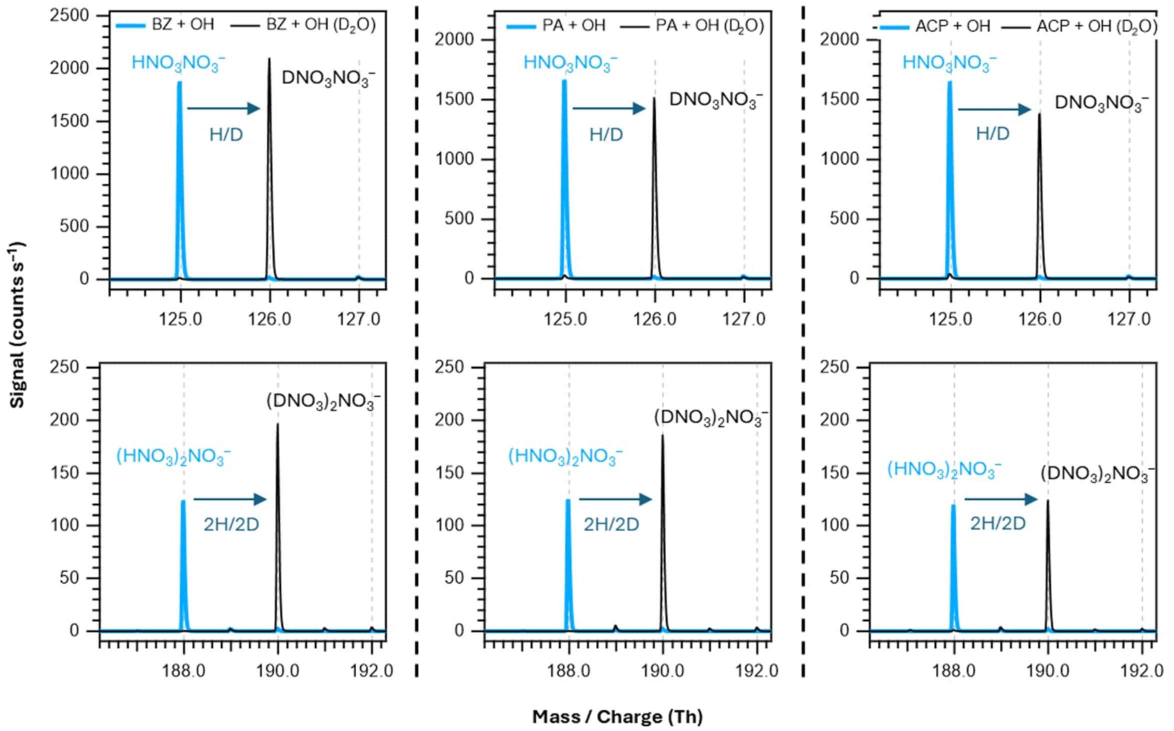

Figure S7. H/D exchange in the reagent ion $HNO_3NO_3^-$ converting it to $DNO_3NO_3^-$ during different aromatic carbonyl OH oxidation reaction in presence of D_2O. BZ = benzaldehyde, PA= phenylacetaldehyde, and ACP = acetophenone.

Changes to manuscript: In lines 156–159, "A near complete H/D conversion (98–99%) was achieved which is monitored by the signals of $HNO_3NO_3^-$ and $(HNO_3)_2NO_3^-$ fully converting to $DNO_3NO_3^-$ and $(DNO_3)_2NO_3^-$, respectively (see Figure S7 in the Supplementary Information)."

10. Lines 156-158: What is the estimated uncertainty in the calculated barrier heights and rate coefficients, and how might this affect conclusions on HOM formation timescales? How do the calculated rate coefficients compare with existing experimental or theoretical data for similar aromatic peroxy radical reactions?

Response: The uncertainties in the calculated barrier heights and the rate coefficients at the employed quantum chemical level of theory is around 1 kcal/mol and a factor of 5, respectively. These do not change our conclusions on HOM formation timescales in our experiments. There is no experimental data but some limited theoretical data on molecular rearrangement rate coefficients. These are sensitive to the aromatic precursor and the site of the initial OH addition during oxidation and consequently cannot be generalized. The molecular rearrangement rate of phenylacetaldehyde derived ipso-BPR calculated here is close to 1 s^{-1}, similar to that of toluene calculated previously (Iyer et al., 2023). It is significantly slower for benzaldehyde and acetophenone, comparable to benzene and ortho-xylene calculated previously (Iyer et al., 2023). The other rate-coefficients calculated in this work are 1,6 H-shifts from the α(OH) carbon by ring-opened peroxy radicals from phenylacetaldehyde and acetophenone. These are in the range of 0.6–0.9 s^{-1}, which is faster than the predicted rate coefficient in Vereecken et

al. (2020) for this reaction (0.18 s^{-1}). This is likely due to the allylic nature of the abstracted H-atom in our case which tends to make these H-abstractions faster.

Iyer et al. (2023), https://doi.org/10.1038/s41467-023-40675-2

Vereecken et al. (2020), https://doi.org/10.5194/acp-20-7429-2020

Changes to manuscript: In lines 450–459, "For $PhCH_2CHO$, the BPR molecular rearrangement rate coefficient of 0.6 s^{-1} is similar to that of toluene reported by Iyer et al.[16] It is significantly slower for PhCHO and $PhCOCH_3$, comparable to benzene and ortho-xylene reported by the same authors. The first 1,6 H-shift in the ring open structure of C2 channel (RB-C2) leads to a resonance-stabilized alkyl radical, $C_xH_{y+1}O_6$ that can add an O_2 and undergo autoxidation to form O_{12}-HOMs as shown. The rate coefficients of the 1,6 H-shift reaction immediately after the BPR ring opening are 0.9 and 0.6 s^{-1} respectively, for $PhCH_2CHO$ and $PhCOCH_3$. These are faster than the predicted rate coefficient by Vereecken et al.[57] for this reaction (0.18 s^{-1}). This is likely due to the allylic nature of the abstracted H-atom in our case which tends to make these H-abstractions faster."

11. Lines 175-176: Why was ΔEdown set to 225 cm-1, and was any sensitivity analysis performed with different ΔEdown values (e.g., 100–300 cm-1)?

Response: The ΔE_{down} value of 225 cm^{-1} is within the range recommended by MESMER for N_2 bath gas and has been used previously in similar simulations (Iyer et al., 2021; Barua et al., 2023). We did not perform a sensitivity analysis but agree that this would be a valuable exercise. We have now run multiple simulations for ΔE_{down} values ranging from 100 to 300 cm^{-1} and they did not change the 1,6 H-shift reaction rate coefficients of the ring broken RB-C2 alkyl peroxy intermediates in $PhCH_2CHO$ and $PhCOCH_3$ oxidation (see Figure S6 below).

Iyer et al. (2021), https://doi.org/10.1038/s41467-021-21172-w

Barua et al. (2023), https://doi.org/10.5194/acp-23-10517-2023

Addition to SI:

S9. Sensitivity analysis with ΔE_{down} in MESMER

In lines 200–209, "In this work, the master equation solver for multi-energy well reactions (MESMER) program[7] was employed to account for the excess energy in peroxy radicals formed in i-BPR molecular rearrangement reaction when estimating the subsequent 1,6 H-shift rate coefficients for $PhCH_2CHO$ and $PhCOCH_3$. Details of simulation parameters are given in Section 2.3 in the main manuscript. We used a ΔE_{down} value of 225 cm^{-1} for simulating collision energy transfer which is within the range recommended by MESMER for N_2 bath gas and has been used previously in similar simulations.[8,9] In addition, we ran multiple simulations for ΔE_{down} values ranging from 100 to 300 cm^{-1} and they did not change the 1,6 H-shift reaction rate coefficients of the ring broken RB-C2 alkyl peroxy intermediates in $PhCH_2CHO$ and $PhCOCH_3$ oxidation (Figure S6)."

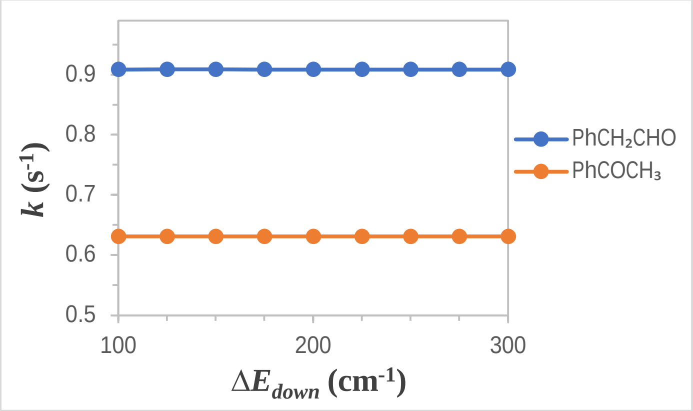

Figure S6. Isomerization (1,6 H-shift) reaction rate coefficients of i-BPR-MR derived ring broken peroxy radicals (RB-C2) as a function of ΔE_{down} values in phenylacetaldehyde ($PhCH_2CHO$ in blue) and acetophenone ($PhCOCH_3$ in orange) oxidation.

12. Lines 188-202: What is the justification for averaging results from the four vapor pressure estimation methods (Nannoolal, SIMPOL, EVAPORATION, Myrdal-Yalkowsky)? Since these models have different underlying assumptions and parameterizations, does averaging meaningfully reduce bias, or does it obscure systematic deviations? Some of the methods (e.g., Myrdal-Yalkowsky) are designed for relatively simple organic compounds—how reliable are they when applied to multifunctional HOM species with peroxides and nitrates?

Response: We thank the reviewer for this insightful comment and are fully aware that the applied methods have different assumptions and parameterizations. However, the averaging of vapor-pressure results from the four methods was simply intended to provide a consensus indicator among their different estimations. It has been previously stated that because of the different compounds used to derive the parameterizations, a combination of them is likely to perform better (Kurtén et al., 2016; Isacman-VanWertz and Aumont, 2021). When several established methods yield different values, taking their average offers a straightforward way to summarize the central tendency of the predictions. This approach does not imply a more accurate or reliable estimation of vapor pressure; rather, it serves as additional information to help readers understand the overall range and mean behavior of the selected models (see Figure S24 in the SI).

Kurtén et al. (2016), https://doi.org/10.1021/acs.jpca.6b02196

Isacman-VanWertz and Aumont (2021), https://doi.org/10.5194/acp-21-6541-2021

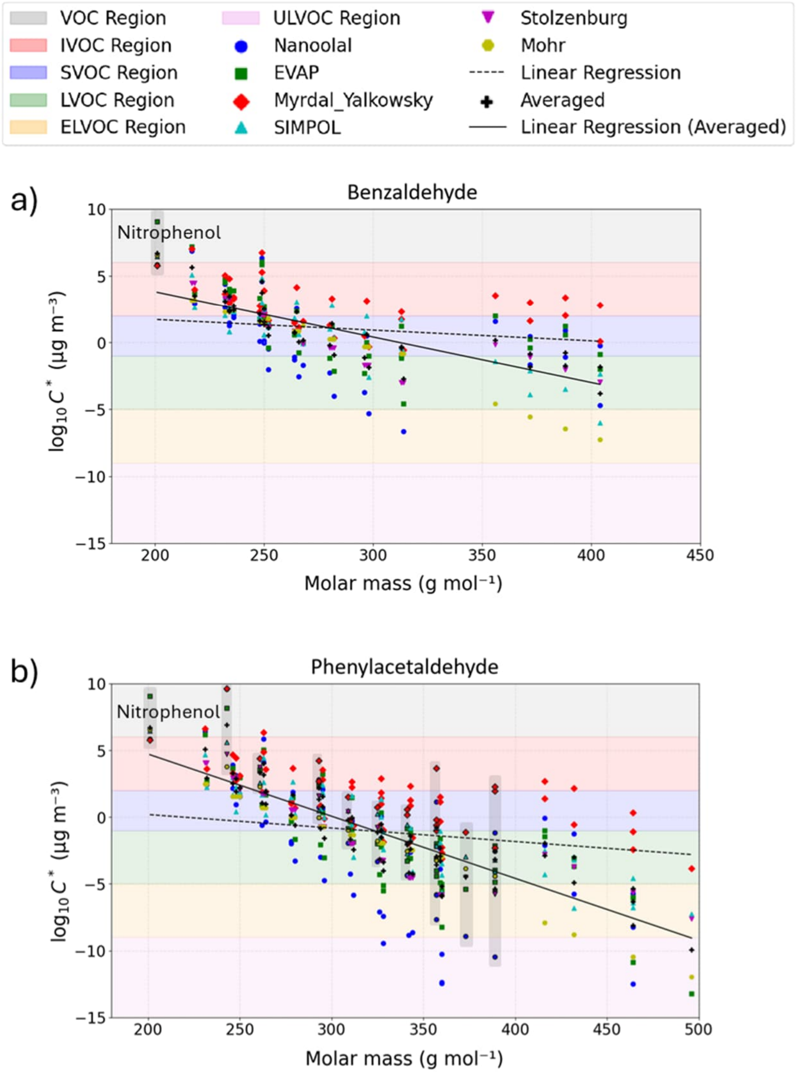

Figure S24. The relationship between molar mass and $\log_{10}C^*$ of the detected oxidation products estimated using six vapor pressure prediction models. Different markers represent individual models, while nitrogen-containing compounds (mostly organonitrates except the nitrophenol) are highlighted with black borders within grey columns.

13. Lines 255-257: Could the authors provide detection limits, response factor corrections, and possible biases in quantification, particularly for nitrogen-containing products? How confident are the authors in the structural assignment of the detected HOMs and organonitrates? Were any complementary techniques (e.g., reference standards) used to confirm the molecular structures?

Response: Using the calibration curve method, we determined a detection limit of 2.82×10^3 molecules/cm^3 for sulfuric acid. HOMs are quantified based on the assumption that they are charged as efficiently as sulfuric acid (also see the response to Comment 16). The calibration coefficient for our NO_3^--CIMS is determined to be 2.0×10^9 molecule/cm^3/ncps.

The specific structures of the identified products depend on their formation mechanisms. In this study, we discuss the most probable formation mechanisms of oxidation products supported by quantum chemical calculations. On top of it, the assigned structures were also

verified by H/D exchange experiments in terms of estimating the number of H-bond donating groups available in them. In general, autoxidation that propagates by complex RO_2 and RO isomerization reactions are likely to have many competing reactions available, almost guaranteeing the existence of several isomeric products species. Here, it is imperative to notice that this competition often occurs on the second and third isomerization reactions which are already too complex to exhaust every possible reaction with quantum chemical computations - whereas often the initiating steps can favor only one path over the others. Although there might be unknown mechanisms that can contribute to other structural isomers of the products, they are expected to have equivalent number of OH, OOH, and (or) C(O)OOH groups but with different spatial orientations. This does not affect the current volatility estimation (discussed in Section S14 in the SI).

Furthermore, there are no chemical standards for HOMs. This is because the polyperoxide HOMs are likely high explosives in the condensed phase and no one can currently synthesize them. Therefore, the calibration factor for H_2SO_4 is commonly used for quantifying HOMs considering that HOMs are detected with on an average as similar sensitivity as H_2SO_4 in NO_3^--CIMS method.

Additon to SI: In lines 59–71, "We determined the calibration coefficient for our NO_3^--CIMS to be 2.0×10^9 molecule/cm^3/ncps by calibrating the instrument for sulfuric acid using the method shown by Kürten et al.[2] A detection limit (LOD) of 2.82×10^3 molecules/cm^3 for sulfuric acid was determined using the equation below.

$$LOD = \frac{3.3 \times \sigma}{S}$$

Here, S is the slope of the calibration curve and σ is the standard deviation of the responses of blank measurements. The HOMs were quantified based on the assumption that they are charged as efficiently as sulfuric acid. The concentrations of the oxidation products including HOMs were calculated by multiplying the individual normalized product signals with the calibration coefficient. The same calibration coefficient was commonly used for all oxidation products because of lack of methods that can account for differences in sensitivity across various oxygenated products. Then, the yields of the products were estimated by dividing their concentrations with the concentration of consumed precursor VOC (i.e., $Y = \Delta HOM/\Delta VOC$)."

14. In Section 3.2, the effect of NO on product distribution is discussed. Could the authors quantify the relative enhancement of low-volatility products? Is there a potential ionization bias in detecting nitrogen-containing compounds?

Response: In Section 3.2, we discussed the effect of NO on different oxygenated products. The relative enhancement of low-volatility products was discussed in Section 3.5 (*volatility estimation and atmospheric implication*) and shown in Figure 6.

In this work, nitrate chemical ionization mass spectrometer (NO_3^--CIMS) was used to detect the oxidation products. Nitrate ionization is selective to higher oxygenated products. Generally, oxidation products with two and more H-bond donating groups can strongly bind with NO_3^-. The identified products listed in Table 2 include products with as low as 4 oxygen atoms. So, the presence of an N atoms will not affect the detection of the products (i.e., organonitrates in

this case) as the addition of NO to RO_2 does not alter the number of H-bond donating groups. Although there is some indication that nitro and nitrate groups similarly increase the binding of the nitrate ion, the results are yet uncertain. Such an example is nitrophenol ($C_6H_4OHNO_2$) that has an anomalously high sensitivity to NO_3^--CIMS (Cheng et al., 2021; Hyttinen et al., 2018) and as a relatively simple primary oxidation product it has a high concentration in comparison with any single HOM compounds sourced by complex multistep chemistry with several branching points in between. Thus, it showed the highest intensity among the oxidation products in all the studied aromatic carbonyl oxidation spectra (Figure 3). However, nitrophenol belongs to volatile organic compounds and its accurate quantification is not as important as the low volatility products studied in this work.

The potential detection bias the reviewer is likely pointing here relates to observing nitrogen containing species as dual peaks in the spectrum as several nitrogenated compounds are charged by the reagent ion dimer as well. This feature was not observed with other species than nitrophenol which was observed at 201 and 264 Th nominal masses.

Cheng et al. (2021), https://doi.org/10.1021/acs.est.0c07988

Hyttinen et al. (2018), https://doi.org/10.1021/acs.jpca.7b10015

Changes to manuscript: In lines 541–546, "We consider that the sensitivity of the oxidation products is equal because NO_3^- ionization is selective to HOMs and because of lack of methods that can account for differences in sensitivities across various oxygenated products. The exception is nitrophenol ($C_6H_4OHNO_2$) that has an anomalously high sensitivity to NO_3^--CIMS[62,63] and thus it showed the highest intensity signal among all the oxidation products in the studied aromatic carbonyl oxidation."

15. In Sections 3.3 and 3.4, the mechanistic interpretation of product formation is primarily attributed to RO2 autoxidation pathways. Could the authors clarify whether alternative pathways (e.g., direct OH addition to aromatics) were considered, and how these may contribute to the observed product spectrum?

Response: Indeed, direct OH addition to the aromatic moiety was shown to be the key contributor to the observed oxidation products (Section 3.1 Lines 302–304, "*… products with a maximum of two more H atoms in their compositions ($C_xH_{y+2}O_z$) compared to their parent precursors (C_xH_yO) which suggests that the oxidation initiated by the addition of OH radical to the aromatic moiety is significant.*").

While Figure 5 in Section 3.3 highlights the *ipso*-BPR (initial OH addition to the *ipso* position) and subsequent autoxidation mechanism, the formation of *ipso*-BPR was shown in the SI and was mentioned in the main manuscript (Section 3.3 Lines 434–436, "*The OH can either add to a carbon with a double bond or abstract an H atom, both forming C-centered radicals. The reaction schemes are provided in Sections S5 and S6 in the Supporting Information.*").

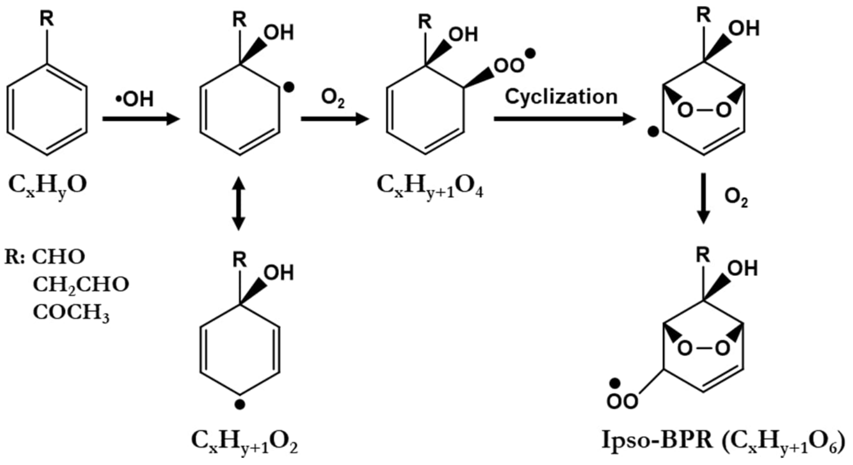

Figure S3. An example of the formation of a bicyclic peroxy radical (BPR, $C_xH_{y+1}O_6$) in OH initiated oxidation of aromatic carbonyls showing the initial addition of OH to the carbon with the substituent group (R: CHO/CH_2CHO/$COCH_3$), i.e., ipso position.

16. Figure 2: The reported signal intensities on the y-axis are surprisingly low (10-6–10-3 normalized counts s-1). The authors are encouraged to explain how the normalization was carried out, and to discuss whether ionization efficiency differences across compounds may bias the apparent product intensities. Furthermore, the spectra are shown without any indication of measurement uncertainty. Including error bars, replicate spectra, or at least a discussion of reproducibility and detection limits would significantly strengthen the robustness of the conclusions; such information could also be provided in the Supporting Information.

Response: We thank the reviewer for pointing out the need of a mention about how normalization of signal intensities was done. We normalized the signal intensities (on the y-axis) by the sum of the reagent ion signals ($[NO_3^-] + [HNO_3NO_3^-] + [(HNO_3)_2NO_3^-]$) which is a common procedure to overcome any difference in reagent ion stability enabling a fair comparison among different spectra. This is the reason the final numbers on the y-axis are very low (10^{-6}–10^{-3}). To clarify, a common calibration coefficient for NO_3^--CIMS is around 10^{10} molecule / cm^3 / ncps and leads to the very impressive detection limit of around 10^4 molecule / cm^3, which is quantified by measuring photochemically produced sulfuric acid (Kurtën et al., 2012). The general quantification of HOM compounds (i.e., compounds with at least 6 O-atoms) in the literature relies on the assumption that they are charged as efficiently as sulfuric acid. This is the state-of-the-science procedure though one can easily see its flaws while appreciating the shifting sensitivity of CIMS. Thus, we feel it is more accurate to provide the normalized counts that are not converted to apparent concentrations. The normal uncertainty range with the measurement technique has been assumed as asymmetric -50% to +100% (about a factor of two).

Kurtën et al. (2012), https://doi.org/10.1021/jp212123n

In terms of reproducibility, the results – formation of HOMs and distribution of oxidation products – are reproducible. Examples of aromatic carbonyl oxidation performed on different dates (and times) are shown in Figure S9 (see below). However, every experiment is in practice different and reproducing exactly the same experimental conditions in two separate

experiments, which require matching several gas flows from unique gas feeds through dedicated mass flow control systems, is almost impossible. This is reflected in the measurements as small fluctuations in the observed product ion intensities. In Figure S9 a–b below, we can see the presence of some background signals at around m/z 373 and m/z 401 in the top panel (black) which are negligible in the bottom panel (blue). Therefore, it is unusual to make an average of several spectra. Instead, it is common practice to present individual mass spectra with mass range of interest normalized by the reagent ion signals for comparisons without further manipulating the raw data.

We have now included the process of normalization, detection limit calculation, and yield calculation in Section S1 in the Supplementary Information. We also included a discussion of reproducibility in Section S11 in the SI.

Addition to SI: In lines 53–75, "The signal intensities of all the detected species were normalized using the following expression:

$$S = \frac{[X * NO_3^-]}{[NO_3^-] + [HNO_3NO_3^-] + [(HNO_3)_2NO_3^-]}$$

where $[X * NO_3^-]$ represents the intensity of an individual species X as NO_3^- adduct. In the denominator, $[NO_3^-]$, $[HNO_3NO_3^-]$, and $[(HNO_3)_2NO_3^-]$ represent the intensities of reagent ion monomer, dimer, and trimer, respectively.

We determined the calibration coefficient for our NO_3^--CIMS to be 2.0×10^9 molecule/cm^3/ncps by calibrating the instrument for sulfuric acid using the method shown by Kürten et al.[2] A detection limit (LOD) of 2.82×10^3 molecules/cm^3 for sulfuric acid was determined using the equation below.

$$LOD = \frac{3.3 \times \sigma}{S}$$

Here, S is the slope of the calibration curve and σ is the standard deviation of the responses of blank measurements. The HOMs were quantified based on the assumption that they are charged as efficiently as sulfuric acid. The concentrations of the oxidation products including HOMs were calculated by multiplying the individual normalized product signals with the calibration coefficient. The same calibration coefficient was commonly used for all oxidation products because of lack of methods that can account for differences in sensitivity across various oxygenated products. Then, the yields of the products were estimated by dividing their concentrations with the concentration of consumed precursor VOC (i.e., Y = ΔHOM/ΔVOC). Also, note that the flow reactor system exhibits unavoidable wall loss of reactive and condensable species, which contributes to the overall uncertainty in the experimental yields. The normal uncertainty range with the measurement technique has been assumed as asymmetric -50% to +100% (about a factor of two)."

S11. Experimental reproducibility

The results we obtained from different aromatic carbonyl oxidation experiments – formation of HOMs and distribution of oxidation products – are reproducible. Here, we show a few representative mass spectra of aromatic carbonyl oxidation experiments performed on different

dates (and times) in Figure S9 below: phenylacetaldehyde (PA, panels a–b in black and grey), benzaldehyde (BZ, panels c–d in red and light red), and acetophenone (ACP, panels e–f in blue and light blue). However, every experiment is practically different and reproducing the experimental conditions of two separate experiments exactly the same, which require matching several gas flows from unique gas feeds through dedicated mass flow control systems, is almost impossible. In the case of PA, we can see the presence of some background signals at around mass/charge (m/z) 373 and 401 in Figure S9a (18.01.2024) which are negligible in Figure S9b performed on 01.02.2024 using same precursor conditions. In BZ (panels c–d of Figure S9) and ACP (panels e–f of Figure S9) experiment pairs, we had little differences in precursor BZ and ozone (O_3) concentrations, respectively, as their initial conditions while other reactants remained same. This was reflected in product signal intensities with somewhat higher signals in 1.3 ppm BZ condition compared to 1 ppm BZ as expected (Figure S9c and d). A similar observation is seen in the case of ACP under 263 ppb versus 312 ppb O_3 conditions (Figure S9e and f). Besides, we see different background signals m/z 226 and m/z 392 in panels e and f, respectively, of Figure S9. On the other hand, we could reproduce the same number and distribution of oxidation products in individual pairs of PA, BZ, and ACP experiments. Therefore, while we can reproduce the oxidation products of a precursor VOC in separate experiments, it is unusual to make an average of several spectra recorded at different times. Instead, it is common practice to present individual mass spectra normalized by the reagent ion signals without further manipulating the raw data for a fair comparison of results under different conditions (including different VOCs) as shown in Figures 1–3 in the main manuscript.

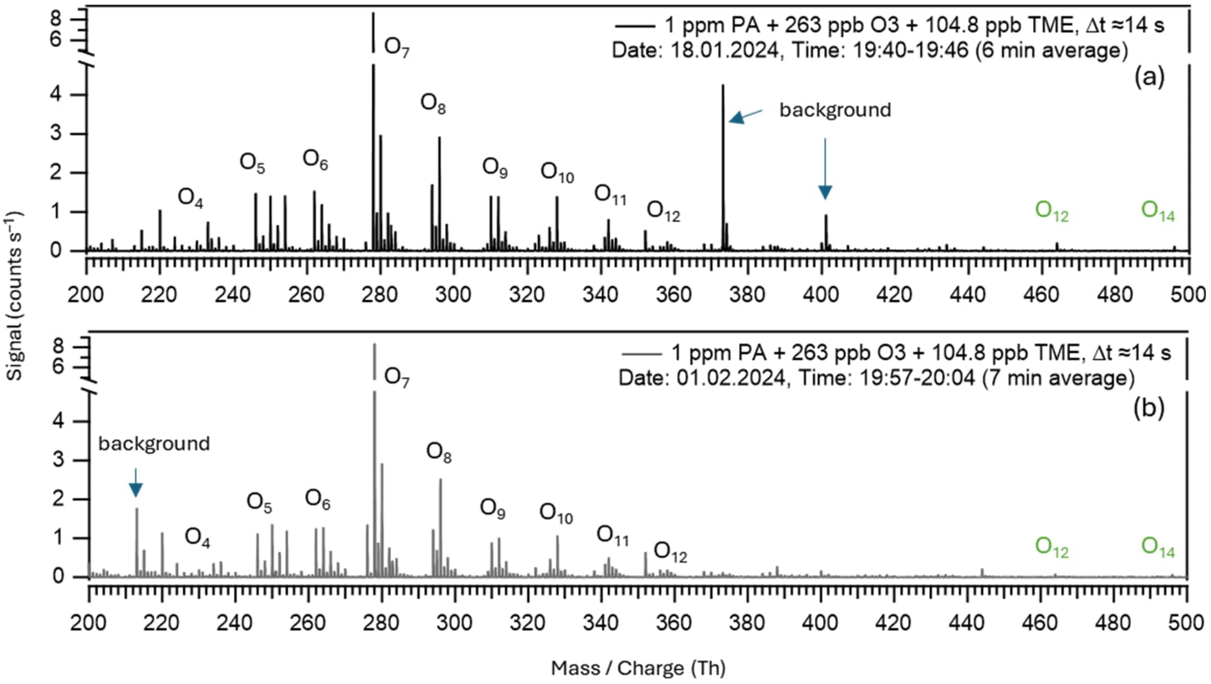

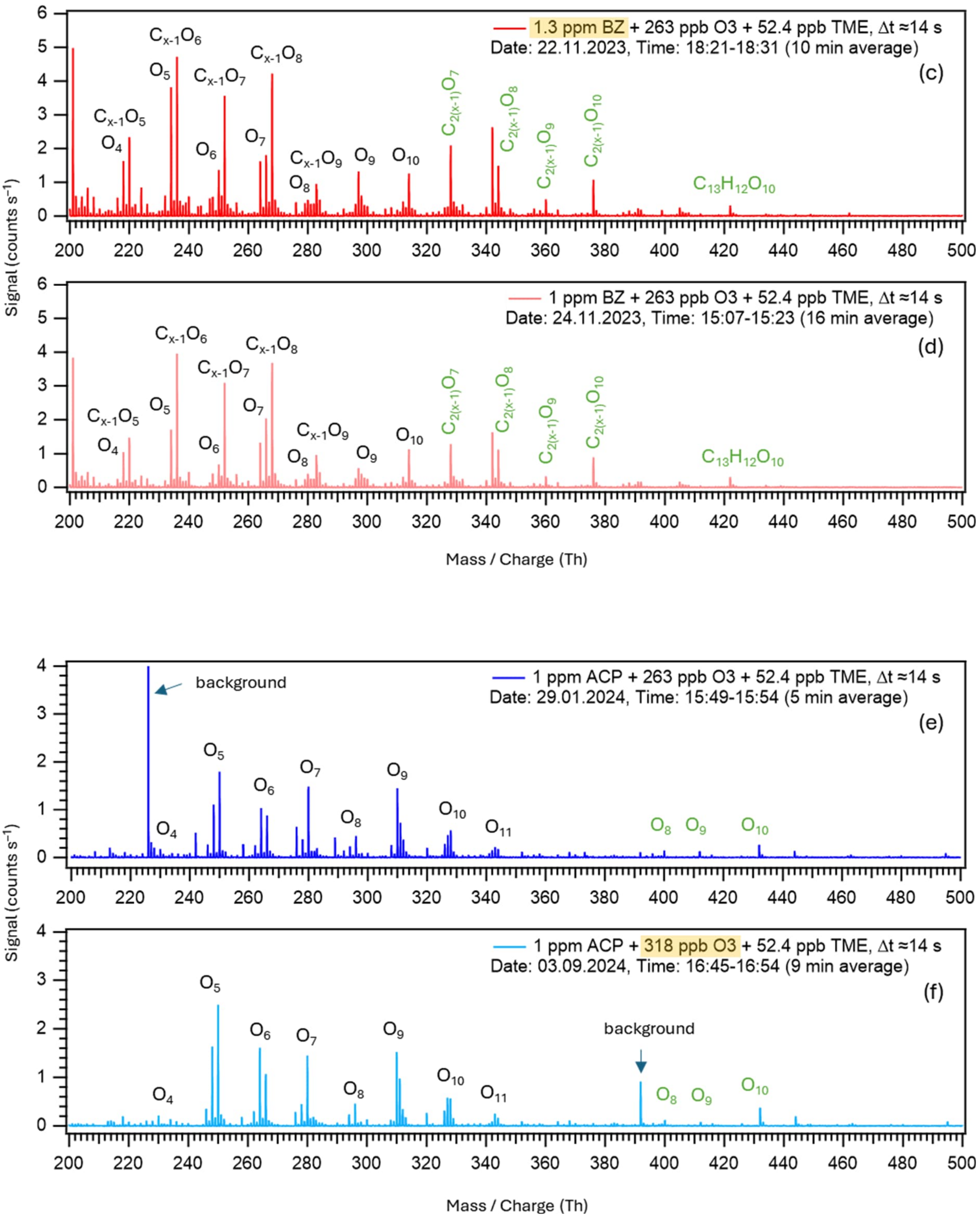

Figure S9. Aromatic carbonyl oxidation spectra recorded in different times showing the reproducibility of the oxidation products: phenylacetaldehyde (PA, panels a–b in black and grey), benzaldehyde (BZ, panels c–d in red and light red), and acetophenone (ACP, panels e–f in blue and light blue). The labels on top of the oxidation product signals (O_{4-12}) indicate the extent of oxygenation in them. Monomeric product signals are labeled in black and that of accretion products are labeled in green. In the case of BZ (panels c–d), the labels starting with C_{x-1} and $C_{2(x-1)}$ indicate the products with loss of one carbon atom compared to precursor BZ and corresponding accretion products, respectively.

Reviewer #2

The manuscript entitled *"Rapid formation of aerosol precursors from the autooxidation of aromatic carbonyls and the remarkable enhancing influence of NO addition"* investigates HOM formation in the OH-initiated oxidation of three aromatic carbonyls through systematic flow-tube experiments with varying reaction times and NO injection. The study also connects the experimental results with quantum chemical calculations. The authors demonstrate that NO injection significantly enhances HOM formation through the alkoxy pathway. The experiments are well designed, and the data analysis and manuscript are clearly presented and organized. However, I still have some concerns. In particular, I find that certain quantitative comparisons are missing, which I think is crucial for you major conclusion. I recommend the authors address these issues before the paper can be considered for publication. My specific comments and concerns are outlined below.

Major concern: (authors should address it seriously).

The paper discusses HOM yields, which depend not only on the HOM detected by CIMS but also on precursor consumption. By definition, yield = Δ(HOM) / Δ(precursor). The figures present normalized HOM signals, which I assume are normalized to reagent ions. However, without accounting for precursor consumption, these normalized signals cannot be directly compared across different systems when discussing yields. I suggest authors address yields comparison by considering the consumption of precursors.

Response: We thank the reviewer for pointing out the need for showing yields (Y = Δ HOM/Δ VOC) instead of normalized signals when discussing HOM yields in the manuscript. Yes, the mass spectra (Figures 1–3) present all oxidation product signals including HOMs normalized by the reagent ion signals ($[NO_3^-] + [HNO_3NO_3^-] + [(HNO_3)_2NO_3^-]$). We discussed HOM yields in Section 3.2 (Enhancement of HOM yields in presence of NO) and showed a trend with varying NO concentrations in Figure 4. *In the revised manuscript in Figure 4, we now modified the numbers in y-axes by percentage yields (considering precursor consumption).* However, the mass spectra (Figures 1–3) are still presented as normalized signal intensities which is very common to show as a continuous mass axis covering the mass range of interest. This preserves the originality of the raw mass spectra while the normalization process helps to overcome any difference in reagent ion stability enabling a fair comparison among different spectra measured under comparable initial conditions. Accordingly, we now carefully replaced the word "yields" by "signal intensities" in the relevant places.

Changes to manuscript:

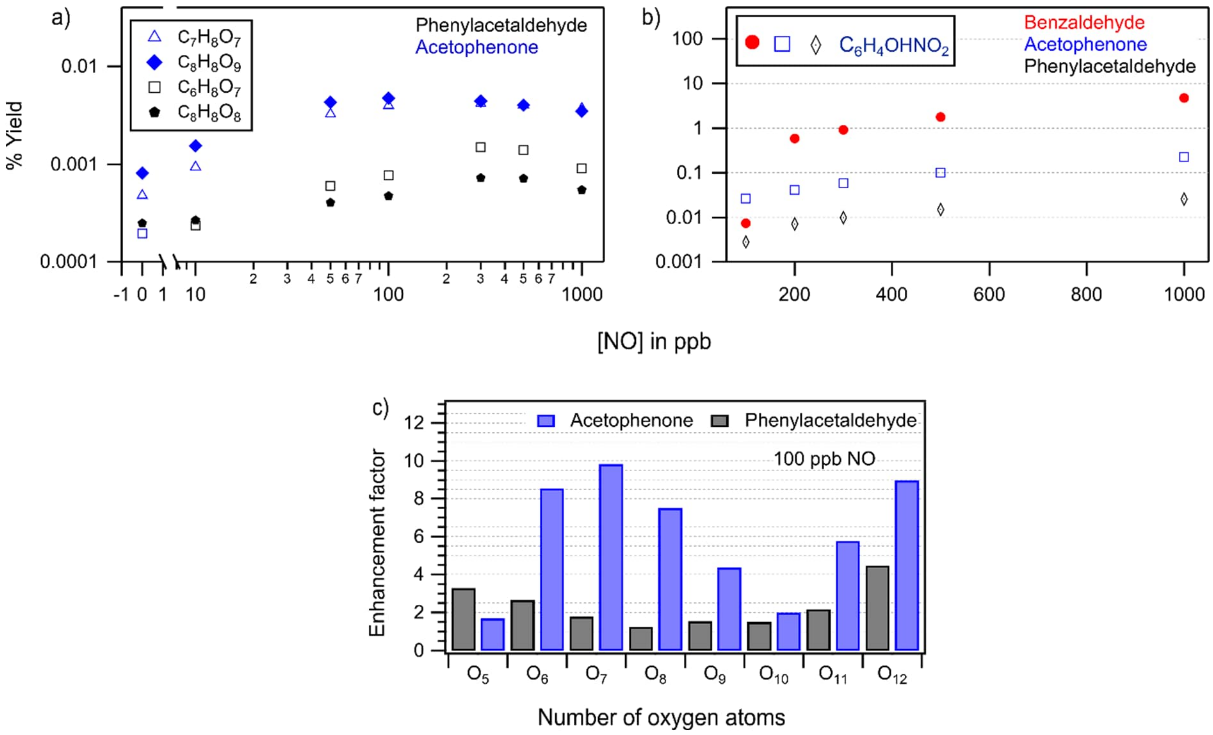

Figure 4. Enhancement of HOM yields in presence of NO during the OH initiated oxidation of phenylacetaldehyde (black) and acetophenone (blue). (a) HOM yields detected by NO_3^--CIMS as a function of NO concentration shown in a logarithmic scale. Several representative HOMs including both non-fragmented C_8 and fragmented $C_{6–7}$ HOMs are shown in filled and unfilled markers, respectively. (b) The yield of nitrophenol ($C_6H_4OHNO_2$) as a function of NO concentration in all the aromatic carbonyl systems studied including benzaldehyde in red. Note the logarithmic scale in y-axis. (c) Total yield enhancement of oxygenated products grouped by different oxygen numbers in presence of 100 ppb NO. Reaction time, Δt = 14 s.

Other comments

1. Lines 50–52: I recommend citing a reference here when discussing the fate of RO_2 radicals. How does the OH radical influence the fate of RO_2?

Response: We thank the reviewer for pointing out the need for a reference there. In the revised manuscript, we have added Jenkin et al. (2019) as a reference which discusses about the reaction of RO_2 with different reaction partners including OH radical.

The reaction between RO_2 and OH can produce RO, ROH, or ROOOH depending on the type of RO_2.

Jenkin et al. (2019), https://doi.org/10.5194/acp-19-7691-2019

2. Lines 56–57: Please also cite a paper for the definition of HOM (e.g., Federico Bianchi, 2019, Chemical Reviews).

Response: The paper, Bianchi et al. (2019) is now cited for HOM definition in the revised manuscript.

3. Line 73: Since NOx is already mentioned in line 51, I suggest moving the description of NOx up to line 51 for clarity.

Response: We respectfully disagree with this proposition and think it is more logical to place NO_x description in lines 75–95. To clarify, the first mention of NO_x came in line 51 when introducing the fate of RO_2 radicals in relation to reactions with other RO_2 radicals, HO_x, and NO_x. Then, we sequentially discussed RO_2 autoxidation leading to HOMs, how fast HOMs can form in different systems, followed by NO_x effect on HOM formation and SOA yields, in the subsequent paragraphs. Because we discussed how NO_x can suppress HOM formation, we think it might be logical to bring the HOM formation process related information beforehand. The current information flow: RO_2 > HOMs > NO_x effect.

In the sentence in question, we now reordered the list of RO_2 reaction partners placing NO_x at the end.

Changes to manuscript: In lines 52–54, "… the fate of the RO_2 radicals depends on their reactivity with a handful of reaction partners including other RO_2, HO_x (OH and HO_2), and NO_x.[14]" instead of "… NO_x, HO_x (OH and HO_2), and other RO_2."

4. Lines 82–83: Please revise the description of the fate of alkoxy radicals. RO radicals first undergo isomerization with rapid rates, followed by O_2 addition to form RO_2, which can then undergo further autooxidation (see: Formation of highly oxygenated organic molecules from α-pinene photooxidation: evidence for the importance of highly oxygenated alkoxy radicals, ACP 2025).

Response: We thank the reviewer for emphasizing the breaking down of the fate of RO radicals and subsequent autoxidation. In the revised manuscript, the description is modified accordingly. The paper mentioned in the comment - Kang et al. (2025) - is also added as a citation.

Changes to manuscript: In lines 85–87, "… it can also form more reactive RO radicals ($RO_2 + NO \rightarrow RO + NO_2$) that undergo fast isomerization followed by O_2 addition and regenerate RO_2. The new RO_2 with one more O atom than the preceding RO_2 can further undergo autoxidation and enhance HOM formation.[33]"

5. Experimental setup:
   - Please provide more details on your CIMS setup, as these data are central to your study: manufacturer, LToF or HToF, mass resolution, and inlet type.
   - How were VOC and NOx concentrations measured? Please include this in the main text.
   - In the H/D section, it is unclear how water influences the chemistry and how H is substituted by D. I suggest adding water-related chemical reactions in the manuscript or SI.

Response: We thank the reviewer for suggestions on providing more information related to experimental setup in the manuscript.

- More details on CIMS setup are provided in Section S1 in the SI including the information below.
  Manufacturer: Tofwerk AG/Aerodyne Research Inc., mass analyzer: HToF, mass resolution: 3500 m/Δm, inlet type: Eisele inlet.

- The reactants were introduced into the flow tube reactor in controlled flow using calibrated mass flow controllers (Alicat Scientific Inc.). A premixed VOC cylinder with an estimated mixing ratio in high-purity nitrogen (5.0 grade) was prepared in-house. The NO cylinder, containing 100 ppm of NO in nitrogen, was obtained commercially from Air Products.
  The initial concentrations of VOC and NO in the gas stream were calculated by using equation:

$$[X] = \frac{measured\ flow_x}{measured\ flow_t} \times [MR]$$

  where species X can be either VOC or NO, $measured\ flow_x$ is the measured flow of the species X introduced in the gas stream, $measured\ flow_t$ is the measured total flow of the gas stream, and MR is the mixing ratio of the species X in the gas cylinder.

- In the presence of excess D_2O, we could exchange the labile H atoms available with the oxidation products by D atoms. An example of such reactions is given below.

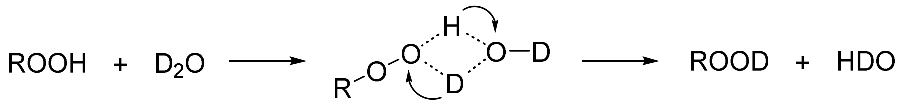

A close comparison of mass spectra obtained from experiments without and with D_2O reveals that the addition of D_2O did not change the distribution of oxidation products except the shifts of product signals equivalent to the number of OH, OOH, and (or) C(O)OOH groups present in them (Figure S8 in the SI). Furthermore, NO_3^- ionization is almost completely insensitive to relative humidity changes.

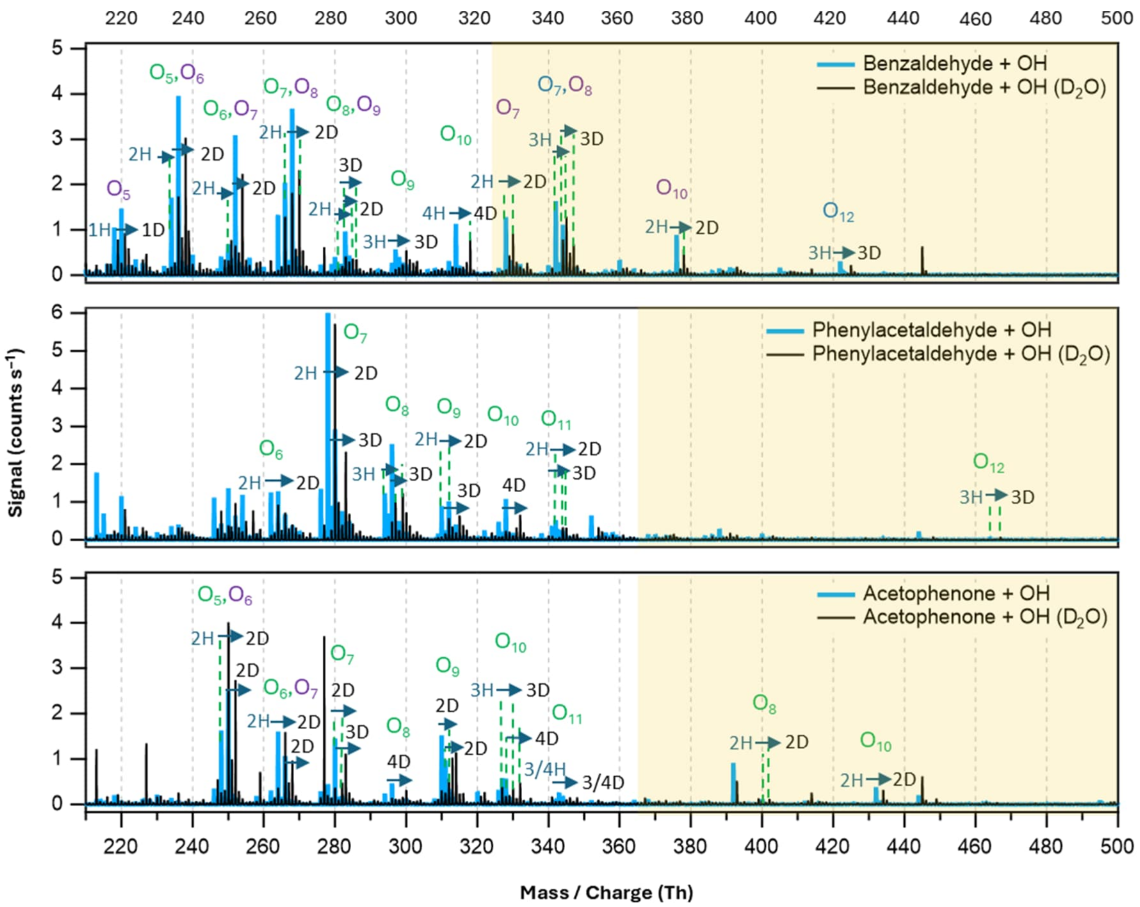

Figure S8. Overlaid nitrate chemical ionization mass spectra of OH initiated oxidation of aromatic carbonyls without (in blue) and with the presence of D_2O (in black). The peak mass shifts during D_2O addition illustrate the number of exchangeable H-atoms present in the product molecular structures. The accretion products are highlighted in light brown background. The labels in green indicate non-fragmented (C_x and C_{2x}) products with different number of oxygen atoms while the labels in purple indicate fragmented (C_{x-1} and $C_{2(x-1)}$) products. The blue labels (O_7 and O_{12}) in the accretion product region of benzaldehyde spectrum (top panel) indicate products with 13 C atoms (i.e., C_7-RO_2 + C_6-RO_2).

Changes to manuscript: In lines 123–127, "The initial concentrations of reactant precursors in the flow reactor were determined by controlling the individual gas flows using calibrated mass flow controllers (Alicat Scientific). The details including the chemicals and gas cylinders are given in Section S1–S2 in the Supplementary Information."

Addition to SI: In lines 10–14, "A nitrate-based chemical ionization mass spectrometer (NO_3^- -CIMS) with an Eisele type[1] atmospheric pressure inlet was coupled to the flow reactor. The mass spectrometer was manufactured by Tofwerk AG/Aerodyne Research Inc. and was equipped with a high-resolution time-of-flight mass analyzer (HToF, mass resolution: 3500 m/Δm)."

In lines 44–51, "The initial concentrations of VOC and NO in the gas stream were calculated by using equation:

$$[X] = \frac{measured\ flow_x}{measured\ flow_t} \times [MR]$$

where species X can be either VOC or NO, $measured\ flow_x$ is the measured flow of the species X introduced in the gas stream, $measured\ flow_t$ is the measured total flow of the gas stream, and MR is the mixing ratio of the species X in the gas cylinder. On the other hand, the time profiles of VOC, NO, NO_2, etc. were estimated from kinetic simulation model discussed in Section S15 below. The average concentrations of OH, and primary RO_2 radicals under different reaction conditions are given in Table S8."

In lines 215–216, "In the presence of excess D_2O, we could exchange the labile H atoms available with the oxidation products by D atoms. An example of such reactions is given below."

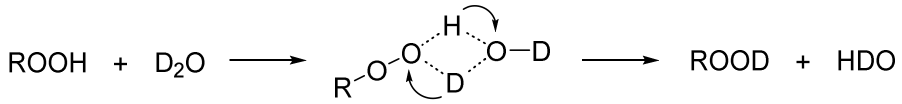

In lines 231–235, "A close comparison of mass spectra obtained from experiments without and with D_2O reveals that the addition of D_2O did not change the distribution of oxidation products except the shifts of product signals equivalent to the number of OH, OOH, and (or) C(O)OOH groups present in them (see Figure S8). Furthermore, NO_3^- ionization is almost completely insensitive to relative humidity changes."

6. Lines 139–140: When D_2O was used, did the reagent ion $HNO_3{\cdot}NO_3^-$ become $DNO_3{\cdot}NO_3^-$? If so, does this imply that HNO_3 in the $HNO_3{\cdot}NO_3^-$ cluster originates from chemical formation in the flow tube rather than nitric acid vapor from the ion source?

Response: The reagent ions $HNO_3NO_3^-$ and $(HNO_3)_2NO_3^-$ are converted to their deuterated form $DNO_3NO_3^-$ and $(DNO_3)_2NO_3^-$, respectively, in the presence of D_2O. However, HNO_3 does not form in the flow-reactor but is present in the sheath air of the Eisele inlet (Sipilä et al., 2015). Monitoring the mass shifts of the reagent ions during H/D exchange experiment is routine process to check the efficiency of the conversion.

Please note that all H/D exchange experiments were conducted without the presence of NO and hence we did not produce HNO_3 in the flow reactor. Also, the ion-molecule reaction (IMR) time in the Eisele inlet (~113 millisecond) used in this work is significant enough for the D_2O + $HNO_3NO_3^-$ (and $(HNO_3)_2NO_3^-$) reaction to exchange labile H atoms with D atoms. The reagent ion conversion is seen to work practically perfectly even with an alternative MION inlet with ~22 millisecond of IMR time.

Sipilä et al. (2015), https://doi.org/10.5194/amt-8-4001-2015

7. Figure 2: This figure combines fragments, non-fragments, and accretion products, making it visually busy and difficult to extract the key message. I suggest grouping compounds into families to provide clearer, zoomed-in insights (e.g., separate C_6HxOx, $C_7H_{8–10}Ox$, C_{14}, C_{13}, and C_{12} families).

Response: We agree with the reviewer that there are many identified product signals labeled in Figure 2 spectra. While splitting each spectrum into different groups of compounds improves clarity, bringing all of them (many spectra from three VOC systems) at one place for comparison is a challenge. We now multiplied the smaller accretion product signals by (5–7 times) for better clarity in Figure 2 and rearranged their labels in Figure 2a in the revised manuscript. Please also note that the labels of fragmented and non-fragmented product signals are color coded and mentioned in the figure caption.

Changes to manuscript:

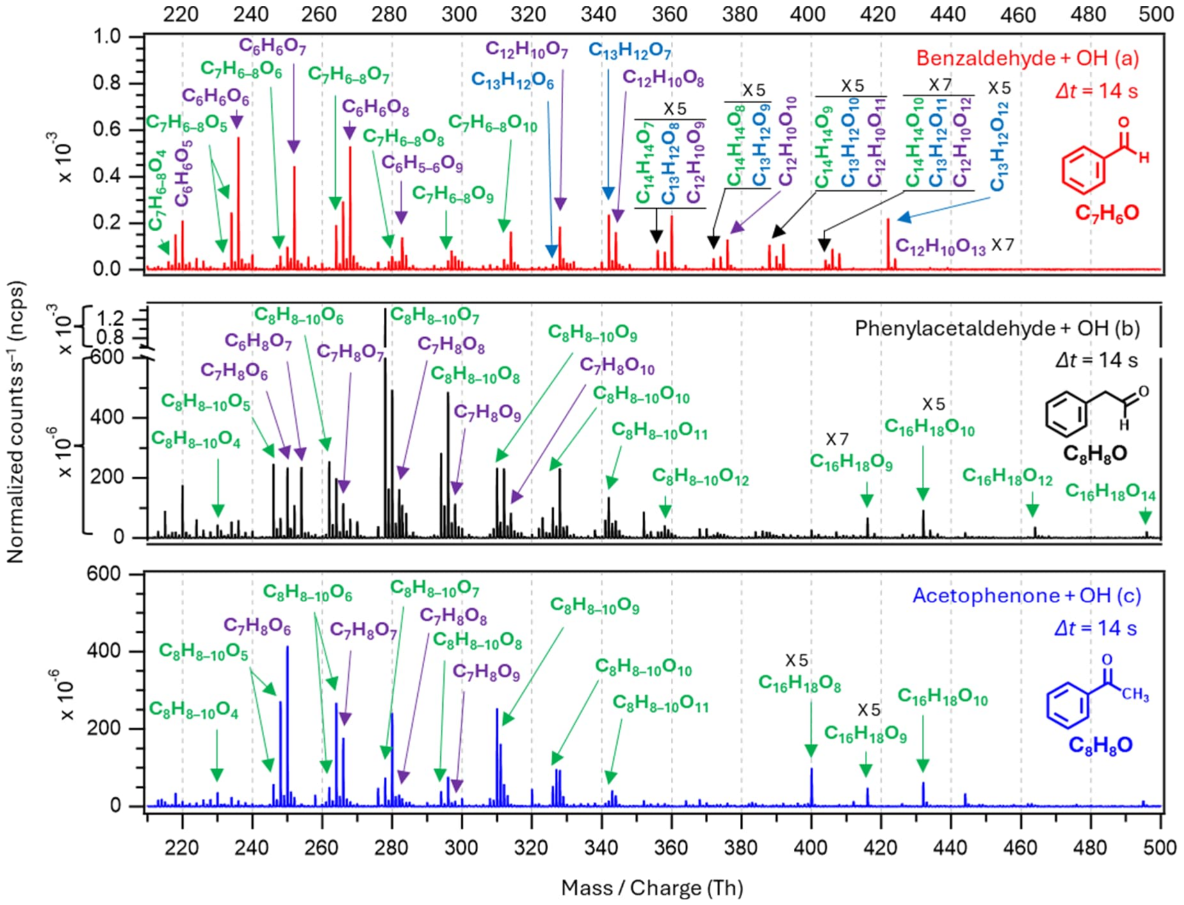

Figure 2. Nitrate chemical ionization mass spectra of OH initiated oxidation of aromatic carbonyls (without NO) in 14 s residence time: benzaldehyde in red (a), phenylacetaldehyde in black (b), and acetophenone in blue (c). In the product peak labels, the NO_3^- is excluded from their corresponding adduct compositions. Non-fragmented products (C_x and C_{2x}) are labelled in green while fragmented products ($C_{x-1,2}$ and $C_{2(x-1)}$) are labelled in purple. The black arrows in panel (a) with multiple compositions (labelled in green, blue, and purple) represent product peaks separated by two mass units starting from the arrow-pointing positions. Several accretion product signals are multiplied by 5–7 times for visibility.

8. Lines 298–299: The appearance of closure products with odd oxygen numbers does not necessarily prove the involvement of alkoxy steps. For example, RO_2 + RO_2 reactions between even-O species can also yield odd-O products (e.g., $C_8H_9O_8$ + $C_8H_9O_8$ → $C_8H_8O_7$

+ $C_8H_{10}O_7$ + O_2). You should either show that RO_2 + RO_2 is negligible relative to RO_2 + HO_2, or explicitly discuss the oxygen number in the RO_2 species.

Response: We completely agree with the reviewer on this point. The odd O-containing closed-shell products can be produced from the odd O-containing RO_2 via H loss and reaction with HO_2. They can also be produced from even O-containing RO_2 + RO_2 reactions (Russell mechanism) producing an alcohol and a carbonyl compound (line 306–307 in the main manuscript). Both processes play an important role in producing odd-even oxygen distribution.

Changes to manuscript: In lines 322–323, "… have a well-defined odd-even oxygen distribution in the identified product peaks including RO_2 radicals"

Addition to manuscript: In lines 327–329, "However, one should also remember that the autoxidation produced even number of oxygen-containing RO_2 can also contribute to the formation of odd-O closed shell products via the Russell mechanism."

9. Lines 328–330: Could you compare the relative roles of bimolecular versus unimolecular reactions across systems? For instance, in sub-second experiments, are RO_2 concentrations comparable? Table 1 shows differing precursor levels (1, 1.5, 3 ppm for benzaldehyde, phenylacetaldehyde, and acetophenone). In Figure S21a, RO_2 in the PA_OH system appears at least two orders of magnitude lower than in ACP_OH, suggesting that RO_2 + RO_2 reactions might be more important in ACP_OH. Could this explain the limited HOM formation in ACP_OH short-time experiments (Figure 1c)? Also, note the inconsistency between RO_2 concentrations in Table S8 and Figure S21a. In Table S8, the three short-time experiments show comparable RO_2 levels.

Response: Please note that Figure S26 (shown below) and Table S8 represent the simulations carried out under 14 s reaction time conditions – all with 1 ppm of aromatic carbonyls (long residence time conditions in Table 1). In Figure S26, the solid dots are OH concentrations that showed a factor of 3.5 lower values in phenylacetaldehyde (black dots) experiment compared to acetophenone (blue dots). A higher oxidant concentration was maintained for acetophenone (ACP) compared to the other two VOCs is because the ACP + OH reactivity is lower (k = 3.86×10^{-12} cm^3 $molecule^{-1}$ s^{-1}, see Table S3 in comparison to Tables S1 and S2). Our aim was to have comparable aromatic carbonyl derived primary RO_2_C radicals (solid lines and markers in Figure S26) in the flow reactor as the starting condition. *For clarity, the legend in Figure S26a is now modified as shown below.*

Indeed, it is the oxidant OH radical which is a limiting reactant (compared to excess VOC) that controls the yield of the primary RO_2 radicals. In the case of short reaction time experiments, the reactant concentrations as well as the reaction times were chosen based on the first appearance of any HOMs in the mass spectrum. Simulations with short reaction time conditions (including 1, 1.5, and 3 ppm of benzaldehyde (BZ), phenylacetaldehyde (PA), and ACP as shown in Table 1) reveal that the concentrations of primary RO_2 radicals produced in different aromatic carbonyl oxidation experiments in 0.9 s are comparable, i.e., 1.09×10^{10}, 1.40×10^{10}, and 1.04×10^{10} molecules cm^{-3} in BZ, PA, and ACP experiment, respectively. In general, with the decrease of reaction time, unimolecular reactions become gradually more relevant than bimolecular reactions. Moreover, the bimolecular reaction rates can vary in the

range of about seven orders of magnitudes depending on specific RO_2 structures (Berndt et al., 2018; Shallcross et al., 2005).

Berndt et al. (2018), https://doi.org/10.1002/anie.201710989

Shallcross et al. (2005), https://doi.org/10.1016/j.atmosenv.2004.09.072

Changes to SI:

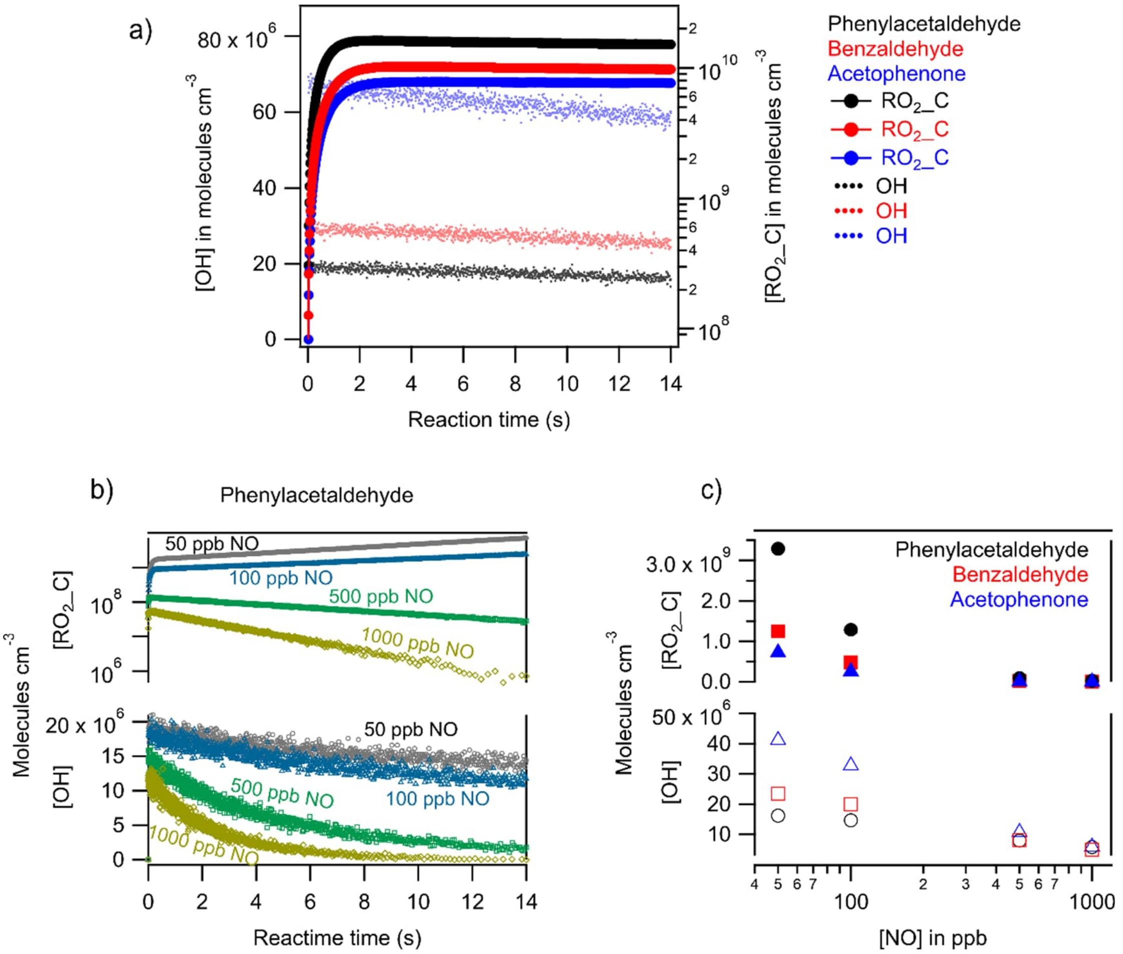

Figure S26. Concentration profiles of OH and primary RO_2_C radicals produced in aromatic carbonyl oxidation derived by chemical kinetic simulations under laboratory flow reactor experimental condition without NO (a) and with NO (b–c). Panel (b) shows results from phenylacetaldehyde. In panel (c), average concentrations of OH and RO_2_C during 14 s reaction time are presented in open and filled markers, respectively.

10. Line 347: Please provide an estimate of the NO remaining in the flow tube, or how much NO participates in the reactions.

Response: In the reactor, the sinks of NO are its reactions with OH ($k = 3.3 \times 10^{-11}$ cm^3 $molecule^{-1}$ s^{-1}), O_3 ($k = 1.8 \times 10^{-14}$ cm^3 $molecule^{-1}$ s^{-1}), and RO_2 ($k = 9.0 \times 10^{-12}$ cm^3 $molecule^{-1}$ s^{-1}). Despite having a lower rate coefficient of the reaction $NO + O_3 \rightarrow NO_2 + O_2$, the production of NO_2 is significant because the other competing reactions of NO are limited by the relatively lower concentrations of OH and RO_2 compared to O_3. For example, at 100

ppb of initial NO (2.46×10^{12} #/cm^3) condition, the simulation results show that the remaining concentrations of NO were in the range of 5.22–5.93×10^{11} #/cm^3 while NO_2 concentrations grew by 1.68–1.74×10^{12} #/cm^3 at the end of 14 s reaction time.

Addition to manuscript: In lines 380–381, "The time series of NO and NO_2 are shown in Figure S27 in the Supplementary Information."

Addition to SI: In lines 544–551, "It is also important to note that NO_2 is produced in the flow reactor via reactions of NO, while NO is directly injected into the reactor from a cylinder. The time series of NO, NO_2, and aromatic carbonyls under different initial NO conditions are shown in Figure S27. While calculating HOM yields (Figure 4 in the main manuscript), the consumed aromatic carbonyls concentrations (ΔVOC) were estimated from the difference of initial and final concentrations (see Figure S27b, d, and f). Under 100 ppb $[NO]_{t=0}$ condition, the ΔVOC concentrations were 7.82×10^{10}, 2.02×10^{11}, and 4.22×10^{10} molecules cm^{-3} of benzaldehyde, phenylacetaldehyde, and acetophenone, respectively."

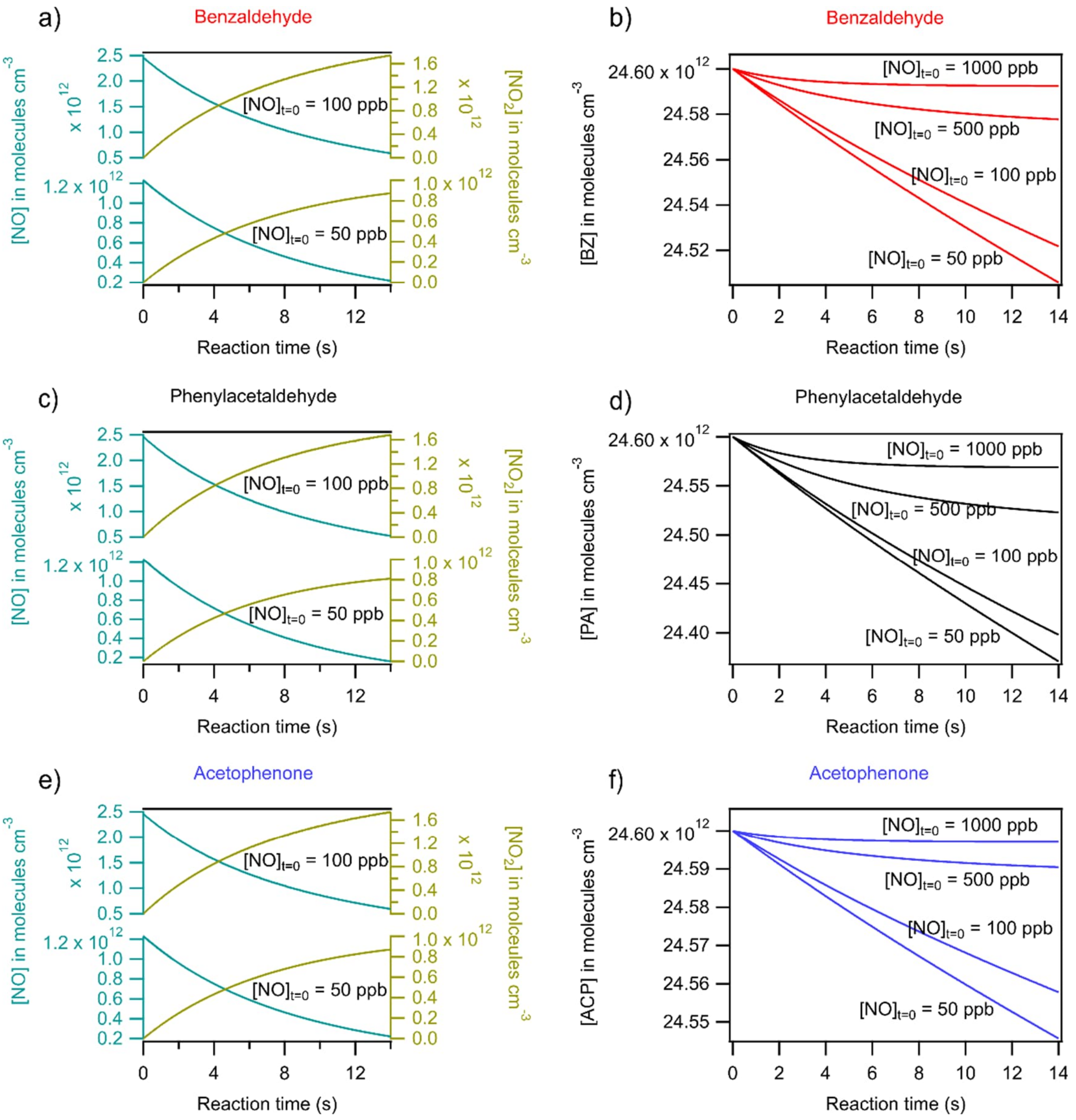

Figure S27. Concentration profiles of NO, NO_2, and aromatic carbonyls as a function of reaction time under different initial $[NO]_{t=0}$ conditions: benzaldehyde (a–b), phenylacetaldehyde (c–d), and acetophenone (e–f). Reaction time, Δt = 14 s. BZ = benzaldehyde, PA = phenylacetaldehyde, and ACP = acetophenone."

11. Lines 350–352: Please specify precursor consumption in each system, especially when comparing across precursors.

Response: In Lines 350–352, the intensities of nitrophenol signals produced during the oxidation of different aromatic carbonyls were compared. Now, in the revised manuscript, the associated figure (Figure 4b below) is modified with yields of nitrophenol instead of normalized intensities. So, the precursor VOC consumption is accounted accordingly.

Changes to manuscript: In lines 383–385, "Figure 4b shows that the yield of nitrophenol is roughly one and two orders of magnitude higher for benzaldehyde than that of acetophenone and phenylacetaldehyde, respectively, during NO injection steps between 200 ppb to 1 ppm."

12. Figure 4: Panel 4b shows negligible nitrophenol formation in the acetophenone and phenylacetaldehyde systems, but Figures 3b and 3c suggest more significant formation. What accounts for this discrepancy? If differences are indeed large across the three precursors in Figure 4b, a log-scale y-axis would help. Also, keep naming consistent (e.g., "acetophenone" in panel a vs. "$PhCOCH_3$" in panel c).

Response: We thank the reviewer for suggestions about Figure 4b scaling and naming consistency in panels a–c in Figure 4.

Regarding nitrophenol signals in Figure 3 (a–c), they represent 100 ppb NO condition and are comparable. However, the difference in nitrophenol yield for benzaldehyde compared to phenylacetaldehyde and acetophenone is significant with the increase of NO concentrations. In the revised manuscript, Figure 4b y-axis is changed to log scale (see below).

The names are corrected accordingly in the revised manuscript, i.e., phenylacetaldehyde and acetophenone instead of $PhCH_2CHO$ and $PhCOCH_3$, respectively in Figure 4c.

Changes to manuscript:

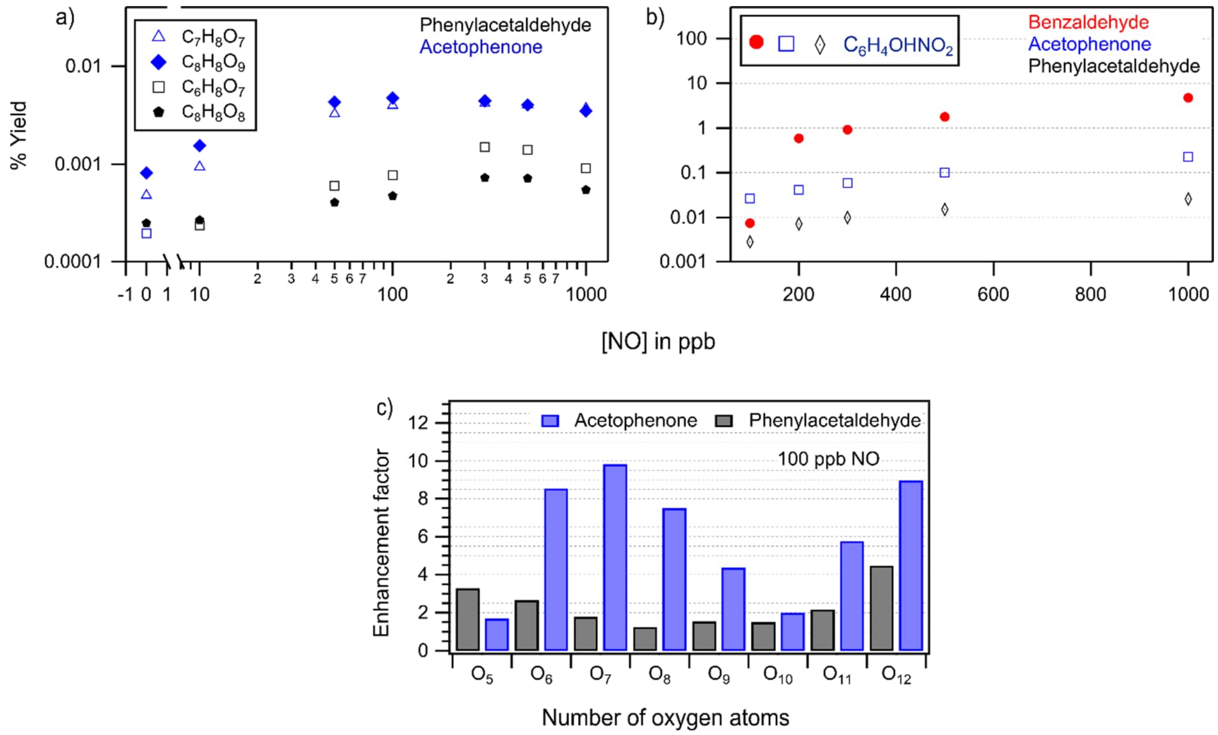

Figure 4. Enhancement of HOM yields in presence of NO during the OH initiated oxidation of phenylacetaldehyde (black) and acetophenone (blue). (a) HOM yields detected by NO_3^--CIMS as a function of NO concentration shown in a logarithmic scale. Several representative HOMs including both non-fragmented C_8 and fragmented C_{6-7} HOMs are shown in filled and unfilled markers, respectively. (b) The yield of nitrophenol ($C_6H_4OHNO_2$) as a function of NO concentration in all the aromatic carbonyl systems studied including benzaldehyde in red. Note the logarithmic scale in y-axis. (c) Total yield enhancement of oxygenated products grouped by different oxygen numbers in presence of 100 ppb NO. Reaction time, Δt = 14 s.

13. Figures 1–3: The y-axis shows normalized signals. The unit should be ncps, not counts/s.

Response: We thank the reviewer for pointing out this. In the revised manuscript, y-axis labels in Figures 1–3 were modified to "*Normalized counts* s^{-1} *(ncps)*" instead of "*Normalized signals (counts* s^{-1}*)*".

14. Volatility discussion: When discussing the relative contributions of different families with varying volatility, please clarify that HOM sensitivity is assumed equal due to their high oxidation and functionalization. However, prior work (Stéphanie Alage, 2024, AMT) showed that 4-nitrocatechol had the highest sensitivity, implying nitrophenols may have enhanced sensitivity. Could this explain the strong nitrophenol signals in your study? Please discuss the potential influence of sensitivity differences on your results and conclusions.

Response: We thank the reviewer for suggestions regarding the clarification of oxidation product sensitivity to be included in the manuscript. We acknowledged the issues accordingly in the revised manuscript.

Changes to manuscript: In lines 541–546, "We consider that the sensitivity of the oxidation products is equal because NO_3^- ionization is selective to HOMs and because of lack of methods that can account for differences in sensitivities across various oxygenated products. The exception is nitrophenol ($C_6H_4OHNO_2$) that has an anomalously high sensitivity to NO_3^--CIMS[62,63] and thus it showed the highest intensity signal among all the oxidation products in the studied aromatic carbonyl oxidation."

Reviewer #3 (Remarks to the Author):

Barua et al. 2025 report results from flow tube reactor experiments on three aromatic volatile organic compounds. They measure the yields of highly oxidised molecules from each VOC under varying chemical conditions and report a mechanism by which nitric oxide can enhance this yield. The mechanism has not been well explored before, but could have important implications for the composition of VOC oxidation products, the loading of secondary organic aerosol (SOA) and the composition of SOA.

The conclusions and claims would be significant and novel to the gas-phase chemistry community, however, as detailed below, the current work does not fully support them, and additional evidence is needed. Because of this, I do not recommend publication as is, but advise major revisions.

The derived rate coefficients are very useful findings for improving chemical mechanisms.

In my experience, the authors are correct that suppression of SOA yields from VOC (photo)oxidation by nitrogen oxides is a widely accepted process (e.g. 10.1126/sciadv.ado1482).

Line 77 – I suggest including another reference for the acceptance of NOx suppression, as this reference provides a sort of overview, rather than a focus on specific VOCs: doi.org/10.1126/sciadv.ado1482

Response: We thank the reviewer for the suggestion. The overview paper (Kenagy et al., 2024) is added as a reference in the revised manuscript.

Line 77 – is this sentence too specific? I mean, it's not only the suppression of accretion products that reduces SOA yield, it's also the suppression of RO2 autoxidation by formation of organonitrates, i.e. the degree to which autoxidation can proceed. But if my understanding is incorrect, please state in introduction that evidence so far indicates autoxidation itself is not affected by organonitrate formation, rather accretion of autoxidation products is affected.

Response: We agree with the reviewer that the sentence in its previous form sounds somewhat specific to HOM accretion products although it should not be limited to it. However, according to current understanding, the accretion products being very condensable are most important contributor to SOA and their reduction directly correlates with lower SOA yield. Also, NO_x

does not necessarily suppress HOMs in all cases. The subsequent sentences describe the process which are also related to the reviewer's next comment. We made relevant adjustments in the revised manuscript.

Changes to manuscript: In lines 79–81, "… thereby the SOA yield. In this process, the reduction of SOA yield is significantly influenced by the suppression of HOM accretion products ($RO_2 + RO_2 \rightarrow ROOR$) which are known to be very condensable.[26,28]"

Line 81 - could this sentence be better connected with the preceding sentence so that the reader knows that the references in the previous sentence detail the alkoxy radical mechanism behind NO enhancement?

Response: We thank the reviewer for pointing this out for better connectivity. Changes are made in the revised manuscript.

Changes to manuscript: In lines 82–84, "However, prior studies[29–32] have shown that in select cases low NO_x concentrations can enhance the formation of HOMs via alkoxy radical (RO) mediated chemistry in alkane and alkenes."

Line 83 – for the sentence that references Wang et al., could this be made clearer as to what the correlation between HOM yields from alkanes and NOx is? E.g., as NOx increases does HOM yield increase? At the moment the sentence just references one NOx mixing ratio rather than a trend. Also, could this sentence be extended to discuss the ratios of NOx to RO2, rather than just the absolute mixing ratio of NOx?

Response: We thank the reviewer for suggesting more clarity to be brought while discussing the findings of Wang et al. (2021) in alkane oxidation in the presence of NO. Yes, there is a trend of increasing the HOM yields with the increase of NO. The ratios of NO_x to RO_2 were not discussed explicitly in that paper.

Changes to manuscript: In lines 88–90, Moreover, Wang et al.[29] have reported that HOM yields increased with the increase of NO (0.1–10 ppb initial NO) during the oxidation of several alkanes that are key components of urban air."

Line 85 – suggest a paragraph break starting with the Pullinen et al. sentence so the reader is better able to distinguish between chemical mechanism introduction and aerosol microphysics introduction.

Response: We thank the reviewer for the suggestion. An adjustment is made in the whole paragraph (i.e., NO_x influence in VOC oxidation chemistry and subsequent SOA formation). More thoughts are added in the response to the next comment.

Line 89-94 – starting with 'In the VOC systems where HOM ..' through to the end of the paragraph, I don't understand the points being made. Are you saying that theoretically increased NO:NO2:RO2:HO2 should be able to increased HOM yield, but that consistently observations show the opposite tendency? Whatever the point(s) are, they need to be clearer, and potentially not right after a statement about uptake coefficients, since these coefficients are more about microphysics than they are about gas-phase chemistry.

Response: The idea of the whole NO_x paragraph is to highlight three points: 1) According to current understanding, NO_x suppresses HOMs and HOM accretion products. These accretion products have been implied to be very condensable, and their suppression was seen to be the major contributor to lower SOA yield. 2) Other studies have implied that NO_x can also enhance HOMs via RO mediated chemistry (alkoxy-peroxy pathway leading to reaction chain propagation). Besides, highly oxygenated organonitrates (HOM-ONs) are formed. 3) VOC oxidation cases that produce less accretion products (i.e., low dimer to monomer ratios) are expected to have less SOA suppression effect in the presence of NO. Instead, with potential increase of HOMs and HOM-ONs they may increase SOA yields given that HOM-ONs can efficiently condense on primary particles.

In the revised manuscript, we now adjusted the paragraph and moved the HOM-ON uptake coefficient information at the end.

Changes to manuscript: In lines 75–101, "In urban environments, nitrogen oxides (NO_x = NO + NO_2) contribute to a major proportion of reactive trace gases in the atmosphere due to anthropogenic emissions mainly involving combustion. During daytime, NO_x controls the oxidation cycle of VOCs by reaction with RO_2 radicals and thus greatly influences the HOM formation pathways. It is widely accepted that high NO_x suppresses HOM formation[23–27] and thereby the SOA yield. In this process, the reduction of SOA yield is significantly influenced by the suppression of HOM accretion products ($RO_2 + RO_2 \rightarrow ROOR$) which are known to be very condensable.[26,28] However, prior studies[29–32] have shown that in select cases low NO_x concentrations can enhance the formation of HOMs via alkoxy radical (RO) mediated chemistry in alkane and alkenes. While NO can terminate RO_2 autoxidation by forming closed shell organonitrates (ONs), it can also form more reactive RO radicals ($RO_2 + NO \rightarrow RO + NO_2$) that undergo fast isomerization followed by O_2 addition and regenerate RO_2. The new RO_2 with one more O atom than the preceding RO_2 can further undergo autoxidation and enhance HOM formation.[33] Moreover, Wang et al.[29] have reported that HOM yields increased with the increase of NO (0.1–10 ppb initial NO) during the oxidation of several alkanes that are key components of urban air. In the VOC systems with low dimer to monomer product ratios, the suppression effect of NO_x on SOA by the expense of only accretion products should be less. Instead, with a potential enhancement of monomeric HOMs and highly functionalized HOM-ONs in presence of NO, a corresponding increase in SOA yield could be expected.[34,35] Yet, at very high NO_x levels HOM formation appears inevitably suppressed by autoxidation terminating early at low oxygen to carbon ratios.

Pullinen et al.[26] showed that the effective uptake coefficients (γ_{eff}) of HOMs and HOM-ONs, arising from the same HOM peroxy radicals, on particles are similar in monoterpene photooxidation. Both with more than six O atoms in the precursor peroxy moiety condense by about 50% (γ_{eff} of about 0.5) to form SOA whereas those with more than eight O atoms condense by about 100%. Therefore, observing the abundance of HOM-ONs in VOC oxidation processes is of importance."

Line 98 – rather than state mixing ratios of NOx, it is more helpful that the reader is told what the ratios of NO:NO2:RO2:HO2 concentration are, so that they have a better chance to judge whether the investigation is relevant to the chemical regimes of their interest.

Response: We thank the reviewer for the suggestion. However, there are certain limitations in the flow reactor system in terms of having direct access to all reactive species concentrations (NO, NO_2, HO_2, etc.) even though no other means can track the fast formation of HOMs. We maintain a laminar flow in the reactor with a relatively much lower total flow compared to a common environmental chamber experiments and thus connecting multiple instruments in the flow reactor is not feasible. We calculated the injected reactant concentrations (i.e., VOC, TME, NO) from their flow ratios with total flow and cylinder concentrations (also shown in the response to a latter comment). The initial concentration of NO_2 is zero which is then increased by the reactions of NO + O_3 and RO_2 + NO while the concentration of NO is decreased resulting in the NO/NO_2 even below 1 depending on initial NO. From simulation under 50–1000 ppb initial NO, we found that the ratio of average NO and average NO_2 (over 14 s reaction time) is in the range of 1.4–7. Under the same initial NO conditions (50–1000 ppb), the average of all NO/NO_2 (i.e., first taking ratio at every time step and then averaging them over 14 s) is in range of 7–28.

Although quantification of RO_2 in a complex system with lots of small VOC is doable, its quantification in a system with bigger RO_2 radicals is problematic due to shifting NO_3^- sensitivity. Besides, the aromatic carbonyl oxidation spectra are dominated by closed-shell products.

In our system, the only source of HO_2 is the reaction of RO_2 radicals forming them along the oxidation process (likely via subsequent RO, i.e., RO + O_2 → R_{-H}=O + HO_2). Due to the general complexity of RO_2 bimolecular reactions and branching ratios, an accurate estimation of HO_2 cannot be achieved.

Nevertheless, we show the simulation derived time series of primary RO_2 radicals (i.e., the first alkyl peroxy radicals formed in the oxidation process), NO, and NO_2 in Section S15 in the SI.

Changes to manuscript: In lines 150–153, "… with the presence of NO with concentrations ranging from 10 ppbv to 1000 ppbv. The chosen NO range not only covers moderately polluted to highly polluted urban scenarios but also goes beyond any polluted environment allowing us to observe the extent of the effect."

Addition to SI: In lines 544–551, "It is also important to note that NO_2 is produced in the flow reactor via reactions of NO while NO is directly injected into the reactor from a cylinder. The time series of NO, NO_2, and aromatic carbonyls under different initial NO conditions are shown in Figure S27. While calculating HOM yields (Figure 4 in the main manuscript), the consumed aromatic carbonyl concentrations (ΔVOC) were estimated from the difference of initial and final concentrations (see Figure S27b, d, and f). Under 100 ppb $[NO]_{t=0}$ condition, the ΔVOC concentrations were 7.82×10^{10}, 2.02×10^{11}, and 4.22×10^{10} molecules cm^{-3} of benzaldehyde, phenylacetaldehyde, and acetophenone, respectively."

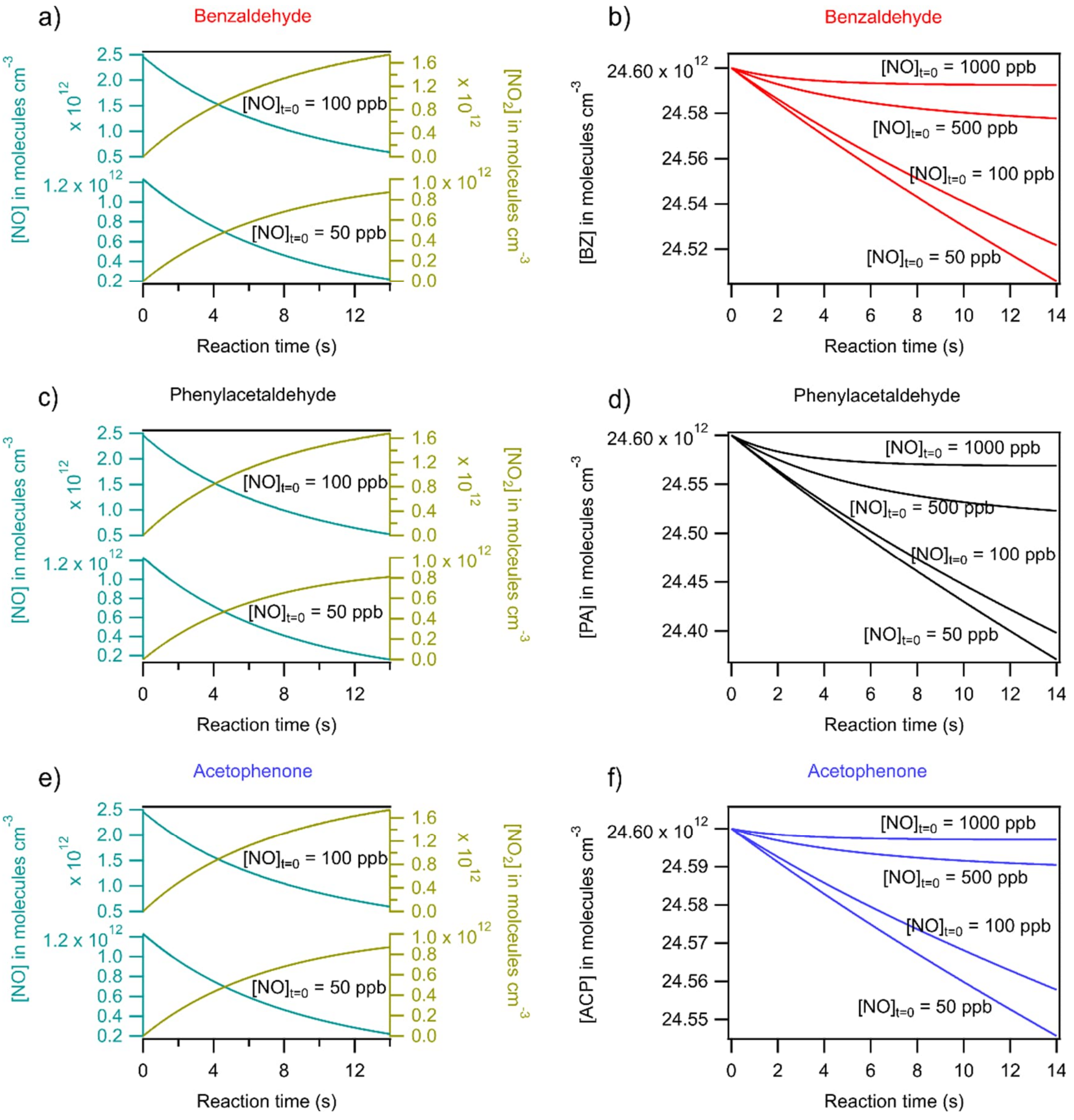

Figure S27. Concentration profiles of NO, NO_2, and aromatic carbonyls as a function of reaction time under different initial $[NO]_{t=0}$ conditions: benzaldehyde (a–b), phenylacetaldehyde (c–d), and acetophenone (e–f). Reaction time, Δt = 14 s. BZ = benzaldehyde, PA = phenylacetaldehyde, and ACP = acetophenone."

Table 1 or Section S1 - temperature, pressure (in main text/tables rather than just captions), relative humidity of experiments and any actinic flux into the flow reactor needs stating. Likewise, a statement about presence or absence of wall effects needs to be included.

Response: The relevant information (room temperature, 1 atm pressure, and < 0.1% RH) is now provided in Section S1 in the revised Supplementary Information. Because we did not introduce any photochemistry to gas mixture, no lamp was used and thus there was no actinic flux measurement. Note that we used a borosilicate flow reactor and we did not observe any effect of laboratory lights. The presence of wall effect is also now acknowledged in Section S1 in the SI.

Addition to SI: In lines 14–18, "All the experiments were conducted at room temperature and 1 atm pressure of air with < 0.1% relative humidity. Because we did not introduce any photochemistry during the experiments, no lamp was used and thus there was no actinic flux measurement. Note that with the use of a borosilicate flow reactor, we did not observe any effect of laboratory lights.

In lines 72–75, "Also, note that the flow reactor system exhibits unavoidable wall loss of reactive and condensable species, which contributes to the overall uncertainty in the experimental yields. The normal uncertainty range with the measurement technique has been assumed as asymmetric -50% to +100% (about a factor of two)."

Section 2.1 or S1 - I don't see how NO is introduced to the reactor. I don't see whether NO2 or HO2 is present in the reactor. Does nucleation of particles occur in any experiments? How is NO (and NO2) concentration measured? To estimate HOM yield, I expect the VOC concentration at the start and end of the reactor needs measuring, is this true?, if so please show the setup in Fig. S1.

Response: The reactants, including NO, were introduced into the flow tube reactor from individual gas cylinders in controlled flow using calibrated mass flow controllers (Alicat Scientific Inc.) as shown in Figure S1 in the SI. The initial concentrations of VOC and NO in the gas stream were calculated by using equation:

$$[X] = \frac{measured\ flow_x}{measured\ flow_t} \times [MR]$$

where species X can be either VOC or NO, $measured\ flow_x$ is the measured flow of the species X introduced in the gas stream, $measured\ flow_t$ is the measured total flow of the gas stream, and MR is the mixing ratio of the species X in the gas cylinder.

No direct measurement of the reactant species inside the reactor was made as explained in response to an earlier comment. The concentration of NO_2 was estimated from chemical simulation. Same for consumed VOC by calculating the difference between its initial and final concentrations from the simulation.

We do not expect significant particle formation to occur in the flow reactor with 14 s reaction time and in absence of seed particles.

Changes to SI:

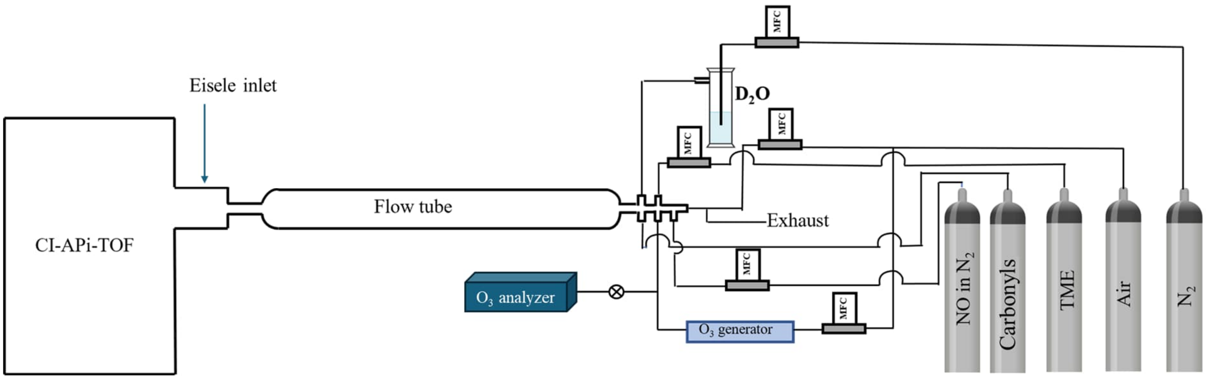

Figure S1. A nitrate (NO_3^-) based chemical ionization mass spectrometer coupled to ambient pressure flow reactor. TME = tetramethylethylene. The oxidant OH radical was produced in situ by TME + O_3 reaction. MFC = mass flow controller. CI-APi-TOF = chemical ionization atmospheric pressure interface time-of-flight mass spectrometer.

In lines 38–40, “The maximum residence time of the experiments was 14 s. The experiments were conducted in the absence of seed particles and we do not expect significant particle formation to occur.”

In lines 44–71, “The initial concentrations of VOC and NO in the gas stream were calculated by using equation:

$$[X] = \frac{measured\ flow_x}{measured\ flow_t} \times [MR]$$

where species X can be either VOC or NO, $measured\ flow_x$ is the measured flow of the species X introduced in the gas stream, $measured\ flow_t$ is the measured total flow of the gas stream, and MR is the mixing ratio of the species X in the gas cylinder. On the other hand, the time profiles of VOC, NO, NO_2, etc. were estimated from kinetic simulation model discussed in Section S15 below. The average concentrations of OH, and primary RO_2 radicals under different reaction conditions are given in Table S8.

The mass spectrometric data processing, including averaging, mass axis calibration, and peak integration, was done using the tofTools v6.03 package for MATLAB. The signal intensities of all the detected species were normalized using the following expression:

$$S = \frac{[X * NO_3^-]}{[NO_3^-] + [HNO_3NO_3^-] + [(HNO_3)_2NO_3^-]}$$

where $[X * NO_3^-]$ represents the intensity of an individual species X as NO_3^- adduct. In the denominator, $[NO_3^-]$, $[HNO_3NO_3^-]$, and $[(HNO_3)_2NO_3^-]$ represent the intensities of reagent ion monomer, dimer, and trimer, respectively.

We determined the calibration coefficient for our NO_3^--CIMS to be 2.0×10^9 molecule/cm^3/ncps by calibrating the instrument for sulfuric acid using the method shown by Kürten et al.[2] A detection limit (LOD) of 2.82×10^3 molecules/cm^3 for sulfuric acid was determined using the equation below.

$$LOD = \frac{3.3 \times \sigma}{S}$$

Here, S is the slope of the calibration curve and σ is the standard deviation of the responses of blank measurements. The HOMs were quantified based on the assumption that they are charged as efficiently as sulfuric acid. The concentrations of the oxidation products including HOMs were calculated by multiplying the individual normalized product signals with the calibration coefficient. The same calibration coefficient was commonly used for all oxidation products because of lack of methods that can account for differences in sensitivity across various oxygenated products. Then, the yields of the products were estimated by dividing their concentrations with the concentration of consumed precursor VOC (i.e., Y = ΔHOM/ΔVOC).”

Table 1 - Whilst the provided absolute mixing ratios are helpful, it would be very useful to have a column dedicated to the ratios of gas-phase concentrations of NO:NO2:RO2:HO2.

Response: We agree with the reviewer and acknowledge the usefulness of these ratios. However, as described in an earlier response to the review comment starting with Line 98, we estimated the varying concentrations (time dependent) of NO and NO_2 from chemical simulation and provided the time series in Figure S27 (see above). The simulation derived concentrations of RO_2 under different conditions and a rough estimation of HO_2 (~ 30% RO_2) are discussed in Section 15 in the SI.

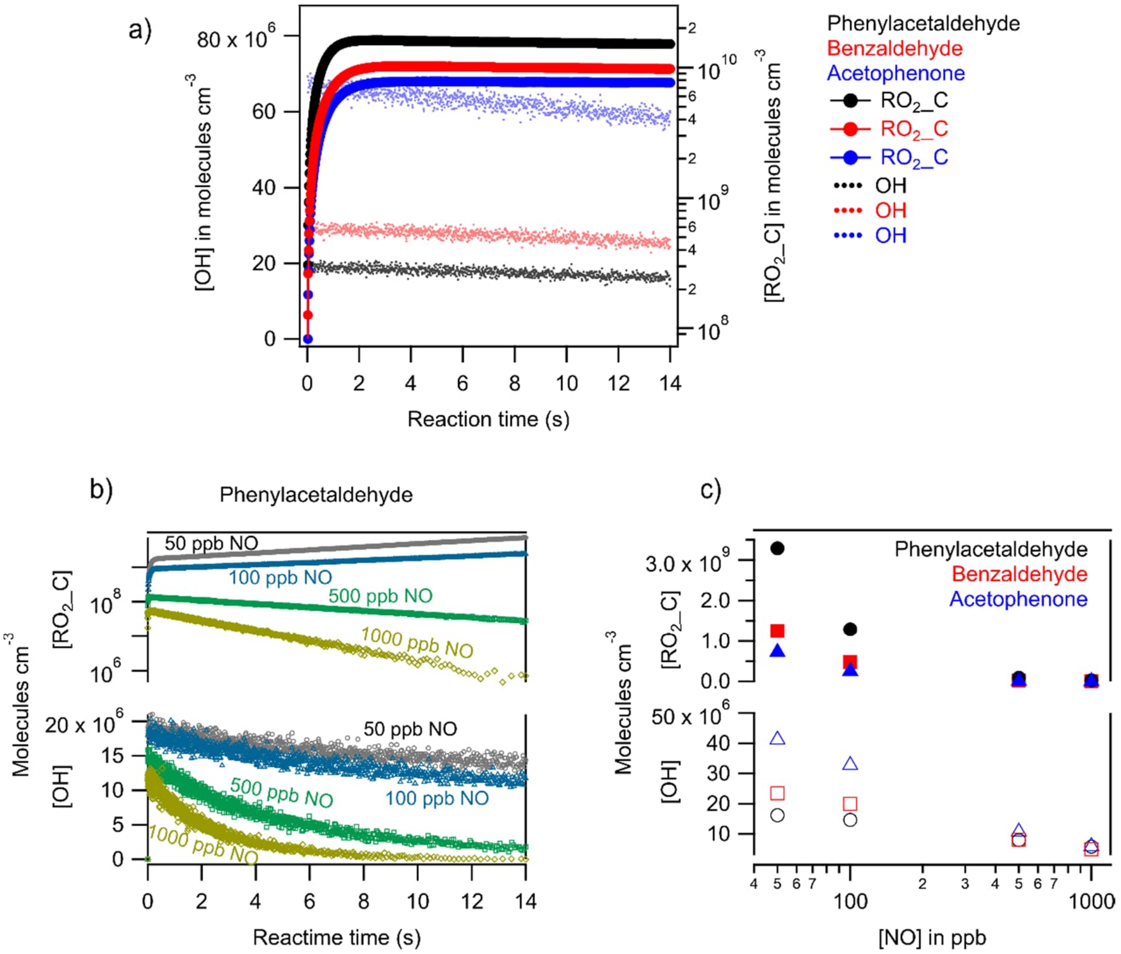

Figure S26. Concentration profiles of OH and primary RO_2_C radicals produced in aromatic carbonyl oxidation derived by chemical kinetic simulations under laboratory flow reactor experimental condition without NO (a) and with NO (b–c). Panel (b) shows results from phenylacetaldehyde. In panel (c), average concentrations of OH and RO_2_C during 14 s reaction time are presented in open and filled markers, respectively.

In lines 567–574, "In the flow reactor system, we presume that the production of HO_2 radicals is about 30% of total primary RO_2 radicals (RO_2_T + RO_2_C). Therefore, to examine the influence of the HO_2 reactions with NO and RO_2 on the concentrations of OH and RO_2_C radicals, we run a separate set of simulations on Kinetiscope including the reaction steps 13–15. An initial HO_2 concentration of 30% of total RO_2 (e.g., 4.78×10^8 molecules cm^{-3} of HO_2 in PA oxidation at 100 ppb NO) obtained from the previous simulation without HO_2 is used in

the subsequent simulation. Figure S28a shows the results of PA oxidation process without and with involving the reaction steps 13–15."

Addition to manuscript: In lines 161–162, "The time profiles of reactive species, VOC, OH, RO_2, NO, and NO_2 under different reaction conditions are shown in Figures S26–S28 in the Supplementary Information."

Table 1 – Please state where in the reactor the provided mixing ratios are applicable, e.g. at the point of injection, at the sampling point, other.

Response: We thank the reviewer for the suggestion. The reference point (i.e., point of injection) of the reactant concentrations is now mentioned as a footnote of Table 1 in the revised manuscript.

Changes to manuscript:

Table 1. The experimental conditions for OH initiated oxidation of studied aromatic carbonyls: benzaldehyde, phenylacetaldehyde, and acetophenone.

Experiment type VOC	[VOC]$^{\Phi}$ ppmv	[TME]$^{\Phi}$ ppbv	[O_3]$^{\Phi}$ ppbv	[OH]$^{\Phi \neq}$ pptv	[NO]$^{\Phi}$ ppbv	D_2O y/n$^{\pm}$	RT‡ s
**Short residence time**							
Benzaldehyde (C_7H_6O)	1	98.7	344	5.2	N/A†	n	1.1
Phenylacetaldehyde (C_8H_8O)	1.5	104.8	263	3.9	N/A	n	0.9
Acetophenone (C_8H_8O)	3	104.8	318	4.8	N/A	n	2.7
**Long residence time**							
Benzaldehyde (C_7H_6O)	1	52.4	263	3.9	N/A	n	14
Phenylacetaldehyde (C_8H_8O)	1	104.8	263	3.9	N/A	n	14
Acetophenone (C_8H_8O)	1	52.4	318	4.8	N/A	n	14
**Experiments with NO**							
Benzaldehyde (C_7H_6O)	1	52.4	263	3.9	50–1000	n	14
Phenylacetaldehyde (C_8H_8O)	1	104.8	263	3.9	10–1000	n	14
Acetophenone (C_8H_8O)	1	52.4	263	3.9	10–1000	n	14
**Experiments with D_2O**							
Benzaldehyde (C_7H_6O)	1	52.4	263	3.9	N/A	y	14
Phenylacetaldehyde (C_8H_8O)	1	104.8	263	3.9	N/A	y	14
Acetophenone (C_8H_8O)	1	52.4	318	4.8	N/A	y	14

$^{\Phi}$ Reactant concentrations at the beginning of the flow reactor.

$^{\neq}$ The initial OH concentrations (prior to adding precursor VOC, i.e., with only TME + O_3 in the flow reactor) were calculated using bimolecular rate coefficients k_{O_3-TME} = 1.5 × 10^{-15} cm^3 s^{-1}, k_{OH-TME} = 1.0 × 10^{-10} cm^3 s^{-1},[35] and the expression $[OH] = (k_{O_3-TME} * [O_3])/k_{OH-TME}$.

† Not applicable (N/A). $^{\pm}$ D_2O added = y, not added = n. ‡ Residence time (RT).

Table 1 – Either in this table (which seems appropriate), or somewhere else, the estimated OH abundance (concentration or mixing ratio) needs stating

Response: We have now included the estimated initial OH concentrations in Table 1 (see above) in the revised manuscript.

Line 205 and associated subsequent lines - To my understanding, the 2D-VBS is a framework for mapping the properties of organic molecules (either individual molecules or groups). But here it referred to as if it's a parametrisation for vapour pressure. Although the parameterisation given in Eq. 2 may be related to 2D-VBS publications, it is a method to estimate vapour pressure, which is fundamentally equivalent to the purpose of the group contribution methods described before it, and so shouldn't be confused with the 2D-VBS. Additionally, on this point, all the methods described for vapour pressure estimation may be applied to the volatility dimension of the 2D-VBS, however as it reads, only eq. 2 is applicable.

Response: We thank the reviewer for this clarification and agree that the 2D-VBS is a framework for representing compound volatility and oxidation state, rather than a direct parameterization for vapor pressure. In the manuscript, our intention was to indicate that Eq. (2), an empirical volatility parameterization proposed in 2D-VBS-related studies, was used to estimate saturation concentrations (C*) for comparison with SOA literature data, not that the 2D-VBS itself provides this equation. We revised the text to clarify that Eq. (2) is a vapor-pressure estimation expression that can be applied within the 2D-VBS framework, and that any of the described vapor-pressure estimation methods could also serve as input to the volatility axis of the 2D-VBS.

Changes to manuscript: In lines 224–229, "In order for comparing the volatility results obtained from the above approaches to values reported in several recent targeted SOA studies, the saturation concentration (proportional to volatility) of each oxidation product at 300 K is calculated within the two-dimensional volatility basis set (2D-VBS)[51] framework. For this purpose, we applied a recent empirical parameterization for complex organic mixtures derived from 2D-VBS studies to estimate the saturation concentration, as shown in Eq. (2):[52]"

Line 219 - is this describing another parameterisation, or alternative values being input to the same parameterisation (eq. 2), please clarify

Response: We thank the reviewer for the comment. The text has been revised to clarify that the Boreal-forest case uses the same parameterization (Eq. 2) with different parameter values, not a separate equation.

Changes to manuscript: In lines 240–242, "Secondly, for comparison purposes, the same parameterization given in Eq. (2) was also applied using alternative parameter values (b_O = 0.2 and b_{CO} = 0.9) that were adjusted based on a Boreal forest dataset.[54]"

Section 2.5 – please separate description of the simulations used to estimate reactive species in the conducted experiments more clearly from descriptions of simulations used to to probe the ambient urban atmosphere. For simulations of conducted experiments please specify how the simulation was setup to reproduce experimental conditions. I don't understand what random

number seed is. If details are included in Section S11, please make better reference to this. Please tell the reader where they can access the input files for the kinetic simulations.

Response: We thank the reviewer for the suggestion. The descriptions of the simulations conducted under laboratory flow reactor conditions and urban atmospheric conditions are now separated accordingly in the revised manuscript.

As Kinetoscope uses a stochastic simulation method, the random seed defines the starting point of the random number sequence that determines which reaction event occurs next and at what time. For example, in one simulation (random seed = 1), the PA + OH → RO_2_C step might be selected earlier, while in another run (random seed = 2), an alternative event such as NO + OH → HONO could occur first. Changing the seed only alters the random order in which molecules react but does not affect the underlying chemistry or the final product distribution, since all runs follow the same reaction mechanism and rate constants. In this work, a single representative random seed (e.g., 12947) was used, as the results were consistent across different seed values, confirming that the conclusions are independent of the specific random sequence used.

Information about input files is given in the "*Associated Content*" section before "*Acknowledgements*".

Changes to manuscript: In lines 246–258, "To estimate the concentrations of reactive species in the flow reactor under different aromatic carbonyl oxidation conditions, we use a kinetic simulator Kinetiscope version 1.1.1136.x64.[55] In the reaction scheme, TME ozonolysis reaction is used as OH radical source. The reactions of NO with OH, O_3, and RO_2 are included for simulating experiments in the presence of NO. The bimolecular reaction of RO_2 radicals are considered as the sink of the species. Simulations are also done to evaluate the feasibility of the formation of HOM from the oxidation of the studied aromatic carbonyls under ambient atmospheric conditions. In this case, atmospherically relevant concentrations of precursor VOCs, OH, NO, RO_2, and HO_2 are used. In both cases, a single-reactor model with constant volume, pressure, and temperature is employed in the simulation as applicable for gas phase chemistry. The temperature is set to 298.15 K. In the simulation setting, a total number of particles 1×10^9 and a random number seed 12947 are used, while the maximum simulation time is set to 14 s. Details of all the simulations are discussed in Section S15 in the Supplementary Information."

Section 2.5 and associated supplementary information (S11, by the way is S2.2 within S11 mis-numbered?) – a relatively simple chemical scheme is applied to estimate some very important values, such as concentrations of OH and HO2. However, no verification of the scheme is provided, though the available measurements must allow for this. For example, relative changes to the concentrations of the pool of carbonyl peroxy radicals. HO2 will react with RO2 to form hydroperoxides, but this is not included in the chemical scheme, it's omission needs to be justified explicitly.

Response: We thank the reviewer for pointing out the typing mistake under Section S11 in the Supplementary Information. Subsection S11.2 is now corrected which was wrong written as S2.2.

We acknowledge the simplistic nature of the current reaction scheme in the simulation model. The model estimates the concentration of carbonyl derived primary RO_2 radicals (i.e., the first alkyl peroxy radicals along the autoxidation route) from different aromatic carbonyl precursors under different conditions. Because these primary RO_2 radicals are low oxygenated species and do not have a H-bond donating group in their structures, they cannot be measured with the detection technique used. We detect the subsequent RO_2 radicals and corresponding closed-shell products along the autoxidation path where the closed-shell products dominate the mass spectra. In the simulation, accounting for the subsequent radical species and products will require the inclusion of different RO_2 branching ratios and relevant mechanisms which are currently unavailable.

We now include RO_2 + HO_2 reaction as another sink of RO_2 in the simulation models under flow reactor condition and ambient atmospheric condition and discuss the results in the revised version of the SI.

Changes to SI: In lines 561–585, "In addition, the reaction of HO_2 with RO_2 can produce closed shell hydroperoxide (ROOH) along with other products.[24] Here, we include these reactions as other sinks of RO_2 in the simulation (reaction steps 14–15).

13. HO_2 + NO => OH + NO_2 (k_{HO_2-NO} = 8.91×10^{-12} cm^3 $molecule^{-1}$ s^{-1})

14. RO_2_T + HO_2 => Sink_c ($k_{RO_2-HO_2}$ = 2.2×10^{-11} cm^3 $molecule^{-1}$ s^{-1})

15. RO_2_C + HO_2 => Sink_d ($k_{RO_2-HO_2}$ = 2.2×10^{-11} cm^3 $molecule^{-1}$ s^{-1})

In the flow reactor system, we presume that the production of HO_2 radicals is about 30% of total primary RO_2 radicals (RO_2_T + RO_2_C). Therefore, to examine the influence of the HO_2 reactions with NO and RO_2 on the concentrations of OH and RO_2_C radicals, we run a separate set of simulations on Kinetiscope including the reaction steps 13–15. An initial HO_2 concentration of 30% of total RO_2 (e.g., 4.78×10^8 molecules cm^{-3} of HO_2 in PA oxidation at 100 ppb NO) obtained from the previous simulation without HO_2 is used in the subsequent simulation. Figure S28a shows the results of PA oxidation process without and with involving the reaction steps 13–15. In all aromatic carbonyl simulations, the reaction steps 13–15 do not seem to alter the average concentrations of OH and RO_2_C radicals given that the reaction scheme (reaction steps 1–15) is lacking a constant source of 30% HO_2.

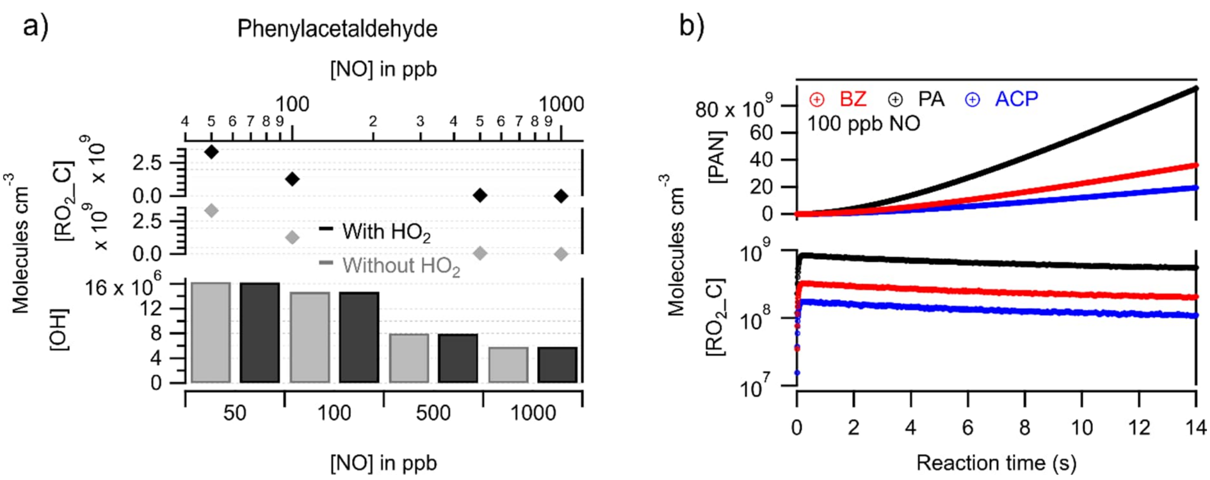

Figure S28. Average concentrations of OH and primary RO_2_C radicals produced in phenylacetaldehyde oxidation derived by chemical kinetic simulations under laboratory conditions with varying NO (a). In panel (a), simulations that include the reactions of HO_2 with NO and RO_2 (reaction steps 13–15) are presented in black, while simulations without these reaction steps are presented in grey. Panel (b) shows the time series of RO_2_C radicals, and maximum possible peroxyacyl nitrate (PAN) concentrations formed in different aromatic carbonyl OH oxidation reactions under 100 ppb initial NO condition. PA = phenylacetaldehyde (black), ACP = acetophenone (blue), and BZ = benzaldehyde (red)."

In lines 636–640 (ambient atmospheric condition), "Considering a lower limit of atmospheric RO_2 as 1.0×10^7 molecules cm^{-3} in VOC limited condition and a higher limit of atmospheric RO_2 as 1.5×10^9 molecules cm^{-3} in NO_x limited condition, we use a generic value of [RO_2] = 5.0×10^8 molecules cm^{-3} in the simulation. The concentration of HO_2 radicals is assumed to be the same as RO_2 so that we maintain an initial [HO_2]/]RO_2] = 1."

Method in general – HOM yield needs to be defined. Also, more needs to be done (probably in section 2.1) to tell the reader how comparable experiments at varying NOx are, even if this only means better reference to supplementary information sections. For example, although ozone and TME mixing ratios are comparable between w/o NOx and w/ NOx experiments, the introduction of NOx will perturb the NO:NO2:RO2:HO2 concentration ratios, as well as the OH concentration, so the reader needs some information about these things to prepare for interpreting the results and conclusion section.

Response: We thank the reviewer for the suggestions. More details about experimental methodology including how HOMs yields were calculated are now provided in Section S1 of the revised SI. The overlaid spectra in Figure 3 were obtained by regulating NO gas flow off (without NO expt.) and on (with NO expt.) while keeping the other reactant concentrations constant. We estimated different reacting species from simulation model and reported the results in Figures S26–S28 and Table S8–S9 in the SI. We observed a decreasing trend of oxidant OH radicals and primary RO_2 radicals with the increase of NO as expected. These conditions are now better connected in Section 2.1 in the revised manuscript with reference to the SI. The results imply that while NO decreases the primary RO_2 concentrations, it also forms more reactive RO radicals via $RO_2 + NO \rightarrow RO + NO_2$ reaction which can subsequently isomerize and add more oxygen atoms promoting enhanced HOMs as observed experimentally.

Changes to manuscript: In Lines 161–162, "The time profiles of reactive species, VOC, OH, RO_2, NO, and NO_2 under different reaction conditions are shown in Figures S26–S28 in the Supplementary Information."

Addition to SI: In lines 48–51, "On the other hand, the time profiles of VOC, NO, NO_2, etc. were estimated from kinetic simulation model discussed in Section S15 below. The average concentrations of OH, and primary RO_2 radicals under different reaction conditions are given in Table S8."

In lines 65–71, "The HOMs were quantified based on the assumption that they are charged as efficiently as sulfuric acid. The concentrations of the oxidation products including HOMs were calculated by multiplying the individual normalized product signals with the calibration

coefficient. The same calibration coefficient was commonly used for all oxidation products because of lack of methods that can account for differences in sensitivity across various oxygenated products. Then, the yields of the products were estimated by dividing their concentrations with the concentration of consumed precursor VOC (i.e., Y = ΔHOM/ΔVOC)."

Method in general – the NO:VOC ratio for the investigated VOCs is likely to be greater than 1 throughout the troposphere. However, the experiments here do not exceed 1. Could the authors therefore explain how their experiments are relevant to the troposphere, and particuarly urban areas? Similarly, could the authors explain how the method can provide evidence that even at high NO:VOC concentration ratio, HOM yields are relatively high?

Response: In the troposphere, although the concentration of these individual VOCs can be lower than that of NO, the total VOC concentration is higher. We are also aware of the concept of VOC-limited ([VOC]/[NO_x] < 5.5) and NO_x-limited ([VOC]/[NO_x] > 5.5) conditions and acknowledge the overall complications in chemical transformation processes in the troposphere. NO_x interferes with all VOC-related chemistry and is not limited to an individual VOC. In the flow reactor, we cannot exactly follow the atmospheric NO:VOC condition of an individual VOC. This is because excess NO can significantly influence VOC + OH reaction as both VOC and NO compete for reaction with OH (in the atmosphere it is NO_2 that competes with VOC but in the flow reactor NO_2 is formed progressively via NO + $O_3 \rightarrow NO_2 + O_2$ and RO_2 + NO $\rightarrow$ RO + NO_2 reactions within the reaction timescale). On the other hand, the formation of primary RO_2 radicals, that drive subsequent autoxidation and HOM formation, in VOC + OH reaction in the flow reactor is limited by the concentration of reactant OH radicals (limiting reactant) despite having a higher VOC concentration. Therefore, the selection of NO concentrations in the experiments is made to observe the trend of oxidation products over a wide range of NO (low to high).

The role of NO in promoting HOM formation in different VOC oxidations is reported in previous studies (Rissanen, 2018; Z. Wang et al., 2021; Nie et al., 2023). The reaction of RO_2 with NO forming RO and subsequent fast RO isomerization reaction play an important role in higher HOM turnover in the oxidation process.

Changes to manuscript: In Lines 150–153, "… with the presence of NO with concentrations ranging from 10 ppbv to 1000 ppbv. The chosen NO range not only covers moderately polluted to highly polluted urban scenarios but also goes beyond any polluted ambient environment allowing us to observe the extent of the effect."

Figure S1 – please include the location of additional sampling instruments, such as the ozone analyser and the instrument for measuring NO and VOC.

Response: We thank the reviewer for suggestions regarding additional information in Figure S1. The location of ozone analyzer is now marked in the modified Figure S1. However, we did not have direct measurements of NO and VOC as explained in the response to an earlier comment.

Changes to SI:

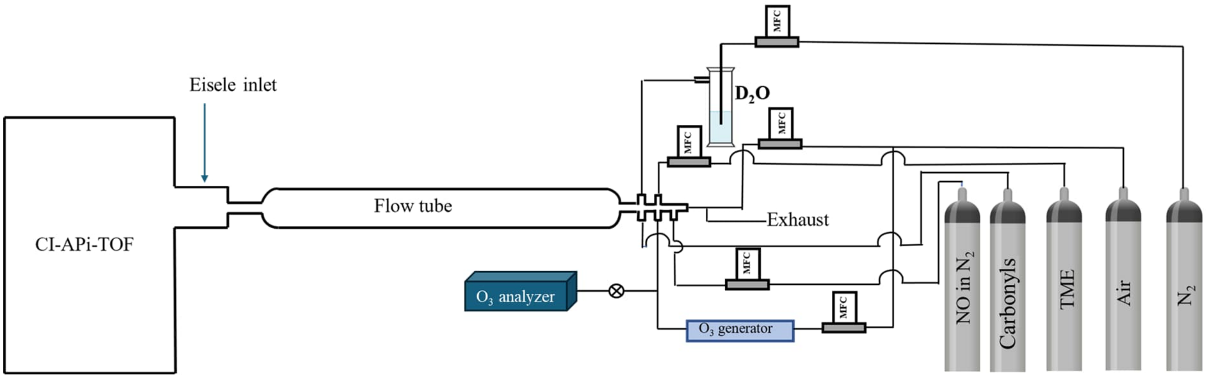

Figure S1. A nitrate (NO_3^-) based chemical ionization mass spectrometer coupled to ambient pressure flow reactor. TME = tetramethylethylene. The oxidant OH radical was produced in situ by TME + O_3 reaction. MFC = mass flow controller. CI-APi-TOF = chemical ionization atmospheric pressure interface time-of-flight mass spectrometer.

Section 3.1 – please do more (e.g. in Fig. captions) to remind the reader that the presented results are for experiments excluding NOx.

Response: The figure captions in Section 3.1 are modified accordingly in the revised manuscript.

Changes to manuscript: In lines 261–262, "In this section, we discuss the formation of HOMs observed in variable residence time experiments of OH initiated aromatic carbonyls oxidation without the presence of NO."

In Figure 1 caption, "… OH initiated oxidation of aromatic carbonyls (without NO) showing the formation of HOMs …"

In Figure 2 caption, "… OH initiated oxidation of aromatic carbonyls (without NO) in 14 s residence time …"

Section 3.2 – this results section is key to the significance of the reported finding that HOM yields increase with increasing NO. However, there are several issues: i) The text suggests that HOM yield is shown in Figs. 3 and 4, however, the ordinate of these plots is a normalised signal. To my understanding, showing a HOM yield would require normalised signal to mean signal divided by consumed VOC, is this the case? If it is, it needs stating that this is what normalised signal means. If it's not, then the main text needs changing to correctly refer to what the plots are showing. Similarly, does enhancement factor in Fig. 4 refer to enhancement of signals normalised by VOC consumption?

Response: We thank the reviewer for pointing out this. *In the revised manuscript, we now modified Figure 4 with yields (measured HOM concentrations divided by consumed VOC).* However, the mass spectra (Figures 1–3) are still presented as normalized signal intensities which is very common to show a continuous mass axis covering the mass range of interest. This preserves the originality of the raw mass spectra while the normalization process helps to overcome any difference in reagent ion stability enabling a fair comparison among different

spectra measured under comparable initial conditions. Accordingly, we now carefully replaced the word "yields" by "signal intensities" in the relevant places.

Changes to manuscript:

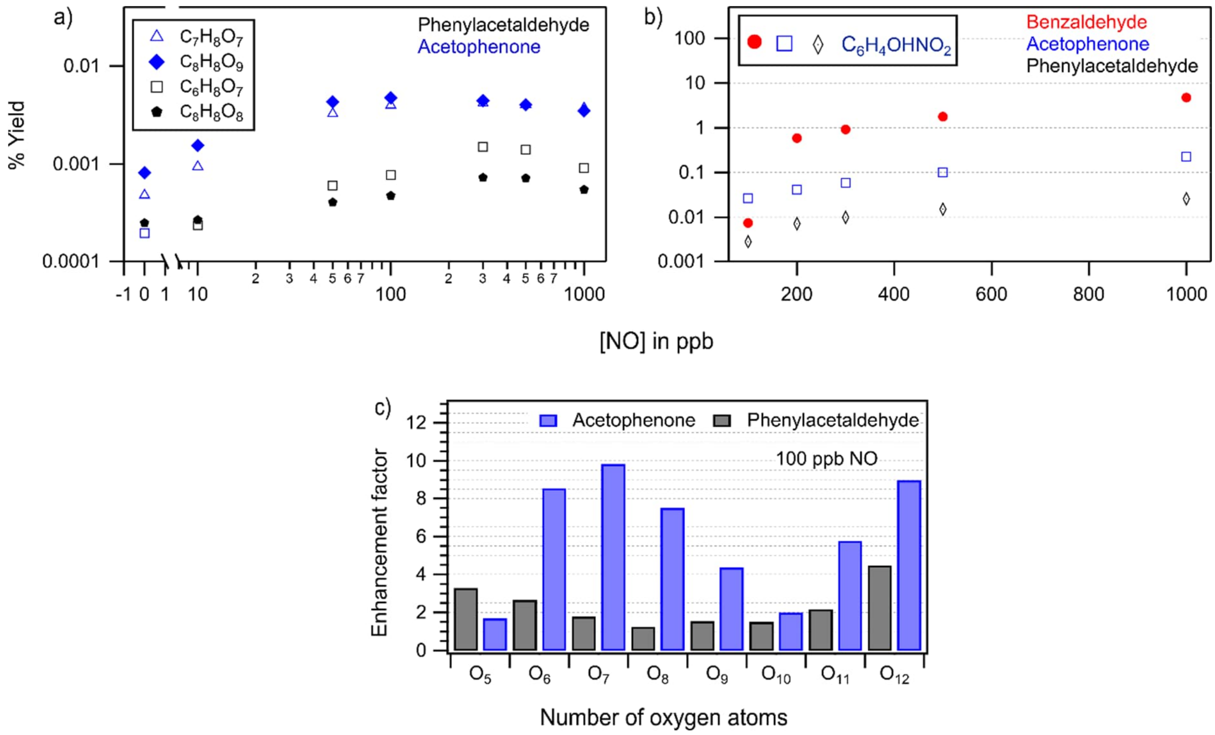

Figure 4. Enhancement of HOM yields in presence of NO during the OH initiated oxidation of phenylacetaldehyde (black) and acetophenone (blue). (a) HOM yields detected by NO_3^--CIMS as a function of NO concentration shown in a logarithmic scale. Several representative HOMs including both non-fragmented C_8 and fragmented C_{6-7} HOMs are shown in filled and unfilled markers, respectively. (b) The yield of nitrophenol ($C_6H_4OHNO_2$) as a function of NO concentration in all the aromatic carbonyl systems studied including benzaldehyde in red. Note the logarithmic scale in y-axis. (c) Total yield enhancement of oxygenated products grouped by different oxygen numbers in presence of 100 ppb NO. Reaction time, Δt = 14 s.

Section 3.5 – again I think results need to be normalised by VOC consumption to make a fair comparison. I like that attention is given as to which HOM monomers are changing abundance, e.g. N-containing and non-N-containing. Could the change in abundance of accretion products also be reported?, as the introduction suggests this is important when considering NO influence.

Response: While we discussed trend of HOM yields under variable NO concentrations in Section 3.2 (Figure 4 in the revised manuscript – see above), we focused on the distribution of oxidation products in different volatility bins taking 100 ppb NO condition as an example in Section 3.5. Normalizing the numbers with consumed VOC will not alter the volatility distribution of the products of individual VOC (pie charts in Figure 6). On the other hand, the individual bar plots with the real numbers in normalized counts per second (ncps) represent the abundance of the oxygenated species with and without NO (switching NO gas line on and off) conditions while the other reactants remained the same. Please also note that at higher NO conditions (under 500 and 1000 ppb NO), the abundance of HOM is lower than that of without

NO condition (ncps values shown in Figure S10a in the SI) although the corresponding yield (Figure 4a above) shows higher which is because the amount of consumed VOC is decreased with the increase of NO.

The accretion products are dropped in the presence of NO which is unsurprising and is now mentioned in the revised manuscript.

Changes to manuscript: In line 368, "However, the intensities of the HOM accretion products decreased as expected."

Final paragraph of section 3.5 and related supplementary information – the NO concentration (1 ppb) used for the polluted urban scenario is about a factor of 100 too small, e.g. Fig. 1 of https://doi.org/10.1016/S1352-2310(01)00378-8. By omitting simulation of representative urban conditions, the implications of the study are substantially curtailed.

Response: We agree with the reviewer that the NO concentrations (0.1 and 1 ppb) used in the atmospheric simulation model represent cleaner to low NO conditions. The concentration of NO at Hyytiälä SMEAR II station (boreal forest) in Finland quite often ranges within several tens of ppt (Liebmann et al., 2018). https://doi.org/10.5194/acp-18-3799-2018. The average NO concentrations in Nordic urban areas, Helsinki, Oslo, Copenhagen, etc., are limited to only a few ppb compared to megacities like London often ranging within several tens of ppb (sometimes even touching 100 ppb) (Clapp et al., 2001). https://doi.org/10.1016/S1352-2310(01)00378-8. Thus, we feel the selection of NO range in the simulation is representative of an urban atmosphere. A "low-NO_x" environment may be thought of the condition when the fate of RO_2 radicals is dominated by isomerization, and self-reaction in a laboratory setup or via a reaction with HO_2 forming hydroperoxides in the atmosphere (Wennberg, 2023). Interestingly, while "high-NO_x" and "low-NO_x" terminologies have been widely used to describe a photochemical condition in the literature, they do not have precise definitions and thus use of more descriptive alternatives (e.g., conditions relevant to fate of RO_2) is appreciated instead (Wennberg, 2023). https://doi.org/10.1021/acsestair.3c00055.

It should be noted that we experimentally observed the formation of HOM in as low as sub-second time scale without the presence of NO. In the presence of high NO concentrations, in 14 s reaction time, we observed enhancement in HOM formation. However, the current simulation only accounts for the suppression effect of NO by including competing reactions with RO_2 autoxidation (lines 698–701 in the SI). To account for the enhancement effect of NO, the simulation requires inclusion of RO mediated reaction kinetics which we are missing and are computationally challenging to achieve. Therefore, in the simulation, we were limited to low NO conditions. Despite having the suppression role of NO in the simulation, the results reflect the experimentally observed fast formation of HOM and thus support our computationally explored autoxidation mechanism.

We thank the reviewer for bringing this point and have adjusted the relevant sentences in the revised manuscript and in the SI.

Changes to manuscript: In lines 561–562, "… we conducted kinetic simulations using our flow reactor conditions and those relevant to the ambient atmospheric conditions." instead of "… those relevant to the urban atmosphere."

Changes to SI: In lines 632–636, "In our simulation, considering an ambient atmosphere, the initial concentration aromatic carbonyl is given 1ppb (2.46×10^{10} molecules cm^{-3}). A generic concentration of oxidant [OH] = 1.0×10^{7} molecules cm^{-3} is used. Two different concentrations of NO (0.1 and 1 ppb, i.e., 2.46×10^{9}, and 2.46×10^{10} molecules cm^{-3}) are used to mimic moderately clean to low NO_x conditions."

Generally in results, it is not clear whether the reported enhancement of HOM yield is due only to direct influence of NO on RO2 (e.g. encouraging the alkoxy channel through RO2+NO), or whether it's due to NO addition decreasing hydroperoxide formation through decreasing HO2 (RO2+HO2->ROOH). I think this needs to be explored more to provide better insight into these key mechanisms. For example, by reporting changes to signals of highly oxidised and less highly oxidised closed-shell hydroperoxides (without N) as NO concentration changes.

Response: While the RO_2 reaction termination via HO_2 (i.e., $RO_2 + HO_2 \rightarrow ROOH + O_2$) produces closed-shell hydroperoxide, the self- and cross reactions of RO_2 (i.e., $RO_2 + R'O_2 \rightarrow R_{-H}{=}O + R'OH + O_2$) produce closed-shell carbonyls and alcohols. Both ROOH and ROH contain one more H atom than the corresponding RO_2 but differ by only one O atom. Therefore, it is experimentally challenging to distinguish between them from their chemical compositions. However, the observations from H/D exchange experiments that we commonly use as a tool to validate probable reaction mechanisms (discussed in Section 3.4) allow us to relate their identities (ROOH and ROH) to some extent. Despite this, there might be yet unexplored branching and multi-step mechanisms with unknown rates that can contribute to the oxidation products with the same chemical composition. Thus, the same chemical composition can have contributions from both ROOH and ROH.

In the presence of NO, a decrease in HO_2 is expected limiting the reaction $RO_2 + HO_2 \rightarrow ROOH$. At the same time, NO can also limit the $RO_2 + RO_2$ reactions and the formation of closed-shell ROH. On the other hand, the reaction of RO_2 with NO produces alkoxy radical RO and the alkoxy-peroxy pathway can lead to oxidation chain propagation enhancing HOMs (https://acp.copernicus.org/articles/25/15715/2025/).

In our system, the only source of HO_2 is the reaction of RO_2 radicals forming them along the oxidation process (likely via subsequent RO, i.e., $RO + O_2 \rightarrow R_{-H}{=}O + HO_2$). We did not have a direct measurement of HO_2. Due to the general complexity of RO_2 bimolecular reactions and branching ratios, an accurate estimation of HO_2 also cannot be achieved.

We now plotted the signals (in normalized counts per second, ncps) of closed-shell $C_8H_{10}O_z$ products (i.e., ROOH and (or) ROH) in phenylacetaldehyde and acetophenone oxidation as a function of varying NO concentrations (see below) and discussed the trend in Section S12 (Additional insights from NO experiments) in the SI.

Changes to manuscript: In lines 429–431, "More insights into the abundance of HOMs under varying NO concentrations with focus on closed shell $C_xH_{y+2}O_z$ products are discussed in Section S12 in the Supplementary Information."

Changes to SI: In Section S12 lines 310–341, "We also inspected the signals of closed-shell $C_8H_{10}O_z$ products (i.e., ROOH and (or) ROH) in phenylacetaldehyde and acetophenone oxidation as a function of varying NO concentrations. In the presence of NO, we observed a

decreasing trend in many of the $C_8H_{10}O_z$ signals (black markers) while some of them showed an initial increase (green markers) in phenylacetaldehyde oxidation (Figure S11a). The opposite – an initial increase in many and a constant decrease in some products – was seen in acetophenone oxidation (green and black markers in Figure S11b). This reflected in the total $C_8H_{10}O_z$ signals (red markers in Figures S11a–b) as a constant decreasing trend in phenylacetaldehyde but as an initial increasing trend in acetophenone oxidation experiments with the increase of NO. Note that the signals that showed an initial increase in their intensities under varying NO, started to go below their initial signal levels (i.e., the signal level when [NO] = 0 ppb) only under very high NO concentrations (i.e., mostly from 500 ppb onwards).

In the presence of NO, a decrease in both HO_2 and RO_2 is expected limiting the reaction $RO_2 + HO_2 \rightarrow ROOH$. At the same time, NO can also limit the $RO_2 + RO_2$ reactions and the formation of closed-shell ROH via Russell mechanism ($RO_2 + R'O_2 \rightarrow R_{-H}{=}O + R'OH + O_2$). On the other hand, the reaction of RO_2 with NO produces alkoxy radical RO and the alkoxy-peroxy pathway can lead to oxidation chain propagation enhancing HOMs.[10] Furthermore, the alkoxy radical RO upon reaction with O_2 can form closed-shell carbonyl ($R_{-H}{=}O$) and recycle HO_2 ($RO + O_2 \rightarrow R_{-H}{=}O + HO_2$). Due to the general complexity of RO_2 bimolecular reactions and branching ratios, an accurate estimation of HO_2 cannot be achieved. Therefore, a direct correlation between HO_2 and $C_8H_{10}O_z$ and their underlying mechanisms cannot be established with certainty.

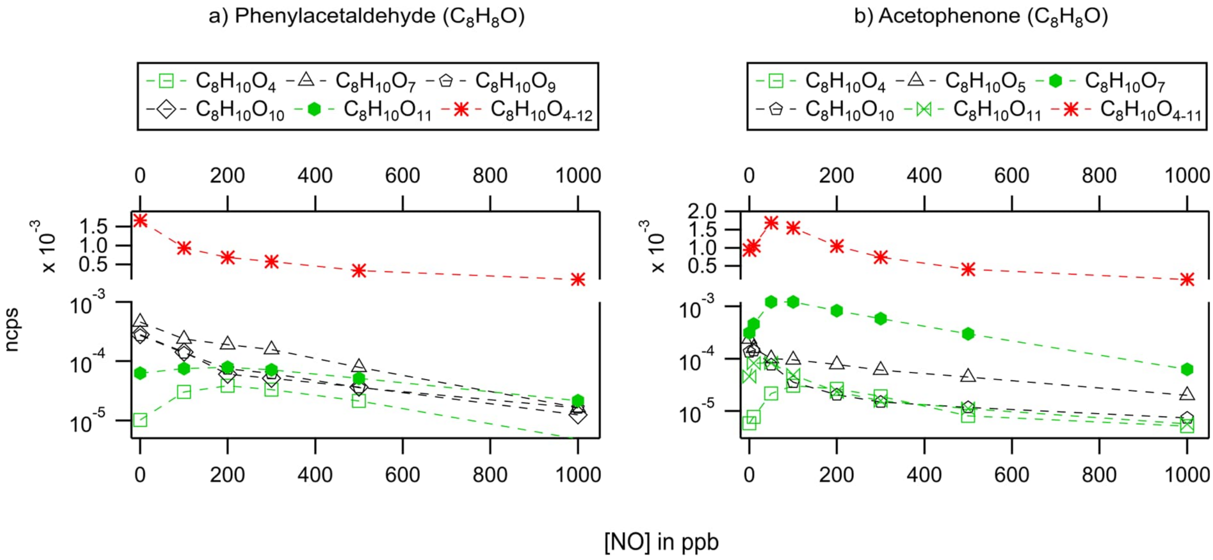

Figure S11: Closed-shell $C_8H_{10}O_z$ product signals as a function of initial NO concentrations in phenylacetaldehyde (a) and acetophenone (b) oxidation experiments initiated by OH radicals. The sum of the products ($C_8H_{10}O_{4–12}$ in panel (a) and $C_8H_{10}O_{4–11}$ in panel (b)) are presented in red markers and excludes $C_8H_{10}O_6$ that overlaps with nitrophenol-$HNO_3NO_3^-$ adduct signal. The products beyond O_{12} in phenylacetaldehyde and beyond O_{11} in acetophenone were also excluded, which did not form in the absence of NO. Products shown with green markers showed an initial increase with NO and dropped below the initial signal level only under very high NO conditions (mostly 500 ppb onwards). The black markers represent the products that showed a decreasing trend with increasing NO."

Discussion generally – could the authors explicitly state what work they think is needed to ensure we correctly capture the chemical behaviour of gas-phase organic radicals of different parent VOC under varying chemical regimes, such that we may accurately represent gas-phase chemistry in atmospheric simulation?

Response: We thank the reviewer for asking our thoughts around this to be added to the manuscript. We have now added this at the end of the *atmospheric implications* section in the revised manuscript.

Addition to manuscript: In lines 570–581, "This work also highlights the non-identical behavior of oxidation across similar species (aromatic carbonyls). The tracking of complex oxidation chemistry leading to functionalized aerosol precursors from different VOCs in future studies requires information from several complementary investigations. Short reaction timescales provide key information on the efficiency of the pseudo-unimolecular autoxidation propagation by peroxy radicals, whereas longer reaction timescales and co-reactant addition, e.g., NO illustrate the ultimate direct aerosol precursor formation processes. Importantly, several complimentary detection schemes are needed as none of the main CIMS-based methodologies are able to cover the whole condensable, and key radical, distributions by themselves. Furthermore, while computational insights into the oxidation mechanisms enable us to determine the key pathways to the formation of the important low-volatility products, experimental structural insights into these products aid in better volatility predictions."

References
Line 35, references 1-3 are quite old and focus on the emissions from transport sources, but they do not seem to verify the claim that 'aromatic carbonyls are major air pollutants'. Could the authors reference some more recent literature that better reports the relative importance of these compounds to air quality?

Response: We thank the reviewer for pointing out the need of more recent citations in favor of the statement "Aromatic carbonyls are major air pollutants …". Recent citations, Ho et al. (2002), Uebori et al. (2004), Igua et al. (2008) and Yang et al. (2023) are included in the revised manuscript.

Ho et al. (2002), https://doi.org/10.1016/s1352-2310(01)00570-2
Uebori et al. (2004), https://doi.org/10.2116/analsci.20.1459
Igua et al. (2008), https://doi.org/10.1002/cphc.200800144
Yang et al. (2023), https://doi.org/10.1016/j.atmosenv.2023.119905

The manuscript is generally very well written, there are just some minor points that need attention:

Line 36: 'constitute indoor air at significant concentrations' doesn't quite make sense, something more like: 'are present in indoor air at significant concentrations' is preferred

Response: Thanks for the suggestion. The sentence is modified accordingly in the revised manuscript.

Changes to manuscript: In lines 35–36, "… are present in consumer products, and are available in indoor air at significant concentrations"

Line 56: 'refer to HOM'?

Response: Thanks for the suggestion. The sentence is modified accordingly in the revised manuscript.

Changes to manuscript: In lines 58–59, "Here, we refer to HOM as the oxidation products that contain $\geq$ 6 O atoms in their chemical composition.[17]"

Simon P. O'Meara

We thank the reviewer for going through our responses to reviewer's comments and the revised manuscript titled "*Nitric oxide enhances secondary aerosol precursor formation from aromatic carbonyls*". We acknowledge the reviewer's concern around the atmospheric relevance of the concentration ratios of NO to RO_2 used in the experiments. Below, the reviewer comments are reproduced in red, followed by detailed author responses in blue, and the changes to the revised manuscript in purple.

Reviewer #3

The authors have largely done an excellent job at addressing the reviewer's comments. I appreciate the substantial investment in time and effort. The results are beneficial to the scientific community and need to be published, however, as detailed below, I am not convinced the signficance is sufficient for Nature Communications.

There remains a major issue with the aspect around the significance of the results for the urban ambient atmosphere, specifically whether the molecular concentration ratio of NO to RO2 from aromatic carbonyls does indeed reach the ratios relevant to the urban atmosphere. The authors state that the NO range covers moderately polluted to highly polluted urban scenarions (section 2.1), whilst in the abstract and conclusion they say that results are relevant to our understanding of polluted air, therefore this aspect is key to the study's stated significance.

In their response to referee 3 comment:

'Method in general – the NO:VOC ratio for the investigated VOCs is likely to be greater than 1 throughout the troposphere. However, the experiments here do not exceed 1. Could the authors therefore explain how their experiments are relevant to the troposphere, and particularly urban areas? Similarly, could the authors explain how the method can provide evidence that even at high NO:VOC concentration ratio, HOM yields are relatively high?'

they respond stating that OH is the limiting reactant to the formation of primary aromatic carbonyl produced RO_2 radicals (RO2_C). I agree, I think the the relative strength of NO reaction with RO2_C against the strength of RO2_C production is key.

Below I provide some calculations to show why I think the rate of NO reaction with RO2_C in the experimeriments is too low relative to the production rate of RO2_C to be representative of the urban atmosphere:

Taking acetophenone as an example, where the literature review at Acetophenone | C6H5COCH3 | CID 7410 - PubChem reports an atmospheric mixing ratio of 0.04 ppb in the US.

And assuming a concentration of OH in ambient air of $5x10^6$ molecules cm^{-3} (Tropospheric OH and HO 2 radicals: field measurements and model comparisons - Chemical Society Reviews (RSC Publishing) DOI:10.1039/C2CS35140D).

And using the rate coefficient equation from the Master Chemical Mechanism v3.3.1 for acetophenone+OH of $6x10^{-12}$ cm^3 $molecule^{-1}$ s^{-1}:

The reaction rate of VOC with OH to produce RO2_C is expected to be:

[ACETOPHENONE (molecules cm^{-3})][OH (molecules cm^{-3})]$6x10^{-12}$

Under the ideal gas law at temperature (298.15 K) and pressure (101325 Pa) relevant to the ambient atmosphere and the flow reactor experiments there is a total concentration of molecules in air of $2.46x10^{19}$ molecules cm^{-3}.

Under ambient conditions the VOC+OH reaction rate is therefore:

$(0.04x10^{-9}*2.46x10^{19})*5x10^{6}*6x10^{-12}$

= approximately $3x10^4$ molecules/cm^3/s for the rate of RO2_C production in the ambient urban atmosphere, whilst NO is typically around $2.46x10^{11}$ molecules cm^{-3} (10 ppb) in urban atmospheres.

Under the experimental conditions here, the reactivity of VOC with OH is dominated whilst [OH] is relatively high in the first 4 seconds of the experiment, with Fig 26 giving a conservative value around $1x10^6$ molecules cm^{-3}, and Table 1 showing 1 ppb of VOC. The resulting RO2_C production rate is:

$(1x10^{-6}*2.46x10^{19})*1x10^{6}*6x10^{-12}$

= approximately $1.5x10^8$ molecules/cm^3/s whilst NO has a maximum of $2.46x10^{13}$ molecules/cm^3 (1 ppm) in experiments.

Therefore, the production rate of RO2_C is approximately 5000 times higher in experiments than in the ambient atmosphere. However, the NO is increased approximately 100 times higher in experiments than in the ambient atmosphere.

For comparison against an urban atmosphere of 10 ppb NO, and using the values for acetophenone and OH concentrations above, the NO mixing ratio used in these experiments is therefore a factor of 50 too small to match the ambient condition. Even for a relatively low NOx location such as Nordic urban areas with a NO value of 1 ppb, the NO mixing ratio used in these experiments is a factor of 5 too small.

Therefore, whilst the results presented here may be useful for scenarios of relatively high VOC to NOx emission ratio, which may be true in some current non-urban areas, and may possible in future scenarios of urban areas, I do not see how the results can be said to apply to current or past urban areas.

Unless the authors can satisfactorily address this issue, I suggest the significance of the paper is insufficient for publication in Nature Communications.

Simon P. O'Meara

Response: We appreciate the reviewer's comments. Here, we present a detailed view on the common ambient conditions, especially relating to the expected concentrations of the reacting partners. A discussion on the constraints of laboratory flow reactor experiments, especially when conducted at short reaction times, is also added.

The ambient concentration regimes

The atmospheric concentrations of these aromatic carbonyls have yet to be definitively ascertained, as they are rarely selected as primary target analytes. Benzaldehyde (BZ) is the simplest aromatic aldehyde with characteristic fragrance resembling bitter almonds and cherries and is commonly used in flavoring and odorants. It is also a product of biomass burning and commonly observed in traffic emissions and consequently is found in significant quantities in both indoor and outdoor air. Phenylacetaldehyde (PA) is a major component of tea plants (Zeng et al., 2023; Xiang et al., 2024) and flowers of several species, and reflecting this has been used in perfumes and odorants for over a century but also in the synthesis of polymers (Kohlpaintner et al., 2013). Acetophenone (ACP) is the simplest aromatic ketone which is also a major component of tea flowers (Dong et al., 2016; Guo et al., 2025), commonly used in fragrances and in resin manufacturing and is used in synthesis of pharmaceuticals. Importantly, the studied aromatic carbonyls are also primary oxidation products of the most abundant substituted aromatics in the atmosphere, i.e., BZ is a primary product of toluene oxidation, PA is a primary product of ethylbenzene oxidation, and ACP is a primary product of cumene oxidation, all having above 6% yield according to the Master Chemical Mechanism (MCM). Thus, it is important to note here that while these VOCs are arguably common in many gas-phase environments, hydrocarbons are rarely speciated in ambient field measurements, except potentially the ones with unique detection characteristics such as a specific chemical composition, e.g., isoprene, benzene and methanol. Hence, a reliable atmospheric concentration range is hard to come by, and generally some inference is needed. The scarce earlier studies we could find report ambient concentrations below 1 ppb for these compounds, e.g., 0.04 ppb ACP pointed by the reviewer, and 0.4 ppb for BZ and 0.4 ppb for ACP as reported by Uebori and Imamura (2004). Note that we did not observe an enhancement effect on HOM yields from BZ oxidation in the presence of NO in the current study. Especially near emission sources, the concentrations of aromatic carbonyls can reach several ppb (see Table 2 below). Furthermore, the indoor concentrations of aromatic carbonyls, including ACP form consumer products and electronics can be several orders of magnitude higher than the outdoor concentrations (e.g., 1.51–151 ppm ACP, converted from liquid phase concentrations in detergents and perfumes reported by Yang et al. (2023), using Henry's law constant H = 110 M/atm). These highlight the likely importance of the studied compounds across diverse urban outdoor and indoor environments. Table 1 shows the concentrations of ACP and BZ in ambient air in two locations while Table 2 shows the concentrations of PA, ACP, and BZ estimated from ethylbenzene, cumene, and toluene measurements, respectively, in various environments.

Table 1. Ambient concentrations of acetophenone (ACP) and benzaldehyde (BZ).

VOC	Location	Concentration		Reference
		μg/m^3	ppb	
ACP	Osaka, Japan (ambient air)	0.1–1.8	0.02–0.4	https://doi.org/10.2116/analsci.20.1459
BZ	,,	0.1–1.9	0.02–0.4	,,
,,	Hong Kong Polytechnic University (HKPU) campus	1.06	0.24	https://doi.org/10.1016/s1352-2310(01)00570-2

Table 2. Estimated concentrations of phenylacetaldehyde (PA), acetophenone (ACP) and benzaldehyde (BZ) as primary oxidation products of ethylbenzene, cumene, and toluene, respectively, in various environments.

VOC	Location	Concentration		Product (6%) ppb	Reference
		μg/m^3	ppb		
Ethylbenzene	Sweden (urban air)	6.2–100	1.43–23.02	PA (0.09–1.4)	https://doi.org/10.1016/0160-4120(85)90033-9
,,	Rio de Janeiro (parking area, indoor)	12.8–100.2	3.0–23.7	PA (0.2–1.4)	https://doi.org/10.1016/j.atmosenv.2015.01.013
,,	Gothenborg, Sweden (automobile)	17.9	4.12	PA (0.25)	https://doi.org/10.1016/0048-9697(91)90359-M
Cumene	Urban (US and non-US)	0.1–900	0.02–183	ACP (0.001–11)	NCBI https://www.ncbi.nlm.nih.gov/books/NBK590648/
,,	Quebec, Canada (indoor)	0.1–45.48	0.02–9.3	ACP (0.001–0.6)	https://doi.org/10.1177/1420326X07089005
Toluene	US (urban air with traffic)	-	21.4–98.1	BZ (1.3–5.9)	NCBI https://www.ncbi.nlm.nih.gov/books/NBK592495/
,,	US (indoor)	10–150	2.7–39.8	BZ (0.2–2.4)	https://doi.org/10.60752/102376.28038902
,,	Japan (gas station)	196	52	BZ (3.1)	https://doi.org/10.1016/j.chemosphere.2019.01.166

Besides, urban atmospheric OH concentrations frequently reach above 1×10^7 molecules cm^{-3} (Lu et al., 2012; Ren et al., 2003; Kanaya et al., 2012) and even at this OH level and higher, the corresponding NO concentrations can be comparatively low (Carslaw et al., 2001; Creasey et al., 2001; Hofzumahaus et al., 2009) reducing the NO:VOC ratio (i.e., in practice the NO:RO_2 ratio).

Considering one order of magnitude higher aromatic carbonyl concentrations (0.4 ppb), a factor of two higher OH concentrations (1×10^7 molecules cm^{-3}) and a rate coefficient of 6×10^{-12} cm^3 $molecule^{-1}$ s^{-1} in ambient atmosphere, the carbonyl derived primary RO_2_C production rate is:

$(0.4 \times 10^{-9} * 2.46 \times 10^{19}) * 1 \times 10^7 * 6 \times 10^{-12}$

$= 5.9 \times 10^5$ molecules cm^{-3} s^{-1}

For 10 ppb of NO, the NO to RO_2_C formation ratio is only 2.5 times higher than in our experiments. If we consider 1 ppb of NO, the ratio is even lower than in our experiments. As speciated atmospheric hydrocarbons are rarely reported from field deployments, and even then generally only a handful of the most well-defined detections are included, it is easy to see how our view of the gas-phase compound variability and relevant concentration ranges is yet far from complete.

Moreover, we believe the reviewer acknowledges that despite the atmospheric RO_2_C production rate would be lower (i.e., considering low precursor VOC concentrations) than in our experiments, it would not halt completely the formation of HOMs but rather slow the HOM turnover. This is because we are dealing with branching from the RO_2_C + NO reaction and the rate coefficients, including those of the subsequent reactions, are the key that define the branching ratios towards various product paths. Our simulation results under atmospheric conditions including 1:1 NO to VOC ratios (i.e., 1 ppb NO conditions) show that the RO_2_C concentrations reach the peak values of around 1×10^6 molecules cm^{-3} (higher with PA) within 1 s reaction time (Figure S29 in the Supplementary Information). Note that the enhancement effect of NO via alkoxy-peroxy pathway is not included in our simulation because of lack of computational rate coefficients of specific RO_2 + NO → RO + NO_2 reactions for the studied systems. Furthermore, our experiments under 1:1 NO to VOC ratios (i.e., 1 ppm NO conditions) showed that the HOM yields increased in comparison to their yields under without NO conditions (Figure 4a in the manuscript, reproduced in Figure 0 below). For ACP, an increase in HOM yields (see Figure 0 below) was observed even at 2:1 NO to VOC ratio (i.e., 2 ppm NO condition, after which the NO interrupts the OH production to an extent that low signal levels make quantifying the signals more uncertain). We did not include this condition in the manuscript to keep consistency of NO range with all studied aromatic carbonyls.

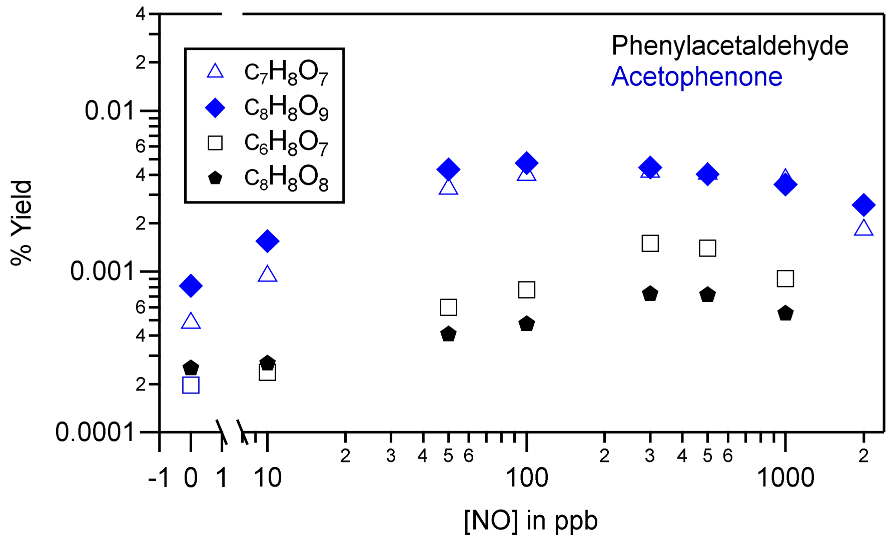

Figure 0. Enhancement of HOM yields in presence of NO during the OH initiated oxidation of phenylacetaldehyde (black) and acetophenone (blue). The yields of HOMs, detected by NO_3^--CIMS, as a function of initial NO concentration shown in a logarithmic scale.

Laboratory measurements

In the ambient the total daytime RO_2 is around 10^8 to 10^9 cm^{-3}. At remote atmospheres this is dominated by CH_3O_2 and HO_2 but closer to emissions and especially in urban locations larger RO_2 make a considerable portion of the budget. For individual RO_2 this partly self-limited concentration will generally stay small. To allow making measurements of the specific RO_2 reactivity under laboratory conditions, higher concentrations are generally necessarily needed for an appreciable signal. To track the first steps of the oxidation at short timescales, a prompt early [RO_2] is needed. This is a common procedure to be able to perform the experiments, though the extreme sensitivity of our detection method allows us to work very close to ambient relevant concentration regimes, as evidenced by the initial [OH] and hence the rather small [RO_2] used here. If RO_2 + NO reactivity is the target, then the concentrations must be set so that enough RO_2 is being produced at ambient relevant [OH], and the resulting RO_2 must be examined under high NO. There is a limit to what can be achieved under laboratory constraints.

This of course connects to making environmental chamber experiments, what the reviewer seems to be hinting here. Yet, it is important to note that absolutely no one is performing experiments (Danda et al., 2024; Henry et al., 2012; Surratt et al., 2009; Shilling et al., 2009) anywhere close to the concentration of RO_2 the reviewer is pointing at (i.e., the reviewer calculated the expected acetophenone related RO_2 production rate to be around 3×10^4 molecules cm^{-3} s^{-1}), and to study the reactivity in the laboratory higher concentrations are needed. One should also understand the complementary of the flow reactor and chamber environments, and necessarily somewhat different concentration regimes are required.

To reflect the concerns of the Reviewer on the used NO:VOC ratio, or rather the NO:RO_2_C ratio, we have now also toned down the wordings a bit in a few key places in the manuscript.

Changes to manuscript: In abstract lines 29–30 we added, "an improvement in our understanding of urban air ..." instead of "polluted air". Also, we removed the ending "especially in high NO_x environments"

In Section 2.1, lines 150–152, we replaced "The chosen NO range not only covers moderately polluted to highly polluted urban scenarios but also goes beyond any polluted ambient environment allowing us to observe the extent of the effect." by
"The NO range was chosen to maintain an experimental NO to VOC ratio up to 1:1 given that the usage of flow reactor platforms generally necessitates using high initial VOC concentration to produce appreciable initial RO_2 radicals."

In the last sentence of conclusions, lines 604–607, "Therefore, in contrast to traditional understanding of NO_x's suppressing effect on HOM, the current study highlights the importance of RO mediated pathways contributing to HOM and condensable material budget even in NOx rich polluted urban areas.", we now replaced the ending by "material budget in urban environments".

We thank the reviewer for going through our responses to reviewer's comments and the revised manuscript titled *"Nitric oxide enhances secondary aerosol precursor formation from aromatic carbonyls"*. We have incorporated all the suggestions and have modified the revised manuscript accordingly. Below, the reviewer comments are reproduced in red, followed by detailed author responses in blue, and the changes and additions to the revised manuscript in purple.

Reviewer #3 (Remarks to the Author):

The authors make a valuable contribution to the field of research around HOMs generation and the relationship with reactants of organic radicals.

In terms of the ratio of parent VOC reactivity with oxidants to NO concentration, the paper goes notably beyond the [NO] maximum of Wang et al. (2021), appears to go beyond that of Nie et al. (2023) and is comparable to Kang et al. (2025) (though comparisons are somewhat challenging given the changing ratios of reactants, including NO:NO2). In their rebuttal, the authors acknowledge that their minimum ratio may not be representative of highly polluted urban atmospheres, and have correspondingly removed references to highly polluted atmospheres in the main manuscript. However, I do not think the authors have revised the manuscript far enough given that the revised text of the manuscript still conveys a message that as NOx increases into concentrations relevant to polluted atmospheres, HOM yields from two aromatic carbonyls increases. Figure 4, combined with the author's calculations (in rebuttal) on the ambient relevance of NO to RO2_c formation ratio, proves that is clearly not the case. And rather than 'starkly contrasting the traditional understanding of oxidation suppression by NOx', I think the messaging should be that consistent with previous publications, increasing NOx from 0 leads to enhancement followed by suppression (relevant to the peak). If the authors want to contrast the value of [NOx] at which HOM yield peaks with previous studies, I think they will need to compare the RO2 formation rate:NO:NO2 ratio (maybe including other parameters in the ratio that the authors know about) between their study and previous studies.

Perhaps stated more clearly, given that:
i) using 1 ppm of NO in their experiments can give NO to RO2_c formation ratio 2.5 times less than in a polluted atmosphere, and
ii) that the HOM yields peak between 100 and 300 ppb of NO (giving a NO to RO2_c formation ratio 25 times less for 100 ppb NO than in polluted atmospheres (all else being the same)),

I think the authors need to point out that at NO to RO2_c formation ratios that can be relevant to polluted urban atmospheres, i.e. 300 ppb NO and above for their experiments, increasing NO is observed to have a suppressing effect on HOM yield, a trend consistent with previous studies finding a decreasing SOA yield and decreasing HOM accretion product yield at increasing NOx.

Simon P. O'Meara

Response: We thank the reviewer for the comments and suggestions. We have now explicitly mentioned the decreasing trend of HOM yields after reaching the peak values, especially under 300 ppb and higher NO conditions, in the relevant places.

In lines 11–12 in the abstract, we have now removed the ending “typical of polluted environments”.

Changes to manuscript: In lines 11–12, “However, the formation of HOM and SOA is thought to be suppressed by high NO_x ($NO + NO_2$) concentrations.”

In lines 19–22 in the abstract, we have replaced the ending “starkly contrasting the traditional understanding of oxidation suppression by NO_x” with “followed by a decreasing trend at above 300 ppb NO.”

Changes to manuscript: In lines 19–22, “Our experiments with varying high NO concentrations (10 ppb to 1 ppm) at 14 s reaction time show that phenylacetaldehyde and acetophenone HOM yields are enhanced by a factor of up to 10 for O_7 HOMs, followed by a decreasing trend at above 300 ppb NO.”

In lines 591–594 in the conclusions, we have also acknowledged the suppression of HOM at higher NO levels relevant to polluted urban areas.

Addition to manuscript: In lines 591–594, “However, at NO levels above 300 ppb—where the [NO]/[RO_2] ratio for aromatic carbonyls is closer to polluted urban atmospheres—we observe a suppression of HOM formation and a corresponding decrease in yields with increasing NO.

In the last sentence of conclusions (lines 605–607), we have removed “in contrast to traditional understanding of NO_x’s suppressing effect on HOM,” and modified the sentence as below.

Changes to manuscript: In lines 605–607, “Therefore, this study highlights how NO modulates the importance of RO-mediated pathways contributing to HOM and condensable material budget in urban environments.”

The manuscript entitled *"Rapid formation of aerosol precursors from the autooxidation of aromatic carbonyls and the remarkable enhancing influence of NO addition"* investigates HOM formation in the OH-initiated oxidation of three aromatic carbonyls through systematic flow-tube experiments with varying reaction times and NO injection. The study also connects the experimental results with quantum chemical calculations. The authors demonstrate that NO injection significantly enhances HOM formation through the alkoxy pathway. The experiments are well designed, and the data analysis and manuscript are clearly presented and organized. However, I still have some concerns. In particular, I find that certain quantitative comparisons are missing, which I think is crucial for you major conclusion. I recommend the authors address these issues before the paper can be considered for publication. My specific comments and concerns are outlined below.

Major concern: (authors should address it seriously).

The paper discusses HOM yields, which depend not only on the HOM detected by CIMS but also on precursor consumption. By definition, yield = Δ(HOM) / Δ(precursor). The figures present normalized HOM signals, which I assume are normalized to reagent ions. However, without accounting for precursor consumption, these normalized signals cannot be directly compared across different systems when discussing yields. I suggest authors address yields comparison by considering the consumption of precursors.

Other comments

1. **Lines 50–52:** I recommend citing a reference here when discussing the fate of RO_2 radicals. How does the OH radical influence the fate of RO_2?
2. **Lines 56–57:** Please also cite a paper for the definition of HOM (e.g., Federico Bianchi, 2019, *Chemical Reviews*).
3. **Line 73:** Since NOx is already mentioned in line 51, I suggest moving the description of NOx up to line 51 for clarity.
4. **Lines 82–83:** Please revise the description of the fate of alkoxy radicals. RO radicals first undergo isomerization with rapid rates, followed by O_2 addition to form RO_2, which can then undergo further autooxidation (see: *Formation of highly oxygenated organic molecules from α-pinene photooxidation: evidence for the importance of highly oxygenated alkoxy radicals*, ACP 2025).
5. **Experimental setup:**
   - Please provide more details on your CIMS setup, as these data are central to your study: manufacturer, LToF or HToF, mass resolution, and inlet type.
   - How were VOC and NOx concentrations measured? Please include this in the main text.

- In the H/D section, it is unclear how water influences the chemistry and how H is substituted by D. I suggest adding water-related chemical reactions in the manuscript or SI.

6. **Lines 139–140:** When D_2O was used, did the reagent ion $HNO_3 \cdot NO_3^-$ become $DNO_3 \cdot NO_3^-$? If so, does this imply that HNO_3 in the $HNO_3 \cdot NO_3^-$ cluster originates from chemical formation in the flow tube rather than nitric acid vapor from the ion source?
7. **Figure 2:** This figure combines fragments, non-fragments, and accretion products, making it visually busy and difficult to extract the key message. I suggest grouping compounds into families to provide clearer, zoomed-in insights (e.g., separate C_6HxOx, $C_7H_{8–10}Ox$, C_{14}, C_{13}, and C_{12} families).
8. **Lines 298–299:** The appearance of closure products with odd oxygen numbers does not necessarily prove the involvement of alkoxy steps. For example, $RO_2 + RO_2$ reactions between even-O species can also yield odd-O products (e.g., $C_8H_9O_8 + C_8H_9O_8 \rightarrow C_8H_8O_7 + C_8H_{10}O_7 + O_2$). You should either show that $RO_2 + RO_2$ is negligible relative to $RO_2 + HO_2$, or explicitly discuss the oxygen number in the RO_2 species.
9. **Lines 328–330:** Could you compare the relative roles of bimolecular versus unimolecular reactions across systems? For instance, in sub-second experiments, are RO_2 concentrations comparable? Table 1 shows differing precursor levels (1, 1.5, 3 ppm for benzaldehyde, phenylacetaldehyde, and acetophenone). In Figure S21a, RO_2 in the PA_OH system appears at least two orders of magnitude lower than in ACP_OH, suggesting that $RO_2 + RO_2$ reactions might be more important in ACP_OH. Could this explain the limited HOM formation in ACP_OH short-time experiments (Figure 1c)? Also, note the inconsistency between RO_2 concentrations in Table S8 and Figure S21a. In Table S8, the three short-time experiments show comparable RO_2 levels.
10. **Line 347:** Please provide an estimate of the NO remaining in the flow tube, or how much NO participates in the reactions.
11. **Lines 350–352:** Please specify precursor consumption in each system, especially when comparing across precursors.
12. **Figure 4:** Panel 4b shows negligible nitrophenol formation in the acetophenone and phenylacetaldehyde systems, but Figures 3b and 3c suggest more significant formation. What accounts for this discrepancy? If differences are indeed large across the three precursors in Figure 4b, a log-scale y-axis would help. Also, keep naming consistent (e.g., “acetophenone” in panel a vs. “$PhCOCH_3$” in panel c).
13. **Figures 1–3:** The y-axis shows normalized signals. The unit should be *ncps*, not *counts/s*.
14. **Volatility discussion:** When discussing the relative contributions of different families with varying volatility, please clarify that HOM sensitivity is assumed equal due to their high

oxidation and functionalization. However, prior work (Stéphanie Alage, 2024, *AMT*) showed that 4-nitrocatechol had the highest sensitivity, implying nitrophenols may have enhanced sensitivity. Could this explain the strong nitrophenol signals in your study? Please discuss the potential influence of sensitivity differences on your results and conclusions.

The authors have largely done an excellent job at addressing the reviewer's comments. I appreciate the substantial investment in time and effort. The results are beneficial to the scientific community and need to be published, however, as detailed below, I am not convinced the signficance is sufficient for Nature Communications.

There remains a major issue with the aspect around the significance of the results for the urban ambient atmosphere, specifically whether the molecular concentration ratio of NO to RO2 from aromatic carbonyls does indeed reach the ratios relevant to the urban atmosphere. The authors state that the NO range covers moderately polluted to highly polluted urban scenarions (section 2.1), whilst in the abstract and conclusion they say that results are relevant to our understanding of polluted air, therefore this aspect is key to the study's stated significance.

In their response to referee 3 comment:

'Method in general – the NO:VOC ratio for the investigated VOCs is likely to be greater than 1 throughout the troposphere. However, the experiments here do not exceed 1. Could the authors therefore explain how their experiments are relevant to the troposphere, and particularly urban areas? Similarly, could the authors explain how the method can provide evidence that even at high NO:VOC concentration ratio, HOM yields are relatively high?'

they respond stating that OH is the limiting reactant to the formation of primary aromatic carbonyl produced RO_2 radicals (RO2_C). I agree, I think the the relative strength of NO reaction with RO2_C against the strength of RO2_C production is key.

Below I provide some calculations to show why I think the rate of NO reaction with RO2_C in the experimeriments is too low relative to the production rate of RO2_C to be representative of the urban atmosphere:

Taking acetophenone as an example, where the literature review at Acetophenone | C6H5COCH3 | CID 7410 - PubChem reports an atmospheric mixing ratio of 0.04 ppb in the US.

And assuming a concentration of OH in ambient air of $5x10^6$ molecules cm^{-3} (Tropospheric OH and HO 2 radicals: field measurements and model comparisons - Chemical Society Reviews (RSC Publishing) DOI:10.1039/C2CS35140D).

And using the rate coefficient equation from the Master Chemical Mechanism v3.3.1 for acetophenone+OH of $6x10^{-12}$ cm^3 $molecule^{-1}$ s^{-1}:

The reaction rate of VOC with OH to produce RO2_C is expected to be:

[ACETOPHENONE (molecules cm^{-3})][OH (molecules cm^{-3})]$6x10^{-12}$

Under the ideal gas law at temperature (298.15 K) and pressure (101325 Pa) relevant to the ambient atmosphere and the flow reactor experiments there is a total concentration of molecules in air of $2.46x10^{19}$ molecules cm^{-3}.

Under ambient conditions the VOC+OH reaction rate is therefore:

$(0.04x10^{-9}*2.46x10^{19})*5x10^6*6x10^{-12}$

= approximately $3x10^4$ molecules/cm^3/s for the rate of RO2_C production in the ambient urban atmosphere, whilst NO is typically around $2.46x10^{11}$ molecules cm^{-3} (10 ppb) in urban atmospheres.

Under the experimental conditions here, the reactivity of VOC with OH is dominated whilst [OH] is relatively high in the first 4 seconds of the experiment, with Fig 26 giving a conservative value around $1x10^6$ molecules cm^{-3}, and Table 1 showing 1 ppb of VOC. The resulting RO2_C production rate is:

$(1x10^{-6}*2.46x10^{19})*1x10^6*6x10^{-12}$

= approximately $1.5x10^8$ molecules/cm^3/s whilst NO has a maximum of $2.46x10^{13}$ molecules/cm^3 (1 ppm) in experiments.

Therefore, the production rate of RO2_C is approximately 5000 times higher in experiments than in the ambient atmosphere. However, the NO is increased approximately 100 times higher in experiments than in the ambient atmosphere.

For comparison against an urban atmosphere of 10 ppb NO, and using the values for acetophenone and OH concentrations above, the NO mixing ratio used in these experiments is therefore a factor of 50 too small to match the ambient condition. Even for a relatively low NO_x location such as Nordic urban areas with a NO value of 1 ppb, the NO mixing ratio used in these experiments is a factor of 5 too small.

Therefore, whilst the results presented here may be useful for scenarios of relatively high VOC to NO_x emission ratio, which may be true in some current non-urban areas, and may possible in future scenarios of urban areas, I do not see how the results can be said to apply to current or past urban areas.

Unless the authors can satisfactorily address this issue, I suggest the significance of the paper is insufficient for publication in Nature Communications.

Simon P. O’Meara
